# Supramolecular interactions between ethylene-bridged oligoureas: nanorings and chains formed by cooperative positive allostery†

**DOI:** 10.1039/d2sc04716k

**Published:** 2022-10-25

**Authors:** David P. Tilly, Matej Žabka, Inigo Vitorica-Yrezabal, Hazel A. Sparkes, Natalie Pridmore, Jonathan Clayden

**Affiliations:** School of Chemistry, University of Bristol Cantock's Close Bristol BS8 1TS UK j.clayden@bristol.ac.uk; Department of Chemistry, University of Manchester Oxford Road Manchester M13 9PL UK david.tilly@manchester.ac.uk

## Abstract

Ethylene-bridged oligoureas are dynamic foldamers in which the polarity of a coherent chain of intramolecular hydrogen bonds may be controlled by intra- or intermolecular interactions with hydrogen-bond donors or acceptors. In this paper, we describe the way that supramolecular interactions between ethylene-bridged oligoureas bearing a 3,5-bis(trifluoromethyl)phenylurea (BTMP) terminus leads to higher-order structures both in the crystalline state and in solution. The oligoureas self-assemble by head-to-tail hydrogen bonding interactions to form either supramolecular ‘nanorings’ with cyclic hydrogen bond chain directionality, or supramolecular helical chains of hydrogen bonds. The self-assembly process features a cascade of cooperative positive allostery, in which each intermolecular hydrogen bond formation at the BTMP terminus switches the native hydrogen bond chain directionality of monomers, favouring further assembly. Monomers with a benzyl urea terminus self-assemble into nanorings, whereas monomers with a *N*-ethyl urea terminus form helical chains. In the crystal state, parallel helices have identical handedness and polarity, whereas antiparallel helices have opposite handedness. The overall dipole moment of crystals is zero due to the antiparallel arrangements of local dipoles in the crystal packing. Supramolecular interactions in solution were also examined by VT and DOSY NMR spectroscopy, up to the point of crystal formation. The size of higher aggregates in dichloromethane was estimated by their hydrodynamic radius. The relative orientation of the monomers within the aggregates, determined by 2D ROESY NMR, was the same as in the crystals, where *syn*-orientations lead to the formation of rings and *anti*-orientations result in chains. Overall, the switch of hydrogen bond polarity propagates intermolecularly in crystal and solution states, constituting an example of intermolecular communication within supramolecular polymers.

## Abstract 2

Hydrogen-bonded urea oligomers form supramolecular aggregates in the crystalline state. Intermolecular hydrogen bonding generates nano-rings or chains, according to the length and substitution pattern of the oligomers.

## Graphical abstract

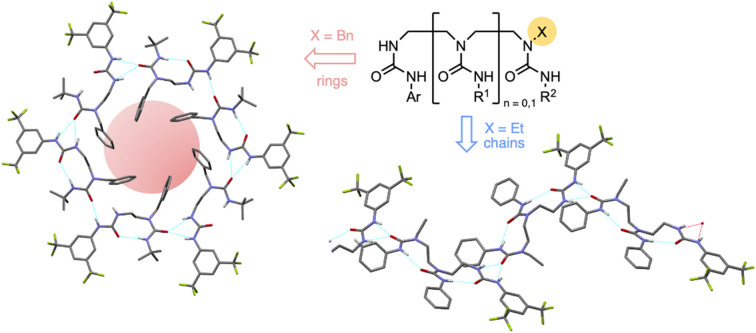

## Introduction

Ethylene-bridged oligoureas^1–3^ are a family of ‘dynamic foldamers’^4^ that show considerable conformational flexibility in solution, but whose structure is nonetheless governed by a single coherent chain of hydrogen bonds that locks every urea in the oligomer into a dipole directionality.^5^ The chain of hydrogen bonds is robust, of reversible directionality, and we have shown that an induced switch in the directionality of the hydrogen bond chain may be exploited as a mechanism for communicating information and for remotely induced bind-and-release cycles.^6^ Previous work employed computational methods to deduce conformational flexibility in solution.^5^ In this paper we give an account of the conformations adopted by this family of oligomers in the solid state, and of the supramolecular interactions they engage in both in the solid state and in solution.

## Results and discussion

**Scheme 1:**
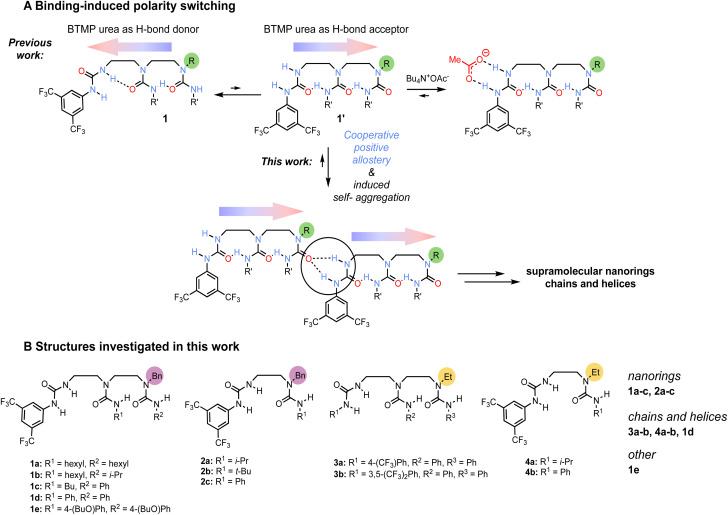
(A) Polarity switching of oligoureas with 3,5-bis(trifluoromethyl)phenylurea (BTMP) terminus (the coloured arrows indicate the polarity in the intramolecular hydrogen-bond chain). In the dominant conformation 1 in solution, BTMP urea acts as a hydrogen-bond donor. Upon addition of acetate, the BTMP urea binds the anion which induces the hydrogen-bond chain polarity switch, and activates the hydrogen-bond acceptor capability of the last urea in the chain (conformation 1′). In this work, we explored the conformational equilibrium between 1 and 1′ to induce self-aggregation in solution and solid phase, ultimately giving polarity-ordered supramolecular structures without any templating. (B) Structures of the investigated oligoureas.

Earlier work^5,6^ on ethylene-bridged oligoureas made of identical alkyl- or aryl-carbamoyl groups employed a terminal 3,5-bis(trifluoromethyl)phenylurea (BTMP)^7^ of differing hydrogen bond donor capabilities to control the oligoureas polarity. In non-polar solvents and diluted conditions, the BTMP urea forms an intramolecular hydrogen bond between its relatively acidic NH group with the carbonyl of the directly adjacent urea in the chain (Scheme 1A, conformation 1), the local preferred conformation is relayed along the chain through intramolecular hydrogen-bonds. On addition of acetate anion, the BTMP urea acts as a hydrogen-bond donating binding site for the anion and adopts an alternative conformation 1′ in which it engages with the adjacent urea in the chain as a hydrogen-bond acceptor, leading to a global polarity switch of the hydrogen-bond chain. We reasoned that the BTMP urea may also induce the formation of supramolecular self-aggregates,^8^ by head-to-tail interactions, raising the possibility of intermolecular communication of information through induced polarity switching.^9^

Several ethylene-bridged oligoureas bearing a BTMP urea terminus were analysed by X-ray crystallography (Scheme 1B), varying the remote urea group of the chain to assess the consequences for self-aggregation in the crystalline state. Crystals were grown by slow evaporation from a hot filtered solution of acetonitrile allowed to stand at room temperature.

### Nanorings

**Fig. 1:**
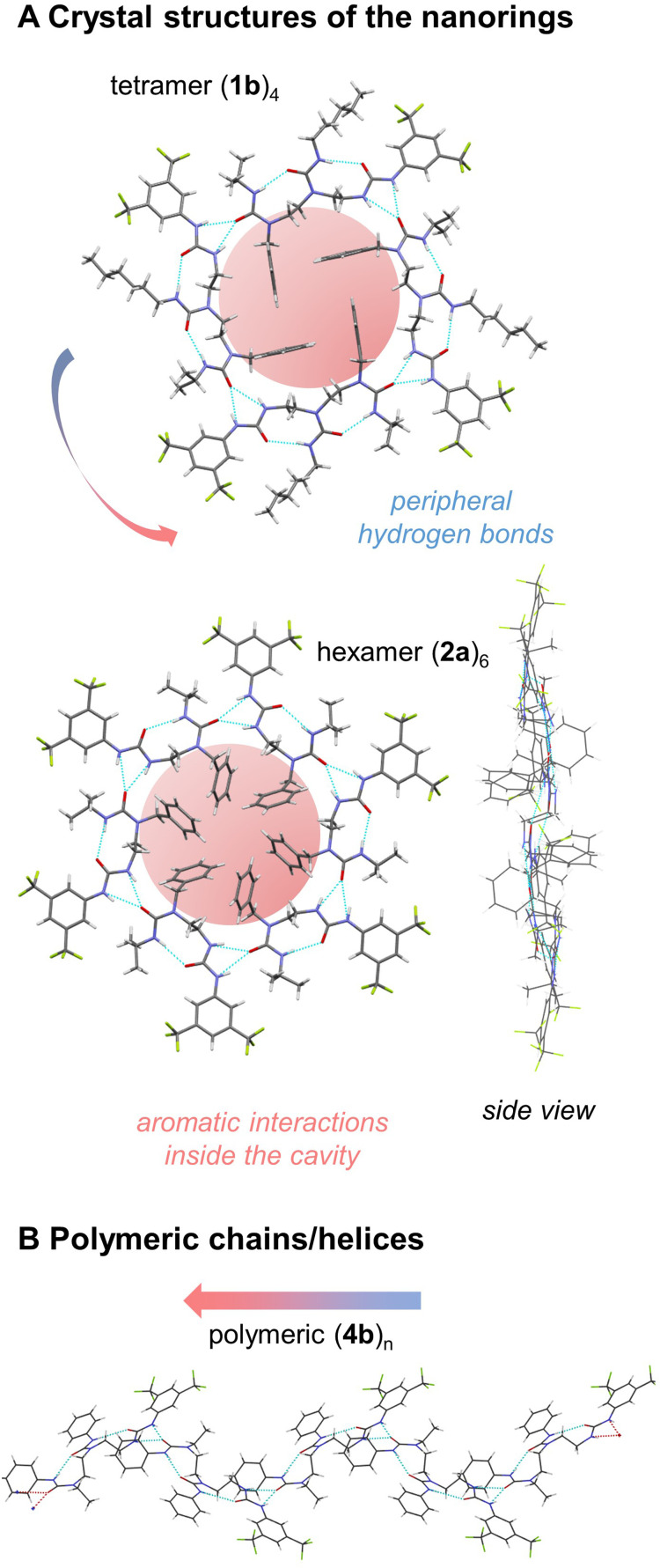
(A) Crystal structures of the nanorings: tetramer (1b)_4_ and hexamer (2a)_6_. Hydrogen bonds are located on the periphery, while aromatic interactions inside the cavity stabilize the structure. The overall dipole moment of the rings is zero. The arrangements of the monomers is *syn* with respect to each other. (B) Polymeric helical chain of hydrogen bonds with polarity directionality (4b)_*n*_. The mutual arrangement of the monomers is *anti*. Aromatic interactions associated with the benzyl groups are absent, and the nanorings do not form. The dipole moment in the crystal is minimized as a result of equal number of parallel and antiparallel chains.

Crystal structures of tris(ureas) 1a–c were examined. All three molecules crystallise in the triclinic space group *P*1̄. In the crystals, four molecules associate by head-to-tail interaction and *syn*-orientation to form a supramolecular ‘nanoring’ (Fig. 1A, ESI 1–3†).^10–12^ The formation of an intermolecular bifurcated hydrogen bond between the relatively acidic NH group of the BTMP urea and the C=O group of the terminal urea in the chain of another molecule results in a local intramolecular conformational preference at the BTMP urea that propagates intramolecularly by formation of an hydrogen bond between the C=O group of the BTMP urea and the NH bond of the adjacent urea in the chain. The resulting directionality of the intramolecular hydrogen bond chain in the self-aggregate is opposite to that observed in solution.^5,6^ The supramolecular macrocyclic tetramers have a centre of symmetry and feature an uninterrupted ring of twelve hydrogen bonds. The benzyl substituents are located inside the macrocycle in parallel pairs, with the urea side chains located outside each macrocycle. The hydrogen bond chains have cyclic directionality, and remarkably the cyclic directionality is identical in all the rings of the crystal. This unidirectional sense of rotation most likely stems from local dipole–dipole interactions between rings, in which individual urea groups of adjacent nanorings adopt an antiparallel arrangement.

Despite the well-established propensity for intermolecular hydrogen bonding in ureas and thioureas,^13,14^ this is the first report of such nanorings in the crystals of a urea or thiourea structure. A crystal structure composed of a single benzene molecule surrounded by a hexamer of hydrogen-bonded cyclohexane-1,3-dione enols is reminiscent of the structures we report, but without the question of hydrogen-bond directionality.^15^

The supramolecular nanorings form without templating. The self-aggregation process features a mechanism of cooperative positive allostery,^16^ as the molecules have dynamic ditopic features with terminal binding sites increasing their binding affinity upon aggregation. The BTMP urea sets a native conformation of molecules in diluted solution, but also programs the molecules to engage in intermolecular head-to-tail hydrogen bonds in concentrated solutions. Each intermolecular hydrogen-bond formation at the BTMP site induces intramolecular conformational changes that reverse the polarity of the opposite chain terminus, activating it to become a hydrogen-bond acceptor, dynamically increasing its affinity for binding at another BTMP urea in an allosteric fashion. The crystals also feature intermolecular communication of information between oligoureas, linearly transmitting information along the supramolecular hydrogen-bond chain by polarity switch.

A further series oligoureas, namely the bis(ureas) 2a–c, were crystallised. Again, supramolecular nanorings formed in the crystalline state (Fig. 1A, ESI 4–6†). This time, rather than four molecules, six bis(ureas) associate in a head-to-tail arrangement to form the supramolecular nanorings. In these structures, adjacent molecules associate through intermolecular bifurcated hydrogen bonds between the most acidic BTMP NH and the carbonyl group of the last urea of the chain. The benzyl substituents were again located inside the ring, with the benzyl groups alternating above and below the average plane of the ring, and their urea side chains lie outside the ring. Each ring again contained 12 continuous hydrogen bonds, identical to the number in the nanorings obtained with longer tris(ureas). The internal diameters of the hexamer nanorings with 2a–c vary from 13.4 to 14.5 Å, slightly larger than the diameters of the tetramer nanorings formed with 1a–c (12.4 Å) (see Fig. ESI 1–6†).

The attractive, weak noncovalent π,π-interactions between the inner benzyl rings of 2a are recognized by NCIplot^17,18^ (see Fig. ESI 52†) and appear to contribute to the overall stabilization of the nanoring (hydrogen atoms positions optimized by B97-3c/def2-mTZVP).^19^ Indeed, the attractive dispersion interaction^20^ was calculated to be −75 kJ·mol^−1^ between the adjacent units by HFLD/def2-TZVP^21^ and appears six times in the nanoring, which could assist during the structure assembly in the crystal formation process (see Tables ESI 12 and 13†). Ring formation is also assisted by minimisation of the dipole moment. The computed net dipole moment of both 2a and its hydrogen bonded dimer are both rather large (6.9 and 16.6 Debye), but this value reduces to zero on formation of the nanoring.

For 2a and 2c, the sense of rotation of hydrogen-bond rings are identical in all the rings in the crystal, but 2b (*tert*-butylurea), which crystallises in the monoclinic space group *P*2_1_/*n*, displays alternating clockwise and anticlockwise hydrogen-bond directionalities in adjacent ‘sheets’ of nanorings. The steric hindrance of the ^*t*^Bu group that terminates this urea is apparently incompatible with the stacking pattern adopted by 2a and 2c and an alternative packing of nanorings occurs for 2b. The nature of the carbamoyl substituents (alkyl or phenyl) in the monomers does not impact the overall shape of the individual nanorings (see Fig. ESI 1–6†).

Homologous (tetrakis)urea oligomers were synthesized (Scheme ESI 4†) to investigate the formation of supramolecular nanorings by trimeric self-association, however the compounds did not crystallise under the various conditions attempted.

### Chains and helices

By contrast, compounds 3a, 3b and 4a, 4b, having an *N*-ethyl instead of an *N*-benzyl terminus, crystallise in the monoclinic space group *P*2_1_. The molecules self-associate in head-to-tail fashion adopting an alternating *anti*-orientation to form extended helical chains of continuously hydrogen bonded ureas as self-assembled supramolecular polymers (Fig. 1B).^22–24^

The directionality of intramolecular hydrogen bond chain is again opposite to that observed in solution: the carbonyl group of each ethylureido group forms intermolecular hydrogen bonds with the acidic BTMP NH groups of another molecule, which activates by intramolecular polarity switch the ditopic capability of the molecules. The supramolecular chains propagate conformational preferences along extended distances beyond the length of individual molecules, constituting a prototype of intermolecular communication of information in a supramolecular polymer. Each supramolecular chain is unidirectional. The supramolecular chains run either parallel or antiparallel with respect to each other in the crystal, with the macrodipole of the chain cancelled by the macrodipole of a directly adjacent antiparallel supramolecular chain. Parallel helices have identical handedness and polarity, whereas antiparallel helices have opposite handedness and polarity.

1d, the analogue of 1c with phenylcarbamoyl inner substituents, crystallises in the monoclinic space group *C*2/*c*, forming supramolecular helical chains of continuously hydrogen bonded ureas rather than nanorings. The molecules self-associate in head-to tail arrangement adopting an *anti*-orientation. This difference in supramolecular architecture (helical chains instead of nanorings) may result from unfavorable steric interactions in the adjacent ‘sheets’ of the crystal. Transposing the crystal packing topology of nanorings of 1c (R^1^ = Bu, R^2^ = Ph) to nanorings that would result from its analogue 1d (R^1^ = Ph, R^2^ = Ph), a steric clash would occur between the 1d carbamoyl R^1^ = Ph with the inner benzyl groups of nanorings located in the adjacent ‘sheets’. Since the benzyl groups participate to stabilising the nanoring structures, the steric interactions likely explain the alternative helix arrangement observed in crystals of 1d. Nanorings are formed with 2c that is a shorter analogue of 1d, nanorings of bis(ureas) 2a–c overlay off-centred in the crystal packing, leading to low steric interactions between the carbamoyl substituents and the inner benzyl groups of the nanorings. Nanoring of tris(ureas) 1a–c are less off-centred in the crystal packing, leading to more steric interactions between the inner benzyl groups of nanorings with the carbamoyl substituents in (tris)ureas.

1e, an analogue of 1d with *p*-^*n*^BuOPh carbamoyl substituents, form crystals in which the molecules self-aggregate without maintaining a coherent chain of intramolecular hydrogen-bonds, and does not form nanorings nor chains.

The dihedral angles of ethylene bridge N–C–C–N bonds are of similar values in the supramolecular chains and in the nanorings (from 152° to 162°, see Table ESI 1†). Molecules adapt their mutual orientation at the bifurcated hydrogen bond positions rather than by a change of dihedral angles at the N–C–C–N bonds to spatially accommodate the carbamoyl substituents to form the supramolecular structures. *Syn*-orientation of monomers form nanorings whereas *anti*-orientation of monomers form supramolecular chains and helices.

### Aggregation in the solution phase

**Fig. 2:**
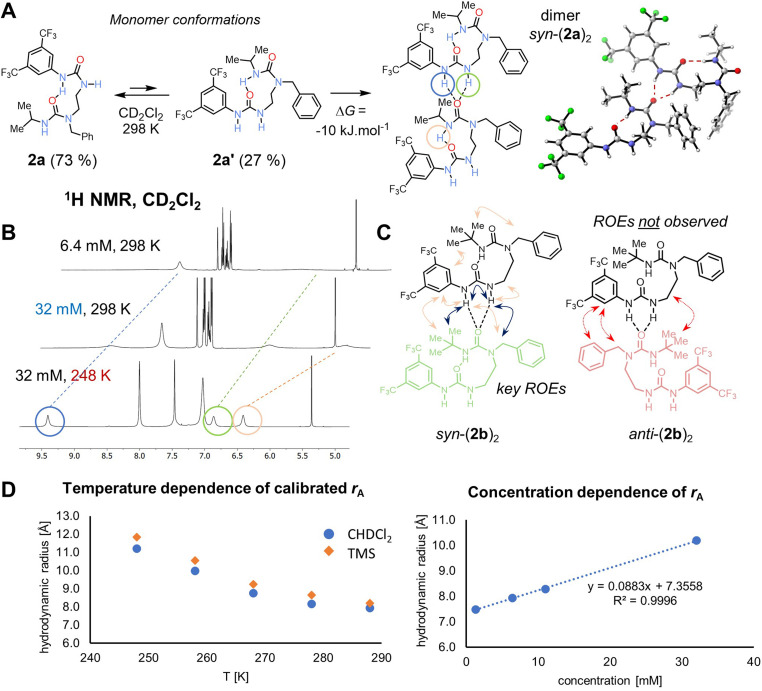
(A) Main equilibrium conformations of monomer 2a and their populations in solution as calculated by B3LYP-D3(BJ)/def2-TZVPP/SMD(CH_2_Cl_2_)//B3LYP-D3(BJ)/def2-SVP. The dimerisation process is slightly exergonic based on both experiment and computations. Right: computed 3D model of the *syn*-dimer of 2a based on its crystal structure. (B) ^1^H NMR spectra of 2a (500 MHz, CD_2_Cl_2_) showing the oligomerization with both increasing concentration and decreasing temperature. Hydrogen-bonded protons are circled. (C) Key ROEs for 2b (shown in blue, 500 MHz, CD_2_Cl_2_, 258 K) observed matching the *syn*-isomer, whereas key ROEs for the *anti*-isomer are missing for the dominant compound. Similar pattern was observed for 2a. (D) Dependence of calibrated hydrodynamic radius *r*_A_ of 2a on temperature (at 6.4 mM) and concentration (at 288 K), hinting at the monomeric species at infinite dilution at room temperature.

The formation of nanoscale structures in the solid phase prompted us to investigate by NMR whether similar aggregates form in solution (Fig. 2A). Intermolecular interactions were evident in the form of the NH signals, which are broad in dilute samples but become sharper with increasing concentrations or decreasing temperature (circled signals in Fig. 2B), both of which would promote intermolecular association by hydrogen bonding. Because of fast exchange relative to NMR time scale, the peaks in the corresponding ^1^H NMR spectra represent the averaged signals from individual components (mono- and oligomers) of the mixture.

Further information was obtained using diffusion-ordered spectroscopy (DOSY), which is well-suited to explore self-aggregation in solution.^25,26^ DOSY experiments using a convection-compensated pulse sequence^27^ with (bis)urea 2a revealed a linear concentration dependence of extracted molecular mass and of hydrodynamic radius over a range of concentrations (1.6–32 mM, CD_2_Cl_2_, 288 K), indicating an aggregation process (see Fig. 2D). This process is also observed upon cooling of the sample in the range 288–248 K (*D* = 8.94 to 3.50 × 10^−10^ m^2^ s^−1^). Similar aggregation and supramolecular stabilization at low temperature caused by noncovalent interactions has been reported.^28,29^ Hydrodynamic radius was extrapolated to a value of 7.35 Å in infinitely diluted solution at 288 K, roughly corresponding to a monomeric structure, while at 32 mM, it is 10.19 Å. We estimate that at 288 K, the aggregation number of 2a at 6.4 mM concentration is 1.26, implying that the dominant species is a monomer, whereas at 248 K it increased to 3.53. We could not cool the sample further due to instrument limits and line broadening.

To detect higher aggregates, leading potentially to a hexamer structure observed in the solid state, we prepared a 54 mM sample of an analogous compound 2b in CD_2_Cl_2_, in which the change of i-Pr to a *t*-Bu substituent led to increased solubility. The extracted hydrodynamic radius reached value 13.12 Å in this case (*D* = 2.89 × 10^−10^ m^2^ s^−1^), suggesting that a tetramer is dominant in the population of aggregates. At this temperature for highly concentrated samples, the compound starts to crystallize out. Similar behaviour was noted in halogen-bonded complexes.^30^ Once the molecule self-associates to reach a reasonable concentration of the hexamer, the crystals emerge.

The association constant in CD_2_Cl_2_ was estimated using the equal *K* model^25^ to be *K* = 96, which corresponds to Δ*G* = −10.9 kJ·mol^−1^ at 288 K (see Fig. ESI 38†). This value matches the DFT computed value of Δ*G*_solv,calc_ = −10.2 kJ·mol^−1^ (B3LYP-D3(BJ)/def2-TZVPP/SMD//B3LYP-D3(BJ)/def2-SVP level of theory) for the dimerization process.

Interestingly, a highly concentrated sample of 2a in acetone-*d*_6_ (25 mM) did not show aggregation with decreasing temperature: a constant molecular mass of around 500 g mol^−1^ (nominal mass 490 g mol^−1^) was calculated from the DOSY data (see Fig. ESI 41 and 42†). This is a strong indication that in a solvent that can compete as a hydrogen-bond acceptor, aggregation no longer takes place.

Overall, these experimental data prove substantial self-association in dichloromethane. However, we were not able to identify the formation of a nanoring comprised of six distinct molecules of 2a or 2b in solution. This fact might be due to the solvent-attenuated or cancelled dispersion interactions^31^ between the benzyl rings in solution by the solvent, in contrast to the crystal formation process. Indeed, as soon as the terminal benzyl substituents are changed into ethyl groups while conserving the rest of the oligourea structure, the nanorings do not form and oligomeric chains emerge instead. Presumably, the assembly in the solid phase is also driven by crystal packing,^32^*i.e.* involves other noncovalent interactions between the layers or chains (as evidenced by a NCI plot of antiparallel chains of 4b (see Fig. ESI 53†); dipole moment alignment/cancellation, *etc.*) that might favour the formation of the rings – a process which takes places also at low temperatures followed by immediate precipitation of the nanorings.

### Hydrogen bond directionality in solution

The H-bond directionality seen in the crystal structures, with the BTMP urea serving as an intramolecular H-bond acceptor rather than a donor, and the trisubstituted alkyl urea as an H-bond donor, is opposite to those deduced previously from solution-phase studies.^6^ Indeed, for bis(urea) 2a, the conformation of the crystal building block is the minor conformation in solution 2a′ as confirmed by DFT computations (Fig. 2A). The BTMP urea's ability to provide a binding site for intermolecular interaction with an adjacent urea carbonyl group evidently drives to the self-association process.

With regard to this intermolecular urea NH⋯O=C interaction, it is notable that in the crystal structures of nanorings, pairs of molecules adopt a *syn*-orientation (Fig. 1A and Fig. 2C), allowing the ring to form, whereas in the polymeric chains (Fig. 1B), an *anti*-orientation is observed, leading to a linear arrangement. To establish the relative orientation of two oligourea residues in a solution-phase dimer, we conducted 2D ROESY experiment of 2b in CD_2_Cl_2_ (52 mM) at 258 K. At these conditions, oligomers are formed. Multiple crosspeaks were detected in this experiment, indicating that the major binding orientation of a potential dimer or oligomer is *syn* (Fig. 2C). Similar results were obtained with 2a. DFT (B3LYP-D3(BJ)/def2-TZVPP/SMD//B3LYP-D3(BJ)/def2-SVP level of theory for 2a) suggested that the *syn*-dimer would be preferred over a potential structure of the *anti*-dimer by 15 kJ·mol^−1^. Local energy decomposition^33^ of the potential *anti*-dimer arrangement points at decreased binding energy in the gas phase – the intermolecular interactions are stronger but so is the electronic preparation higher due to more severe geometry distortion requirements. Indeed, aryl–aryl interactions between benzyl substituents are seen by VT NMR down to 248 K, where severe broadening of the signals due to their mutual interactions and conformational restrictions are noted, whereas the BTMP urea peaks remain sharp. For the major conformation, no ROE cross peaks were observed between the two different aryl rings, between the *t*-Bu group and the ethylene linker, or between the benzylic CH_2_ and BTMP ring, as would be expected in the *anti*-arrangement.

Additionally, a separate minor sharp H-bond peak appears at *δ*_H_ 10.50 ppm at low temperatures, which corresponds to an *E*,*Z*-disubstituted^34^*N*-aryl-*N*′-alkylurea monomer with a single intramolecular H-bond (conformation 2a, Fig. 2A, ∼3% population at 248 K). This compound is a major structure at room temperature (calculated 73% population) presumably in fast exchange with the oligomer-forming conformation (∼27% population), but the exchange rate is slowed down by cooling down and thus detectable by ROESY. Compared to room temperature, the population of conformer 2a is decreased by the competing aggregation process. Similar compounds have been observed.^6^ DFT-computed ^1^H chemical shift of the aryl NH intramolecular bond (TPSS/pcSseg-3/SMD) at *δ*_H_ 10.15 ppm matches the experimental value of 10.50 ppm.

The calculated ^1^H chemical shifts of the monomer and dimer of 2a suggest that upon binding of another molecule, the NH protons of the aryl urea moiety move downfield from *δ*_H_ 6.70 and 5.32 ppm to 8.88 and 7.60 ppm (experimental *δ*_H_ 8.05 and 6.29 to 8.84 and 6.43 ppm at 288 K). The shift of the alkyl urea NH should not change (calculated *δ*_H_ 7.46 ppm), however a shift is observed for the most upfield peak (experimental *δ*_H_ 5.14 to 6.41 ppm at 32 mM). This is reasonable: as the compound self-associates, the alkyl urea is also affected by the H-bonding.

In summary, we have shown the orientation of the bis(ureas) is *syn* in solution at low temperature, analogous to the orientation in the crystal structure for these compounds.

## Conclusions

Dynamic foldamers built from ethylene-bridged oligoureas exhibit global hydrogen bond directionality while retaining structural flexibility. This feature can be exploited for molecular communication, and a minor conformation with exposed binding site (BTMP urea) was shown previously to bind to anions and increase its population. In this work, the design of the oligoureas allows self-aggregation of this conformer to form oligomers in solution. These lead ultimately to nanorings and chains in the crystal structures. The nanorings and chains show uninterrupted hydrogen-bond directionality, comprised of 12 hydrogen bonds around the nanoring periphery. In this way, either four tris(ureas) or six bis(ureas) aggregate to form the nanoring. Benzyl substitution at the terminus distal to the binding site allowed the nanorings to be formed without templating due to noncovalent aromatic interactions in the cavity of the ring, whereas the absence of these benzyl substituents caused the formation of helical chains instead. The overall dipole moment in the nanorings is zero, the macrodipoles in the supramolecular chains mutually cancel out thanks to antiparallel arrangements in the crystals.

The oligomerization process was followed in solution by VT and DOSY NMR up to the point of the crystal formation. The relative orientation of the monomers within the oligomer was established to be the same in dichloromethane solution (ROESY NMR) and solid phase (X-ray crystal structure). *Syn*-orientation leads to the formation of the rings, while the *anti*-orientation usually results in chains. In a competing H-bond acceptor solvent such as acetone, the oligomerisation does not take place. Each intermolecular hydrogen bonding activates the ditopic behaviour of the monomers by polarity switching, triggering a cascade of cooperative positive allostery to form the supramolecular structures. The hydrogen bond chain polarity switch propagates through intermolecular interaction along distances beyond the length of individual molecules. Overall, the study demonstrates the ability of urea-based foldamers to perform intermolecular communication of information in a supramolecular polymer, both in solution and solid phase.

## Data availability

The datasets supporting this article have been uploaded as part of the ESI.†

## Author contributions

J. C. secured the funding, and directed the project. J. C., D. P. T. conceived the project, designed the molecules. D. P. T. carried out the chemical syntheses, carried out the structure characterisations, grew the single crystals. M. Ž. carried out the aggregation studies in the solution phase, performed the DFT calculations. I. V.-Y., H. A. S., N. P. carried out the crystal structure characterisations. J. C., D. P. T., M. Ž. wrote the manuscript.

## Conflicts of interest

There are no conflicts to declare.

## Supplementary Material

SC-013-D2SC04716K-s001

SC-013-D2SC04716K-s002
