# Supplementary material for: Supramolecular interactions between ethylene-bridged oligoureas: nanorings and chains formed by cooperative positive allostery
Source: Chem Sci. 2022 Oct 25;13(44):13153–9. doi: 10.1039/d2sc04716k (PMC9667931; doi:10.1039/d2sc04716k)
Supplement: SC-013-D2SC04716K-s001 [file SC-013-D2SC04716K-s001.pdf]

Electronic Supplementary Material (ESI) for Chemical Science.
This journal is © The Royal Society of Chemistry 2022

Supramolecular interactions between ethylene-bridged oligoureas: nanorings and chains by cooperative positive allostery

David P. Tilly,*[a,b] Matej Žabka,[a] Inigo Vitorica-Yrezabal,[b] Hazel A. Sparkes,[a] Natalie Pridmore[a] and Jonathan Clayden*[a]

[a] School of Chemistry, University of Bristol, Cantock's Close, Bristol BS8 1TS, UK
[b] Department of Chemistry, University of Manchester, Oxford Road, Manchester, M13 9PL UK

Supporting Information

Contents

1. **General Information** 2
2. **General synthetic schemes**.................... 3
3. **Procedures and Characterisation of compounds** 5
4. **^{1}H and ^{13}C NMR spectra of synthesised compounds**.................... 29
5. **Crystal data and structure refinement**.................... 76
6. **NMR studies** 104
7. **Computational studies** 126

1. General Information

All reactions were performed under a nitrogen atmosphere. All reagents and chemicals were obtained from chemical suppliers and used without further purification. Anhydrous solvents were dispensed under nitrogen from a solvent purification system (Innovative Technologies PureSolve PS-MP-5) or were purchased from commercial suppliers.

Thin-layer chromatography (TLC) was performed using pre-coated plates (Macherey-Nagel Polygram SIL G/UV254). Visualisation was achieved by way of UV light (at 254 nm), staining with either potassium permanganate, phosphomolybdic acid (in ethanol), or ninhydrin (in ethanol). Stained TLC plates were heated for visualization. Flash column chromatography was carried out either manually using Fluorochem 60 silica (40-60 µm particle size) or using an automated Biotage® Isolera Spektra Four with gradient elution on pre-packed silica gel Biotage® SNAP Ultra columns or ZIP Sphere columns. Nuclear Magnetic Resonance spectra (^{1}H NMR and ^{13}C NMR) were recorded on Bruker Nano 400, Jeol ECS 400 or Bruker Avance III HD 500 Cryo with 5 mm DCH ^{13}C–^{1}H/D Cryo Probe (500 MHz) spectrometers. All NMR characterisation experiments were performed at 25 °C. The spectra were plotted in Mestrenova 14.2.1 or TopSpin 4.1.4. Chemical shifts (δ) are quoted in parts per million (ppm) relative to the specified deuterated solvent. Spectra were calibrated using the residual solvent peaks for $CDCl_3$ (δH: 7.26 ppm; δC: 77.16 ppm), CD_2Cl_2 (δH: 5.32 ppm; δC: 53.84 ppm) and $(CD_3)_2SO$ (δH: 2.50 ppm; δC: 39.52 ppm) as appropriate. Coupling constants (*J*) are quoted in Hz and are rounded to the nearest 0.1 Hz. Splitting patterns are abbreviated to singlet (s), doublet (d), triplet (t), quartet (q), multiplet (m) or some combination thereof.

Infrared spectra were recorded on a Perkin Elmer Spectrum Two FT-IR spectrometer with samples applied as neat films. Absorptions maxima (vmax) of interest are quoted in wavenumbers as ν in cm-1 for the most intense bands.

High resolution spectrometry experiments (HR-MS) were recorded by staff at the University of Bristol on a Synapt G2S Waters for nanospray experiments, MicrOTOFII Bruker Daltonics and Orbitrap Elite Thermo Scientific for electrospray ionisation experiments.

Melting points were measured on a Stuart SMP10 melting point apparatus and are uncorrected.

2. General synthetic schemes

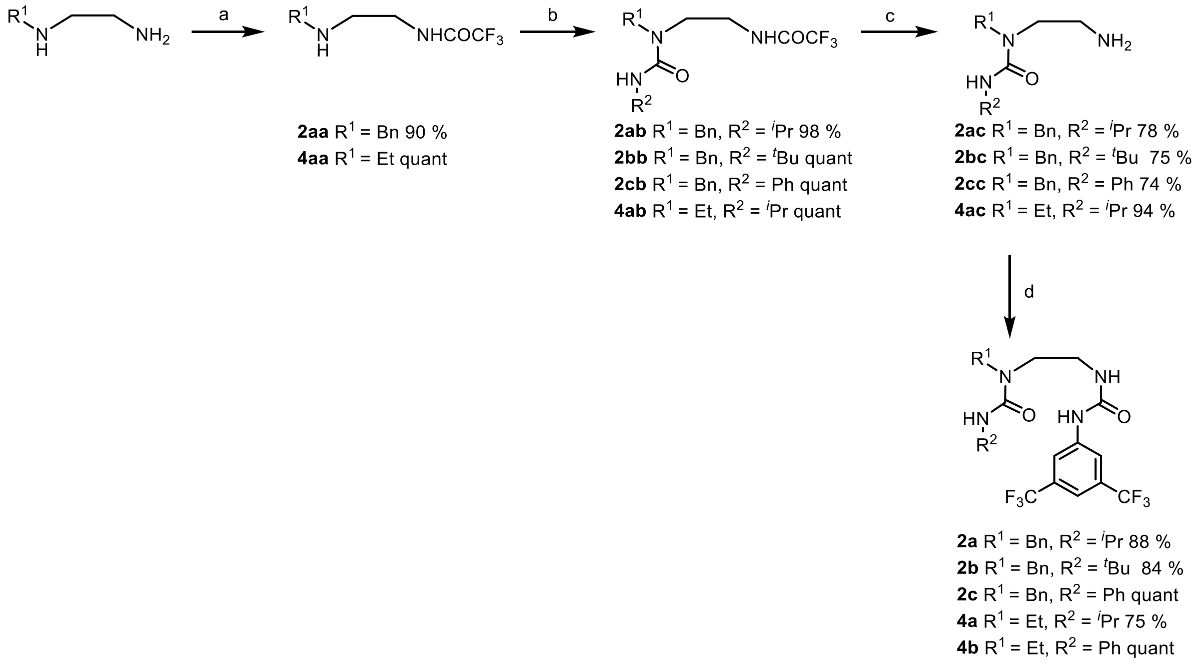

Scheme SI1. Synthesis of diureas **2a-c**, **4a-b**. Reagents and conditions: (a) CF_3CO_2Et (1 equiv.), MeOH, -78 °C to RT, 12h; (b) R^2NCO (1.2 equiv.), 1,2-DCE, 50 °C, 12 h; (c) NaOH aq. 0.2 M (1 equiv.), MeOH, 40 °C, 12 h.; (d) 3,5-$(CF_3)_2PhNCO$ (1.1 equiv.), CH_2Cl_2, RT, 16 h.

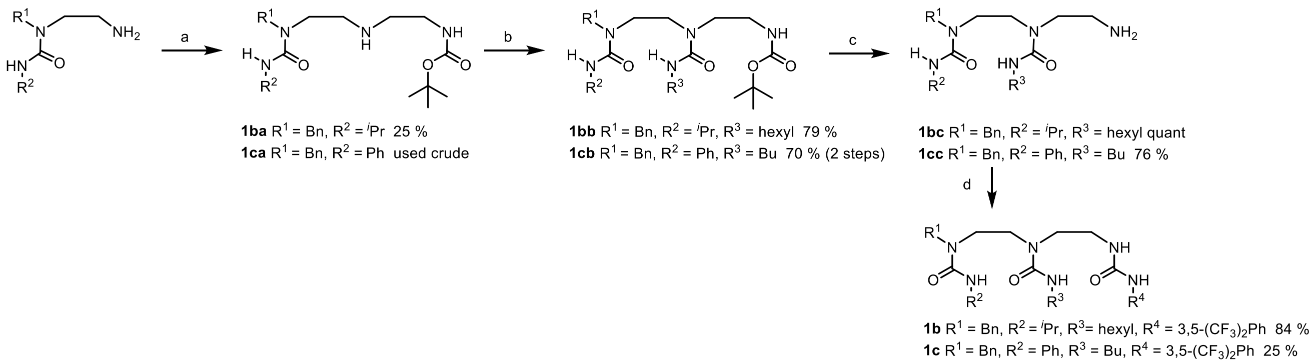

Scheme SI2. Synthesis of triureas **1b**, **1c**. Reagents and conditions: (a) *N*-Boc-2-aminoacetaldehyde (1.5 equiv.), MeOH, RT, 12 h. then $NaBH_4$ (3 equiv.), RT, 5 h.; (b) R^3NCO (1.5 equiv.), CH_2Cl_2, RT, 12 h.; (c) CF_3CO_2H (18 equiv.), CH_2Cl_2, RT, 12 h.; (d) R^4NCO (1.1 equiv.), CH_2Cl_2, RT, 16 h.

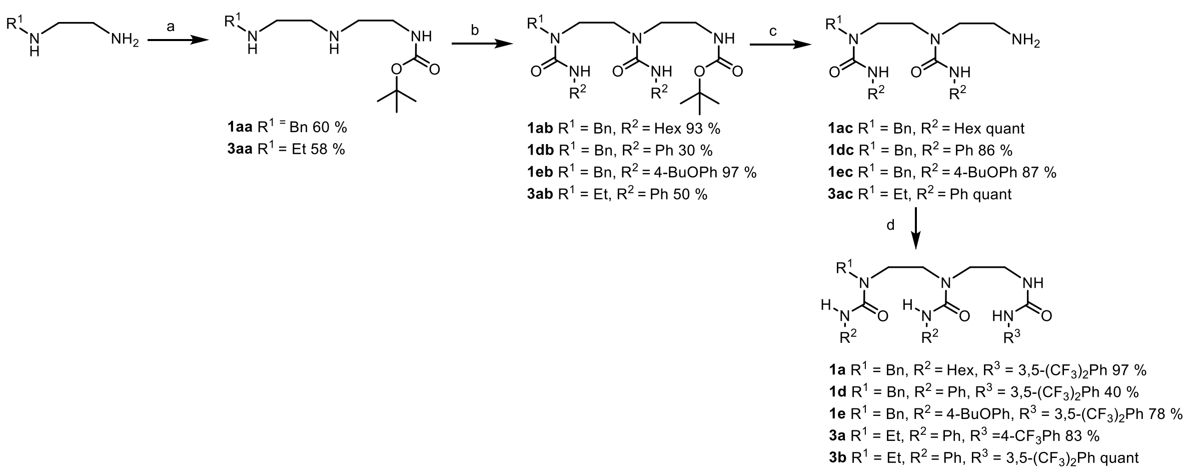

Scheme SI3. Synthesis of triureas **1a**, **1d**, **1e**, **3a-b**. Reagents and conditions: (a) *N*-Boc-2-aminoacetaldehyde (0.67 equiv.), MeOH, RT, 12 h. then $NaBH_4$ (1.35 equiv.), RT, 5 h.; (b) RNCO (3 equiv.), CH_2Cl_2, RT, 12 h.; (c) CF_3CO_2H (18 equiv.), CH_2Cl_2, RT, 12 h.; (d) R^3NCO (1.1 equiv.), CH_2Cl_2, RT, 16 h.

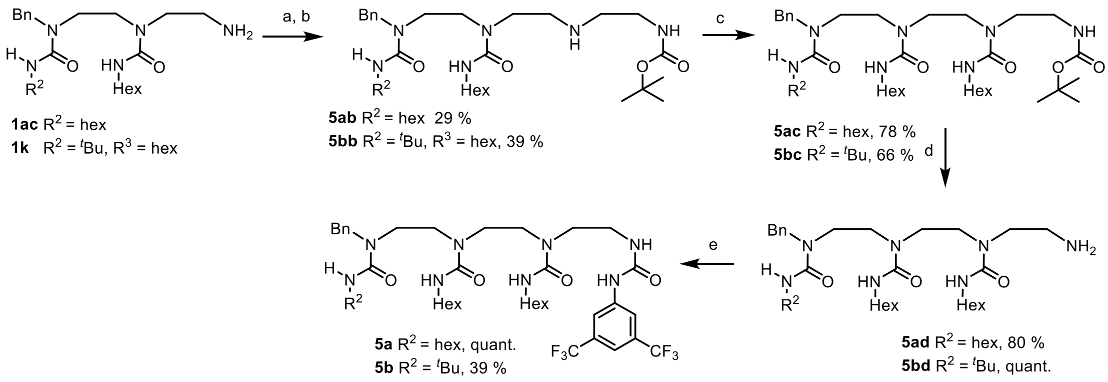

Scheme SI4. Synthesis of tetraureas **5a**, **5b**. Reagents and conditions: (a) *N*-Boc-2-aminoacetaldehyde (1.5 equiv.), MeOH, RT, 12 h.; (b) $NaBH_4$ (3 equiv.), RT, 5 h.; (c) HexylNCO (1.5 equiv.), CH_2Cl_2, RT, 12 h.; (d) CF_3CO_2H (18 equiv.), CH_2Cl_2, RT, 12 h.; (e) $(CF_3)_2$PhNCO (1.2 equiv.), CH_2Cl_2, RT, 4 h.

3. Procedures and characterisation of compounds

3.1. General experimental Procedures

General procedure A for installing a carbamoyl substituent on the *N*-trifluoroacetyl-*N'*-R¹ethylenediamine

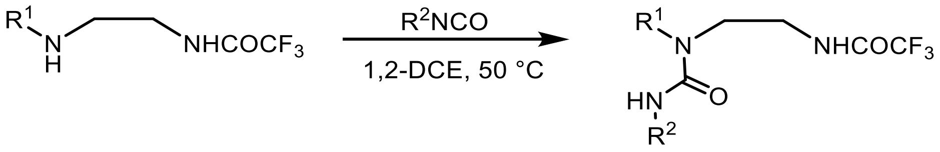

To a 0.1 M solution of *N*-trifluoroacetyl-*N'*-alkylethylenediamine in anhydrous 1,2-dichloroethane was added the isocyanate (1.2 equiv.), the solution was stirred at 50 °C for 12 hours. The mixture was washed at 20 °C with water (20 mL), the organic layer was dried over Na_2SO_4, filtered, concentrated under reduced pressure. Flash chromatography of the residue on silica gel (eluent CH_2Cl_2:MeOH 95:5) provided the *N*-trifluoroacetyl-*N'*-(alkylcarbamoyl)-*N'*-alkylethylenediamine.

General procedure B for the deprotection of trifluoroacetamide from *N*-trifluoroacetyl-*N'*-(alkylcarbamoyl)-*N'*-alkylethylenediamines

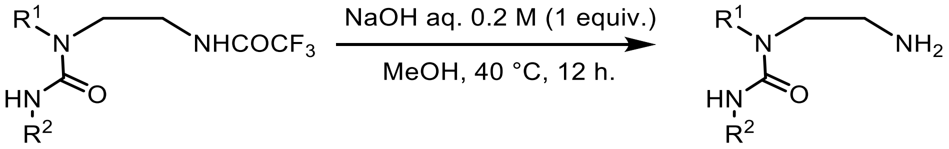

To a 0.07 M solution of *N*-trifluoroacetyl-*N*'-(alkyl/arylcarbamoyl)-*N*'-R^{l}ethylenediamine in MeOH was added a 0.2M aqueous solution of NaOH (1 equiv. per trifluoroacetamide) and the mixture was stirred for 12 hours at 40 °C. The mixture was concentrated under reduced pressure to remove MeOH, the aqueous phase was extracted with CH_2Cl_2. The organic layer was dried over Na_2SO_4, filtered and concentrated under reduced pressure. Products tend to degrade on silica, no chromatography purification is preferred for that step.

General procedure C for the reductive amination with *N*-Boc-2-aminoacetaldehyde

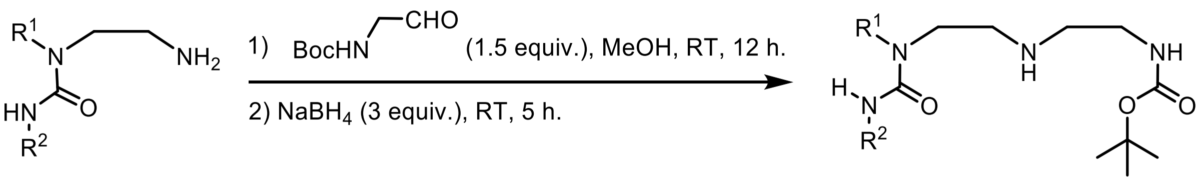

A 0.1 M solution of alkylamine in anhydrous MeOH and *N*-Boc-2-aminoacetaldehyde (1.5 equiv.) was stirred at 20 °C for 12 hours. Sodium borohydride $NaBH_4$ (3 equiv.) was then added portionwise (1 equiv. every 30 minutes) at 0 °C, the resulting mixture was stirred for 5 hours at 20 °C. Water (20 mL) was added, the mixture was stirred for 30 minutes at 20 °C, then was concentrated under reduced pressure to remove MeOH. Brine was added, the aqueous phase was extracted with $CHCl_3$: *i*PrOH 2:1 mixture. The organic layer was dried over Na_2SO_4, filtered and concentrated under reduced pressure. Flash chromatography of the residue on silica gel gave the (*tert*butoxycarbonyl)alkylamine product. Some products tend to degrade on silica, no chromatography purification is preferred for that step.

General procedure D for installing carbamoyl substituents

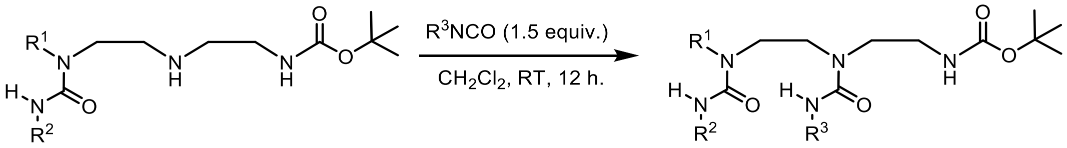

To a 0.1M solution of secondary amine in CH_2Cl_2 was added the desired alkyl/arylisocyanate (1.5 equiv. per NH group to substitute) and the mixture was stirred at 20 °C for 12 hours. Water was added, the resulting mixture was extracted using CH_2Cl_2. The organic layer was dried over Na_2SO_4, filtered and concentrated under reduced pressure. The crude product was purified by silica gel flash chromatography (CH_2Cl_2:MeOH 95:5) to afford the desired diurea compound.

General procedure E for the deprotection of *N*-Boc using trifluoroacetic acid

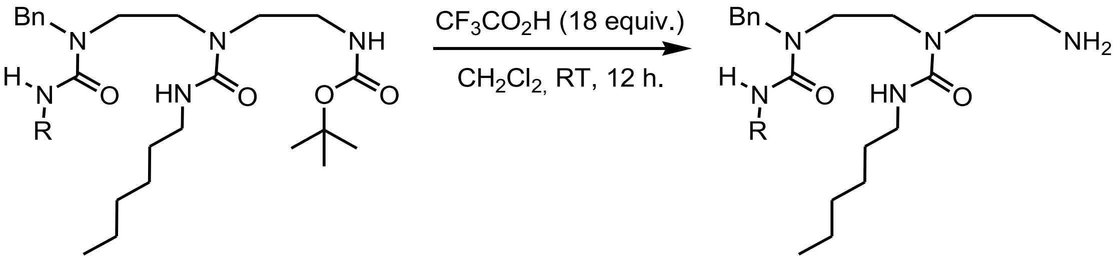

A 0.05 M solution of *N*-Boc protected amine in CH_2Cl_2 and trifluoroacetic acid (18 equiv.) was stirred for 12 hours at 20 °C. Water then CH_2Cl_2 were added, the organic layer was washed with aqueous $NaHCO_3$, dried over Na_2SO_4, filtered and concentrated under reduced pressure. The residue was purified by flash chromatography on silica gel (eluent: CH_2Cl_2:MeOH 95:5) to give the unprotected amine.

General procedure F for the preparation of *N*-alkyl-*N'*-(*tert*butoxycarbonyl)diethylenetriamine by reductive amination of *N*-alkylethylenediamines

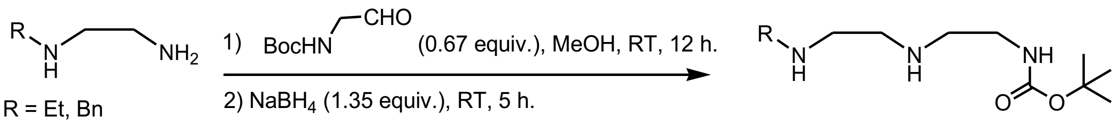

A solution of *N*-alkylethylenediamine (0.1 mM) and *N*-Boc-2-aminoacetaldehyde (0.67 equiv.) in anhydrous MeOH was stirred for 12 hours at 20 °C. Sodium borohydride $NaBH_4$ (1.35 equiv.) was added in 3 equal portions over 1 hour then the mixture was stirred for 5 hours. Water (2 mL) was added to the mixture and after stirring for 1 h, the mixture was concentrated under reduced pressure. The residue was purified by flash chromatography on silica gel using neat MeOH as eluent, to provide the desired compound.

General procedure G for installing the *N*-(3,5-*bis*(trifluoromethyl)phenylcarbamoyl) substituent

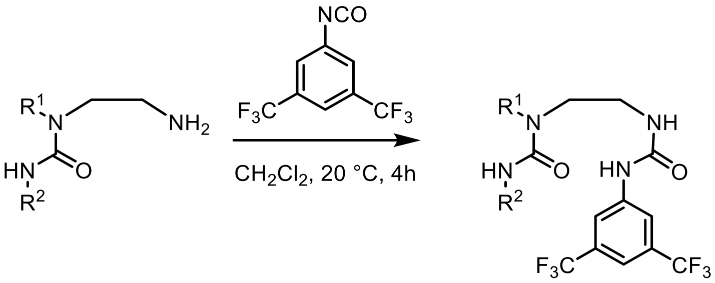

To a 0.05 M solution of primary amine in CH_2Cl_2 was added dropwise at 20 °C the 3,5-*bis*(trifluoromethyl)phenyl isocyanate (1.2 equiv. per primary amine), the mixture was stirred for 4 hours. Water was added, the aqueous layer was extracted using CH_2Cl_2. The organic layer was dried over Na_2SO_4, filtered and concentrated under reduced pressure. The residue was purified by flash chromatography on silica (eluent: CH_2Cl_2 : MeOH 95:5) to give the desired urea.

General procedure H

A solution of oligoethylenediamine (1.00 equiv, 1.6 M) in anhydrous MeOH was cooled to -78 °C then ethyl trifluoroacetate (1 equiv. per primary amine) was added dropwise over 30 minutes. The resultant solution was stirred for 12 hours while allowing the temperature to raise to 20 °C. The mixture was concentrated under reduced pressure then purified using silica gel flash chromatography (eluent: 100% MeOH). The resulting compound was dissolved in anhydrous CH_2Cl_2 to a concentration of 0.5M, then isocyanate (between 1.5 and 2 equiv. per secondary amine) was added to the mixture and the resultant solution was stirred between 16 hours and 2 days (for longer amines) at 40 °C. After concentration under reduced pressure, the residue was purified using silica gel flash chromatography (eluent: 97:3 CH_2Cl_2:MeOH).

3.2. Procedures and characterisation of compounds

2aa *N*-trifluoroacetyl-*N′*-benzylethylenediamine

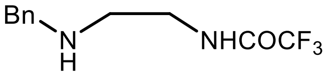

To a 0.4 M solution of *N*-benzylethylenediamine (2.5 g, 16.7 mmol) in anhydrous MeOH at -78 °C was added dropwise over 30 minutes ethyl trifluoroacetate (2 mL, 16.9 mmol, 1.01 equiv.). The mixture was stirred for 12 hours while allowing the temperature to slowly rise to 20 °C. The solvent was removed under reduced pressure, the residue was dissolved in CH_2Cl_2 (40 mL). The organic layer was washed with water, dried over Na_2SO_4, filtered and concentrated under reduced pressure. The residue was purified using flash chromatography on silica gel (CH_2Cl_2:MeOH 95:5), providing the title compound as a yellow oil (3.683g, 15.0 mmol, 90%). Analytical data agree with reported literature[1]. **^{1}H NMR** (400 MHz, $CDCl_3$) δ 7.41 – 7.17 (m, 5H, 5 x CH_{Ph}), 7.10 (bs, 1H, N*H*COCF$_3$), 3.77 (s, 2H, C*H*$_2$Ph), 3.38 (s, 1H, N*H*Bn), 3.36 (dd, *J* = 5.8, 6 Hz, 2H, C*H$_2$*), 2.80 (t, *J* = 6 Hz, 2H, C*H$_2$*). **^{13}C NMR** (101 MHz, $CDCl_3$) δ 157.3 (q, *J* = 36.8 Hz, *C*CF$_3$), 139.8 (*C*$_{Ph}$), 128.7 (2C, 2 x *C*H$_{Ph}$), 127.4 (2C, 2 x *C*H$_{Ph}$), 120.3 (*C*H$_{Ph}$), 116.0 (q, *J* = 287.7 Hz, *C*F$_3$), 53.3 (*C*H$_2$Ph), 46.8 (*C*H$_2$), 39.2 (*C*H$_2$).

2ab *N*-Trifluoroacetyl-*N*'-(isopropylcarbamoyl)-*N*'-benzylethylenediamine

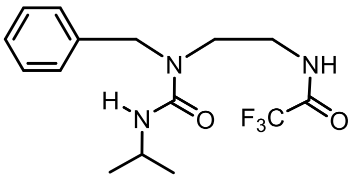

Following general procedure A with *iso*propyl isocyanate (0.476 mL, 4.8 mmol) and *N*-trifluoroacetyl-*N'*-benzylethylenediamine **2aa** (1 g, 4 mmol), the title compound was obtained as a white solid (1.3 g, 4.7 mmol, 98%). **^{1}H NMR** (400 MHz, $CDCl_3$) δ 8.72 (s, 1H, N*H*$COCF_3$), 7.38 (t, *J* = 7.2 Hz, 2H, 2 x CH_{Ph}), 7.31 (t, *J* = 7.2 Hz, 1H, CH_{Ph}), 7.19 (d, *J* = 7.4 Hz, 2H, 2 x CH_{Ph}), 4.40 (s, 2H, CH_2Ph), 4.30 (d, *J* = 7.3 Hz, 1H, N*Hi*Pr), 3.79 – 3.99 (m, *J* = 6.6 Hz, 1H, C*H*(CH_3)$_2$), 3.62 (dd, *J* = 6.8, 4.2 Hz, 2H, CH_2N), 3.43 (m, 2H, CH_2NH), 1.04 (d, *J* = 6.5 Hz, 6H, 2 x CH_3CH). **^{13}C NMR** (101 MHz, $CDCl_3$) δ 159.3 (C=O_{urea}), 158.0 (q, *J* = 37.1 Hz, *C*=OCF_3), 136.4 (C_{Ph}), 129.4 (2C, 2 x CH_{Ph}), 128.2 (CH_{Ph}), 126.3 (2C, 2 x CH_{Ph}), 116.04 (q, *J* = 287.3 Hz, CF_3), 51.6 (CH_2Ph), 46.3 (CH_2N), 43.1 (*C*H(CH_3)$_2$), 40.9 (CH_2NH), 23.1 (2CH_3, (CH_3)$_2$CH). **FTIR (neat)** vmax = 3246, 3066, 2974, 1709, 1626, 1531, 1211, 1178, 1159 cm^{-1}. **HR – MS** (ESI, positive ion mode) – *m/z* for $[C_{15}H_{20}F_3N_3O_2+H]^+$ 332.1580, observed 332.1584 ; *m/z* for $[C_{15}H_{20}F_3N_3O_2+Na]^+$ 354.1400, observed 354.1397. **MP** 93-94 °C.

2bb *N*-Trifluoroacetyl-*N*'-(tertbutylcarbamoyl)-*N*'-benzylethylenediamine

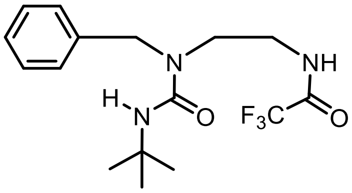

Following general procedure A with *tert*butyl isocyanate (475 mg, 4.8 mmol) and *N*-trifluoroacetyl-*N'*-benzylethylenediamine **2aa** (1 g, 4 mmol), the title compound was obtained as a white solid (1.39 g, 4 mmol, quantitative yield). **^{1}H NMR** (400 MHz, $CDCl_3$) δ 8.85 (s, 1H, N*H*Boc), 7.37 (t, *J* = 7.2 Hz, 2H, 2 x CH_{Ph}), 7.30 (t, *J* = 7.2 Hz, 1H, CH_{Ph}), 7.19 (d, *J* = 7.4 Hz, 2H, 2 x CH_{Ph}), 4.37 (s, 3H, CH_2Ph, N*H*), 3.63 (dd, *J* = 6.7, 4.2 Hz, 2H, CH_2), 3.42 (q, *J* = 4.6 Hz, 2H, CH_2NH), 1.21 (ms, 9H, 3 x CH_3C). **^{13}C NMR** (101 MHz, $CDCl_3$) δ 159.2 (C=O_{urea}), 158.0 (q, *J* = 37.0 Hz, *C*=OCF_3), 136.5 (C_{Ph}), 129.3 (2C, 2 x CH_{Ph}), 128.2 (CH_{Ph}), 126.3 (2C, 2 x CH_{Ph}), 116.0 (q, *J* = 287.5 Hz, CF_3), 51.9 (CH_2Ph), 51.3 (*C*(CH_3)$_3$), 46.1 (CH_2), 40.9 (CH_2), 29.1 (3 x CH_3). **FTIR (neat)** vmax = 3237, 2966, 1709, 1633, 1525, 1209, 1178, 1154 cm^{-1}. **HR – MS** (ESI, positive ion mode) – *m/z* for $[C_{16}H_{22}F_3N_3O_2+H]^+$ 346.1737, observed 346.1739 ; *m/z* for $[C_{16}H_{22}F_3N_3O_2+Na]^+$ 368.1556, observed 368.1552. **MP** 98-99 °C.

2cb *N*-trifluoroacetyl-*N*'-(phenylcarbamoyl)-*N*'-benzylethylenediamine

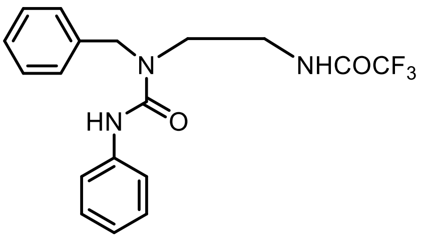

Following general procedure A with phenyl isocyanate (0.4 mL, 3.7 mmol) and *N*-trifluoroacetyl-*N′*-benzylethylenediamine **2aa** (750 mg, 3 mmol), the title compound was obtained as a white solid (1.095 g, 3 mmol, quantitative yield). **^{1}H NMR** (400 MHz, DMSO) δ 9.53 (s, 1H, N*H*COCF$_3$), 8.46 (s, 1H, N*H*Ph), 7.50 (d, *J* = 8.0 Hz, 2H, 2 x C*H*$_{Ph}$), 7.37 (t, *J* = 7.5 Hz, 2H, 2 x C*H*$_{Ph}$), 7.32 – 7.14 (m, 5H, 5 x C*H*$_{Bn}$), 6.97 (t, *J* = 7.3 Hz, 1H, C*H*$_{Ph}$), 4.65 (s, 2H, C*H*$_2$N), 3.47 (d, *J* = 6.0 Hz, 2H, C*H*$_2$N). **^{13}C NMR** (101 MHz, DMSO) δ156.6 (q, *J* = 36.2 Hz, *C*OCF$_3$), 155.4 (*C*O), 140.3 (*C*$_{Ph}$), 138.4 (*C*$_{Ph}$), 128.6 (2C, 2 x *C*H$_{Ph}$), 128.3 (2C, 2 x *C*H$_{Ph}$), 127.1 (3C,3 x *C*H$_{Ph}$), 122.0 (*C*H$_{Ph}$), 120.1 (2C, 2 x *C*H$_{Ph}$), 115.9 (q, J = 288.2 Hz, *C*F$_3$) 49.7 (*C*H$_2$Ph), 44.7 (*C*H$_2$), 37.9 (*C*H$_2$). **HR – MS** (ESI, positive ion mode) – *m/z* for $[C_{18}H_{18}N_3O_2F_3]^+$ 365.1346, found 365.1338.

4ab *N*-trifluoroacetyl-*N*'-(isopropylcarbamoyl)-*N*'-ethylethylenediamine

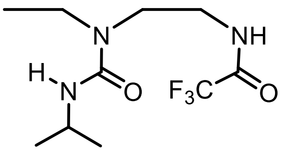

To a solution of *N*-ethylethylenediamine (0.2 mL, 1.9 mmol) in anhydrous MeOH (4 mL) at -78 °C was added dropwise ethyl trifluoroacetate (0.225 mL, 1.9 mmol, 1.01 equiv.). The mixture was stirred for 4 hours while allowing the temperature to raise to 20 °C. The solvent was removed under reduced pressure and the residue **4aa** was dissolved in anhydrous CH_2Cl_2 (2 mL). Following general procedure A with isopropylisocyanate (0.185 mL, 1.9 mmol, 1.2 equiv.), the residue was purified by flash chromatography (CH_2Cl_2:MeOH 95:5) on silica gel to give the title compound as a colourless oil (520 mg, 1.9 mmol, quantitative). **^{1}H NMR** (400 MHz, CDCl$_3$) δ 8.95 (s, 1H, N*H*COCF$_3$), 4.8 (d, *J* = 7.6 Hz, 1H, N*H*$_{iPr}$), 3.83 – 3.70 (m, 1H, C*H*(CH$_3$)$_2$), 3.35 – 3.22 (m, 4H, 2 x CH$_2$), 3.10 (q, *J* = 7.2, 7.1 Hz, 2H, CH$_2$), 1.12 – 0.79 (m, 9H, 3 x CH$_3$). **^{13}C NMR** (101 MHz, CDCl$_3$) δ 158.3 (C=O$_{iPrNH}$), 157.7 (q, *J =* 36.8 Hz, *C*=OCF$_3$), 115.8 (q, *J =* 287.2 Hz, *C*F$_3$), 44.5 (*C*H$_2$), 42.6 (*C*H$_{iPr}$), 41.9 (*C*H$_2$), 40.1 (*C*H$_2$), 22.7 (2C, 2 x *C*H$_3$), 13.1 (*C*H$_3$). **FTIR (neat)** vmax = 3242, 2072, 2975, 2934, 1712, 1625, 1529, 1217, 1182, 1156 cm^{-1}. **HR – MS** (ESI, positive ion mode) – *m/z* for $[C_{10}H_{18}F_3N_3O_2+H]^+$ 270.1424, observed 270.1418.

2ac *N*-(isopropylcarbamoyl)-*N*-benzylethylenediamine

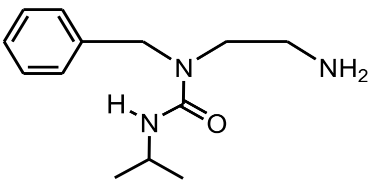

Following general procedure B with *N*-trifluoroacetyl-*N*'-(isopropylcarbamoyl)-*N*'-benzylethylenediamine **2ab** (1.2 g, 3.62 mmol), the title compound was obtained as an oil (667 mg, 2.82 mmol, 78%). **^{1}H NMR** (400 MHz, $CDCl_3$) δ 7.33 – 7.21 (m, 5H, 5 x CH_{Ar}), 5.72 (m, 1H, NH_{iPr}), 4.49 (s, 2H, CH_2Ph), 3.94 (dq, *J* = 6.5 Hz, 1H, $CH(CH_3)_2$), 3.27 (t, *J* = 5.7 Hz, 2H, CH_2NBn), 2.77 (t, *J* = 5.7 Hz, 2H, CH_2NH_2), 1.33 (s, 2H, NH_2), 1.10 (d, *J* = 6.5 Hz, 6H, 2 x CH_3CH). **^{13}C NMR** (101 MHz, $CDCl_3$) δ 159.0 (C=O), 138.7 (C_{Ar}), 128.6 (2C, 2 x CH_{Ar}), 127.4 (2C, 2 x CH_{Ar}), 127.2 (CH_{Ar}), 50.8 (CH_2Ph), 50.6 (CH_2NBn), 42.5 ($CH(CH_3)_2$), 40.9 (CH_2NH_2), 23.4 (2C, 2 x CH_{3iPr}). **FTIR (neat)** vmax = 3353, 2969, 2924, 2875, 1621, 1530, 1453, 1211 cm^{-1}. **HR – MS** (ESI, positive ion mode) – *m/z* for $[C_{13}H_{21}N_3O_1+H]^+$ 236.1757, observed 236.1755.

2bc *N*-(tertButylcarbamoyl)-*N*-benzylethylenediamine

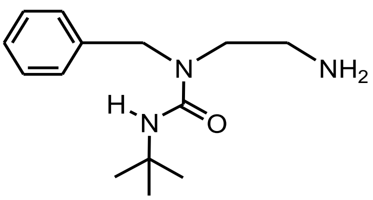

Following general procedure B with *N*-trifluoroacetyl-*N*'-(tertbutylcarbamoyl)-*N*'-benzylethylenediamine **2bb** (1.27 g, 3.68 mmol), the title product was obtained as an oil (700 mg, 2.76 mmol, 75%). **^{1}H NMR** (400 MHz, $CDCl_3$) δ 7.37 – 7.30 (m, 2H, 2 x CH_{Ar}), 7.30 – 7.23 (m, 3H, 3 x CH_{Ar}), 5.71 (s, 1H, N*H*), 4.47 (s, 2H, NH_2), 4.25 (s, 2H, CH_2Ph), 3.31 (t, *J* = 5.8 Hz, 2H, CH_2NBn), 2.79 (t, *J* = 5.8 Hz, 2H, CH_2NH_2), 1.32 (s, 9H, 3 x CH_3C). **^{13}C NMR** (101 MHz, $CDCl_3$) δ 158.8 (C=O), 138.8 (C_{Ar}), 128.6 (2C, 2 x CH_{Ar}), 127.3 (2C, 2 x CH_{Ar}), 127.2 (CH_{Ar}), 51.0 (CH_2Ph), 50.7 (CH_2NBn), 50.5 ($C(CH_3)_3$), 41.0 (CH_2NH_2), 29.4 (3C, 3 x CH_{3tBu}). **FTIR (neat)** vmax = 3288, 2963, 1635, 1528, 1452, 1359, 1211 cm^{-1}. **HR – MS** (ESI, positive ion mode) – *m/z* for $[C_{14}H_{23}N_3O_1+H]^+$ 250.1914, observed 250.1914.

2cc *N*-(phenylcarbamoyl)-*N*-benzylethylenediamine

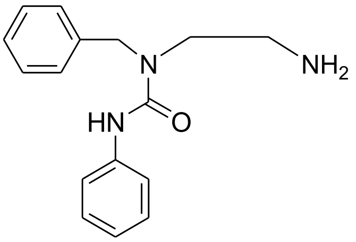

To a solution of *N*-trifluoroacetyl-*N*'-(phenylcarbamoyl)-*N*'-benzylethylenediamine **2cb** (738 mg, 3 mmol) in methanol (40 mL) was added an aqueous solution of 0.2M sodium hydroxide (15 mL, 3 mmol) and the mixture was stirred at 20 °C for 15 h. Methanol was removed under reduced pressure, the aqueous phase was extracted with dichloromethane. The organic phase was dried (Na_2SO_4), concentrated under reduced pressure. The residue was purified by chromatography on silica (eluent/ dichloromethane methanol 95:5 to 1:1) to yield the title compound (590 mg, 74%). **^{1}H NMR** (400 MHz,

$CDCl_3$) *δ* 9.52 (s, 1H, N*H*), 7.28 (d, *J* = 8.0 Hz, 2H, 2 x CH_{Ph}), 7.24 – 7.07 (m, 7H, 7 x CH_{Ph}), 6.85 (t, *J* = 7.4 Hz, 1H, CH_{Ph}), 4.40 (s, 2H, CH_2Ph), 3.22 (t, *J* = 5.1 Hz, 2H, CH_2), 2.89 (s, 3H, NH_3^+), 2.65 (t, *J* = 5.1 Hz, 2H, CH_2). **^{13}C NMR** (101 MHz, $CDCl_3$) *δ* 157.7 (*C*=O), 140.2 (C_{Ph}), 138.1 (C_{Bn}), 128.7 (2C, 2 x CH_{Ph}), 128.6 (2C, 2 x CH_{Bn}), 127.6 (2C, 2 x (CH_{Bn}), 127.3 ($CH_{Bn\ para}$), 122.2 ($CH_{Ph\ para}$), 119.6 (2C, 2 x $CH_{Ph\ ortho}$), 50.7 (CH_2Ph), 49.7 (CH_2), 40.4 (CH_2). **HR – MS** (ESI, negative ion mode) – *m/z* for $[C_{16}H_{18}N_3O]^+$ 268.1455, found 268.1443 ; **HR – MS** (ESI, positive ion mode) – *m/z* for $[C_{16}H_{20}N_3O]^+$ 270.1601, found 270.1585.

4ac *N*-(isopropylcarbamoyl)-*N*-ethylethylenediamine

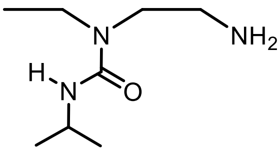

Following general procedure B, *N*-trifluoroacetyl-*N*'-(isopropylcarbamoyl)-*N*'-ethylethylenediamine **4ab** (493 mg, 1.8 mmol) afforded the title product as an oil (290 mg, 1.67 mmol, 94%). **^{1}H NMR** (400 MHz, $CDCl_3$) δ 5.43 (d, *J* = 6.5 Hz, 1H, N*H*), 3.93 – 3.74 (m, *J* = 6.6 Hz, 1H CH_{iPr}), 3.29 – 3.09 (m, 4H, 2 x CH_2), 2.78 (t, *J* = 5.8 Hz, 2H, CH_2N), 1.33 (d, *J* = 7.1 Hz, 2H, NH_2), 1.13 – 0.93 (m, 9H, 3 x CH_3). **^{13}C NMR** (101 MHz, $CDCl_3$) δ 158.4 (*C*=O), 50.0 (CH_2N), 42.3 (CH_2), 41.9 (CH_2), 41.3 (CH_2), 23.5 (2C, 2x CH_{3iPr}), 13.5 (CH_{3Et}). **FTIR (neat)** vmax = 3332, 2970, 2930, 2874, 1518, 1534, 1490, 1219 cm^{-1}. **HR – MS** (ESI, positive ion mode) – *m/z* for $[C_8H_{19}N_3O+H]^+$ 174.1601, observed 174.1595.

2a *N*-(3,5-bis(trifluoromethyl)phenylcarbamoyl)-*N*'-(isopropylcarbamoyl)-*N*'-benzylethylenediamine

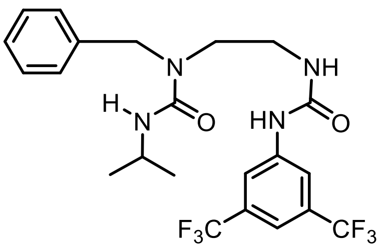

To a solution of *N*-(isopropylcarbamoyl)-*N*-benzylethylenediamine **2ac** (44 mg, 0.17 mmol) in CH_2Cl_2 (1 mL) was added dropwise at 20 °C 3,5-bis(trifluoromethyl)phenyl isocyanate (0.032 mL, 0.18 mmol), the mixture was stirred for 12 hours. The mixture was concentrated under reduced pressure, the residue was purified by flash chromatography (eluent: CH_2Cl_2:MeOH 95:5) on silica gel to afford *N*-(3,5-bis(trifluoromethyl)phenylcarbamoyl)-*N*'-(isopropylcarbamoyl)-*N*'-benzylethylenediamine **2a** (77 mg, 0.136 mmol, 88%) as a white solid. **^{1}H NMR** (400 MHz, $CDCl_3$) δ 8.90 (s, 1H, NH_{Ar}), 7.78 (s, 2H, 2 x CH_{Ar}), 7.33 (d, *J* = 2.0 Hz, 1H, CH_{Ar}), 7.22 – 7.13 (m, 2H, 2 x CH_{Ar}), 7.14 – 7.06 (m, 1H, CH_{Ar}), 7.02 (dd, *J* = 7.0, 1.7 Hz, 2H, 2 x CH_{Ar}), 6.67 – 6.14 (m, 2H, 2 x N*H*), 4.34 (s, 2H, CH_2Ph), 3.93 – 3.68 (q, *J* = 6.6 Hz, 1H, C*H*($CH_3)_2$), 3.29 – 3.02 (m, 2H, CH_2), 3.00 – 2.72 (d, *J* = 8.8 Hz, 2H, CH_2NH), 1.19 – 0.88 (d, *J* = 6.5 Hz, 6H, 2 x CH_3). **^{13}C NMR** (101 MHz, $CDCl_3$) δ 158.7 (*C*=O_{NHiPr}), 156.8 (*C*=O_{Ar}), 141.5 (C_{Ar}), 137.9 (C_{Ph}), 132.1 (q, *J* = 33.1 Hz, 2C, 2 x *C*CF_3), 129.0 (2C, 2 x CH_{Ar}), 127.7 (CH_{Ar}), 126.5 (2C, 2 x CH_{Ar}), 123.5 (q, *J* = 271.5 Hz, 2C, 2 x CF_3), 118.0 (2C, 2 x CH_{Ar}), 115.1 (CH_{Ar}), 51.2 (CH_2Ph), 47.0 (CH_2N), 43.5 (CH_{iPr}), 38.9 (CH_2NH), 22.9 (2C, 2 x

CH_3). **FTIR (neat)** vmax = 3299, 2975, 1692, 1609, 1567, 1473, 1386, 1276, 1175, 1128 cm^{-1}. **HR – MS** (ESI, positive ion mode) – *m/z* for $[C_{22}H_{24}F_6N_4O_2+H]^+$ 491.1876, observed 491.1863. **MP** 162 – 164 °C.

2b *N*-(3,5-bis(trifluoromethyl)phenylcarbamoyl)-*N*'-(tertbutylcarbamoyl)-*N*'-benzylethylenediamine

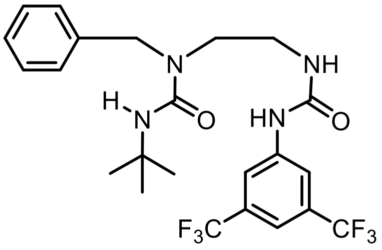

To a solution of *N*-(tertbutylcarbamoyl)-*N*-benzylethylenediamine **2bc** (50 mg, 0.2 mmol) in CH_2Cl_2 (1 mL) was added dropwise at 20 °C 3,5-bis(trifluoromethyl)phenyl isocyanate (0.038 mL, 0.22 mmol), the mixture was stirred for 12 hours. The mixture was concentrated under reduced pressure, the residue was purified by flash chromatography on silica gel (eluent: CH_2Cl_2:MeOH 95:5) to afford *N*-(3,5-bis(trifluoromethyl)phenylcarbamoyl)-*N*'-(tertbutylcarbamoyl)-*N*'-benzylethylenediamine **2b** (84 mg, 0.168 mmol, 84%) as a white solid. **^{1}H NMR** (400 MHz, $CDCl_3$) δ 8.79 (s, 1H, NH_{Ar}), 7.94 (s, 2H, 2 x CH_{Ar}), 7.42 (s, 1H, CH_{Ar}), 7.34 – 7.27 (m, 2H, 2 x CH_{Ar}), 7.27 – 7.19 (t, *J* = 7.4 Hz, 1H, CH_{Ar}), 7.19 – 7.10 (m, 2H, 2 x CH_{Ar}), 6.30 (bm, 1H, N*H*CH_2), 5.29 (s, 1H, N*H*), 4.43 (s, 2H, CH_2Ph), 3.38 (m, *J* = 7.1 Hz, 2H, CH_2), 3.21 (m, 2H, CH_2NH), 1.29 (s, 9H (CH_3)$_3$C). **^{13}C NMR** (101 MHz, $CDCl_3$) δ 158.6 (*C*=ONH*t*Bu), 156.4 (*C*=O), 141.5 (C_{Ar}), 137.4 (C_{Ph}), 132.0 (q, 2C, *J* = 33.1Hz, 2 x *C*CF_3), 129.1 (2C, 2 x CH_{Ph}), 127.6 (CH_{Ph}), 126.5 (2C, 2 x CH_{Ph}), 123.5 (q, 2C, *J* = 272.6 Hz, 2 x CF_3), 118.1 (2C, 2 x CH_{Ar}), 115.1 (CH_{Ar}), 51.4 (CH_2Ph), 47.3 (CH_2N), 39.0 (CH_2NH), 29.3 (3C, (CH_3)$_3$C). **FTIR (neat)** vmax = 3327, 2982, 1693, 1611, 1556, 1473, 1385, 1274, 1173, 1125 cm^{-1}. **HR – MS** (ESI, positive ion mode) – *m/z* for $[C_{23}H_{26}F_6N_4O_2+H]^+$ 505.2033, observed 505.2021. **MP** 149 °C.

2c *N*-(phenylcarbamoyl)-*N*-benzyl-*N*'-(3,5-bis(trifluoromethyl)phenylcarbamoyl)ethylenediamine

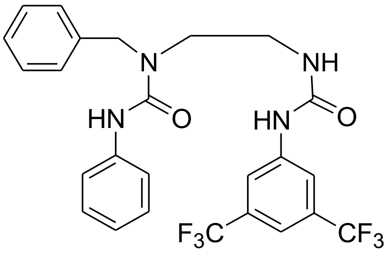

To a solution of *N*-(phenylcarbamoyl)-*N*-benzylethylenediamine **2cc** (62 mg, 0.23 mmol) in dichloromethane (3 mL) was added 3,5-bis(trifluoromethyl)phenylisocyanate (0.04 mL, 0.23 mmol) and the mixture was stirred at 20 °C for 12 h. After concentration under reduced pressure, the residue was purified by chromatography on silica (eluent dichloromethane methanol 95:5) to yield the title product as a white solid (120 mg, quantitative). **^{1}H NMR** (400 MHz, DMSO) *δ* 9.50 (s, 1H, N*H*), 8.70 (s, 1H, N*H*), 8.16 – 8.07 (s, 2H, 2 x C$H_{(CF3)2Ph\ ortho}$, 7.60 – 7.53 (m, 3H, C$H_{(CF3)2Ph\ para}$, 2 x C$H_{Ph\ ortho}$), 7.39 – 7.32 (m, 2H, 2 x C*H* $_{Bn}$), 7.32 – 7.24 (m, 3H, 3 x C*H* $_{Bn}$), 7.23 – 7.17 (m, 2H, 2 x C$H_{Ph\ meta}$), 6.97 – 6.90 (td, *J* = 7.3, Hz, 1H, C$H_{Ph\ para}$), 6.70 (t, *J* = 5.5 Hz, 1H, N*H*CH_2), 4.63 (s, 2H, CH_2Ph), 3.43 (t, *J* = 6.9 Hz, 2H, CH_2), 3.31 – 3.21 (td, *J* = 5.5, 6.9 Hz, 2H, CH_2NH). **^{13}C NMR** (101 MHz, DMSO) *δ* 155.6

(C=O), 155.4 (C=O), 142.4 ($C_{(CF3)2Ph}$), 140.6 (C_{Ph}), 138.8 (C_{Bn}), 130.6 (q, J = 32.5 Hz, 2 x CCF$_3$), 128.5 (2C, 2 x $CH_{Ph\ meta}$), 128.2 (2C, 2 x CH_{Bn}), 127.3 (2C, 2 x CH_{Bn}), 127.0 (1C, $CH_{Bn\ para}$), 123.3 (q, J = 272.6 Hz, 2C, 2 x CF_3), 121.8 (1C, $CH_{Ph\ para}$), 119.6 (2C, 2 x $CH_{Ph\ ortho}$), 117.50 (d, J = 4.0 Hz, 2C, 2 x $CH_{(CF3)2Ph\ ortho}$), 113.6 (m, 1C, $CH_{(CF3)2Ph\ para}$), 49.7 (1C, CH$_2$Ph), 45.8 (1C, CH_2), 38.5 (1C, CH$_2$NH). **HR – MS** (ESI, positive ion mode) – *m/z* for $[C_{25}H_{23}F_6N_4O_2]^+$ 525.1720, found 525.1708. **MP** 206-208 °C.

4a *N*-(3,5-bis(trifluoromethyl)phenylcarbamoyl)-*N'*-(isopropylcarbamoyl)-*N'*-ethylethylenediamine

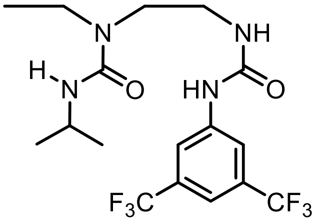

To a solution of *N*-(isopropylcarbamoyl)-*N*-ethylethylenediamine **4ca** (24 mg, 0.14 mmol) in dichloromethane (3 mL) was added 3,5-bis(trifluoromethyl)phenylisocyanate (0.027 mL, 0.15 mmol) and the mixture was stirred at 20 °C for 12 h. After concentration under reduced pressure, the residue was purified by chromatography on silica (eluent dichloromethane methanol 95:5) to yield the title product as a white solid (45 mg, 75%). **^{1}H NMR** (400 MHz, CDCl$_3$) δ 8.92 (s, 1H, NH), 7.86 (s, 2H, 2 x CH_{Ph}), 7.34 (s, 1H, CH_{Ph}), 6.53 (s, 1H, NH), 5.12 (s, 1H, NH^iPr), 3.92 – 3.75 (m, J = 6.6 Hz, 1H, CH(CH$_3$)$_2$), 3.31 (tt, J = 10.8, 4.4 Hz, 4H, 2 x CH_2N), 3.21 (q, J = 7.1 Hz, 2H, NCH_2CH$_3$), 1.12 – 1.05 (m, 9H, 3 x CH_3). **^{13}C NMR** (101 MHz, CDCl$_3$) δ 158.1 (C=O^{i}Pr), 156.5 (C=O), 141.6 (C_{Ph}NH), 132.1 (q, J = 33.1 Hz, 2C, 2 x $\underline{C}$CF$_3$), 123.5 (q, J = 272.6 Hz, 2 x CF_3), 118.0 (2C, 2 x CH_{Ph}), 115.0 (hept, J = 3.7 Hz, CH_{Ph}), 46.4 (CH_2), 43.2 (CH(CH$_3$)$_2$), 42.3 (CH_2), 39.2 (CH_2), 23.2 (2C, (CH$_3$)$_2$CH), 13.7 (CH$_3$CH$_2$). **HR – MS** (ESI, positive ion mode) – *m/z* for $[C_{17}H_{23}N_4O_2F_6]^+$ 4429.1720, found 429.1708. **MP** 176-178 °C.

4b *N*-(3,5-bis(trifluoromethyl)phenylcarbamoyl)-*N'*-(phenylcarbamoyl)-*N'*-ethylethylenediamine

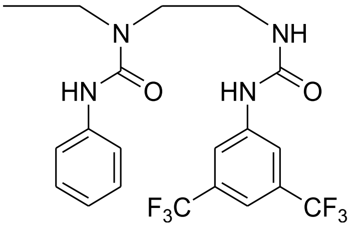

To a solution of *N*-(3,5-bis(trifluoromethyl)phenylcarbamoylethylethylenediamine **4ac** (33 mg, 0.15 mmol) in dichloromethane (1.5 mL) was added phenyl isocyanate (0.015 mL, 0.15 mmol) and the mixture was stirred at 20 °C for 12 h, after which the mixture was concentrated under reduced pressure. The residue was washed with dichloromethane to afford the title product as a white solid (68 mg, quantitative). **^{1}H NMR** (400 MHz, DMSO) δ 9.50 (s, 1H, (CF$_3$)$_2$PhNH), 8.43 (s, 1H, NHPh), 8.12 (d, J = 1.6 Hz, 2H, 2 x C$H_{(CF3)2Ph}$), 7.55 (s, 1H, C$H_{(CF3)2Ph}$), 7.47 (d, J = 7.7 Hz, 2H, 2 x C$H_{Ph\ ortho}$), 7.27 – 7.12 (dd, J = 7.7, 7.2 Hz, 2H, C$H_{Ph\ meta}$), 6.98 – 6.86 (t, J = 7.2 Hz, 1H, C$H_{Ph\ para}$), 6.68 (t, J = 5.5 Hz, 1H, NHCH$_2$), 3.44 – 3.38 (m, 4H, 2 x CH_2), 3.29 (q, J = 6.3 Hz, 2H, CH_2), 1.10 (t, J =

7.0 Hz, 3H, CH_3). **^{13}C NMR** (101 MHz, DMSO) δ 155.5 (*C*=ONH(CF_3)$_2$Ph), 154.9 (*C*=ONHPh), 142.4 ($C_{(CF3)2Ph}$), 140.6 (C_{Ph}), 130.63 (q, *J* = 32.5 Hz, 2C, 2 x *C*(CF_3)), 128.2 (2C, 2 x $CH_{Ph\ meta}$), 123.4 (q, *J* = 272.6 Hz, 2C, 2 x CF_3), 121.7 (CH_{Phpara}), 119.7 (2C, 2 x $CH_{Ph\ ortho}$), 117.4 (2C, 2 x $CH_{(CF3)2Ph}$), 113.7 (1C, $CH_{(CF3)2Ph}$), 45.7 (CH_2), 41.6 (CH_2CH_3), 38.8 (CH_2), 13.8 (CH_3). **HR – MS** (ESI, positive ion mode) – *m/z* for $[C_{20}H_{21}N_6O_2N_4]^+$ 463.1563, found 463.1551. **MP** 193-195 °C.

1ba *N*-(isopropylcarbamoyl)-*N*-benzyl-*N*'-(*tert*butoxycarbonyl)diethylenetriamine

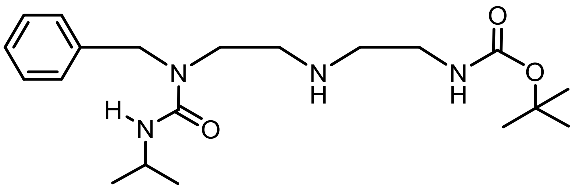

Following general procedure C, *N*-(isopropylcarbamoyl)-*N*-benzylethylenediamine **2ac** (667 mg, 2.84 mmol) reacted with *N*-Boc-2-aminoacetaldehyde (677 mg, 4.26 mmol) to afford the title compound as colourless oil (260 mg, 0.71 mmol, 25% after chromatography). **^{1}H NMR** (400 MHz, $CDCl_3$) δ 7.29 – 7.22 (m, 2H, 3 x CH_{Ar}), 7.22 – 7.15 (m, 3H, 3 x CH_{Ar}), 5.40 (m, 1H, N*H*), 4.82 (m, 1H, N*H*), 4.41 (s, 2H, CH_2Ph), 3.97 – 3.80 (m, 1H, C*H*(CH_3)$_2$), 3.26 (t, *J* = 5.7 Hz, 2H, CH_2NBn), 3.11 (m, 2H, CH_2NHBoc), 2.67 – 2.58 (m, 4H, 2 x CH_2NH), 1.60 (s, 1H, N*H*), 1.37 (s, 9H, 3 x CH_3C), 1.04 (d, *J* = 6.5 Hz, 6H, 2 x CH_3CH). **^{13}C NMR** (101 MHz, $CDCl_3$) δ 158.8 (C=O_{urea}), 156.2 (C=O_{Boc}), 138.6 (C_{Ar}), 128.8 (2C, 2 x CH_{Ar}), 127.5 (2C, 2 x CH_{Ar}), 127.4 (CH_{Ar}), 79.4 (*C*(CH_3)$_3$), 51.0 (CH_2Ph), 49.3 (CH_2NH), 48.4 (CH_2NH), 48.2 (CH_2NBn), 42.6 (*C*H(CH_3)$_2$), 40.3 (CH_2NHBoc), 28.5 (3C, 3 x CH_3C), 23.5 (2C, 2 x CH_{3iPr}). **FTIR (neat)** vmax = 3304, 2971, 2930, 1698, 1627, 1529, 1364, 1248, 1171 cm^{-1}. **HR – MS** (ESI, positive ion mode) – *m/z* for $[C_{20}H_{34}N_4O_3+H]^+$ 379.2704, observed 379.2699.

1bb *N*-(isopropylcarbamoyl)-*N*-benzyl-*N*'-(tertbutoxycarbonyl)-*N*''-(hexylcarbamoyl)diethylenetriamine

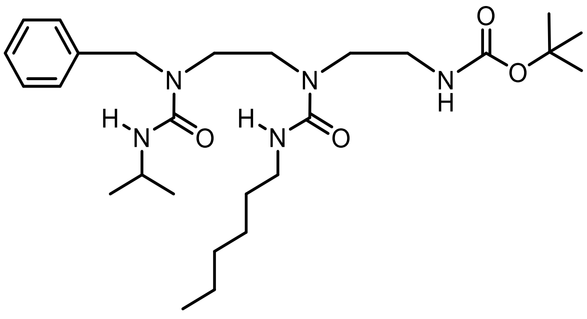

Following general procedure D with *N*-(isopropylcarbamoyl)-*N*-benzyl-*N*'-(tertbutoxycarbonyl)diethylenetriamine **1ba** (472 mg, 1.25 mmol) and hexyl isocyanate (1.5 equiv.) afforded the title compound (495 mg, 0.98 mmol, 79%) as a colourless oil. **^{1}H NMR** (400 MHz, $CDCl_3$) δ 7.31 (dd, *J* = 7.9, 6.4 Hz, 2H, 2 x CH_{Bn}), 7.25 (d, *J* = 7.3 Hz, 1H, CH_{Pn}), 7.22 – 7.17 (m, 2H, 2 x CH_{Bn}), 6.20 (s, 1H, N*H*Boc), 5.44 – 5.06 (m, 2H, 2 x N*H*), 4.44 (s, 2H, CH_2Ph), 3.99 – 3.83 (m, 1H, C*H*(CH_3)$_2$), 3.38 – 3.26 (m, 2H, CH_2NBn), 3.26 – 3.15 (m, 6H, 3 x CH_2N), 3.08 (q, *J* = 6.4 Hz, 2H, CH_2NH), 1.52 (t, *J* = 7.3 Hz, 2H, CH_2), 1.40 (s, 9H, (CH_3)$_3$CO), 1.37 – 1.23 (m, 6H, 3 x CH_2), 1.09 (d, *J* = 6.5 Hz, 6H, (CH_3)$_2$CH), 0.96 – 0.77 (m, 3H, C$H_{3\ hexyl}$). **^{13}C NMR** (101 MHz, $CDCl_{3,}$) δ 158.7 (C=O), 158.0 (C=O), 156.7 (C=O), 138.1 (C_{Ar}), 128.8 (2C, 2 x CH_{Ar}), 127.5 (CH_{Ar}), 127.0 (2C, 2 x CH_{Ar}),

79.5 ($C(CH_3)_3O$), 51.4 (CH_2Ph), 47.5 (CH_2N), 46.8 (2C, 2 x CH_2N), 46.6 (CH_2N), 42.7 (CH_2), 41.0 (CH_2), 39.9 (CH_2NH), 31.6 (CH_2), 29.9 (CH_2), 28.4 ($(CH_3)_3CO$), 26.7 (CH_2), 23.2 ($(CH_3)_2CH$), 22.6 (CH_2), 14.1 (CH_3). **FTIR (neat)** vmax = 3299, 2967, 2929, 2871, 1689, 1626, 1536, 1355, 1248, 1173 cm^{-1}. **HR – MS** (ESI, positive ion mode) – *m/z* for $[C_{27}H_{47}N_5O_4+H]^+$ 506.3701, observed 506.3687.

1cb *N*-(phenylcarbamoyl)-*N*-benzyl-*N*'-(tertbutoxycarbonyl)-*N*''-(butylcarbamoyl)diethylenetriamine

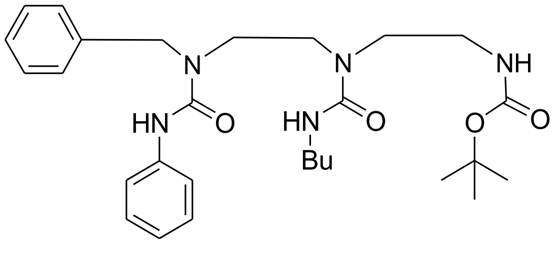

Following general procedure C, *N*-(isopropylcarbamoyl)-*N*-benzylethylenediamine **2cb** (667 mg, 2.84 mmol) reacted with *N*-Boc-2-aminoacetaldehyde (677 mg, 4.26 mmol) to afford **1ca**. Following general procedure D with the amine **1ca** (107 mg, 0.26 mmol) in CH_2Cl_2 (1 mL) and butyl isocyanate (0.03 mL, 0.26 mmol), the product was obtained after purification by silica gel flash chromatography (CH_2Cl_2:MeOH 9:1) (93 mg, 0.18 mmol, 70%). **^{1}H NMR** (400 MHz, $CDCl_3$) δ 7.54 (d, *J* = 7.9 Hz, 2H, 2 x $CH_{Ph\ ortho}$), 7.36 – 7.09 (m, 7H, N*H*Ph, 2 x $CH_{Ph\ meta}$, 4 x CH_{Bn}), 6.95 – 6.81 (m, 2H, 2 x $CH_{Ph\ para}$), 6.04 (s, 1H, N*H*Bu), 5.13 (t, *J* = 5.6 Hz, 1H, N*H*Boc), 4.49 (s, 2H, CH_2Ph), 3.32 (t, *J* = 7.3 Hz, 2H, CH_2N), 3.20 – 3.10 (m, 4H, CH_2NH_{Bu}, CH_2N), 3.06 (t, *J* = 7.2 Hz, 2H, CH_2N), 2.95 (td, J = 6.8, 5.6 Hz, 2H, CH_2NHBoc), 1.42 – 1.38 (m, 2H, CH_2), 1.33 (s, 9H, $(CH_3)_3C$), 1.30 -1.26 (m, 2H, CH_2), 0.83 (t, 3H, *J* = 7.3 Hz, CH_{3Bu}). **^{13}C NMR** (101 MHz, $CDCl_3$) δ 158.8 (*C*=ONHBu), 156.9 (*C*=ONHBoc), 156.2 (*C*=ONHPh), 140.2 (C_{Ph}), 138.2 (C_{Bn}), 128.8 (2C, CH_{meta}), 128.6(3C, $CH_{Ph\ para}$, 2 x CH_{Bn}), 127.5 (2C, CH_{Bn}), 122.4 (1C, $CH_{Ph\ para}$), 119.7 (2C, 2 x $CH_{Ph\ ortho}$), 79.9 (1C, $C(CH_3)_3$), 51.1 (CH_2Ph), 47.8 (CH_2N), 47.6 (CH_2N), 46.0 (CH_2N), 40.7 (CH_2N), 39.8 (CH_2NHBoc), 31.9 (1C, CH_2), 28.3 (3C, $(CH_3)_3C$), 20.1 (1C, CH_2), 13.9 (1C, CH_3). **HR – MS** (ESI, positive ion mode) – *m/z* for $[C_{28}H_{41}N_5O_4Na]^+$ 534.3051, observed 534.3031.

1bc *N*-(isopropylcarbamoyl)-*N*-benzyl-*N*''-(hexylcarbamoyl)diethylenetriamine

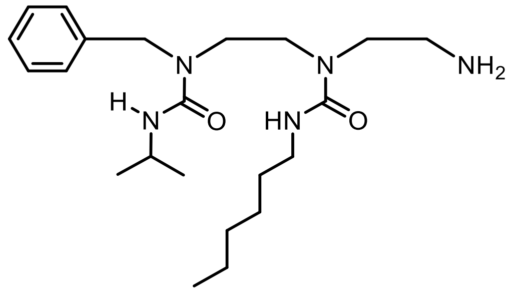

Following general procedure E with *N*-(isopropylcarbamoyl)-*N*-benzyl-*N*'-(*tert*butoxycarbonyl)-*N*''-(hexylcarbamoyl)diethylenetriamine **1bb** (195 mg, 0.38 mmol), the title compound was obtained as a colourless oil (154 mg, 0.38 mmol, quantitative). **^{1}H NMR** (400 MHz, $CDCl_3$) δ 7.22 (dd, *J* = 8.1 Hz, 6.4 Hz, 2H, 2 x CH_{Ar}), 7.18 – 7.06 (m, 3H, 3 x CH_{Ar}), 6.52 (t, *J* = 5.3 Hz, 1H, NH_{Hexyl}), 5.33 (m, 1H, N*Hi*Pr), 4.37 (s, 2H, CH_2Ph), 3.95 – 3.78 (m, 1H, $CH(CH_3)_2$), 3.23 (m, 2H, CH_2NBn), 3.16 – 2.95 (m, 6H, 3 x CH_2), 2.67 (m, 2 H, CH_2NH_2), 2.30 (m, 2H, NH_2), 1.39 (m, 2H, CH_2), 1.27 – 1.12 (m, 6H, 3 x CH_2), 1.04 (d, *J* = 6.5 Hz, 6H, $(CH_3)CH$), 0.84 – 0.71 (m, 3H, CH_{3hexyl}). **^{13}C NMR** (101 MHz, $CDCl_3$) δ159.8 (C=O $_{hexyl\ urea}$), 157.9

(C=O $_{iPr\ urea}$), 138.4 (C_{Ar}), 128.7 (2C, 2 x CH_{Ar}), 127.3 (CH_{Ar}), 127.2 (2C, 2 x CH_{Ar}), 51.4 (CH_2N), 51.1 (CH_2Ph), 46.6 (CH_2), 46.0 (CH_2NBn), 42.7 ($CH(CH_3)_2$), 41.1 (CH_2NH_2), 40.8 (CH_2N), 31.5 (CH_2), 30.0 (CH_2), 26.7 (CH_2), 23.1 (2C, 2 x CH_{3iPr}), 22.6 (CH_2), 14.0 ($CH_{3\ Hexyl}$). **FTIR (neat)** vmax = 3285, 2958, 2928, 2858, 1623, 1538, 1260 cm^{-1}. **HR – MS** (ESI, positive ion mode) – *m/z* for $[C_{22}H_{39}N_5O_2+H]^+$ 406.3177, observed 406.3164.

1cc *N*-(phenylcarbamoyl)-*N*-benzyl-*N*''-(butylcarbamoyl)diethylenetriamine

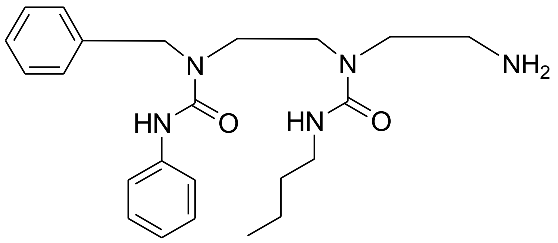

Following general procedure E, diurea **1cb** (170 mg, 0.33 mmol) in CH_2Cl_2 (10 mL) and trifluoroacetic acid (0.75 mL) were stirred for 12 hours at 20 °C. Water then CH_2Cl_2 were added, the organic layer was washed with aqueous $NaHCO_3$, dried over Na_2SO_4, filtered and concentrated under reduced pressure. The residue was purified by flash chromatography on silica gel (eluent: CH_2Cl_2 : MeOH 9:1) to give the unprotected amine (103 mg, 0.25 mmol, 76%). **^{1}H NMR** (400 MHz, $CDCl_3$) δ 7.98 (bs, NH_3^+), 7.35 (d, J = 7.3 Hz, 2H, 2 x CH_{Ph}), 7.32 – 7.16 (m, 7H, 7 x CH_{Ph}), 6.99 (t, J = 7.2 Hz, 1H, $CH_{Ph\ para}$), 6.80 (s, 1H, N*H*), 4.5 (s, 2H, CH_2Ph), 3.71 – 3.38 (s, 4H, 2 x CH_2), 3.37 – 3.25 (m, 2H, CH_2), 3.23 – 3.07 (m, 2H, CH_2), 3.05 – 2.78 (m, 2H, CH_2), 1.53-1.40 (m, 2H, CH_{2Bu}), 1.38 – 1.23 (m, 2H, CH_2CH_3), 0.86 (t, J = 7.1 Hz, 3H, CH_3Bu). **^{13}C NMR** (101 MHz, $CDCl_3$) δ 156.6 (2C, 2 x *C*=O), 138.7 (C_{Bn}), 136.5 (C_{Ph}), 129.4 (2C, 2 x $CH_{Ph\ ortho}$), 128.9 (2C, 2 x CH_{Bn}), 128.3 (2C, 2 x CH_{Bn}), 126.7 ($CH_{Bn\ para}$), 123.5 (CH_{Phpara}), 120.2 (2C, 2 x $CH_{Ph\ meta}$), 52.5 (CH_2Ph), 47.3 (CH_2N), 46.3 (CH_2N), 45.8 (CH_2N), 41.1 (CH_2N), 39.5 (CH_2N), 31.6 (CH_2), 20.1 (CH_2), 13.8 (CH_3). **HR – MS** (ESI, positive ion mode) – *m/z* for $[C_{23}H_{33}N_5O_2+H]^+$ 412.2707, observed 412.2690.

1b *N*-(isopropylcarbamoyl)-*N*-benzyl-*N*''-(hexylcarbamoyl)-*N'*-(3,5-*bis*(trifluoromethyl)phenylcarbamoyl)diethylenetriamine

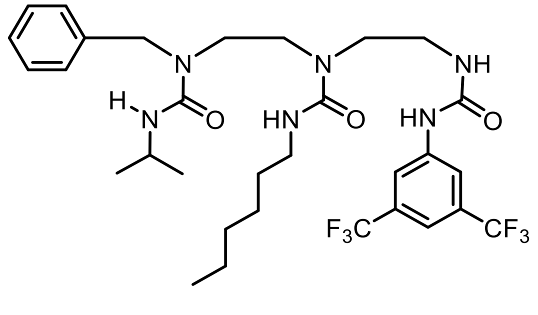

Following general procedure G using *N*-(isopropylcarbamoyl)-*N*-benzyl-*N*''-(hexylcarbamoyl)diethylenetriamine **1bc** (21 mg, 0.05 mmol) and 3,5-*bis*(trifluoromethyl)phenyl isocyanate (0.01 mL, 0.06 mmol), the title compound was obtained as a white solid (28 mg, 0.042 mmol, 84%). **^{1}H NMR** (400 MHz, $CDCl_3$) δ 8.82 (s, 1H, N*H*Ar), 7.84 (s, 2H, CH_{Ar}), 7.34 (s, 1H,

CH_{Ar}), 7.24 (t, *J* = 7.5 Hz, 2H, 2x CH_{Ph}), 7.16 (d, *J* = 7.5 Hz, 1H, CH_{Ph}), 7.14 – 7.08 (m, 2H, 2 x CH_{Ph}), 6.68 (s, 1H, N*H*CH_2), 6.48 (s, 1H, NH_{Hexyl}), 4.75 (m, 1 H, N*Hi*Pr), 4.37 (s, 2H, CH_2Ph), 3.92 – 3.73 (m, 1H, C*H*(CH_3)$_2$), 3.48 – 3.32 (m, 2H, CH_2), 3.32 – 3.25 (m, 2H, CH_2), 3.22 (m, 2H, CH_2), 3.14 (m, 4H, 2 x CH_2), 1.44 (dd, *J* = 7.4 Hz, 2H, CH_2), 1.26 – 1.05 (m, 6H, 3 x CH_2), 1.02 (d, *J* = 6.5 Hz, 6H, 2 x (CH_3)$_2$CH $_{iPr}$), 0.75 (t, *J* = 6.7 Hz, 3H, CH_{3hexyl}). **^{13}C NMR** (101 MHz, $CDCl_3$) δ 159.1 ($C{=}O_{Hexyl\ urea}$), 158.5 ($C{=}O_{iPr\ urea}$), 156.3 (C=O), 141.7 (C_{Ar}), 137.1 (C_{Ph}), 132.09 (q, *J* = 33.0 Hz, 2C, 2 x *C*CF_3), 129.2 (2C, 2 x CH_{Ph}), 128.0 (CH_{Ph}), 126.4 (2C, 2 x CH_{Ph}), 124.9 (q, *J* = 272.2 Hz, 2C, 2 x CF_3), 117.9 (CH_{Ar}), 114.9 (CH_{Ar}), 52.2 (CH_2Ph), 48.4 (CH_2), 47.9 (CH_2), 47.1 (CH_2), 43.1 (CH_{iPr}), 41.5 (CH_2), 39.3 (CH_2), 31.6 (CH_2), 29.8 (CH_2), 26.8 (CH_2), 23.2 (2C, 2 x CH_3CH_{iPr}), 22.6 (CH_2), 14.1 (CH_{3Hexyl}). **FTIR (neat)** vmax = 3313, 2957, 2927, 2857, 1694, 1609, 1571, 1276, 1138 cm^{-1}. **HR – MS** (ESI, positive ion mode) – *m/z* for $[C_{31}H_{42}F_6N_6O_3+H]^+$ = 661.3295, observed 661.3298. **MP** 167-169 °C.

1c *N*-(phenylcarbamoyl)-*N*-benzyl-*N*''-(butylcarbamoyl)-*N'*-(3,5-*bis*(trifluoromethyl)phenylcarbamoyl)diethylenetriamine

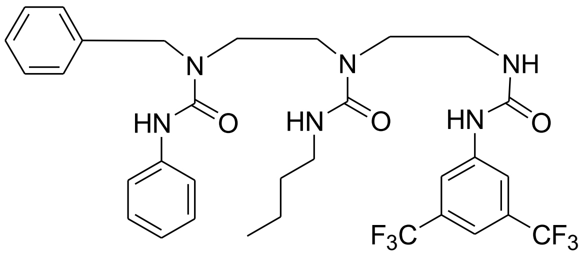

Following general procedure G, to a solution of primary amine **1cc** (100 mg, 0.24 mmol) in CH_2Cl_2 (5 mL) was added dropwise at 20 °C 3,5-*bis*(trifluoromethyl)phenyl isocyanate (0.042 mL, 0.24 mmol), the mixture was stirred for 12 hours. Addition of water was followed with extraction of the aqueous layer using CH_2Cl_2. The organic layer was dried over Na_2SO_4, filtered and concentrated under reduced pressure. The residue was purified by flash chromatography on silica gel (eluent: CH_2Cl_2 : MeOH 95:5) to afford the triurea **1c** (40 mg, 0.06 mmol, 25%) along with recovered starting material (40 mg, 40%). **^{1}H NMR** (500 MHz, $CDCl_3$) *δ* 8.39 (s, 1H, N*H*Ph(CF_3)$_2$), 7.66 (s, 2H, 2 x $CH_{Ph(CF3)2}$), 7.57 – 7.40 (m, 2H, 2 x $CH_{Ph\ ortho}$), 7.30 (s, 1H, $CH_{Ph(CF3)2}$), 7.25 (t, *J* = 7.6 Hz, 2H, 2 x $CH_{Ph\ meta}$), 7.22 – 7.07 (m, 6H, 6 x CH_{Ar}), 6.93 (t, *J* = 7.4 Hz, 1H, $CH_{Ph\ para}$), 6.29 (s, 1H, N*H*), 5.88 (s, 1H, N*H*Bu), 4.55 (s, 2H, CH_2Ph), 3.45 – 3.29 (m, 2H, CH_2N), 3.17 – 3.06 (m, 4H, 2 x CH_2N), 3.05 – 2.95 (m, 2H, CH_2N), 2.81 – 2.52 (m, 2H, CH_2N), 1.35 - 1.26 (m, 2H, CH_2), 1.20 – 1.12 (m, 2H, CH_2), 0.68 (t, *J* = 7.4 Hz, 3H, CH_3). **^{13}C NMR** (126 MHz, $CDCl_3$) *δ* 159.2 (*C*=O), 157.2 (*C*=O), 156.5 (*C*=O), 141.2 ($C_{Ph(CF3)2}$), 139.5 (C_{Ph}), 137.5 (C_{Bn}), 131.97 (q, *J* = 33.2 Hz, 2C, 2 x *C*CF_3), 129.2 (2C, 2 x CH_{Bn}), 129.0 (2C, 2 x CH_{Bn}), 127.9 (1C, $CH_{Bn\ para}$), 127.0 (2C, 2 x CH_{Ph}), 123.4 (q, *J* = 272.6 Hz, 2C, 2 x CF_3), 123.5 (CH_{Phpara}), 120.7 (2C, 2 x $CH_{Ph\ ortho}$), 117.9 (2C, 2 x $CH_{Ph(CF3)2}$), 115.1 (m, 1C, $CH_{para\ Ph(CF3)2}$), 51.6 (CH_2Ph), 47.4 (CH_2N), 47.2 (CH_2N), 46.5 (CH_2N), 41.0 (NHCH_2), 38.6 (CH_2N), 31.9 (CH_2), 20.1 (CH_2), 13.6 (CH_3). **HR – MS** (ESI, positive ion mode) – m/z for $[C_{32}H_{36}N_6O_3F_6Na]+$ 689.2645, found 689.2617. **MP** 174-176 °C.

Synthesis of triureas 1a, 1d, 1e, 3a-c

1aa *N*-benzyl-*N*'-(*tert*butoxycarbonyl)diethylenetriamine

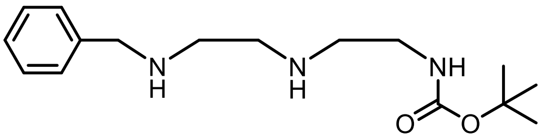

Following general procedure F with *N*-benzylethylenediamine (0.3 mL, 2 mmol), the title compound was obtained as an oil (233 mg, 1.2 mmol, 60%). **^{1}H NMR** (400 MHz, $CDCl_3$) δ 7.31 – 7.10 (m, 5H, 5 x CH_{Ar}), 5.12 – 4.90 (s, 1H, N*H*Boc), 3.78 – 3.65 (s, 2H, CH_2Ph), 3.41 – 3.30 (s, 2H, 2 x N*H*), 3.21 – 3.04 (dd, *J* = 5.9 Hz, 2H, CH_2N), 2.71 – 2.57 (m, 6H, 3 x CH_2N), 1.48 – 1.27 (s, 9H, (CH_3)$_3$C). **^{13}C NMR** (101 MHz, $CDCl_3$) δ 156.2 (C=O), 140.4 (C_{Ar}), 128.5 (2C, 2 x CH_{Ar}), 128.2 (2C, 2 x CH_{Ar}), 127.0 (CH_{Ar}), 79.2 (*C*(CH_3)$_3$), 54.0 (CH_2Ph), 50.4 (CH_2N), 49.1 (CH_2N), 49.0 (CH_2N), 48.8 (CH_2N), 28.5 (3C, (CH_3)$_3$C). **FTIR (neat)** vmax = 3327, 2974, 2930, 2882, 1697, 1495, 1452, 1364, 1249, 1168 cm^{-1}. **HR – MS** (ESI, positive ion mode) – *m/z* for $[C_{16}H_{27}N_3O_2+H]^+$ 294.2176, observed 294.2168.

3aa *N*-ethyl-*N*'-(tertbutoxycarbonyl)diethylenetriamine

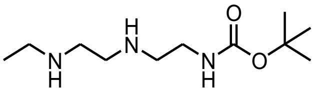

Following general procedure F with *N*-ethylethylenediamine (0.3 mL, 2 mmol), the carbamate was isolated as a colourless oil (268 mg, 1.16 mmol, 58%). **^{1}H NMR** (400 MHz, $CDCl_3$) δ 5.45 (s, 1H, N*H*), 3.71 (dd, *J* = 7.0 Hz, 2H, NCH_2), 3.23 (m, 2H. NCH_2), 3.00 – 2.91 (m, 4H, 2 x NCH_2), 2.76 (t, *J* = 5.4 Hz, 2H, NCH_2), 1.44 (s, 9H, C(CH_3)$_3$), 1.23 (t, *J* = 6.5 Hz, 3H, CH_3). **^{13}C NMR** (101 MHz, $CDCl_3$) δ 156.9 (*C*=O), 81.4 (*C*(CH_3)$_3$), 58.4 (NCH_2), 49.1 (NCH_2), 47.1 (NCH_2), 46.0 (NCH_2), 40.1 (NCH_2), 28.5 (3C, C(CH_3)), 18.4 (CH_3). **FTIR (neat)** vmax = 3396, 2977, 2969, 1696. **HR-MS** (ESI, positive ion mode) – *m/z* for $[C_{11}H_{25}N_3O_2+H]^+$ 232.2025, observed 232.2019.

1ab *N,N''-bis*(hexylcarbamoyl)-*N*-benzyl-*N*'-(tertbutoxycarbonyl)diethylenetriamine

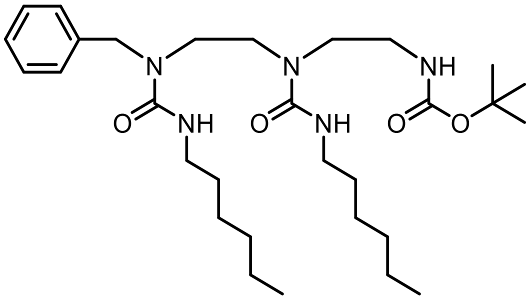

Following general procedure D with *N*-benzyl-*N*'-(*tert*butoxycarbonyl)diethylenetriamine **1aa** (233 mg, 0.8 mmol) and hexyl isocyanate (0.34 mL, 2.38 mmol, 3 equiv.), the title compound (400 mg, 0.744 mmol, 93%) was obtained as a colourless oil after silica gel flash chromatography (eluent: CH_2Cl_2:MeOH 98:2). **^{1}H NMR** (400 MHz, $CDCl_3$) δ 7.28 – 7.22 (m, 2H, 2 x CH_{Ar}), 7.21 – 7.17 (m, 1H, CH_{Ar}), 7.17 – 7.11 (m, 2H, 2 x CH_{Ar}), 6.16 (s, 1H, N*H*), 5.38 (s, 1H, N*H*), 5.24 (m, 1H, N*H*), 4.38 (s, 2H, CH_2Ph), 3.27 (dd, *J* = 9.5, 5.5 Hz, 2H, CH_2), 3.21 – 3.07 (m, 8H, 4 x CH_2), 3.06 – 2.97 (m, 2H, CH_3), 1.51 – 1.31 (m, 4H, 2 x CH_2), 1.34 (s, 9H, (CH_3)$_3$C), 1.30 – 1.07 (m, 12H, 6 x CH_2), 0.86 – 0.72 (m, 6H, 2 x CH_3). **^{13}C NMR** (101 MHz, $CDCl_3$) δ 158.7 (2 x C=O), 156.7 (C=O), 138.1 (C_{Bn}), 128.8 (2C, 2 x CH_{Bn}), 127.5 (CH_{Bn}), 127.0 (2C, 2 x CH_{Bn}), 79.6 (*C*(CH_3)$_3$), 51.6 (CH_2Ph), 47.5 (CH_2N), 46.8 (2C, 2 x CH_2N), 41.0 (2C, 2 x CH_2), 39.9 (CH_2NHBoc), 31.6 (2C, 2 x CH_2), 29.9 (2C, 2 x CH_2), 28.4 (3C, (CH_3)$_3$C), 26.7 – 26.6 (2C, 2 x CH_2), 22.6 (2C, 2 x CH_2), 14.1 – 14.0 (2C, 2 x CH_3). **FTIR (neat)** vmax = 3301, 2956,

2928, 2858, 1689, 1626, 1536, 1219 cm^{-1}. **HR – MS** (ESI, positive ion mode) – *m/z* for $[C_{30}H_{53}N_5O_4+H]^+$ 548.4170, observed 548.4155.

1db *N*-(phenylcarbamoyl)-*N*-benzyl-*N*'-(tertbutoxycarbonyl)-*N*''-(phenylcarbamoyl)diethylenetriamine

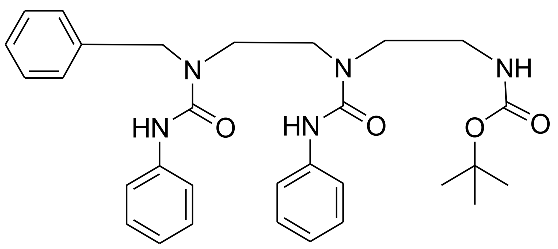

Following general procedure D with the *N*-benzyl-*N*'-(*tert*butoxycarbonyl)diethylenetriamine **1aa** (187 mg, 0.45 mmol) in CH_2Cl_2 (3 mL) and phenyl isocyanate (0.13 mL, 1.2 mmol), the product was obtained after purification by silica gel flash chromatography (CH_2Cl_2:MeOH 9:1) (72 mg, 0.14 mmol, 30%). **^{1}H NMR** (400 MHz, $CDCl_3$) δ 8.50 (s, 1H, N*H*Ph), 7.57 (d, *J* = 8.0 Hz, 2H, 2 x C*H* $_{Ph1ortho}$), 7.50 (d, *J* = 7.2 Hz, 2H, 2 x C*H*$_{Ph2\ ortho}$), 7.27 – 7.14 (m, 10H, N*H*Ph, 4 x C*H*$_{Ph\ meta}$, 5 x C*H*$_{Bn}$), 6.95 – 6.88 (m, 2H, 2 x C*H*$_{Ph\ para}$), 5.14 (s, 1H, N*H*Boc), 4.52 (s, 2H, C*H*$_2$Ph), 3.40 (dd, *J* = 9.6, 5.9 Hz, 2H, C*H*$_2$NBn), 3.23 (q, *J* = 8.1, 6.9 Hz, 4H, 2 x C*H*$_2$N), 3.05 (t, *J* = 7.3 Hz, 2H, C*H*$_2$NH), 1.35 (s, 9H, (C*H*$_3$)$_3$C). **^{13}C NMR** (101 MHz, $CDCl_3$) δ 157.2 (*C*=O), 156.3 (*C*=O), 156.2 (*C*=O), 139.9 (2C, 2 x *C*$_{Ph}$), 138.0 (1C, *C*$_{Bn}$), 129.0 (2C, 2 x C*H*$_{Ph}$), 128.8 (2C, 2 x C*H*$_{Ph}$), 128.7 (2C, 2 x C*H*$_{Ph}$), 127.8 (1C, *C*H$_{Bnpara}$), 127.5 (2C, 2 x C*H*$_{Ph}$), 122.7 (2C, 2 x *C*H$_{Ph1-2\ para}$), 119.9 (2C, 2 x *C*H $_{Ph2\ ortho}$), 119.7 (2C, 2 x *C*H $_{Ph1\ ortho}$), 80.4 (1C, *C*(CH$_3$)$_3$), 51.5 (1C, *C*H$_2$Ph), 47.8 (1C, *C*H$_2$NBn), 46.2 (2C, 2 x *C*H$_2$N), 40.1 (1C, *C*H$_2$NH), 28.4 (3C, (*C*H$_3$)$_3$C). **HR – MS** (ESI, positive ion mode) – *m/z* for $[C_{30}H_{37}N_5O_4Na]^+$ 554.2738, observed 554.2720.

1eb *N,N''-bis*(4-butoxyphenylcarbamoyl)-*N*-benzyl-*N*'-(*tert*butoxycarbonyl)diethylenetriamine

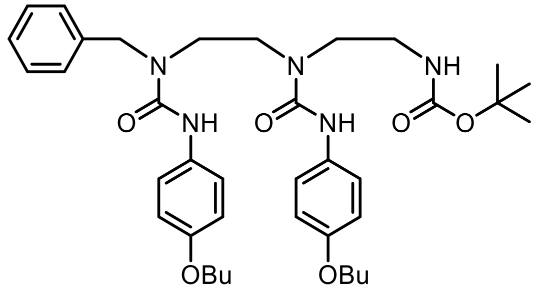

Following general procedure D with *N*-benzyl-*N*'-(*tert*butoxycarbonyl)diethylenetriamine **1aa** (60 mg, 0.2 mmol) and para-*n*-butoxylphenyl isocyanate (0.11 mL, 0.6 mmol, 3 equiv.), the title compound was obtained as a white solid after the residue was purified by silica gel flash chromatography (eluent: CH_2Cl_2:MeOH 95:5) (130 mg, 0.194 mmol, 97%). **^{1}H NMR** (500 MHz, $CDCl_3$) δ 8.53 – 8.32 (m, 1H, N*H*), 7.57 – 7.50 (m, 2H, 2 x C*H*$_{Ar}$), 7.42 (d, *J* = 8.1 Hz, 2H, 2 x C*H*$_{Ar}$), 7.40 – 7.35 (m, 2H, 2 x C*H*$_{Ar}$), 7.35 – 7.29 (m, 3H, 3 x C*H*$_{Ar}$), 6.88 – 6.79 (m, 4H, 4 x C*H*$_{Ar}$), 5.06 (s, 1H, N*H*), 4.63 (s, 2H, C*H*$_2$Ph), 3.99 – 3.91 (m, 4H, 2 x C*H*$_2$O), 3.53 (t, *J* = 7.6 Hz, 2H, C*H*$_2$N), 3.41 – 3.32 (m, 4H, 2 x C*H*$_2$N), 3.19 (dd, *J* = 6.8 Hz, 2H, C*H*$_2$N), 1.82 – 1.71 (m, 4H, 2 x C*H*$_2$), 1.55 – 1.47 (m, 4H, 2 x C*H*$_2$), 1.46 (s, 9H, (C*H*$_3$)$_3$C), 1.02 – 0.95 (m, 6H, 2 x C*H*$_3$). **^{13}C NMR** (126 MHz, $CDCl_3$) δ 157.1 (C=O), 156.5 (C=O), 156.5 (C=O), 155.1 (*C*$_{Ar}$), 155.0 (*C*$_{Ar}$), 138.1 (*C*$_{Bn}$), 133.0 (*C*$_{Ar}$), 132.8 (*C*$_{Ar}$), 127.8 (2C, 2 x *C*H$_{Bn}$), 127.5 (*C*H$_{Bn}$), 121.7 (2C, 2 x *C*H$_{Bn}$), 121.3 (2C, 2 x *C*H$_{Ar}$), 114.8 (2C, 2 x *C*H$_{Ar}$), 114.8 (4C, 4 x *C*H$_{Ar}$), 80.5 (*C*(CH$_3$)$_3$), 68.1 (2C, 2 x *C*H$_2$), 51.7 (*C*H$_2$Ph), 47.9 (*C*H$_2$N), 47.7 (*C*H$_2$N), 46.6 (*C*H$_2$N), 40.2 (*C*H$_2$NNBoc), 31.5 (2C, 2 x *C*H$_2$), 28.5 (3C, (*C*H$_3$)$_3$C), 19.4

(2C, 2 x CH_2), 14.0 (2C, 2 x CH_3). **FTIR (neat)** vmax = 3292, 2957, 2931, 1643, 1510, 1220 cm^{-1}. **HR – MS** (ESI, positive ion mode) – *m/z* for $[C_{38}H_{53}N_5O_6+H]^+$ 676.4069, observed 676.4057. **MP** 81-83 °C.

3ab *N*-ethyl-*N,N''-bis*(phenylcarbamoyl)-*N'*-(tertbutoxycarbonyl)diethylenetriamine

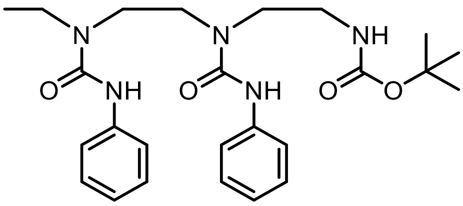

Following general procedure D with *N*-ethyl-*N'*-(*tert*butoxycarbonyl)diethylenetriamine **1aa** (200 mg, 0.87 mmol) and phenyl isocyanate (0.28 mL, 2.5 mmol, 3 equiv.) (the mixture was stirred at 80 °C for 16 hours), the residue was purified using silica gel flash chromatography (CH_2Cl_2:MeOH 95:5) to afford the title compound as a white solid (200 mg, 0.42 mmol, 50%). **^{1}H NMR** (400 MHz, $CDCl_3$) δ 8.82 – 8.52 (s, 1H, N*H*), 8.15– 7.84 (s, 1H, N*H*), 7.75 – 7.65 (d, *J* = 8.0 Hz, 2H, 2 x C*H*A_r), 7.65 – 7.53 (d, *J* = 8.0 Hz, 2H, 2 x C*H*A_r), 7.38 – 7.21 (m, 4H, 4 x C*H*A_r), 7.09 – 6.97 (m, 2H, 2 x C*H*A_r), 5.18 (t, *J* = 5.6 Hz, 1H, N*H*), 3.54 – 3.37 (m, 8H, 4 x CH_2), 3.27 (dd, *J* = 6.9, 6.6 Hz, 2H, CH_2NH), 1.49 (s, 9H, $(CH_3)_3C$), 1.27 – 1.17 (t, *J* = 7.1 Hz, 3H, CH_3CH$_2$). **^{13}C NMR** (101 MHz, $CDCl_3$) δ 157.2 (C=O_{Boc}), 156.3 (C=O), 155.7 (C=O), 140.0 (C_{Ar}), 139.2 (C_{Ar}), 128.8 (2C, 2 x CH_{Ar}), 128.8 (2C, 2 x CH_{Ar}), 122.7 (2C, 2 x CH_{Ar}), 119.9 (2C, 2 x CH_{Ar}), 119.7 (2C, 2 x CH_{Ar}), 80.5 ($C(CH_3)_3$), 47.9 (2C, 2 x CH_2), 46.1 (CH_2), 43.2 (CH_2), 40.3 (CH_2), 28.5 (3C, $(CH_3)_3C$), 14.3 (CH_3). **FTIR (neat)** vmax = 3311, 3137, 1710, 1664 cm^{-1}. **HR – MS** (ESI, positive ion mode) – *m/z* for $[C_{25}H_{35}N_5O_4+Na]^+$ = 492.2581, observed 492.2588. **MP** 130-131 °C.

1ac *N,N''-bis*(hexylcarbamoyl)-*N*-benzyldiethylenetriamine, trifluoroacetic salt

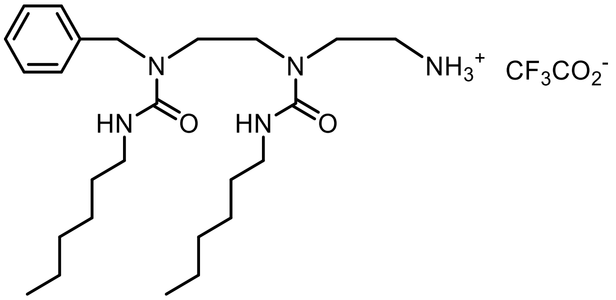

Following general procedure E, *N,N''-bis*(hexylcarbamoyl)-*N*-benzyl-*N'*-(*tert*butoxycarbonyl)diethylenetriamine **1ab** (400 mg, 0.73 mmol) afforded the title compound (325 mg, 0.73 mmol, quantitative yield) as an oil. **^{1}H NMR** (500 MHz, $CDCl_3$) δ 9.98 (s, 2H, N*H*), 8.17 (d, *J* = 6.0 Hz, 3H, NH_3), 7.38 (dd, *J* = 8.1, 6.7 Hz, 2H, 2 x CH_{Ph}), 7.33 – 7.22 (m, 1H, CH_{Ph}), 7.20 (dd, *J* = 7.1, 1.8 Hz, 2H, 2 x CH_{Ph}), 6.93 (s, 1H, N*H*), 4.62 (m, 1H, N*H*), 4.42 (s, 2H, CH_2Ph), 3.59 – 3.39 (m, 4H, 2 x CH_2N), 3.36 (dd, *J* = 9.3, 5.7 Hz, 2H, CH_2NBn), 3.23 – 2.95 (m, 6H, 3 x CH_2NH), 1.64 – 1.43 (m, 2H, CH_2), 1.43 – 1.05 (m, 14H, 7 x CH_2), 0.88 (6H, 2 x CH_3). **^{13}C NMR** (126 MHz, $CDCl_3$) δ 161.0 (q, *J* = 38.2 Hz, *C*=OCF_3), 159.8 (C=O), 159.0 (C=O), 136.5 (C_{Ar}), 129.3 (2C, 2 x CH_{Ar}), 128.1 (CH_{Ar}), 126.4 (2C, 2 x CH_{Ar}), 115.8 (q, *J* = 288.8 Hz, CF_3), 52.3 (CH_2Ph), 47.4 (CH_2N), 46.5 (CH_2N), 46.3 (CH_2N), 41.5 (CH_2NH), 42.0 (CH_2NH), 39.8 (CH_2NH_2), 31.5 (CH_2), 31.4 (CH_2), 29.7 (CH_2), 29.5 (CH_2), 26.6 (CH_2), 26.3 (CH_2), 22.4 (2C, 2 x CH_2), 13.9 (2C, 2 x CH_3). **FTIR (neat)** vmax = 2956, 2929, 2858, 1678, 1627, 1538, 1201, 1174 cm^{-1}. **HR – MS** (ESI, positive ion mode) – *m/z* for $[C_{25}H_{46}N_5O_2]^+$ 448.3646, observed 448.3629.

1dc *N*-(phenylcarbamoyl)-*N*-benzyl-*N*''-(phenylcarbamoyl)diethylenetriamine

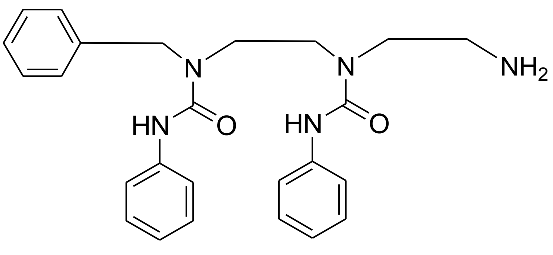

Following general procedure E, diurea **1db** (72 mg, 0.13 mmol) in CH_2Cl_2 (5 mL) and trifluoroacetic acid (0.35 mL) was stirred for 12 hours at 20 °C. Water then CH_2Cl_2 were added, the organic layer was washed with aqueous $NaHCO_3$, dried over Na_2SO_4, filtered and concentrated under reduced pressure. The residue was purified by flash chromatography on silica gel (eluent: CH_2Cl_2:MeOH 9:1) to give the unprotected amine (50 mg, 0.11 mmol, 86%). **^{1}H NMR** (400 MHz, $CDCl_3$) δ 8.88 (s, 1H, NH), 8.09 – 7.57 (m, 2H, 2 x CH_{Ph}), 7.53 – 7.33 (m, 2H, 2 x CH_{Ph}), 7.31 – 7.01 (m, 9H, 9 x CH_{Ph}), 6.92 – 6.80 (m, 2H, 2 x C$H_{Ph\ para}$), 6.70 (s, 1H, N*H*), 4.35 (s, 2H, CH_2Ph), 3.29 (s, 4H, 2 x CH_2N), 3.17 (s, 2H, CH_2N), 2.81 (s, 2H, CH_2N). **^{13}C NMR** (101 MHz, $CDCl_3$) δ 156.6 (2C, 2 x *C*=O), 139.3 (C_{Ph}), 138.6 (C_{Ph}), 136.3 (C_{Ph}), 129.4 (2C, 2 x CH_{Ph}), 129.0 (2C, 2 x CH_{Ph}), 128.9 (2C, 2 x CH_{Ph}), 128.4 ($CH_{Bn\ para}$), 126.7 (2C, 2 x CH_{Ph}), 123.6 ($CH_{Ph\ para}$), 123.5 ($CH_{Ph\ para}$), 121.0 (2C, 2 x CH_{Ph}), 120.2 (2C, 2 x CH_{Ph}), 52.6 (CH_2Ph), 47.7 (CH_2N), 46.0 (CH_2N), 45.9 (CH_2N), 39.4 (CH_2N). **HR – MS** (ESI, positive ion mode) – *m/z* for $[C_{25}H_{29}N_5O_2+H]^+$ 432.2394, observed 432.2379.

1ec *N,N''-bis*(4-butoxyphenylcarbamoyl)-*N*-benzyldiethylenetriamine, trifluoroacetic salt

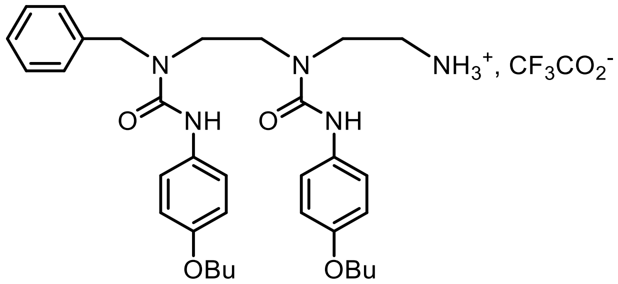

Following general procedure E, *N,N''-bis*(4-butoxyphenylcarbamoyl)-*N*-benzyl-*N*'-(*tert*butoxycarbonyl)diethylenetriamine **1eb** (96 mg, 0.14 mmol) afforded the title compound (70 mg, 0.122 mmol, 87%) as a white solid. **^{1}H NMR** (500 MHz, $CDCl_3$) δ 8.84 (s, 1H, N*H*), 7.96 (s, 3H, NH_3^+), 7.44 – 7.33 (m, 4H, 4 x C*H*Ar), 7.30 (m, 1H, C*H*Ar), 7.23 (d, *J* = 7.5 Hz, 2H, 2 x C*H*Ar), 7.18 – 7.01 (m, 2H, 2 C*H*Ar), 6.92 – 6.57 (m, 4H, 4 C*H*Ar), 4.58 (s, 1H, N*H*), 4.47 (s, 2H, CH_2Ph), 3.87 (t, *J* = 6.5 Hz, 2H, CH_2O), 3.83 (t, *J* = 6.6 Hz, 2H, CH_2O), 3.60 – 3.16 (m, 8H, 4 x CH_2), 3.06 – 2.66 (m, 2H, CH_2), 1.77 – 1.70 (m, 2H, CH_2), 1.67 (m, 2H, CH_2), 1.51 – 1.36 (m, 4H, 2 x CH_2), 0.97 (t, *J* = 7.4 Hz, 3H, CH_3), 0.93 (t, *J* = 7.4 Hz, 3H, CH_3). **^{13}C NMR** (126 MHz, $CDCl_3$) δ 162.2 (q, *J* = 36.1 Hz, C=OCF_3), 157.5 (C=O), 156.9 (C=O), 155.6 (2 x C_{Ar}), 136.5 (C_{Bn}), 131.9 (C_{Ar}), 131.4 (C_{Ar}), 129.3 (2C, 2 x CH_{Bn}), 128.2 (CH_{Bn}), 126.7 (2C, 2 x CH_{Bn}), 122.9 (2C, 2 x CH_{Ar}), 122.3 (2C, 2 x CH_{Ar}), 116.4 (q, *J* = 290.8 Hz, CF_3), 114.7 (2C, 2 x CH_{Ar}), 114.6 (2C, 2 x CH_{Ar}), 68.0 (2C, 2 x CH_2), 52.5 (CH_2Ph), 50.4 (CH_2N), 47.5 (CH_2N), 46.1 (CH_2N), 45.7 (CH_2N), 39.1 (CH_3NBn), 31.4 (2C, 2 x CH_2), 19.3 (2C, 2 x CH_2), 13.9 (2C, 2 x CH_3). **FTIR (neat)** vmax = 3320, 2959, 2930, 2872, 1644, 1509, 1219 cm^{-1}. **HR – MS** (ESI, positive ion mode) – *m/z* for $[C_{33}H_{46}N_5O_4]^+$ 576.3544, observed 576.3529. **MP** 64-65 °C.

3ac *N,N''-bis*(phenylcarbamoyl)-*N*-ethyldiethylenetriamine, trifluoroacetic salt

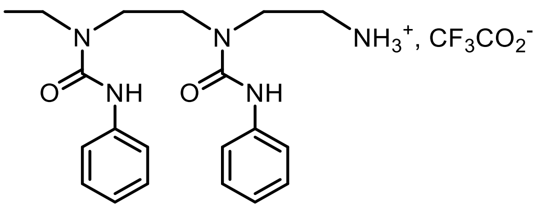

Following general procedure E, *N,N''-bis*(phenylcarbamoyl)-*N*-ethyl-*N*'-(*tert*butoxycarbonyl)diethylenetriamine **3ab** (18 mg, 0.038 mmol) afforded the product (20 mg, quant) as a white solid. **^{1}H NMR** (400 MHz, $CDCl_3$) *δ* 9.18 (s, 1H, N*H*), 7.45-7.33 (m, 4H, 4 x CH_{Ph}), 7.21 – 7.09 (m, 4H, 4 x CH_{Ph}), 6.95 – 6.84 (m, 2H, 4 x CH_{Phpara}), 5.88 (bs, 3H, NH_3^+), 3.41 – 3.31 (m, 2H, CH_2), 3.31 – 3.13 (m, 6H, 6 x CH_2), 2.91 – 2.80 (m, 2H, CH_2), 1.05 (t, *J* = 7.1 Hz, 3H). **^{13}C NMR** (101 MHz, $CDCl_3$) *δ* 157.5 (*C*=O), 155.9 (*C*=O), 139.7 (C_{Ph}), 139.3 (C_{Ph}), 128.9 (2 x CH_{Ph}), 128.8 (2 x CH_{Ph}), 123.0 (CH_{Ph}), 123.0 (CH_{Ph}), 120.3 ((2 x CH_{Ph})), 120.2 (2 x CH_{Ph}), 47.8 (CH_2), 46.9 (CH_2), 46.1 (CH_2), 43.4 (CH_2), 39.8 (CH_2), 14.1 (CH_3).

1a *N,N''-bis*(hexylcarbamoyl)-*N*-benzyl-*N'*-(3,5-*bis*(trifluoromethyl)phenylcarbamoyl)diethylenetriamine

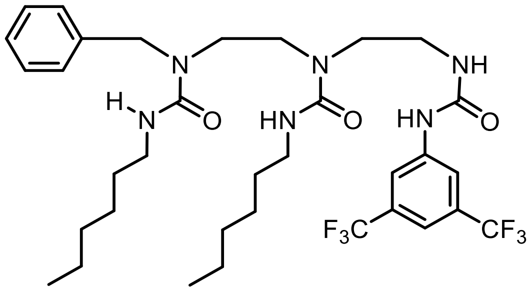

Following general procedure G with *N,N''-bis*(hexylcarbamoyl)-*N*-benzyldiethylenetriamine **1ac** (23 mg, 0.06 mmol) and 3,5-*bis*(trifluoromethyl)phenyl isocyanate (0.011 mL, 0.06 mmol), the title urea was obtained as a white solid (40 mg, 0.058 mmol, 97%). **^{1}H NMR** (500 MHz, CD_2Cl_2) δ 9.11 (s, 1H, N*H*Ar), 7.95 (s, 2H, CH_{Ar}), 7.42 (s, 1H, CH_{Ar}), 7.29 (m, 2H, 2 x CH_{Ph}), 7.24 – 7.19 (m, 2H, 2 x CH_{Ph}), 7.18 (d, *J* = 7.6 Hz, 1H, CH_{Ph}), 6.83 – 6.17 (m, 2H, 2 x N*H*), 4.48 (s, 2H, CH_2Ph), 3.41 – 3.34 (m, 2H, CH_2NBn), 3.29 (m, 2H, CH_2N), 3.25 – 3135 (m, 6H, 3 x CH_2), 3.09 (m, 2H, CH_2NH), 1.49 (m, 4H, 2 x CH_2), 1.33 – 1.12 (m, 12H, 6 x CH_2), 0.83 (m, 6H, 2 x C$H_{3\ hexyl}$). **^{13}C NMR** (126 MHz, CD_2Cl_2) δ 159.6 (C=O_{urea}), 159.6 (C=O_{urea}), 156.7 (C=$O_{Ar\ urea}$), 142.5 (C_{Ar}), 138.4 (C_{Ph}), 132.3 (q, *J* = 33.0 Hz, 2C, 2 x $C_{Ar}CF_3$), 129.5 (2C, 2 x CH_{Ph}), 128.1 (CH_{Ph}), 127.0 (2C, 2 x CH_{Ph}), 124.1 (q, *J* = 272.5 Hz, 2C, 2 x CF_3), 118.2 (2C, 2 x CH_{Ar}), 115.0 (CH_{Ar}), 52.5 (CH_2Ph), 48.4 (CH_2N), 48.0 (CH_2NBn), 47.6 (CH_2N), 41.8 (CH_{2hexyl}NH), 41.7 (CH_{2hexyl}NH), 39.8 (CH_2NH), 32.1 (CH_{2Hexyl}), 32.1 (CH_{2Hexyl}), 30.4 (CH_{2Hexyl}), 30.3 (CH_{2Hexyl}), 27.2 (CH_{2Hexyl}), 27.1 (CH_{2Hexyl}), 23.1 (2C, 2 x $CH_{2\ Hexyl}$), 14.3 (2C, 2 x CH_{3Hexyl}). **FTIR (neat)** vmax = 3312, 3088, 2958, 2930, 2866, 1692, 1611, 1572, 1387, 1276, 1137 cm^{-1}. **HR – MS** (ESI, positive ion mode) – *m/z* for $[C_{34}H_{48}F_6N_6O_3+H]^+$ 703.3765, observed 703.3747 ; *m/z* for $[C_{34}H_{48}F_6N_6O_3+Na]^+$ 725.3584, observed 725.3561. **MP** 137-139 °C.

1d ***N*-(phenylcarbamoyl)-*N*-benzyl-*N*''-(phenylcarbamoyl)-*N'*-(3,5-*bis*(trifluoromethyl)phenylcarbamoyl)diethylenetriamine**

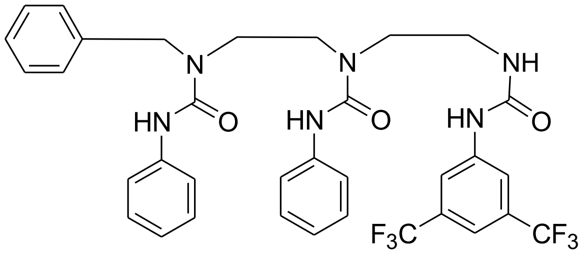

Following general procedure G with a solution of primary amine **1dc** (50 mg, 0.116 mmol) in CH_2Cl_2 (3 mL) and 3,5-*bis*(trifluoromethyl)phenyl isocyanate (0.022 mL, 0.13 mmol), the residue was purified by flash chromatography on silica gel (eluent: CH_2Cl_2 : MeOH 95:5) to afford the triurea **1d** (35 mg, 0.051 mmol, 44%) along with recovered starting material (20 mg, 40%). **^{1}H NMR** (500 MHz, Acetone d6) δ 9.12 (s, 1H, N*H*Ph(CF_3)$_2$), 8.20 (s, 2H, 2 x C$H_{Ph(CF3)2}$), 7.80 (d, *J* = 8.1 Hz, 2H, 2 x C$H_{Ph\ ortho}$), 7.78-7.74 (m, 2H, 2 x C$H_{Ph\ ortho}$), 7.56 (s, 1H, C$H_{Ph(CF3)2}$), 7.41 – 7.29 (m, 4H, 4 x CH $_{Bn}$), 7.28 – 7.21 (m, 5H, 4 x C$H_{Ph\ meta}$, CH $_{Bn\ para}$), 7.02 – 6.92 (m, 2H, 2 x C$H_{Ph\ para}$), 4.69 (s, 2H, CH_2Ph), 3.66 – 3.48 (m, 6H, 3 x CH_2N), 3.41 (q, *J* = 7.0, 6.0 Hz, 2H, CH_2N). **^{13}C NMR** (126 MHz, Acetone d6) δ 156.3 (3C, 3 x *C*=O), 155.8 (1C, *C*=ONBn), 142.3 ($C_{Ph(CF3)2}$), 141.0 (C_{Ph}), 140.7 (C_{Ph}), 139.0 (C_{Bn}), 131.5 (q, *J* = 32.7 Hz, 2C, 2 x *C*CF_3), 128.5 (2C, 2 x *C*$H_{Ph\ meta}$), 128.4 (2C, 2 x *C*$H_{Ph\ metao}$), 128.3 (1C, *C*$H_{Bn\ para}$), 127.5 (2C, 2 x *C*H_{Bn}), 127.1 (2C, 2 x *C*H_{Bn}), 123.7 (q, *J* = 272.0 Hz, 2C, 2 x *C*F_3), 122.1 (1C, *C*$H_{Ph\ para}$), 121.9 (1C, *C*$H_{Ph\ para}$), 119.4 (2C, 2 x *C*$H_{Ph\ ortho}$), 119.2 (2C, 2 x *C*$H_{Ph\ ortho}$), 117.9 (2C, 2 x *C*H $_{Ph(CF3)2\ ortho}$), 114.3 (*C*H $_{Ph(CF3)2\ para}$), 50.6 (*C*H_2Ph), 47.6 (1C, *C*H_2N), 47.5 (1C, *C*H_2N),39.6 (1C, *C*H_2N), 39.5 (1C, *C*H_2N). **HR – MS** (ESI, positive ion mode) – m/z for [$C_{34}H_{32}N_6O_3F_6$Na]+ 709.2332, found 709.2302. **MP** 216-218 °C.

1e *N,N''-bis*(4-butoxyphenylcarbamoyl)-*N*-benzyl-*N'*-(3,5-*bis*(trifluoromethyl)phenylcarbamoyl) diethylenetriamine

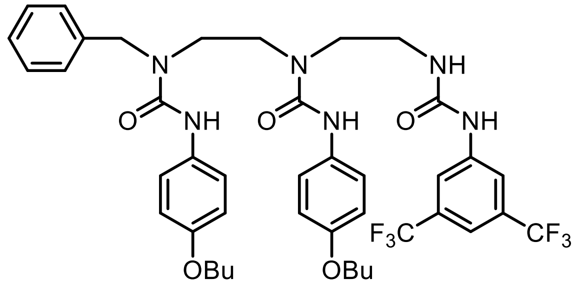

Following general procedure G using *N,N''-bis*(4-butoxyphenylcarbamoyl)-*N*-benzyldiethylenetriamine **1ec** (24 mg, 0.042 mmol) and 3,5-*bis*(trifluoromethyl)phenyl isocyanate (0.007 mL, 0.042 mmol), the title compound was obtained (28 mg, 0.032 mmol, 78%) as a white solid. **^{1}H NMR** (500 MHz, CD_2Cl_2) δ 9.33 (bs, 1H, NH_{urea}), 8.82 – 8.44 (m, 2H, 2 x NH_{urea}), 7.77 (s, 2H, 2 x CH_{Ar}), 7.57 – 7.48 (m, 2H, 2 x CH_{Ar}), 7.46 (s, 1H, CH_{Ar}), 7.48 – 7.39 (m, 2H, 2 x CH_{Ar}), 7.35 – 7.29 (m, 2H, 2 x CH_{Bn}), 7.26 (t, *J* = 7.6 Hz, 2H, 2 x CH_{Bn}), 7.07 (m, 1H, C*H*Bn), 6.86 – 6.67 (m, 4H, 4 x CH_{Ar}), 6.54 (s, 1H, N*H*CH_2), 4.69 (s, 2H, CH_2Ph), 3.92 (t, *J* = 6.6 Hz, 2H, CH_2O), 3.85 (t, *J* = 6.6 Hz, 2H, CH_2O), 3.58 – 3.47 (m, 2H, CH_2), 3.47 – 3.34 (m, 2H, CH_2), 3.27 – 3.10 (m, 2H, CH_2) , 2.88 – 2.65 (m, 2H, CH_2NH), 1.81 – 1.68 (m, 4H, 2 x CH_2), 1.57 – 1.42 (m, 4H, 2 x CH_2), 1.03 – 0.96 (m, 6H, 2 x CH_3). **^{1}H NMR** (400 MHz, $CDCl_3$) δ 8.18 (s, 2H, 2N*H*), 7.68 (s, 3H, N*H*, 2 x C*H*Ar), 7.20 (d, *J* = 7.5 Hz, 10H, 10 x C*H*Ar), 6.71 (d, *J* = 8.5 Hz, 2H, 2 x C*H*Ar), 6.65 (d, *J* = 8.3 Hz, 2H, 2 x C*H*Ar), 6.48 (s, 1H, NHCH_2), 4.59 (s, 2H, CH_2Ph), 3.83 (dt, *J* = 10.9, 6.5 Hz, 4H), 3.58 (m, 2H), 3.40 (s, 4H), 2.98 (m, 2H), 1.82 – 1.65 (m, 4H, 2 x CH_2), 1.45 (hd, *J* = 7.4, 3.2 Hz, 4H, 2 x CH_2), 0.96

(td, J = 7.4, 2.7 Hz, 6H, 2 x CH_3). **^{13}C NMR** (126 MHz, CD_2Cl_2) δ 158.1 (C=O), 157.4 (C=O), 157.3 (C=O), 156.3 (C_{Ar}), 155.6 (C_{Ar}), 141.8 (C_{Ar}), 138.5 (C_{Bn}), 133.2 (C_{Ar}), 132.5 (C_{Ar}), 132.1 (q, J = 32.9 Hz, 2C, 2 x CCF_3), 129.6 (2C, 2 x CH_{Bn}), 128.0 (CH_{Bn}), 127.0 (2C, 2 x CH_{Bn}), 124.0 (q, J = 272.6 Hz, 2C, 2 x CF_3), 123.3 (2C, 2 x CH_{Ar}), 121.6 (2C, 2 x CH_{Ar}), 118.5 (2C, 2 x CH_{Ar}), 115.4 (CH_{Ar}), 115.2 (2C, 2 x CH_{Ar}), 115.0 (2C, 2 x CH_{Ar}), 68.4 (2C, 2 x CH_2O), 52.2 (CH_2Ph), 48.3 (CH_2N), 48.2 (CH_2N), 47.2 (CH_2NBn), 39.6 (CH_2NH), 31.9 (2C, 2 x CH_2), 19.7 (2C, 2 x CH_2), 14.2 (2C, 2 x CH_3). **FTIR (neat)** vmax = 3298, 2963, 2937, 2872, 1640, 1552, 1511, 1278, 1130 cm^{-1}. **HR – MS** (ESI, positive ion mode) – *m/z* for $[C_{42}H_{48}F_6N_6O_5+H]^+$ 831.3663, observed 831.3654. **MP** 170-171 °C.

3a *N*-Ethyl-*N,N''-bis*(phenylcarbamoyl)-*N*'-(4-trifluoromethylphenylcarbamoyl)diethylenetriamine

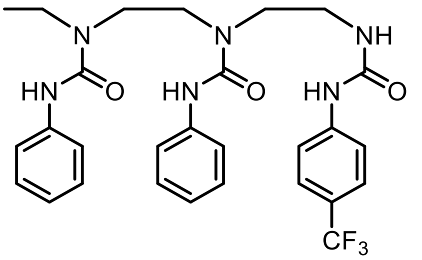

To a solution of *N*-ethyl-*N,N''-bis*(phenylcarbamoyl)diethylenetriamine **3ac** (20 mg, 0.054 mmol) in CH_2Cl_2 (1 mL) was added 4-trifluoromethylphenyl isocyanate (0.01 mL, 0.07 mmol, 1.3 equiv.) and the mixture was stirred at 20 °C for 16 hours. The product crushed out of solution (25 mg, 0.0448 mmol, 83%). **^{1}H NMR** (400 MHz, $CD_2Cl_2+CH_3OH$) δ 8.70 (s, 1H, N*H*), 8.28 (s, 1H, N*H*), 7.67 (s, 1H, N*H*), 7.48 (dd, 2H, J = 8.6, 1.2 Hz, 2 x CH_{Ar}), 7.42 – 7.35 (m, 6H, 6 x CH_{Ar}), 7.22 – 7.06 (m, 4H, 4 x CH_{Ar}), 7.04 – 6.83 (m, 2H, 2 x CH_{Ar}), 6.19 – 6.09 (t, 1H, J = 4.8 Hz, N*H*CH_2), 3.48 – 3.39 (m, 6H, 3 x CH_2N), 3.33 (q, J = 7.1 Hz, 2H, CH_2CH_3), 3.26 (q, J = 6.4, 4.8 Hz, 2H, CH_2NH), 1.13 (t, J = 7.1 Hz, 3H, CH_3CH_2). **^{13}C NMR** (101 MHz, $CD_2Cl_2+CH_3OH$) δ 157.6 (C=O $_{\text{urea Ph}}$), 157.1 (C=O $_{\text{urea PhCF3}}$), 156.7 (C=O $_{\text{urea CH3CH2NCO}}$), 143.7 (C_{Ar}), 143.6 (C_{Ar}), 140.3 (C_{Ar}), 140.0 (C_{Ar}), 129.2 (2C, 2 x CH_{Ar}), 129.2 (2C, 2 x CH_{Ar}), 126.6 (q, J = 3.7 Hz), 123.65 (q, J = 33.2 Hz, CCF_3), 121.8 (q, J = 271.4 Hz, CF_3), 123.5 (CH_{Ar}), 123.4 (CH_{Ar}), 120.9 (2C, 2 x CH_{Ar}), 120.8 (2C, 2 x CH_{Ar}), 118.5 (2C, 2 x CH_{Ar}), 118.4 (2C, 2 x CH_{Ar}), 48.3 (CH_2N), 47.8 (CH_2N), 46.8 (CH_2N), 43.7 (CH_2CH_3), 40.1 (CH_2NH), 14.3 (CH_3). **FTIR (neat)** vmax = 3300, 3113, 2936, 1642, 1599, 1548, 1319, 1247, 1111, 1066 cm^{-1}. **HR – MS** (ESI, positive ion mode) – *m/z* for $[C_{28}H_{31}F_3N_6O_3+Na]^+$ 579.2302, observed 579.2286. **MP** 184-185 °C.

3b *N*-Ethyl-*N,N''-bis*(phenylcarbamoyl)-*N*'-(3,5-*bis*(trifluoromethyl)phenylcarbamoyl)diethylenetriamine

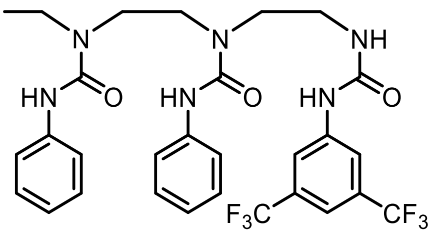

Following general procedure E, *N*-ethyl-*N,N''-bis*(phenylcarbamoyl)-*N*'-(tertbutoxycarbonyl)diethylenetriamine **3ab** (43 mg, 0.09 mmol) afforded *N*-ethyl-*N,N''-bis*(phenylcarbamoyl)diethylenetriamine **3ac** (27 mg, 0.072 mmol, 80%) that was used without purification to the next step. Following general procedure G, **3ac** (8.5 mg, 0.023 mmol) afforded the title product **3b** (14 mg, quantitative yield) as a white solid. **^{1}H NMR** (400 MHz, DMSO – d_6) δ 9.44 (s, 1H, NH_{Ar3}), 8.86 (s, 1H,

N*H*), 8.60 (s, 1H, N*H*), 8.11 (d, *J* = 1.6 Hz, 2H, 2 x CH_{Ar3}), 7.65 – 7.46 (m, 5H, 2 x CH_{Ar1}, 2 x CH_{Ar2}, CH_{Ar3}), 7.30 – 7.13 (m, 4H, 2 x CH_{Ar1}, 2 x CH_{Ar2}), 6.93 (m, 2H, CH_{Ar1}, CH_{Ar2}), 6.69 (s, 1H, N*H*CH_2), 3.57 – 3.35 (m, 8H, 4 x CH_2), 3.31 (m, 2H, CH_2NH), 1.09 (t, *J* = 7.0 Hz, 3H, CH_3). **^{13}C NMR** (101 MHz, DMSO – d_6) δ 155.5 (C=O), 155.4 (C=O), 155.1 (C=O $_{Ar3}$), 142.4 (C_{Ar3}), 140.4 (2C, C_{Ar1}, C_{Ar2}), 130.6 (2C, *J* = 32.5 Hz, 2 x *C*CF_3), 128.2 (4C, 2 x *C*$H_{Ar1,}$ 2 x *C*H_{Ar2}), 124.7 (2C, *J* = 274.15 Hz, 2 x *C*F_3), 121.8 (2C, *C*$H_{Ar1,}$ *C*H_{Ar2}), 119.8 (2C, 2 x *C*H_{Ar2}), 119.3 (2C, 2 x *C*H_{Ar1}), 117.4 (2C, 2 x *C*H_{Ar3}), 113.6 (*C*H_{Ar3}), 47.1 – 45.4 (4C, 4 x *C*H_2N), 42.0 (*C*H_2CH_3), 14.0 (*C*H_3). **FTIR (neat)** vmax = 3301, 2933, 1646, 1552, 1385, 1277, 1130 cm^{-1}. **HR – MS** (ESI, positive ion mode) – *m/z* for $[C_{29}H_{30}F_6N_6O_3+H]^+$ 625.2356, observed 625.2346. **MP** 218-219 °C.

Compound 5ab: *N,N*''-*bis*(hexylcarbamoyl)-*N*-benzyl-*N*'-(tertbutoxycarbonyl)triethylenetetramine

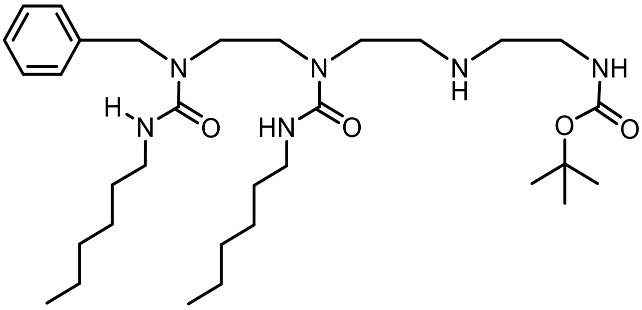

Following general procedure C with *N,N''-bis*(hexylcarbamoyl)-*N*-benzyldiethylenetriamine **1ac** (336 mg, 0.75 mmol), *N*-Boc-2-aminoacetaldehyde (191 mg, 1.2 mmol) and $NaBH_4$ (89 mg, 2.4 mmol), the title compound **5ab** was obtained (110 mg, 0.22 mmol, 29 %) as a colourless oil. **^{1}H NMR** (400 MHz, $CDCl_3$) δ 7.30 (m, 2H, 2 x CH_{Ph}), 7.24 (d, *J* = 7.0 Hz, 1H, CH_{Ph}), 7.23 – 7.16 (m, 2H, 2 x CH_{Ph}), 6.46 (t, *J* = 5.4 Hz, 1H, N*H*Boc), 5.54 (s, 1H, N*H*hexyl), 5.07 (s, 1H, N*H*hexyl), 4.46 (s, 2H, C*H*$_2$Ph), 3.32 (m, 2H, C*H*$_2$NBn), 3.24 – 3.08 (m, 8H, 4 x C*H*$_2$), 2.69 (m, 4H, 2 x C*H*$_2$N), 2.46 (bs, 1H, N*H*), 1.53 – 1.42 (m, 4H, 2 x CH_{2hexyl}), 1.41 (s, 9H, (C*H*$_3$)$_3$C), 1.34 – 1.11 (m, 12H, 6 x CH_{2hexyl}), 0.95 – 0.66 (m, 6H, 2 x C$H_{3\ hexyl}$). **^{13}C NMR** (101 MHz, $CDCl_3$) δ 159.6 (C=O_{Boc}), 158.7 (C=O_{urea}), 156.3 (C=O_{urea}), 138.3 (C_{Ph}), 128.8 (2C, 2 x *C*H_{Ph}), 127.5 (*C*H_{Ph}), 127.2 (2C, 2 x *C*H_{Ph}), 79.4 (*C*(CH_3)$_3$), 51.3 (*C*H_2Ph), 49.5 (*C*H_2N), 48.7 (*C*H_2N), 46.9 (2C, 2 x CH_2N), 46.2 (*C*H_2NBn), 40.9 (2C, 2 x *C*$H_2NH_{\ hexyl}$), 39.9 (*C*H_2NHBoc), 31.6 (2C, 2 x *C*H_2), 30.1 (2C, 2 x *C*H_2), 28.5 (3C, (*C*H_3)$_3$C), 26.6 (2C, 2 x *C*H_2), 22.7 (2C, 2 x *C*H_2), 14.1 (2C, 2 x *C*$H_{3\ hexyl}$). **FTIR (neat)** vmax = 3292, 2955, 2927, 2857, 1638, 1625, 1535, 1364, 1252, 1171 cm^{-1}. **HR – MS** (ESI, positive ion mode) – *m/z* for $[C_{32}H_{58}N_6O_4+H]^+$ 591.4592, observed 591.4581.

Compound 5ac: *N,N*'',*N'''*-tri(hexylcarbamoyl)-*N*-benzyl-*N*'-(*tert*butoxycarbonyl)triethylenetetramine

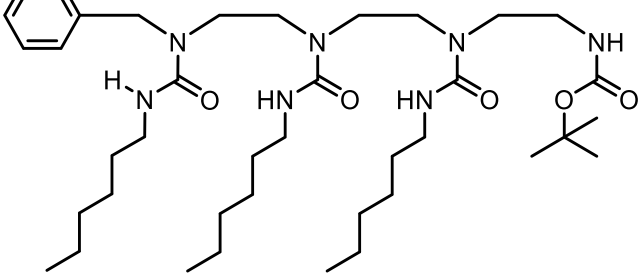

Following general procedure D starting from *N*,*N*''-*bis*(hexylcarbamoyl)-*N*-benzyl-*N*'-(*tert*butoxycarbonyl)triethylenetetramine **5ab** (100 mg, 0.17 mmol) and hexyl isocyanate (40 µL, 0.25 mmol), the title compound **5ac** was obtained as a colourless oil after silica gel flash chromatography (CH_2Cl_2:MeOH 97:3) (94 mg, 0.13 mmol, 78 %). **^{1}H NMR** (400 MHz, $CDCl_3$) δ 7.26 (d, *J* = 7.3 Hz, 2H, 2 x CH_{Ph}), 7.21 (d, *J* = 6.5 Hz, 1H, CH_{Ph}), 7.17 – 7.12 (m, 2H, 2 x CH_{Ph}), 6.46 (t, *J* = 5.2 Hz, 1H, N*H*), 6.19 (s, 1H, NH_{hexyl}), 5.14 (m, 1H, NH_{hexyl}), 5.45 – 4.80 (bs, 1H, NH_{hexyl}), 4.38 (s, 2H, CH_2Ph), 3.28 (m, 2H, CH_2), 3.24 – 2.99 (m, 16H, 8 x CH_2), 1.53 – 1.41 (m, 4H, 2 x C$H_{2\ hexyl}$), 1.40 – 1.34 (2 H, C$H_{2\ hexyl}$), 1.35 (s, 9 H, , (CH_3)$_3$C), 1.31 – 1.19 (m, 12 H, 6 x C$H_{2\ hexyl}$), 1.19 – 1.08 (m, 6H, 3 x C$H_{2\ hexyl}$), 0.89 – 0.70 (m, 9H, 3 x C$H_{3\ hexyl}$). **^{13}C NMR** (101 MHz, $CDCl_3$) δ 158.8 (C=O), 158.7 (C=O), 158.7 (C=O), 156.8 (C=O), 137.8 (C_{Ph}), 129.0 (2C, 2 x CH_{Ph}), 127.7 (CH_{Ph}), 126.9 (2C, 2 x CH_{Ph}), 79.8 (*C*(CH_3)$_3$), 52.0 (CH_2Ph), 47.6 – 47.2 (5C, 5 x CH_2N), 41.1 – 41.0 (3 x $CH_{2\ hexyl}$), 40.0 (CH_2N), 31.7 – 31.5 (3 x $CH_{2\ hexyl}$), 30.0 – 29.9 (3 x $CH_{2\ hexyl}$), 28.4 (3C, (CH_3)$_3$C), 26.8 – 26.6 (3 x $CH_{2\ hexyl}$), 22.6 (3C, 3 x $CH_{2\ hexyl}$), 14.1 (3C, 3 x $CH_{3\ hexyl}$). **FTIR (neat)** vmax = 3295, 2955, 2928, 2858, 1690, 1628, 1642, 1355, 1266, 1262 cm^{-1}. **HR – MS** (ESI, positive ion mode) – *m/z* for $[C_{21}H_{32}N_7O_5+H]^+$ 740.5409, observed 740.5554.

Compound 5bc: *N*'',*N*'''-*bis*(hexylcarbamoyl)-*N*-benzyl-*N*-(tertbutylcarbamoyl)-*N*'-(tertbutoxycarbonyl)triethylenetetramine

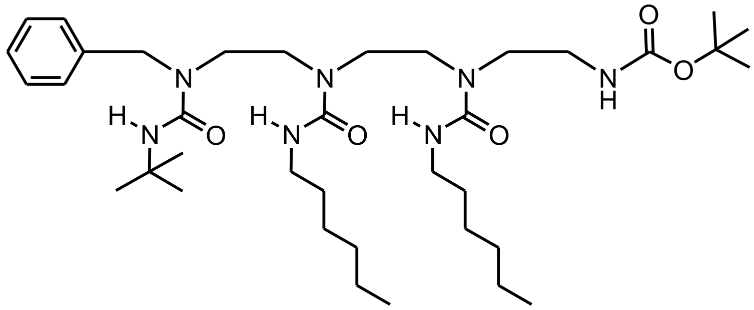

Following general procedure C using *N*-benzyl-*N*-(*tert*butylcarbamoyl)-*N*''-(hexylcarbamoyl) triethylenetetramine (336 mg, 0.75 mmol), *N*-Boc-2-aminoacetaldehyde (191 mg, 1.2 mmol) and $NaBH_4$ (89 mg, 2.4 mmol), *N*''-(hexylcarbamoyl)-*N*-benzyl-*N*-(tertbutylcarbamoyl)-*N*'-(tertbutoxycarbonyl)triethylenetetramine **5bb** was obtained (173 mg, 0.29 mmol, 39 %) and was used without extensive purification in procedure D (hexyl isocyanate 68 µL, 0.45 mmol) to give title compound **5bc** as a colourless oil (135 mg, 0.194 mmol, 66 %). **^{1}H NMR** (400 MHz, $CDCl_3$) δ 7.37 – 7.31 (m, 2H, 2 x CH_{Ar}), 7.30 – 7.24 (m, 1H, CH_{Ar}), 7.20 (d, *J* = 7.7 Hz, 2H, 2 x CH_{Ar}), 6.72 (t, 1H, N*H*C$H_{2\ hexyl}$), 6.32 (t, 1H, N*H*C$H_{2\ hexyl}$), 5.28 (m, 1H, N*H*Boc), 4.62 (s, 1H, N*H*tBu), 4.39 (s, 2H, CH_2Ph), 3.38 – 3.31 (m , 2H, CH_2 N), 3.30 – 3.10 (m, 14H, 7 x CH_2), 1.61 – 1.48 (m, 4H, 2 x C$H_{2\ hexyl}$), 1.41 (s, 9H, 3 x C$H_{3\ Boc}$), 1.38 – 1.25 (m, 12 H, 6 x C$H_{2\ hexyl}$), 1.24 (s, 9H, 3 x C$H_{3\ tBu\ urea}$), 0.96 – 0.76 (m, 6H, 2 x C$H_{3\ Hexyl}$). **^{13}C NMR** (101 MHz, $CDCl_3$) δ 158.8 (C=O), 158.8 (C=O), 157.9 (C=$O_{tBu\ urea}$), 156.8 (C=O_{Boc}), 137.7 (C_{Ar}), 129.0 (2C, 2 x CH_{Ar}), 127.7 (CH_{Ar}), 126.7 (2C, 2 x CH_{Ar}), 79.6 (*C*(CH_3)$_{3\ Boc}$), 52.4 (CH_2Ph), 51.0 (*C*(CH_3)$_3$), 47.7– 47.0 (5 x C, CH_2N), 41.1 (2C, 2 x CH_2NH), 40.1 (CH_2NH), 31.7 (CH_2), 31.7 (CH_2), 29.9 (CH_2), 29.8 (CH_2), 29.3 (3C, 3 x CH_3C), 28.4 (3C, 3 x $CH_{3\ Boc}$), 26.8 (CH_2), 26.7 (CH_2), 22.6 (2C,

2 x CH_2), 14.1 (2C, 2 x CH_3). **FTIR (neat)** vmax = 3304, 2928, 2863, 1687, 1628, 1532, 1364, 1299, 1169 cm^{-1}. **HR – MS** (ESI, positive ion mode) – *m/z* for $[C_{37}H_{67}N_7O_5+H]^+$ 690.5276, observed 690.5270.

Compound 5ad: *N*-benzyl-*N*,*N*'',*N*'''-tri(hexylcarbamoyl)triethylenetetramine

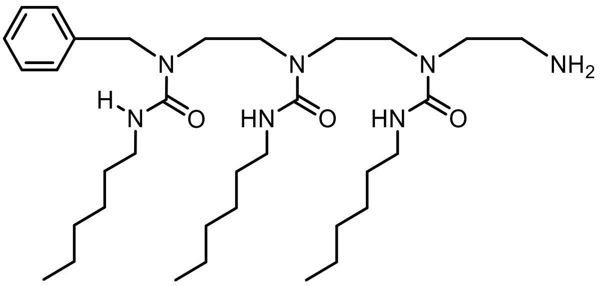

Following general procedure E with *N*,*N*'',*N*'''-tri(hexylcarbamoyl)-*N*-benzyl-*N*'-(*tert*butoxycarbonyl)triethylenetetramine **5ac** (100 mg, 0.13 mmol), the title compound **5ad** was obtained (70 mg, 0.104 mmol, 80 %) as an oil. **^{1}H NMR** (400 MHz, $CDCl_3$) δ 7.26 (t, *J* = 7.4 Hz, 2H, 2 x CH_{Ph}), 7.19 (s, 1H, CH_{Ph}), 7.15 (d, *J* = 7.6 Hz, 2H, 2 x CH_{Ph}), 6.61 (t, *J* = 5.4 Hz, 1H, N*H*), 6.52 (t, *J* = 5.3 Hz, 1H, N*H*), 5.35 (s, 1H, N*H*), 4.40 (s, 2H, C*H*$_2$Ph), 3.27 (m, 2H, C*H*$_2$), 3.21 – 3.02 (m, 14 H, 7 x C*H*$_2$), 2.76 (m, 2H, C*H*$_2$NH$_2$), 1.93 (s, 2H, N*H*$_2$), 1.53 – 1.32 (m, 6H, 3 x C*H*$_{2\ hexyl}$), 1.32 – 1.10 (m, 18H, 9 x C*H*$_{2\ hexyl}$), 0.80 (m, 9H, 3 x C*H*$_{3\ hexyl}$). **^{13}C NMR** (101 MHz, $CDCl_3$) δ 160.1 (C=O$_{urea}$), 158.8 (C=O$_{urea}$), 158.7 (C=O$_{urea}$), 138.2 (*C*$_{Ph}$), 128.9 (2 C, 2 x *C*H$_{Ph}$), 127.5 (*C*H$_{Ph}$), 127.1 (2 C, 2 x *C*H$_{Ph}$), 51.9 (*C*H$_2$), 51.8 (*C*H$_2$Ph), 47.2 (*C*H$_2$), 47.2 (*C*H$_2$), 47.1 (*C*H$_2$), 46.9 (*C*H$_2$), 41.4 (*C*H$_2$NH$_2$), 41.1 – 40.9 (3 x *C*H$_{2\ hexyl}$), 31.6 (3C, 3 x *C*H$_{2\ hexyl}$), 30.1 (3C, 3 x *C*H$_{2\ hexyl}$), 26.8 (2C, 2 x *C*H$_{2\ hexyl}$), 26.6 (*C*H$_{2\ hexyl}$), 22.7 (3C, 3 x *C*H$_2$), 14.2 (*C*H$_{3\ hexyl}$), 14.1 (2C, 2 x *C*H$_{3\ hexyl}$). **FTIR (neat)** vmax = 3297, 2918, 2850, 1628, 1544, 1215 cm^{-1}. **HR – MS** (ESI, positive ion mode) – *m/z* for $[C_{34}H_{63}N_7O_3+H]^+$ 618.5065, observed 618.5060.

Compound 5bd: *N*-benzyl-*N*-(*tert*butylcarbamoyl)-*N*'',N'''-*bis*(hexylcarbamoyl)triethylenetetramine

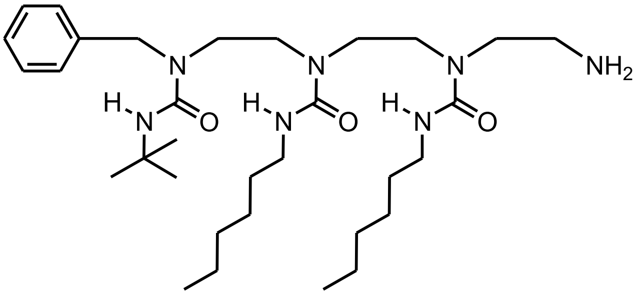

Following general procedure E using *N*'',*N*'''-*bis*(hexylcarbamoyl)-*N*-benzyl-*N*-(*tert*butylcarbamoyl)-*N*'-(*tert*butoxycarbonyl)triethylenetetramine **5bc** (130 mg, 0.19 mmol), the title compound **5bd** was isolated as a colourless oil (115 mg, 0.19 mmol, quantitative yield). **^{1}H NMR** (400 MHz, $CDCl_3$) δ 7.30 (m, 2H, 2 x CH_{Ar}), 7.23 (t, 1H, CH_{Ar}), 7.18 (d, *J* = 7.2 Hz, 2H, 2 x CH_{Ar}), 6.71 (t, 1H, N*H*$_{hexyl}$), 6.37 (t, *J* = 5.4 Hz, 1H, N*H*$_{hexyl}$), 4.80 (bs, 1H, N*Ht*Bu), 4.39 (s, 2H, C*H*$_2$Ph), 3.39 – 3.03 (m, 14H, *CH*$_2$N), 2.80 (m, 2H, *CH*$_2$NH$_2$), 2.27 (bs, 2H, N*H*$_2$), 1.29 – 1.17 (m, 16H, 8 x C*H*$_2$), 1.24 (s, 9H, 3 x C*H*$_3$, *t*Bu), 0.88 – 0.80 (m, 6H, 2 x C*H*$_3$). **^{13}C NMR** (101 MHz, $CDCl_3$) δ 159.9 (*C*=O), 158.7 (*C*=O),

157.8 (C=O)NHtBu), 138.0 (C_{Ar}), 128.9 (2C, 2 x CH_{Ar}), 127.6 (CH_{Ar}), 126.9 (2C, 2 x CH_{Ar}), 52.1 (CH_2Ph), 50.9 ($C(CH_3)_3$), 47.4 – 47.0 (4C, 4 x CH_2N), 41.3 (CH_2N), 41.1 (CH_2), 40.1 (CH_2), 31.7 (CH_2), 31.6 (CH_2), 30.0 (CH_2), 29.9 (CH_2), 29.3 (3C, 3 x CH_3C), 28.4 (CH_2NH_2), 26.8 (CH_2), 26.8 (CH_2), 22.6 (2C, 2 x CH_2), 14.1 (CH_3), 14.1 (CH_3). **FTIR (neat)** vmax = 3298, 2956, 2927, 2857, 1631, 1538, 1268 cm^{-1}. **HR – MS** (ESI, positive ion mode) – *m/z* for $[C_{32}H_{59}N_7O_3+H]^+$ 590.4752, observed 590.4755.

Compound 5a: *N*-benzyl-*N*,*N*'',N'''-tri(hexylcarbamoyl)-*N*'-(3,5-*bis*(trifluoromethyl)phenylcarbamoyl)triethylenetetramine

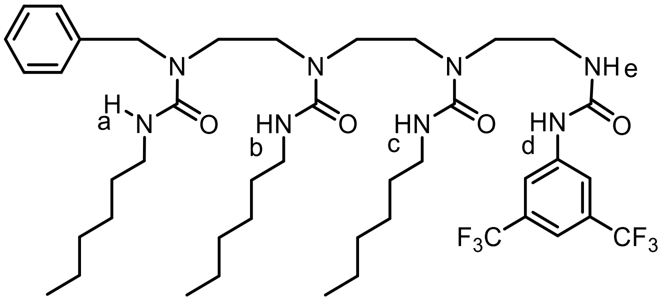

Following general procedure G, *N*-benzyl-*N*,*N*'',N'''-tri(hexylcarbamoyl)triethylenetetramine **5ad** (13 mg, 0.02 mmol) gave the title compound **5a** (18 mg, quantitative yield) as a white solid. **^{1}H NMR** (400 MHz, CD_2Cl_2) δ 9.06 (s, 1H, NH_d), 7.97 (s, 2H, CH_{Ar}), 7.42 (s, 1H, CH_{Ar}), 7.35 (t, *J* = 7.4 Hz, 2H, 2 x CH_{Ph}), 7.27 (t, *J* = 7.3 Hz, 1H, CH_{Ph}), 7.22 (d, *J* = 7.5 Hz, 2H, 2 x CH_{Ph}), 6.84 (t, *J* = 5.3 Hz, 1H, NHb), 6.64 (s, 1H, N*Hc*), 6.39 (s, 1H, N*He*), 4.92 (bs, 1 H, N*H*a), 4.44 (s, 2H, CH_2Ph), 3.41 (m, 2H, CH_2), 3.37 – 3.04 (m, 16H, 8 x CH_2), 1.60 – 1.45 (m, 4H, 2 x CH_2), 1.44 – 1.11 (m, 20H, 10 x CH_2), 0.97 – 0.67 (m, 9H, 3 x CH_{3hexyl}). **^{13}C NMR** (101 MHz, CD_2Cl_2) δ159.5 (2C, 2 x C=O), 159.4 (C=O), 156.5 (C=O_{Ar} urea), 142.7 (C_{Ar}), 138.0 (C_{Ph}), 132.2 (q, *J* = 33.0 Hz, 2C, 2 x $C_{Ar}CF_3$), 131.7 (2C, 2 x CH_{Ph}), 129.5 (CH_{Ph}), 128.3 (2C, 2 x CH_{Ph}), 124.1 (q, *J* = 272.5 Hz, 2C, 2 x CF_3), 118.2 (CH_{Ar}), 115.0 (CH_{Ar}), 52.8 (CH_2Ph), 49.0 – 47.8 (5C, 5 x CH_2N), 41.8 – 41.5 (3C, 3 x CH_{2Hexyl}), 40.0 (CH_2N), 32.2 – 32.1 (3C, 3 x CH_{2Hexyl}), 30.5 – 30.4 (3C, 3 x CH_{2Hexyl}), 27.3 – 27.0 (3C, 3 x CH_{2Hexyl}), 23.2 – 23.1 (3C, 3 x CH_{2Hexyl}), 14.4 – 14.3 (3C, 3 x CH_{3Hexyl}). **FTIR (neat)** vmax = 3301, 2955, 2928, 1623, 1542, 1385, 1275, 1177, 1132 cm^{-1}. **HR – MS** (ESI, positive ion mode) – *m/z* for $[C_{43}H_{67}F_6N_8O_4+H]^+$ 873.5184, observed 873.5200 ; *m/z* for $[C_{43}H_{66}F_6N_8O_4+Na]^+$ 895.5001, observed 895.5009. **MP** 50-51 °C.

Compound 5b: *N*-benzyl-*N*-(*tert*butylcarbamoyl)-*N*'',*N*'''-*bis*(hexylcarbamoyl)-*N*'-(3,5-*bis*(trifluoromethyl)phenylcarbamoyl)triethylenetetramine

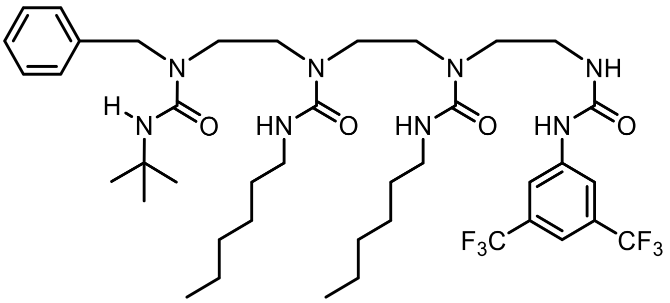

Following general procedure G, *N*-benzyl-*N*-(tertbutylcarbamoyl)-*N*'',*N*'''-*bis*(hexylcarbamoyl)triethylenetetramine **5bd** (28 mg, 0.047 mmol) afforded the title compound **5b** (15 mg, 0.0183 mmol, 39 %) as a solid. **^{1}H NMR** (400 MHz, $CDCl_3$) δ 8.88 (s, 1H, N*H*Ar), 7.96 (s, 2H, 2 x CH_{Ar}), 7.43 (s, 1H, CH_{Ar}), 7.39 (dd, *J* = 8.1, 6.4 Hz, 2H, 2 x CH_{Ph}), 7.35 – 7.29 (m, 1H, CH_{Ph}), 7.25 – 7.15 (m, 2H, 2 x CH_{Ph}), 7.10 (s, 1H, NH_{hexyl}), 6.93 (s, 1H, N*H*), 6.19 (s, 1H, NH_{hexyl}), 4.39 (s, 2H, CH_2Ph), 4.32 (s, 1H, N*H*), 3.38 (m, 12H, 6 x CH_2), 3.31 – 3.25 (m, 2H, C$H_2$$N_{hexyl}$), 3.25 – 3.17 (m, 2H, C$H_2$$N_{hexyl}$), 1.62 (m, 2H, C$H_{2\ hexyl}$), 1.54 (dd, *J* = 7.3 Hz, 2H, C$H_{2\ hexyl}$), 1.43 – 1.26 (m, 12H, 6 x C$H_{2\ hexyl}$), 1.24 (s, 9H, (CH_3)$_3$C), 0.96 – 0.76 (m, 6H, 2 x C$H_{3\ hexyl}$). **^{13}C NMR** (101 MHz, $CDCl_3$) δ 159.2 (C=O_{urea}), 158.9 (C=O_{urea}), 158.1 (C=O_{tBu}), 156.2 (C=$O_{Ar\ urea}$), 141.8 (C_{Ar}), 137.0 (C_{Ph}), 132.1 (q, *J* = 33.2 Hz, 2C, 2 x *C*CF_3), 129.3 (2C, 2 x *C*H_{Ph}), 128.1 (*C*H_{Ph}), 126.4 (2C, 2 x *C*H_{Ph}), 123.3 (q, *J* = 272.6 Hz, 2C, 2 x *C*F_3), 117.9 (2C, 2 x *C*H_{Ar}), 114.9 (*C*H_{Ar}), 53.1 (*C*H_2Ph), 51.2 (*C*(CH_3)$_3$), 48.7 – 47.4 (5 x *C*H_2N), 41.5 – 41.2 (3 x *C*$H_{2\ hexyl}$), 39.5 (*C*H_2N), 31.8 – 31.6 (3 x *C*$H_{2\ hexyl}$), 29.9 (*C*$H_{2\ hexyl}$), 29.7 (*C*$H_{2\ hexyl}$), 29.3 (*C*$H_{2\ hexyl}$), 29.3 (3C, (*C*H_3)$_3$C), 26.8 (3C, 3 x *C*$H_{2\ hexyl}$), 22.7 (3C, 3 x *C*$H_{2\ hexyl}$), 14.1 (3C, 3 x *C*$H_{3\ hexyl}$). **FTIR (neat)** vmax = 3308, 2958, 2929, 2863, 1626, 1543, 1387, 1275, 1132 cm^{-1}. **HR – MS** (ESI, positive ion mode) – *m/z* for $[C_{41}H_{62}F_6N_8O_4+H]^+$ 845.4871, observed 845.4872. **MP** 75-76 °C.

4. ^{1}H and ^{13}C NMR spectra of synthesised compounds

2aa *N*-trifluoroacetyl-*N'*-benzylethylenediamine (400 MHz, $CDCl_3$)

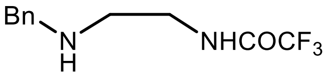

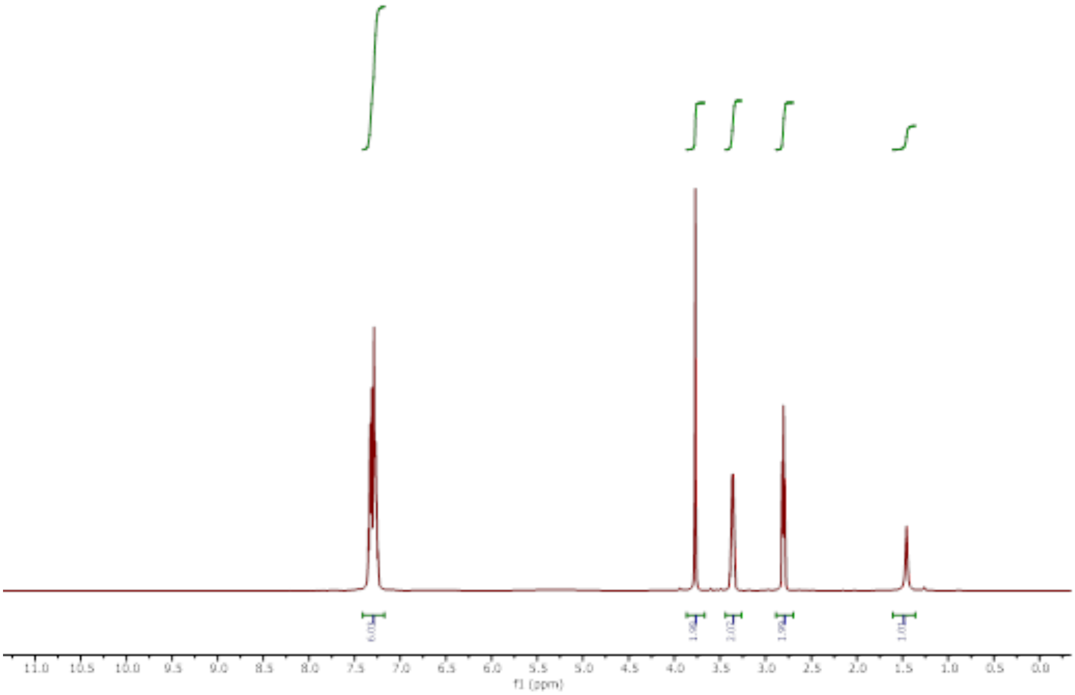

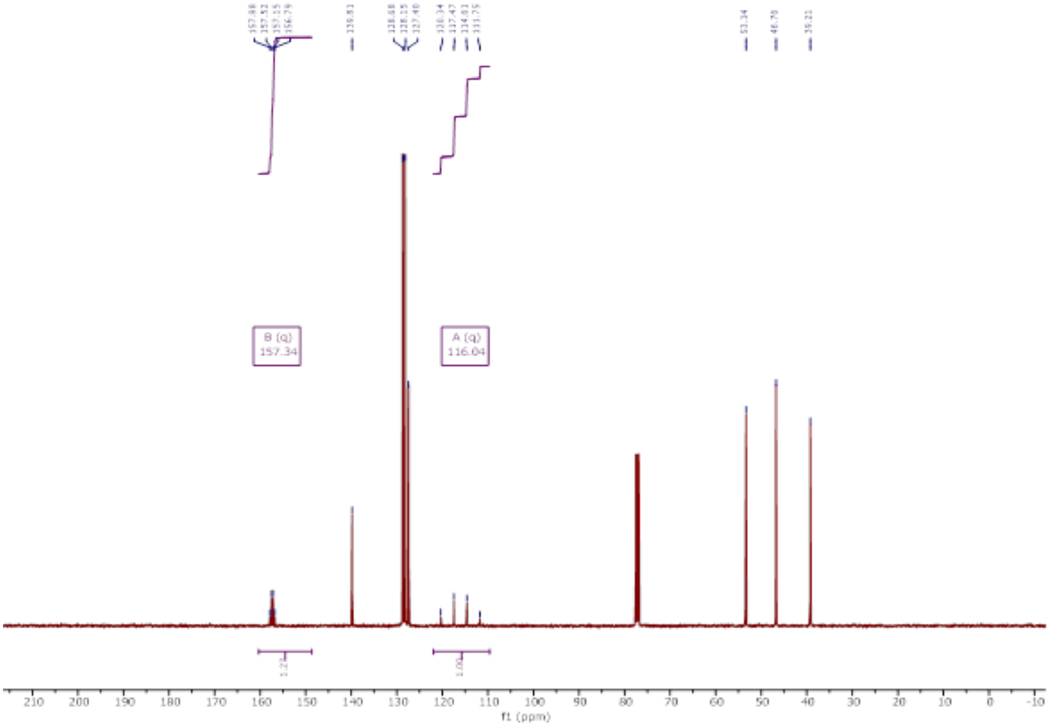

2ab *N*-Trifluoroacetyl-*N*'-(isopropylcarbamoyl)-*N*'-benzylethylenediamine (400 MHz, $CDCl_3$)

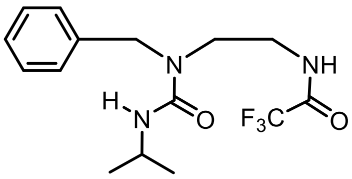

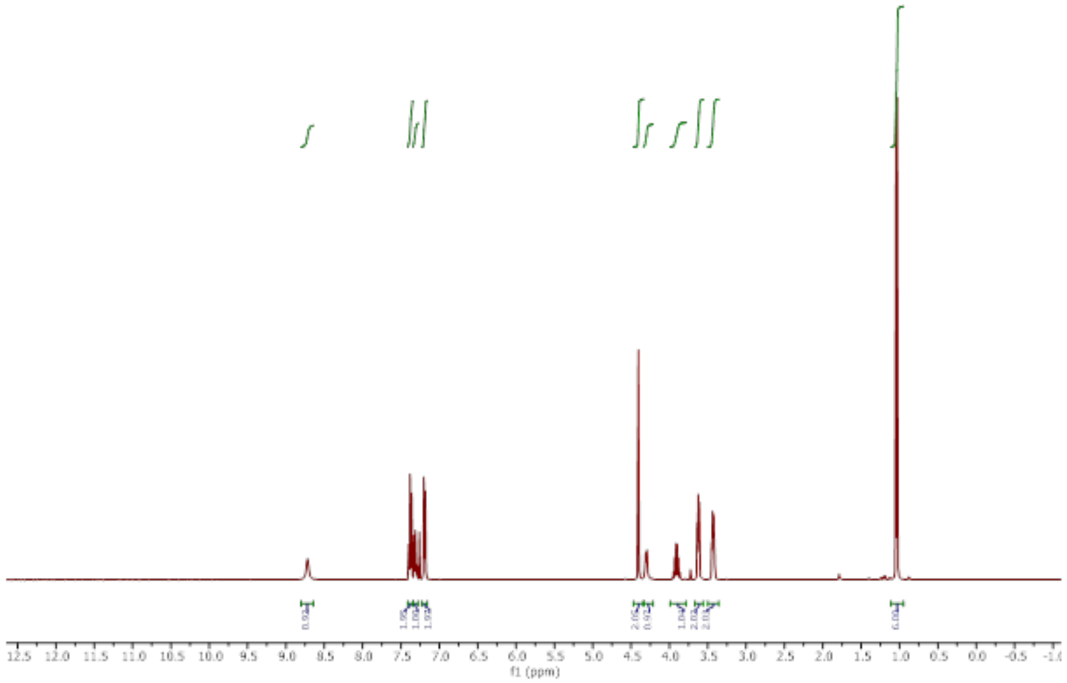

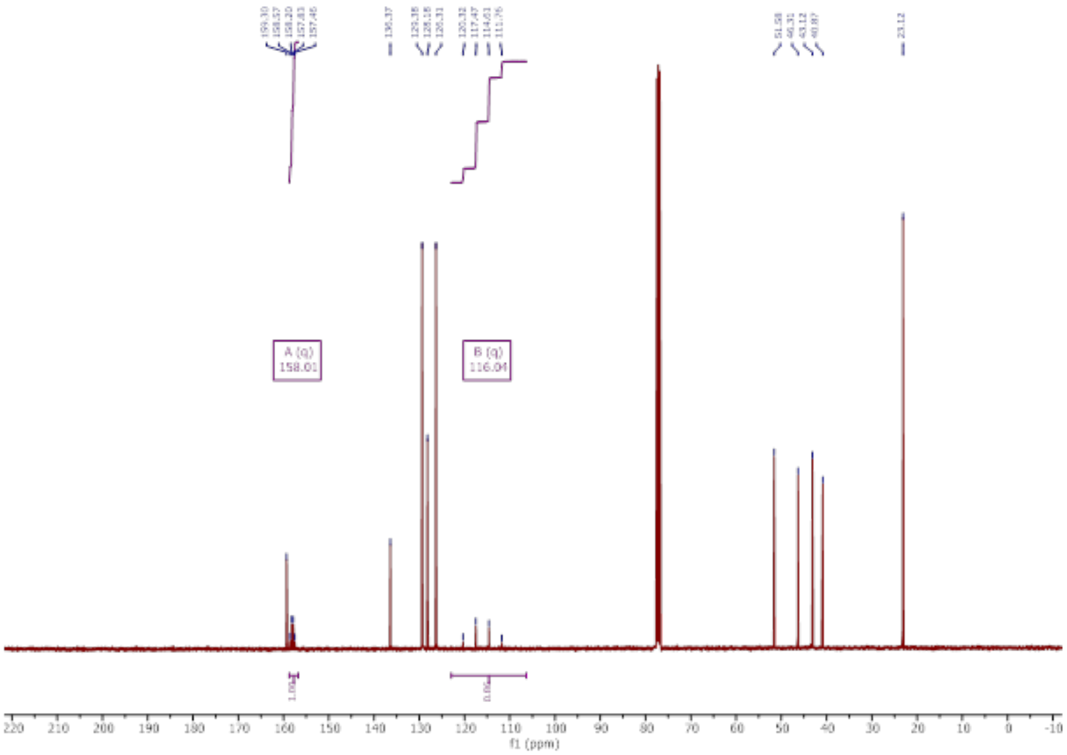

2bb *N*-Trifluoroacetyl-*N*'-(tertbutylcarbamoyl)-*N*'-benzylethylenediamine (400 MHz, $CDCl_3$)

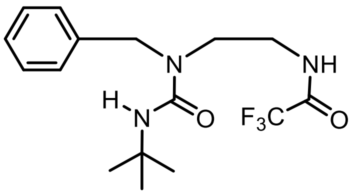

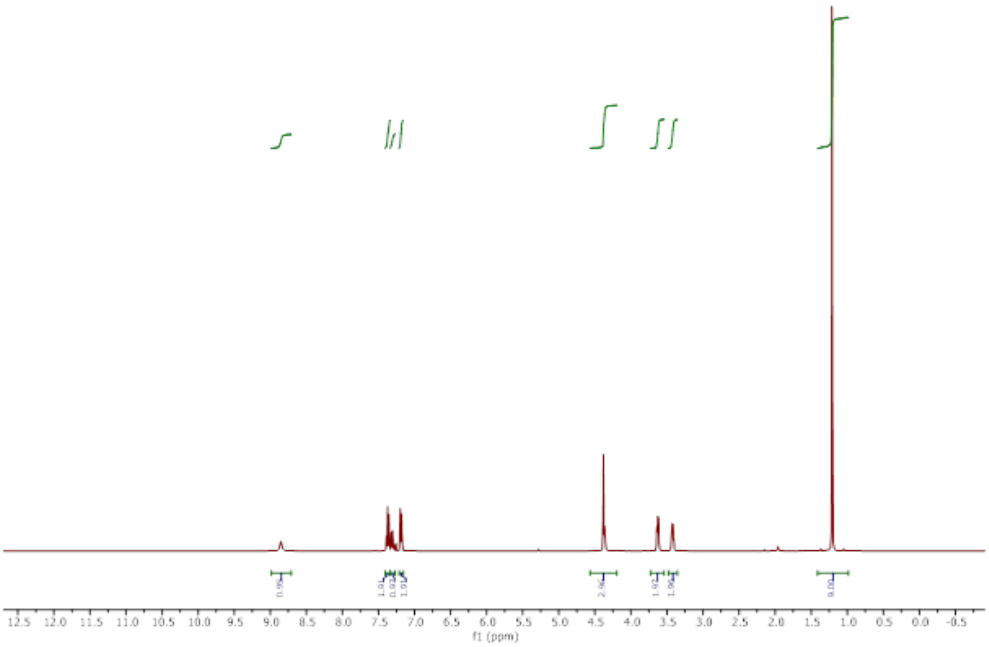

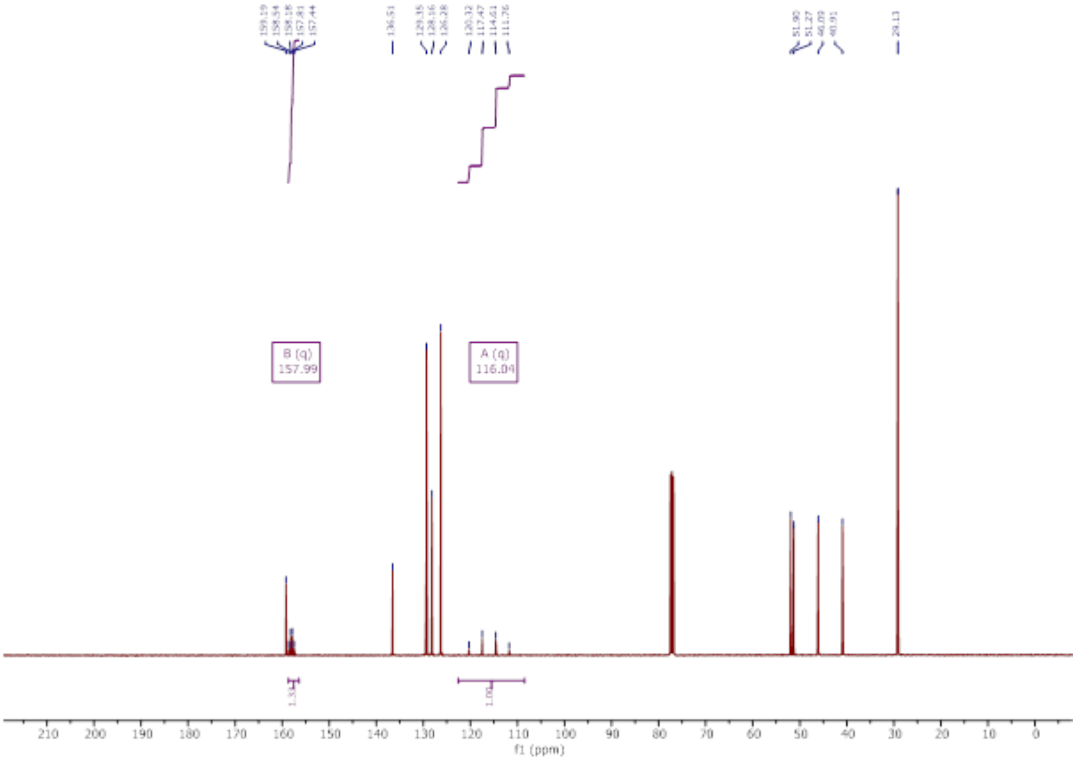

2cb *N*-trifluoroacetyl-*N*'-(phenylcarbamoyl)-*N*'-benzylethylenediamine (400 MHz, DMSO-d6)

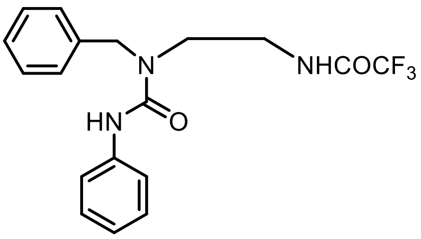

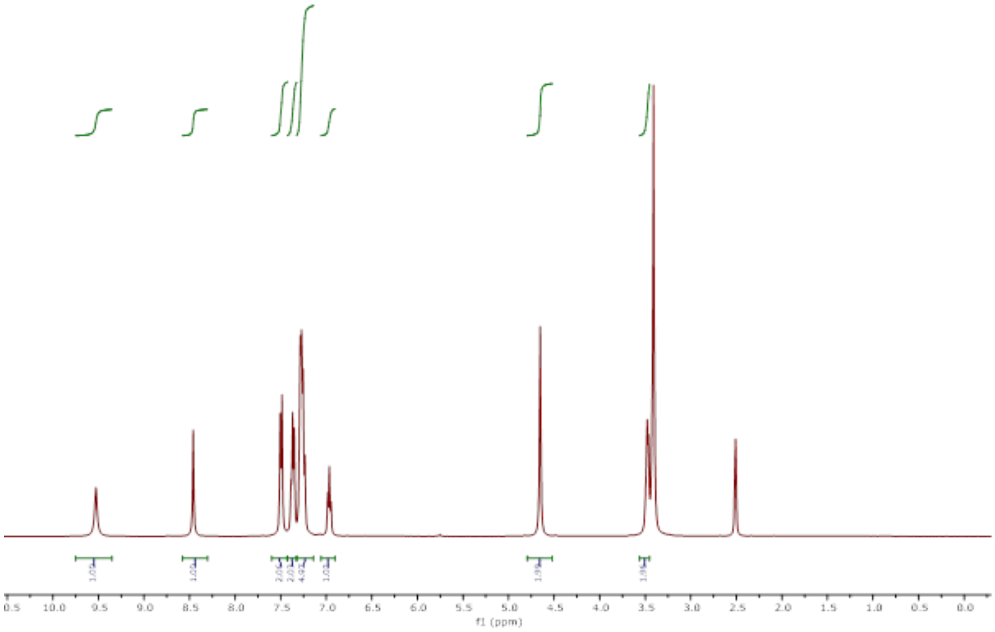

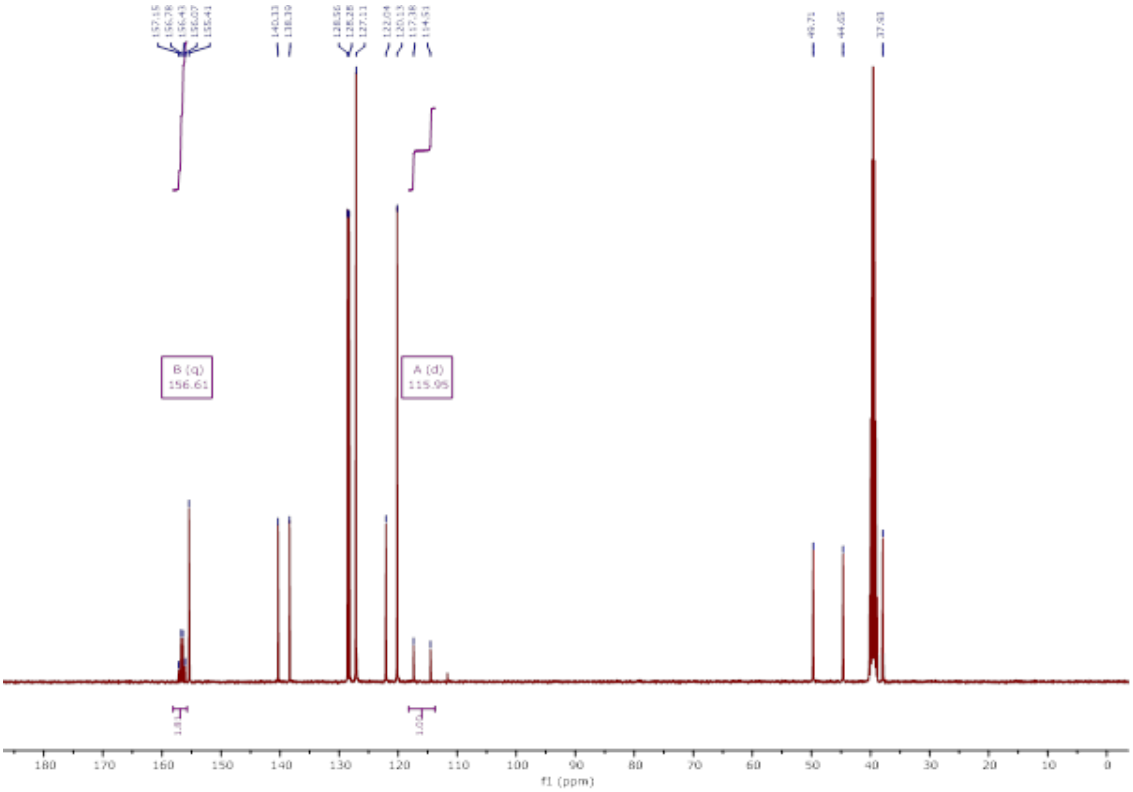

4ab *N*-trifluoroacetyl-*N*'-(isopropylcarbamoyl)-*N*'-ethylethylenediamine (400 MHz, $CDCl_3$)

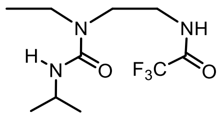

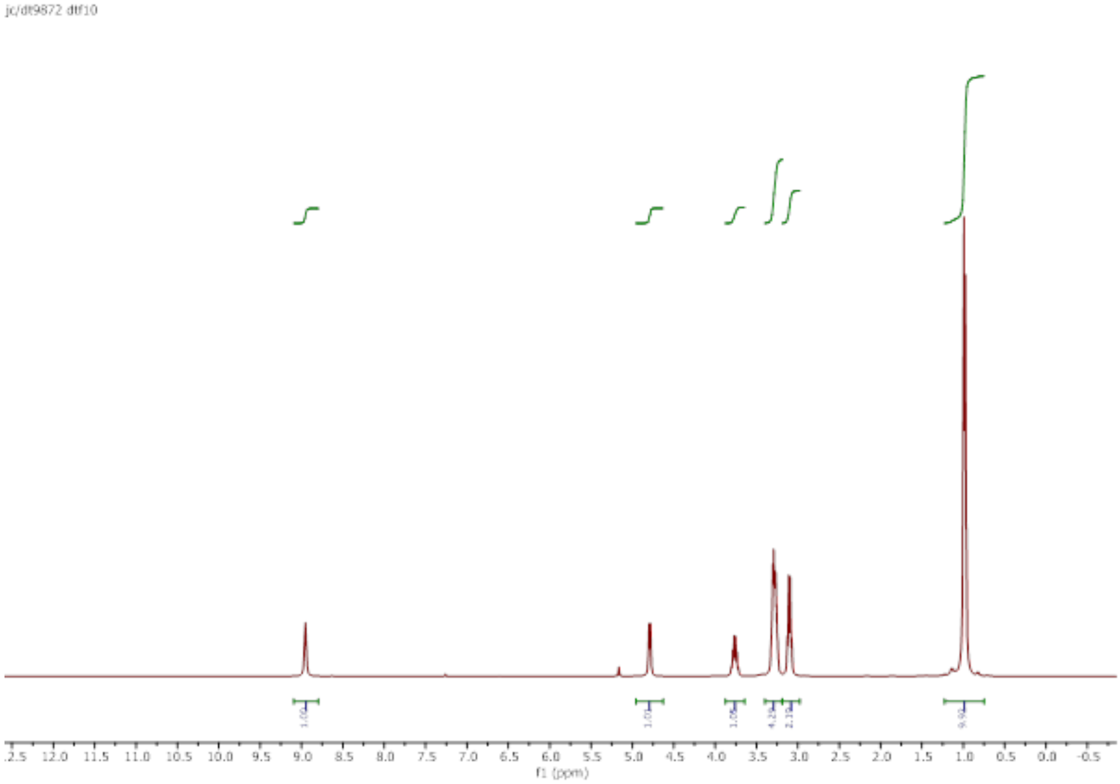

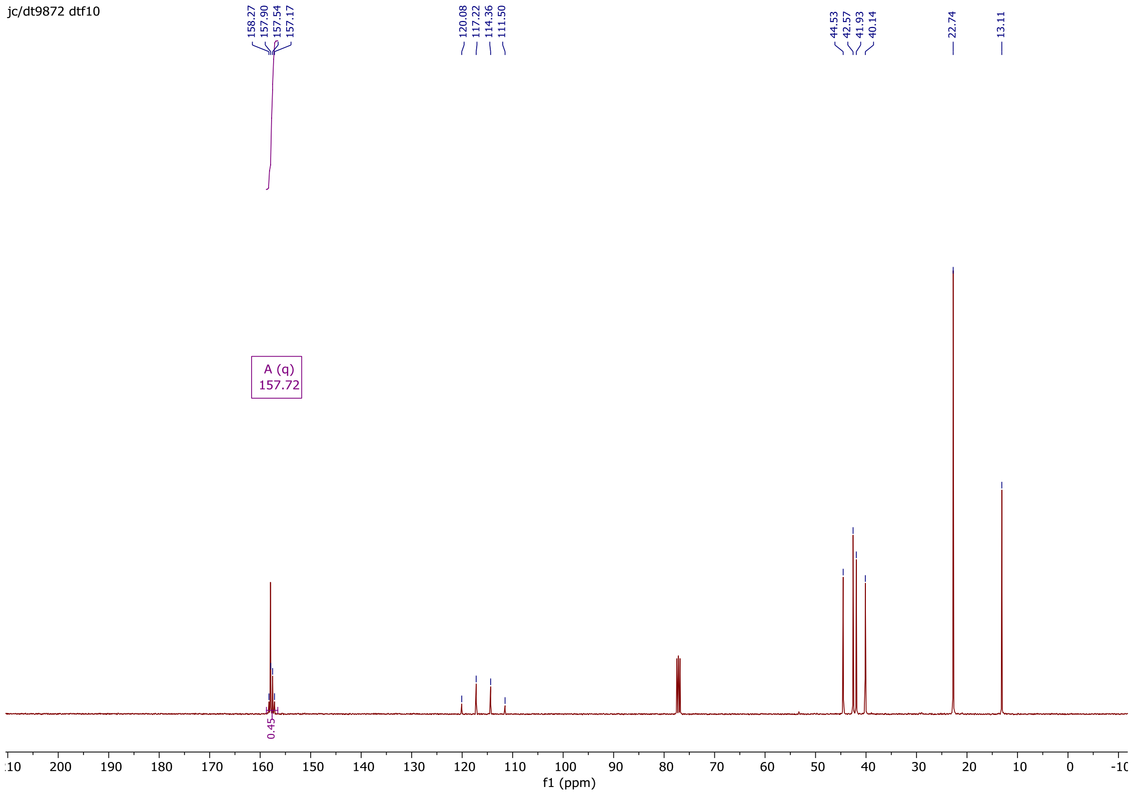

2ac *N*-(isopropylcarbamoyl)-*N*-benzylethylenediamine (400 MHz, $CDCl_3$)

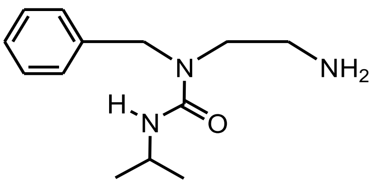

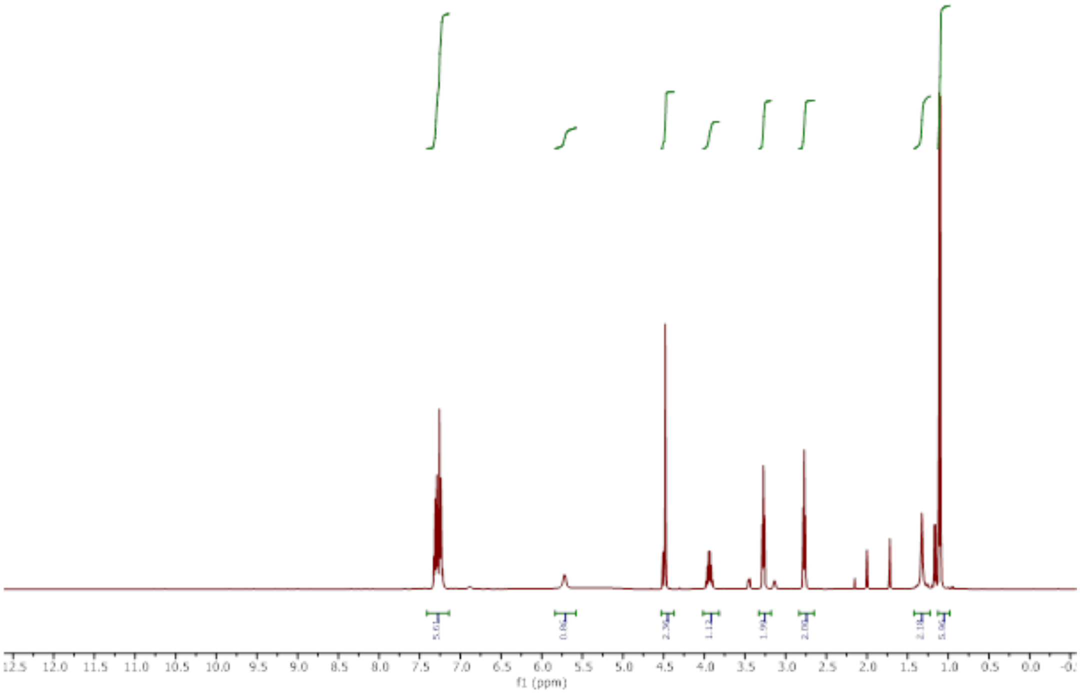

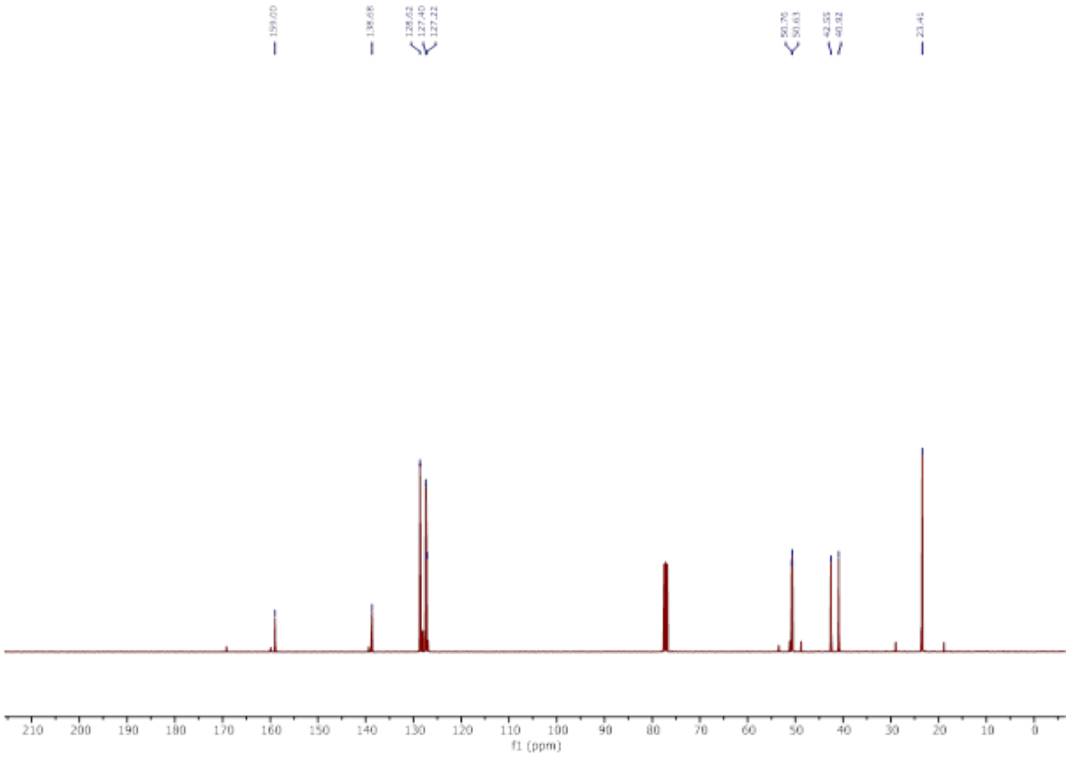

2bc *N*-(tertButylcarbamoyl)-*N*-benzylethylenediamine (400 MHz, $CDCl_3$)

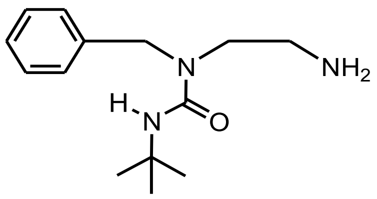

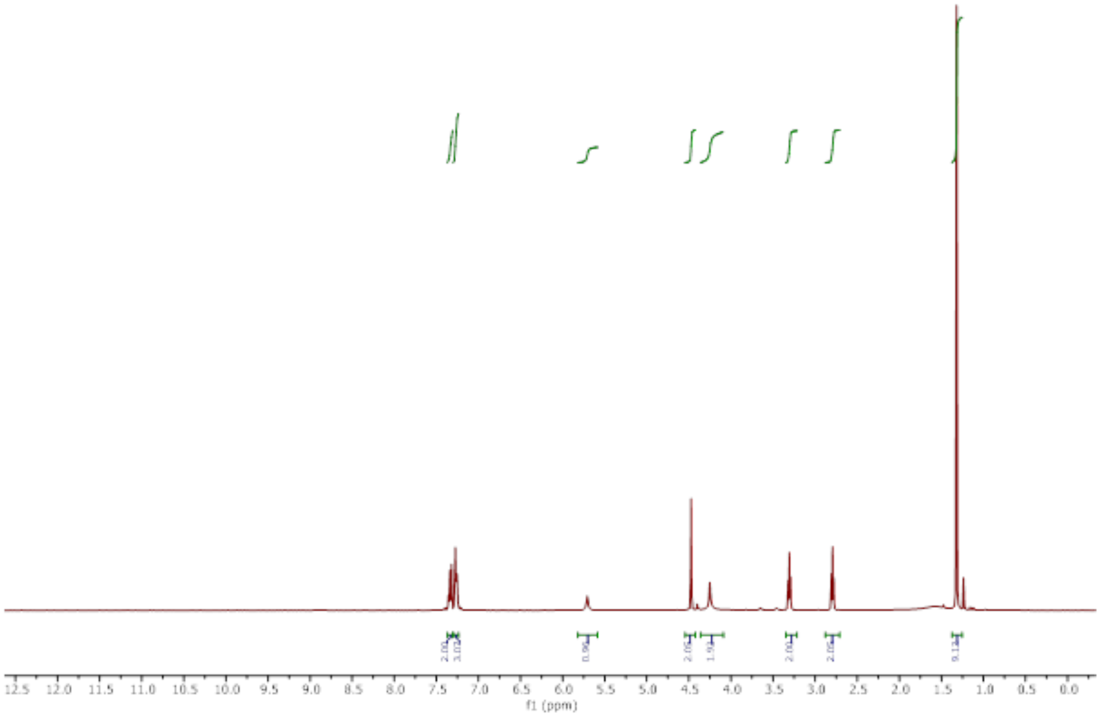

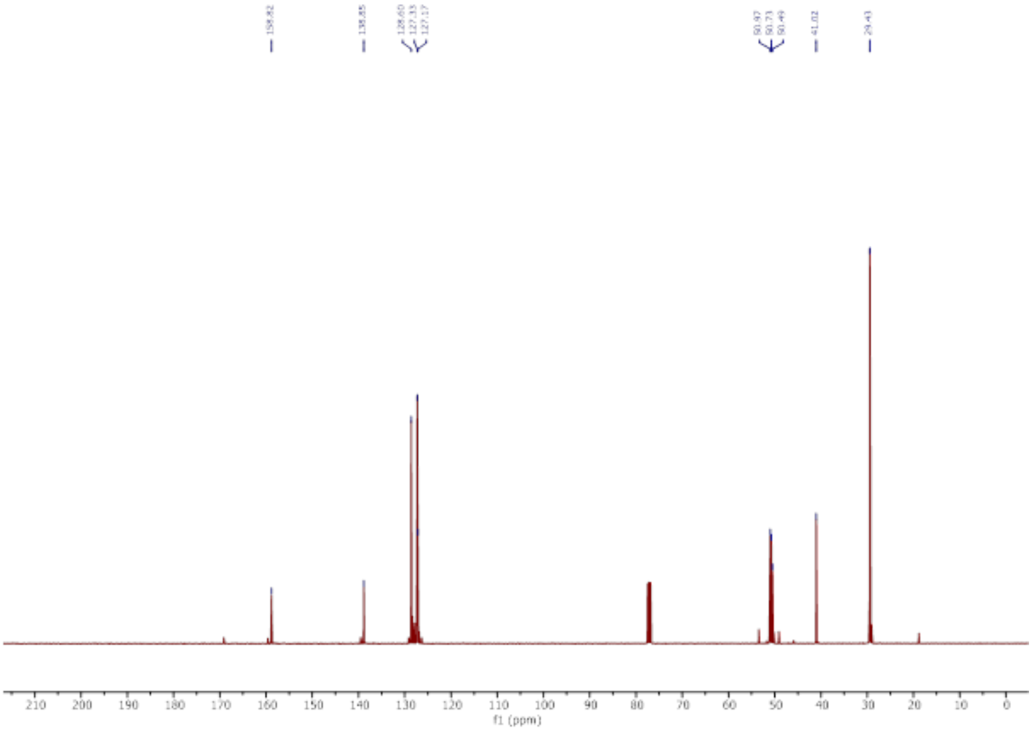

2cc *N*-(phenylcarbamoyl)-*N*-benzylethylenediamine (400 MHz, $CDCl_3$)

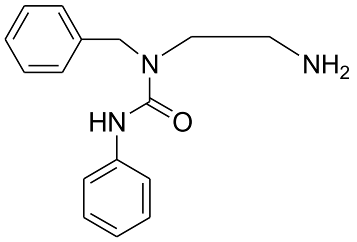

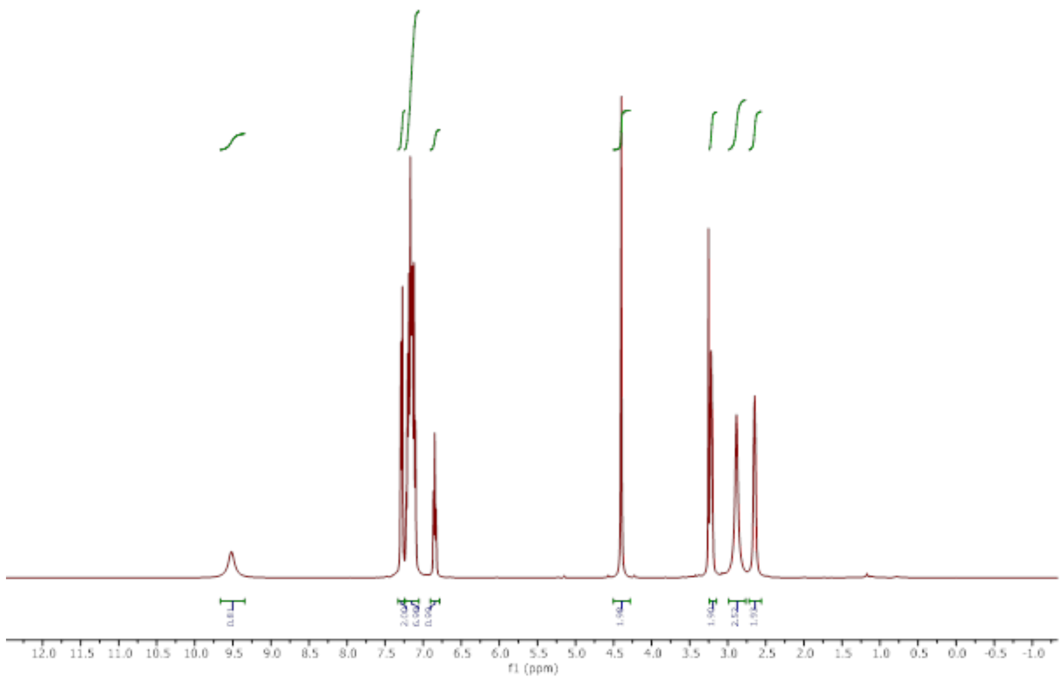

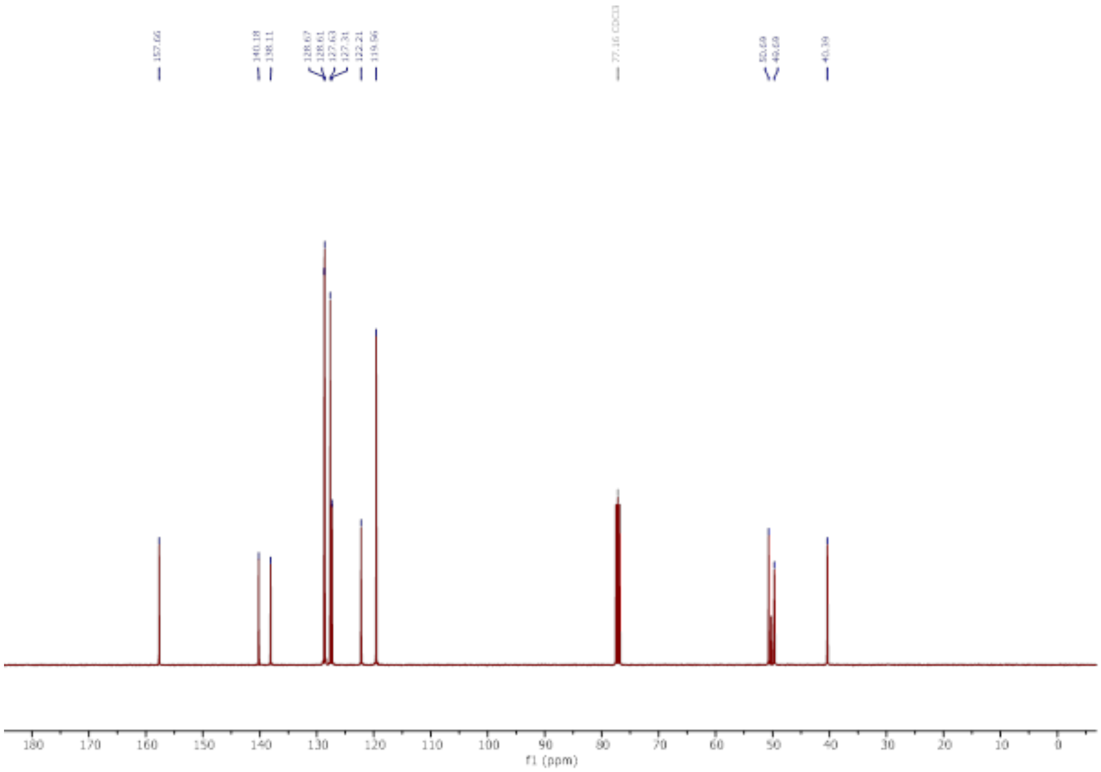

4ac *N*-(isopropylcarbamoyl)-*N*-ethylethylenediamine (400 MHz, $CDCl_3$)

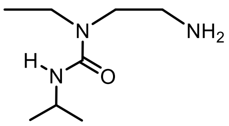

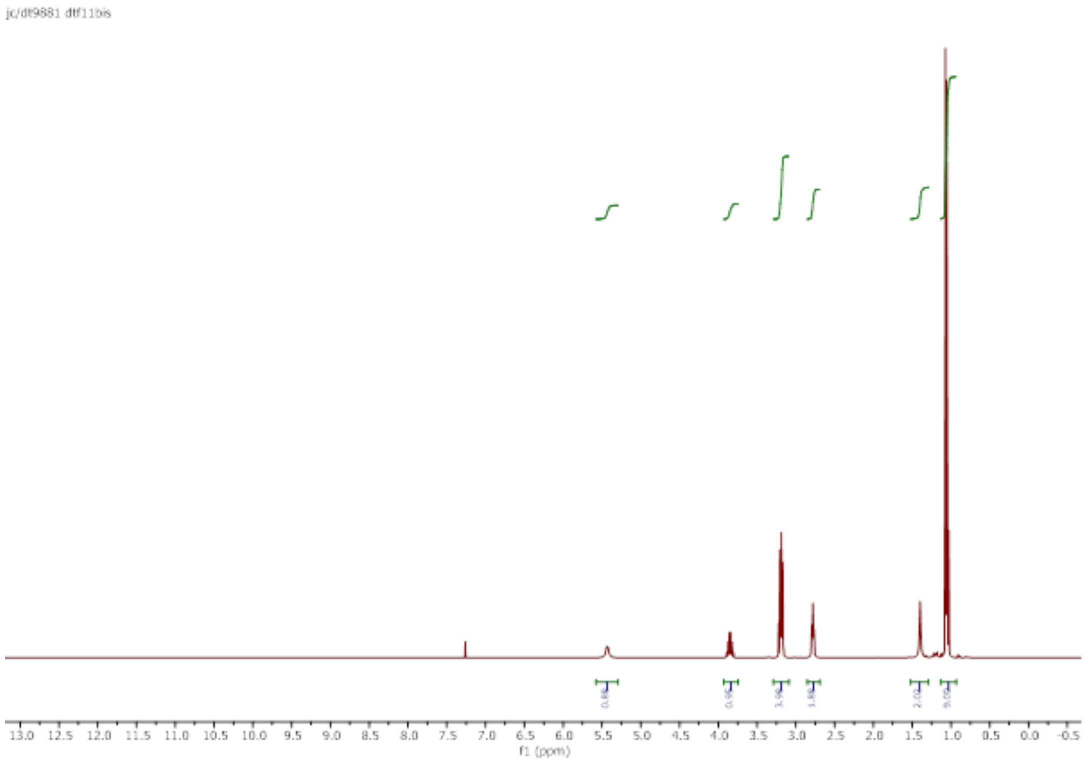

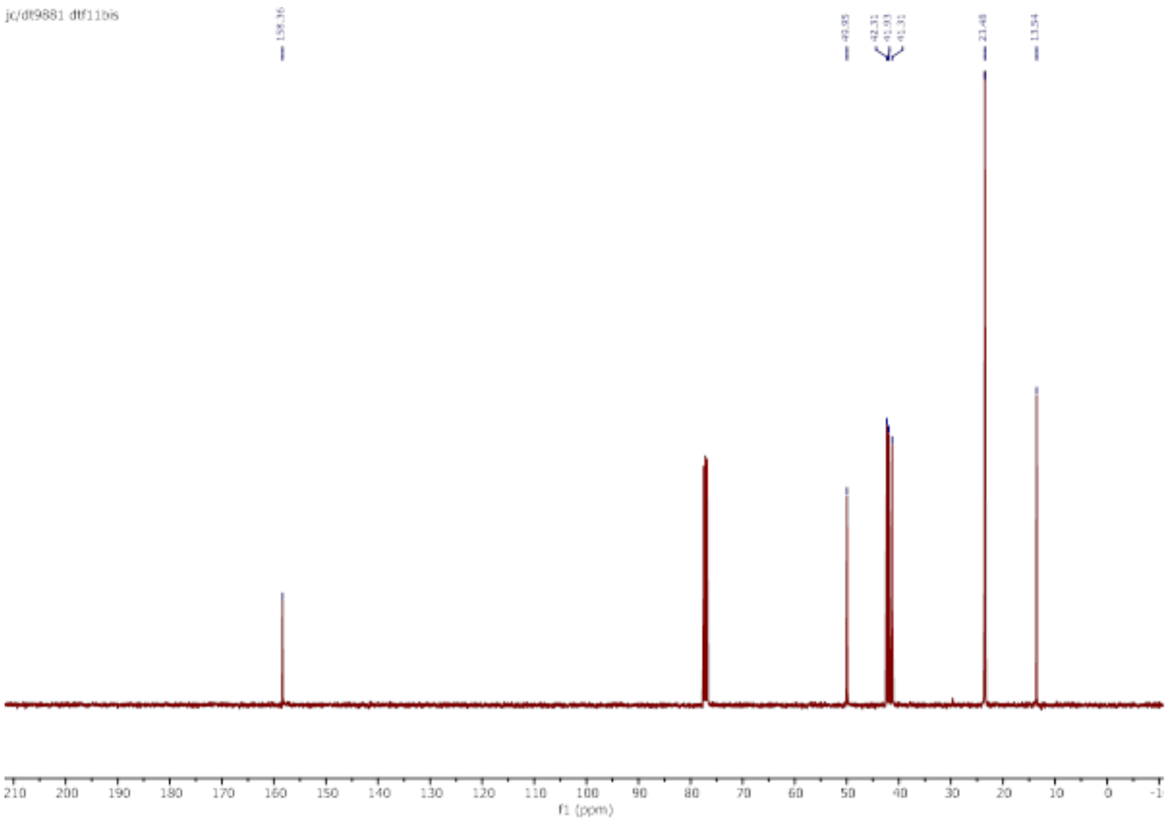

2a ***N*****-(3,5-bis(trifluoromethyl)phenylcarbamoyl)-*****N*****'-(isopropylcarbamoyl)-*****N*****'-benzylethylenediamine (400 MHz, $CDCl_3$)**

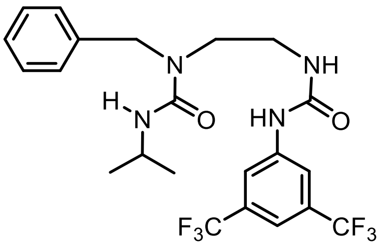

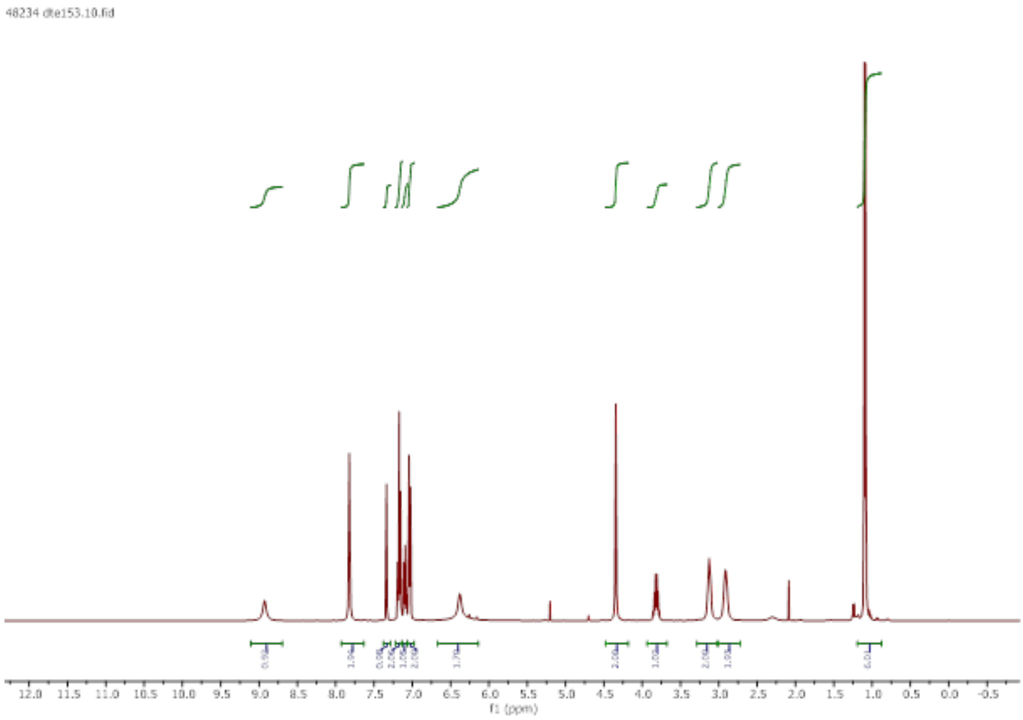

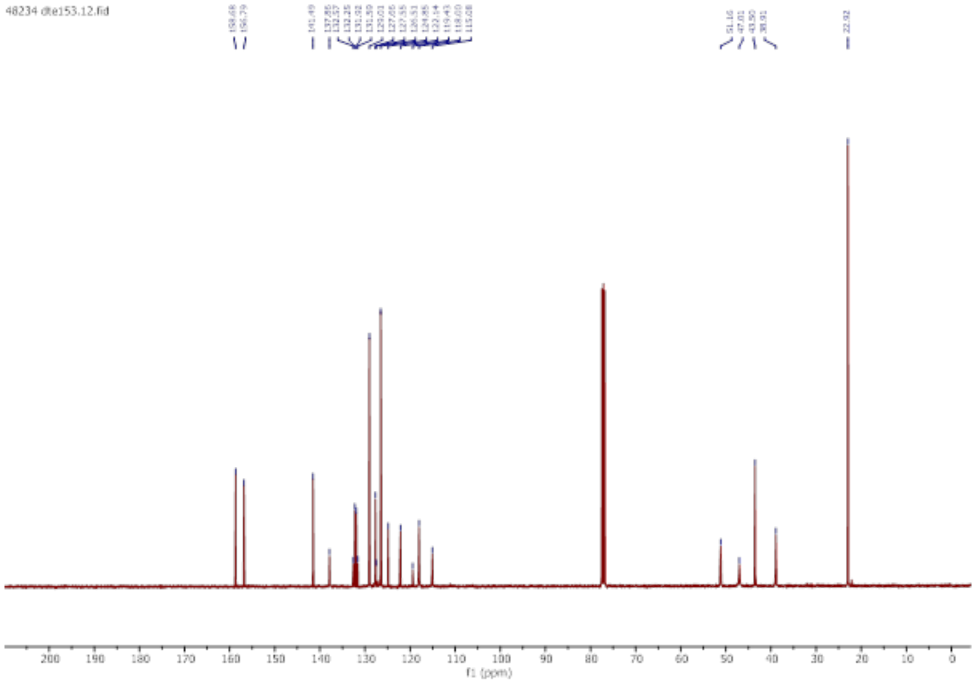

2b ***N*-(3,5-bis(trifluoromethyl)phenylcarbamoyl)-*N*'-(tertbutylcarbamoyl)-*N*'-benzylethylenediamine (400 MHz, $CDCl_3$)**

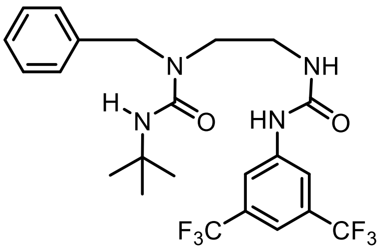

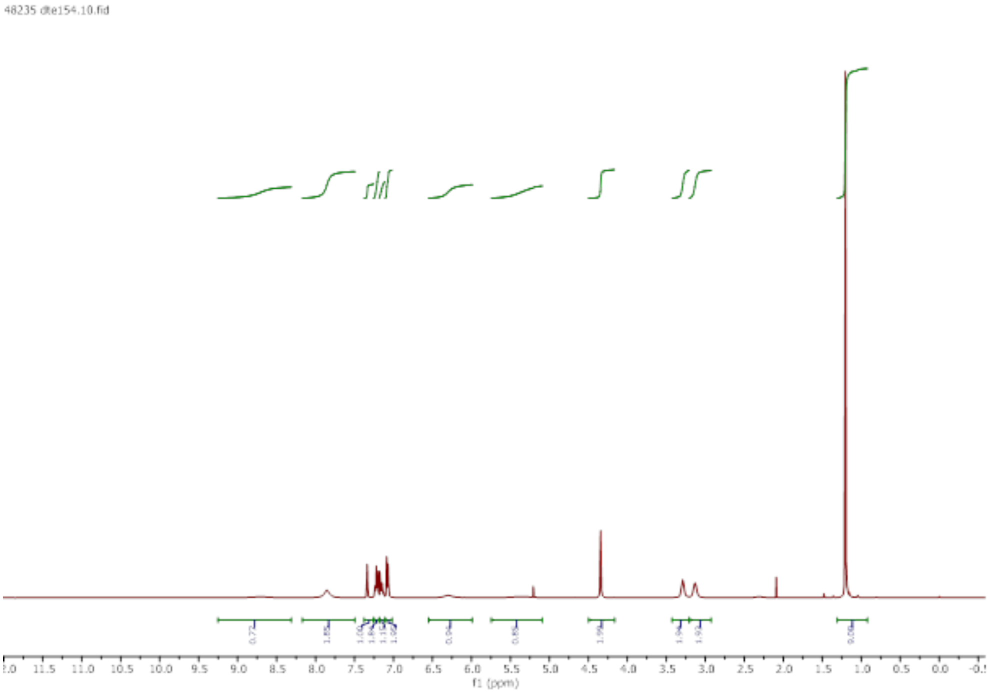

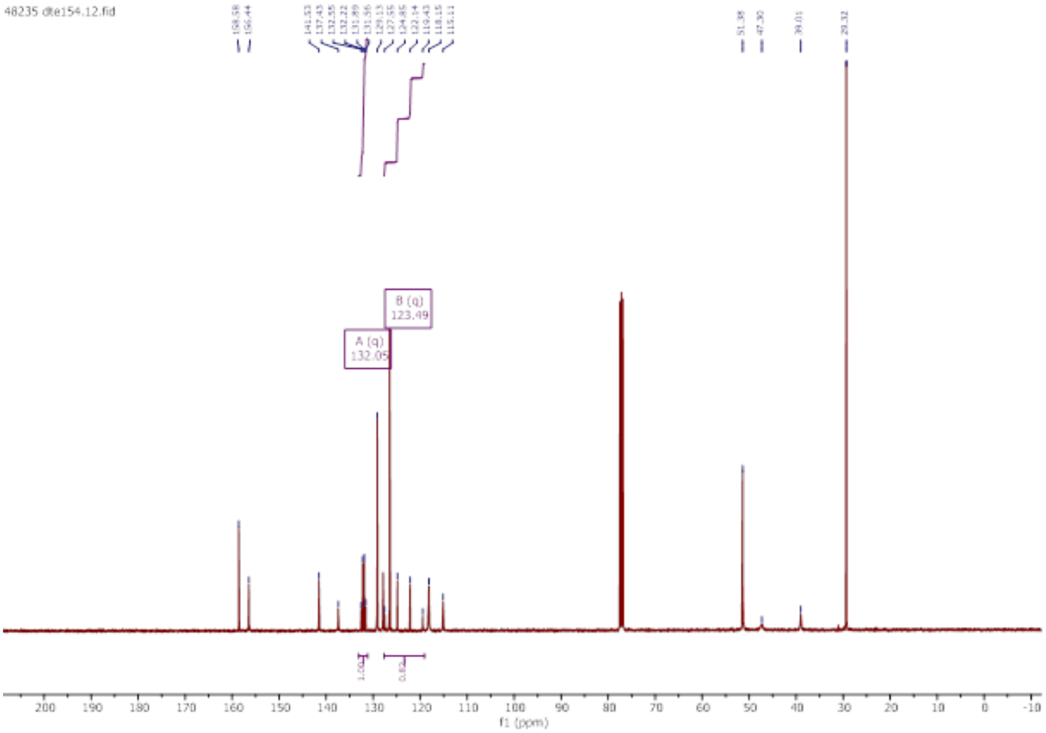

2c *N*-(phenylcarbamoyl)-*N*-benzyl-*N*'-(3,5-bis(trifluoromethyl)phenylcarbamoyl)ethylenediamine (400 MHz, DMSO d6)

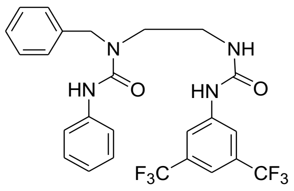

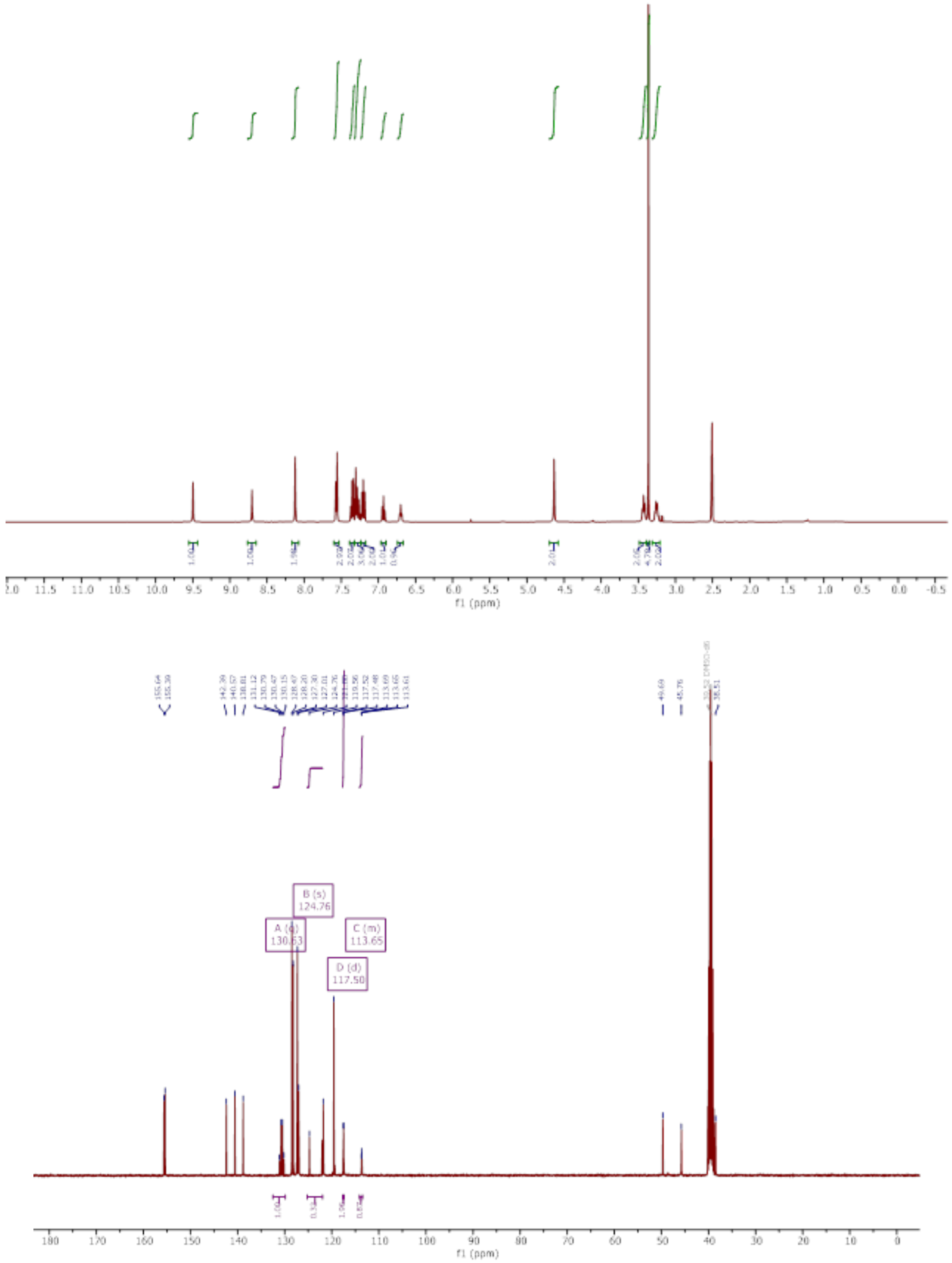

4a *N*-(3,5-bis(trifluoromethyl)phenylcarbamoyl)-*N'*-(isopropylcarbamoyl)-*N'*-ethylethylenediamine (400 MHz, $CDCl_3$)

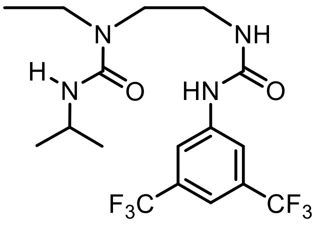

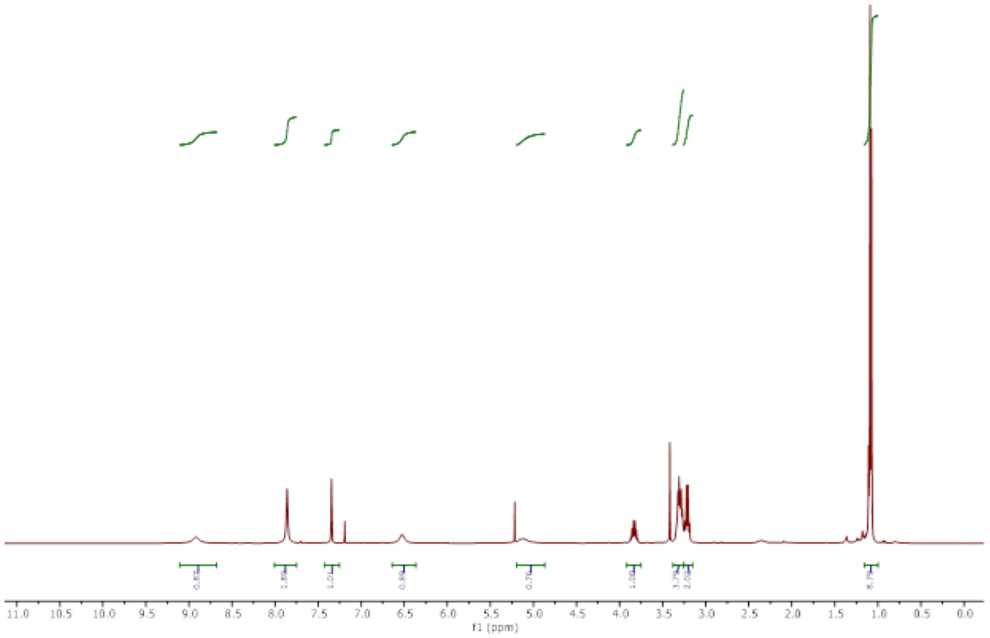

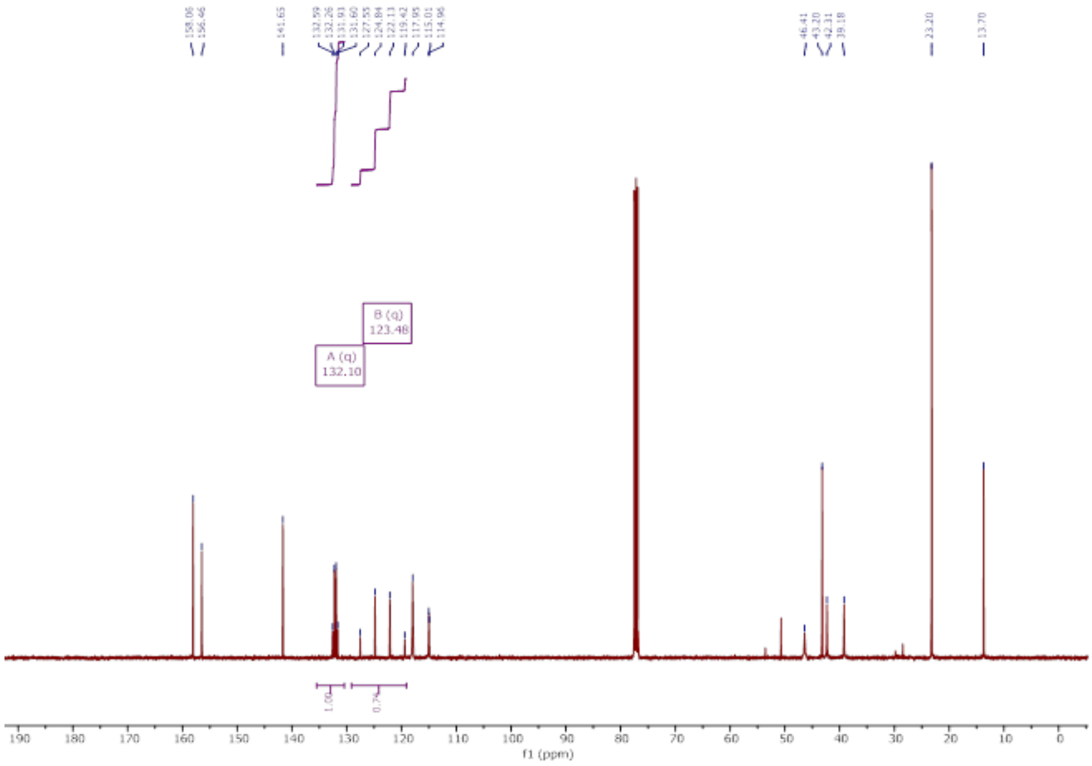

4b ***N*-(3,5-bis(trifluoromethyl)phenylcarbamoyl)-*N*'-(phenylcarbamoyl)-*N*'-ethylethylenediamine (400 MHz, DMSO d6)**

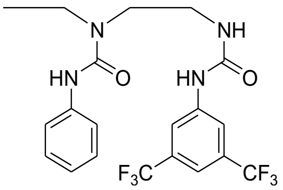

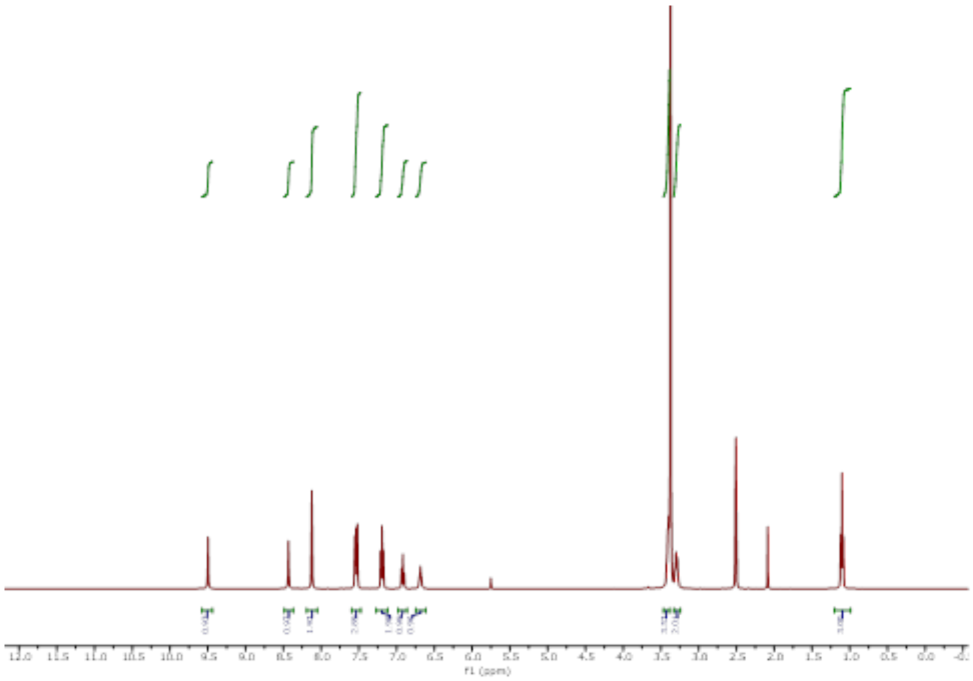

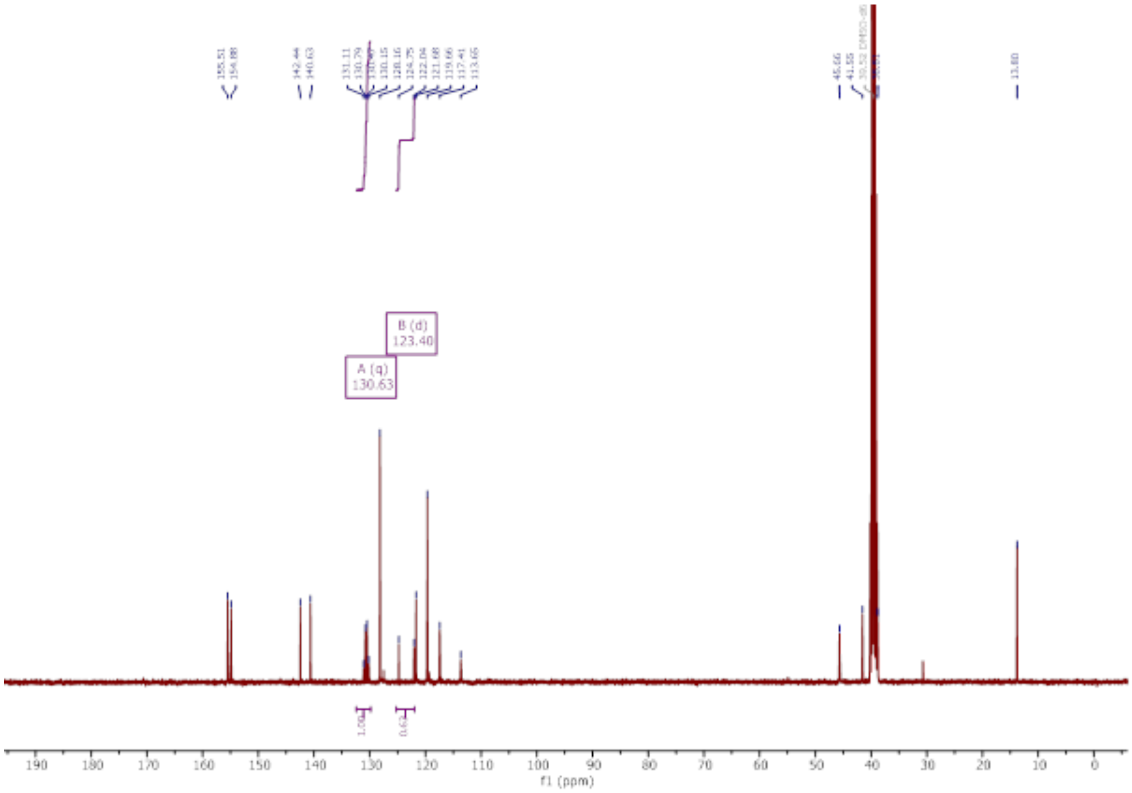

1ba ***N*-(isopropylcarbamoyl)-*N*-benzyl-*N*'-(*tert*butoxycarbonyl)diethylenetriamine (400 MHz, $CDCl_3$)**

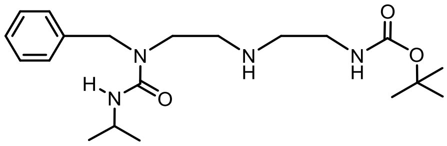

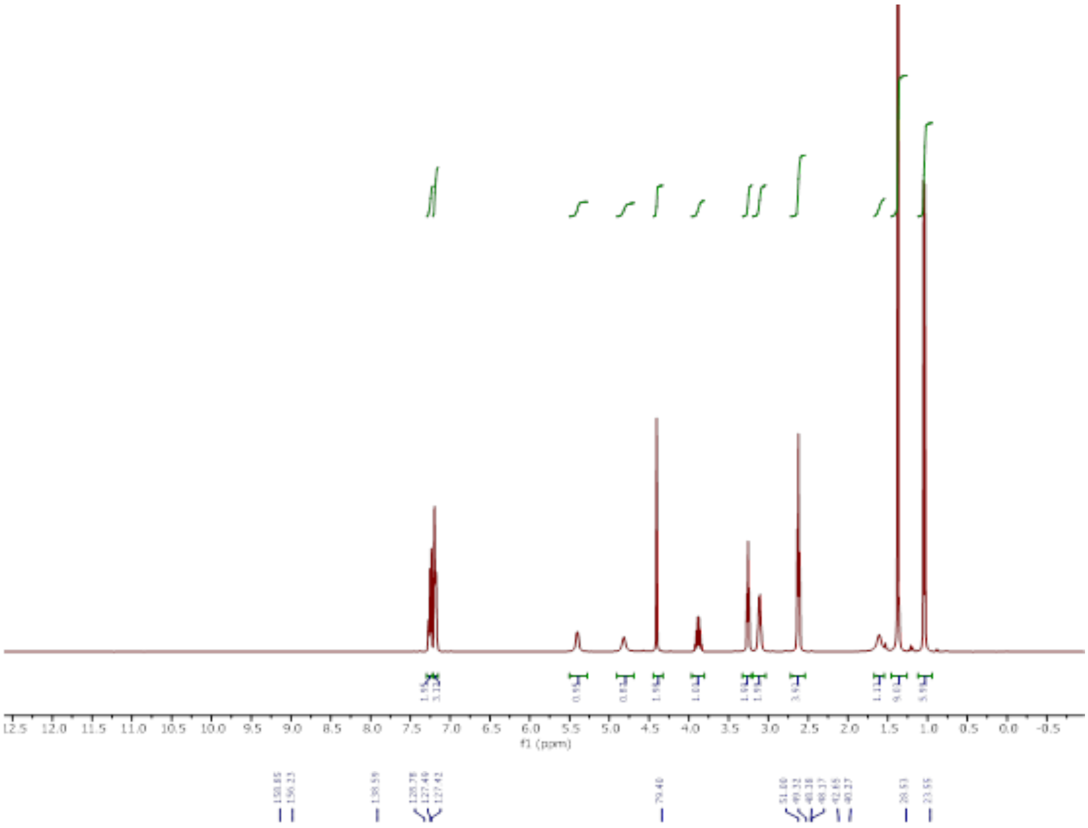

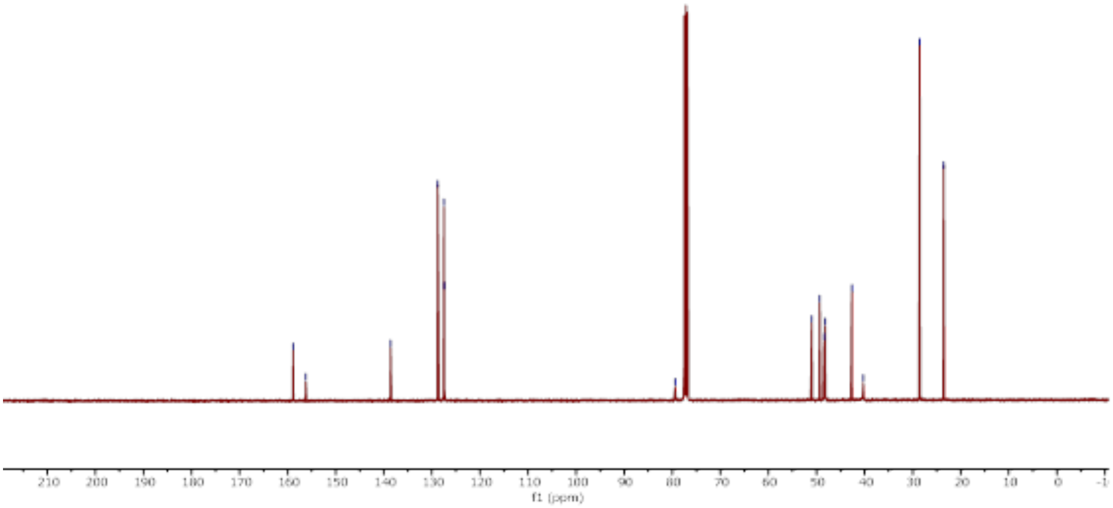

1bb ***N*-(isopropylcarbamoyl)-*N*-benzyl-*N'*-(tertbutoxycarbonyl)-*N''*-(hexylcarbamoyl)diethylenetriamine (400 MHz, $CDCl_3$)**

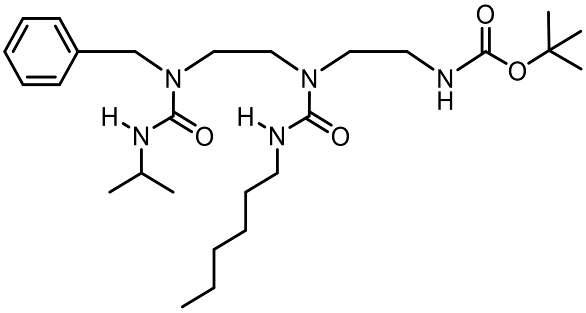

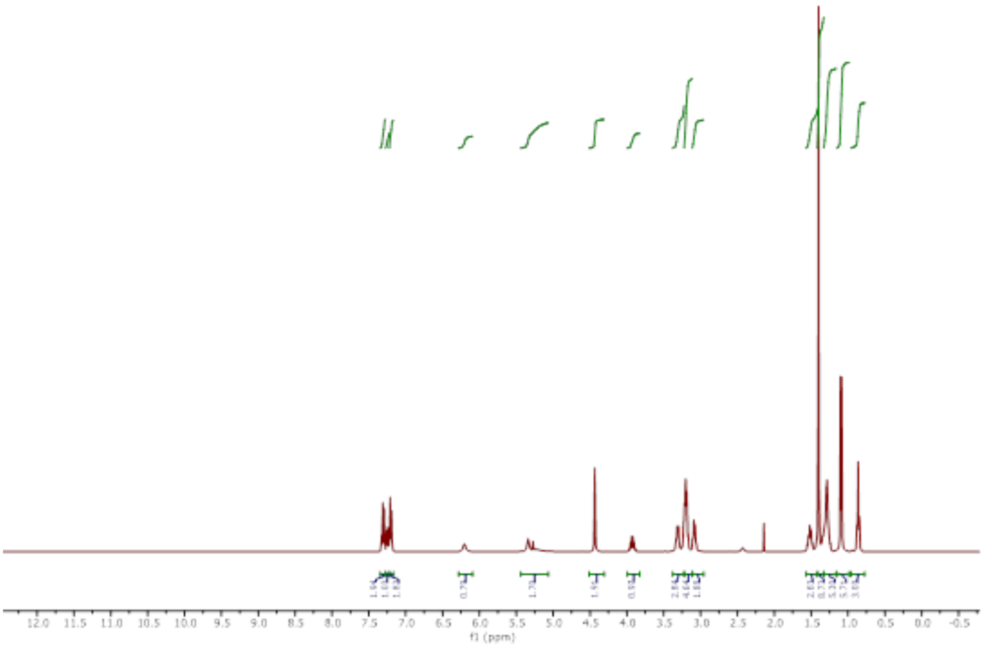
f1 (ppm)

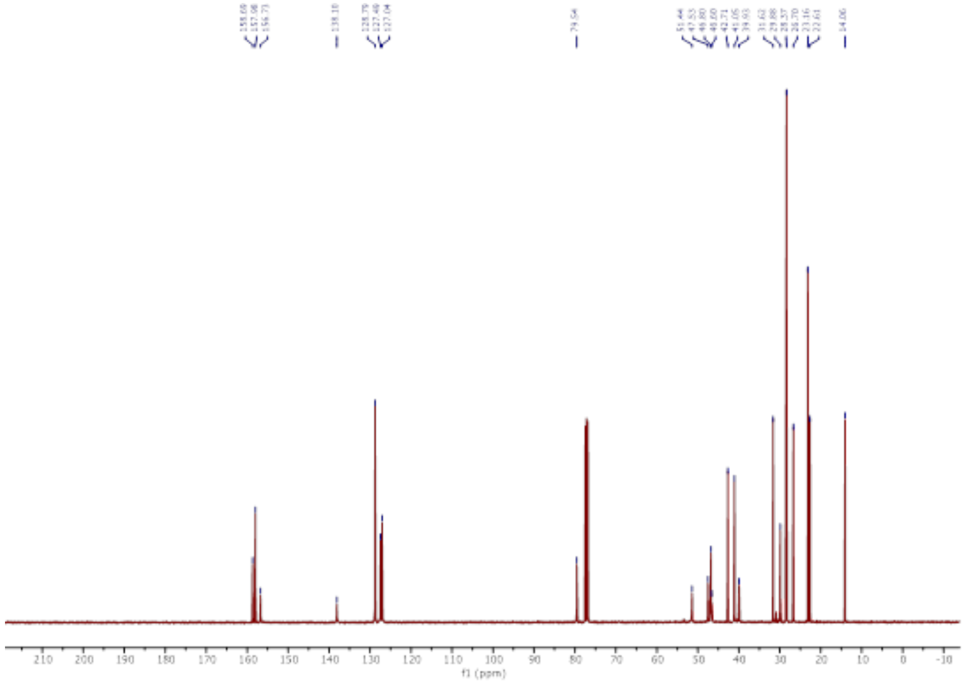
f1 (ppm)

1cb ***N*-(phenylcarbamoyl)-*N*-benzyl-*N*'-(tertbutoxycarbonyl)-*N*''-(butylcarbamoyl)diethylenetriamine (400 MHz, $CDCl_3$)**

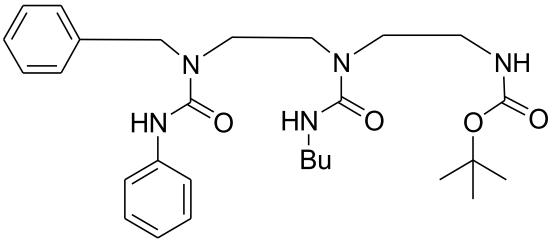

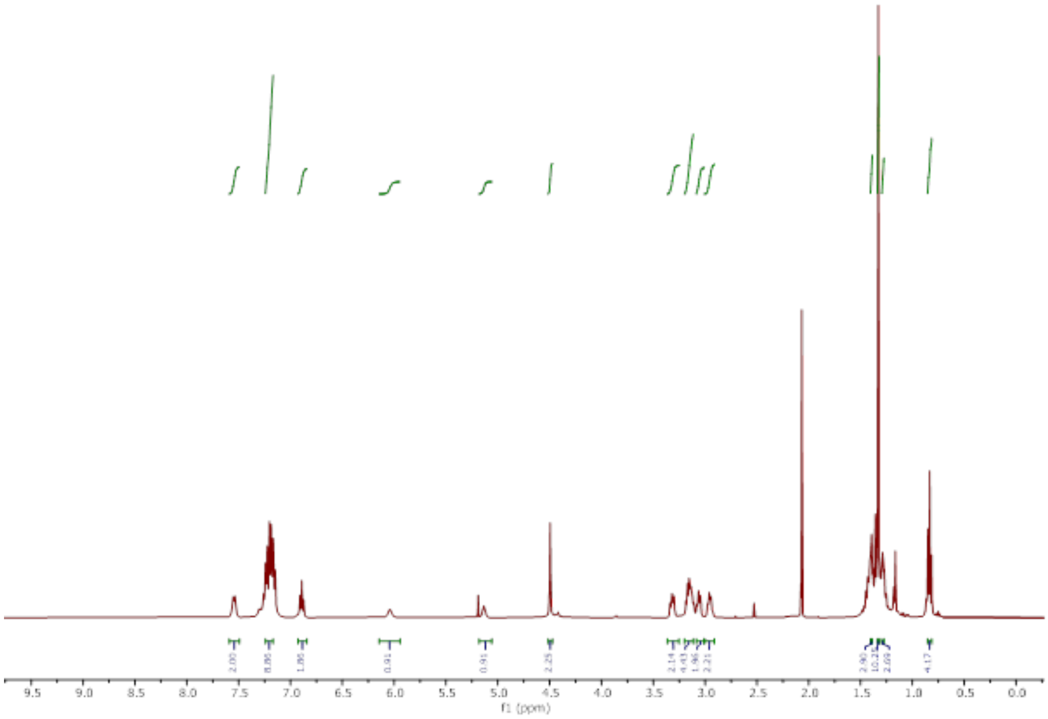

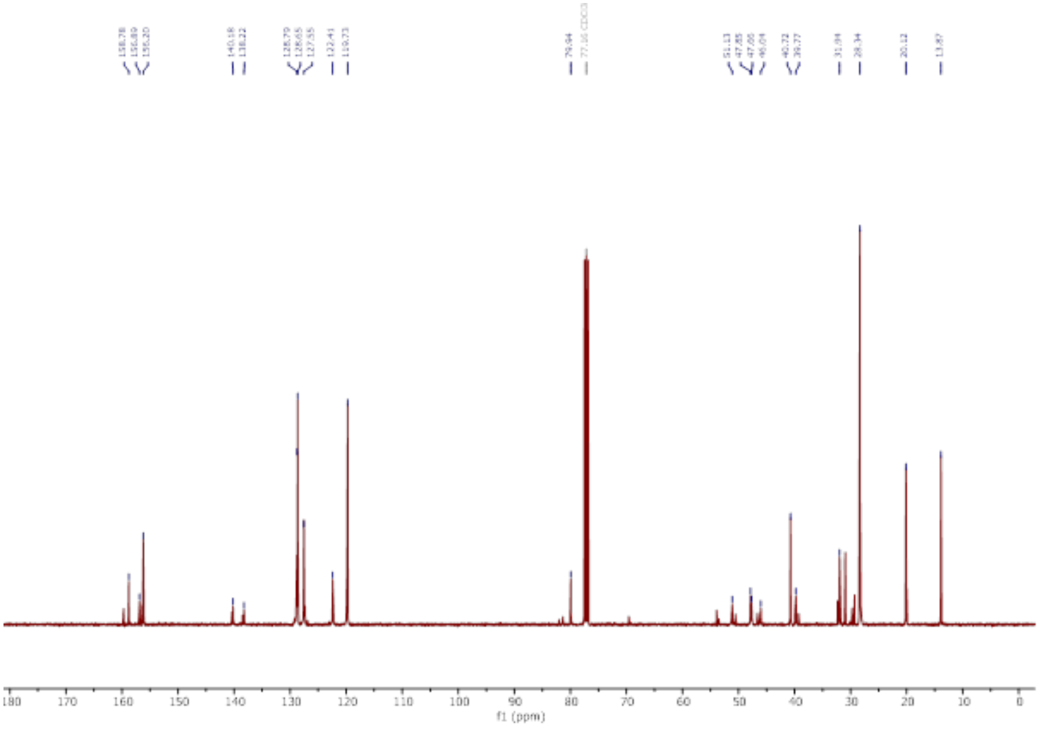

1bc *N*-(isopropylcarbamoyl)-*N*-benzyl-*N*''-(hexylcarbamoyl)diethylenetriamine (400 MHz, $CDCl_3$)

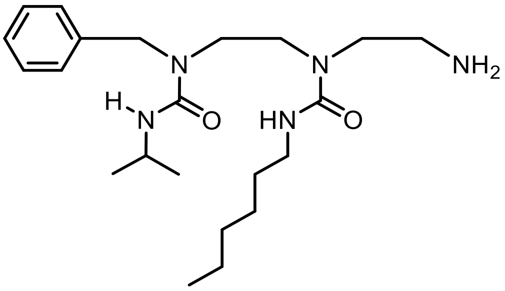

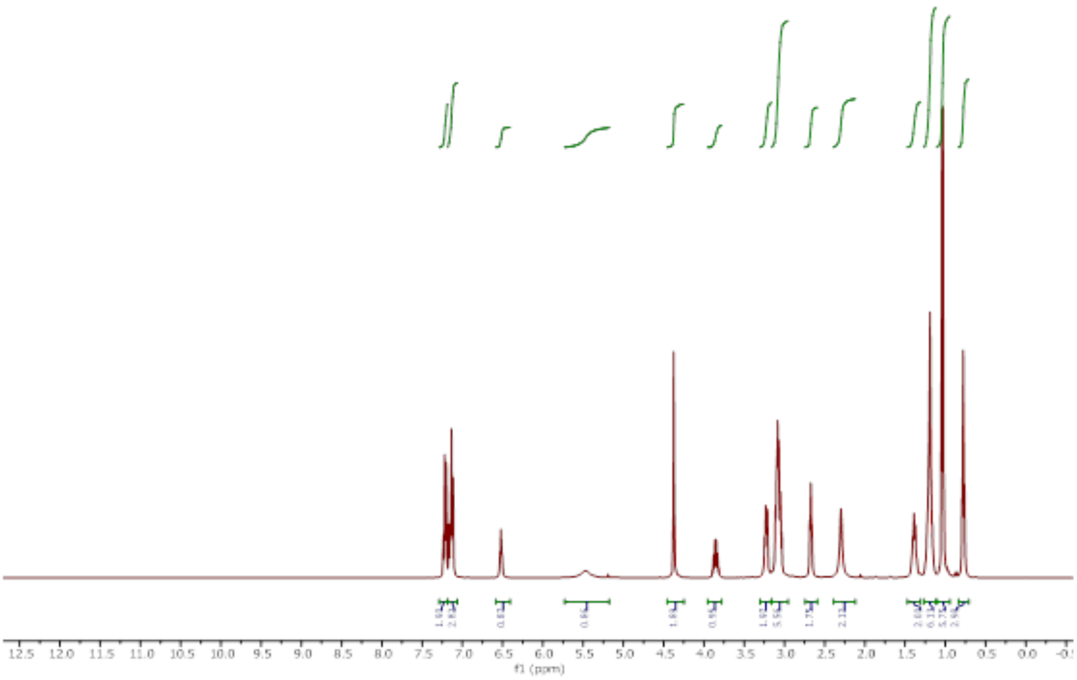

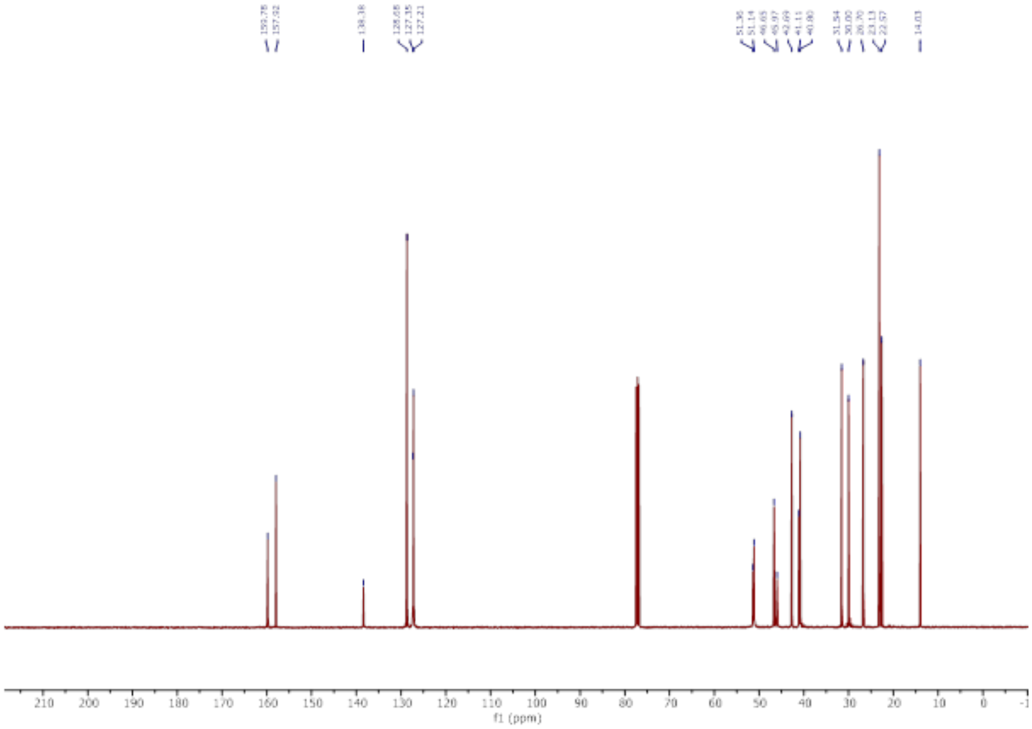

1cc *N*-(phenylcarbamoyl)-*N*-benzyl-*N*''-(butylcarbamoyl)diethylenetriamine (400 MHz, $CDCl_3$)

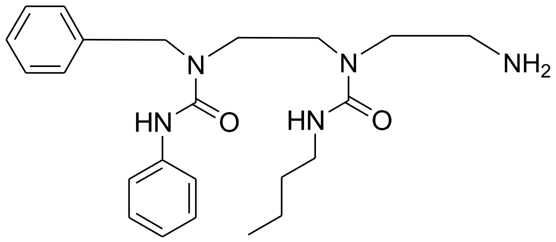

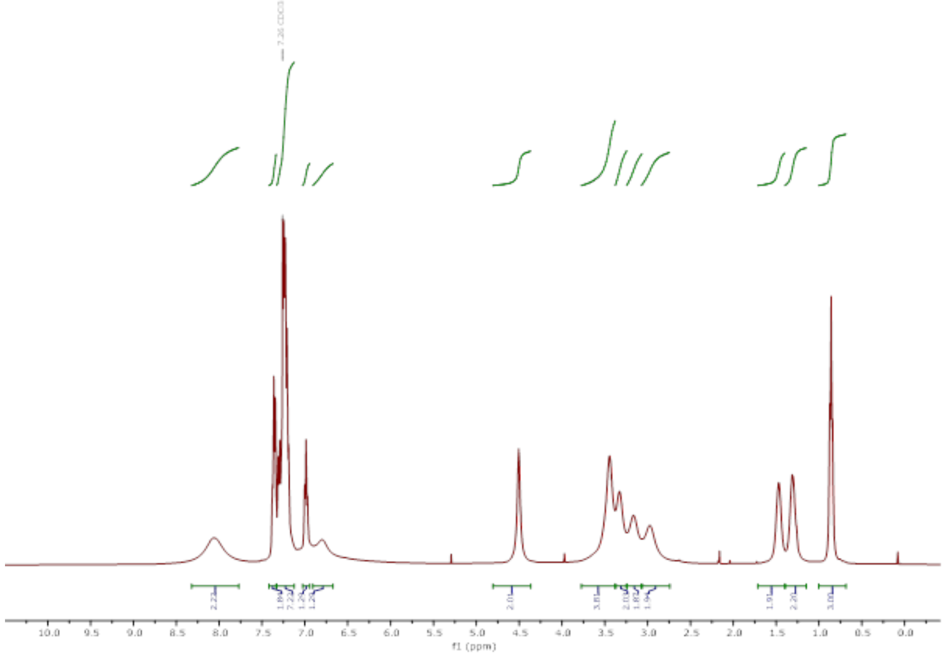

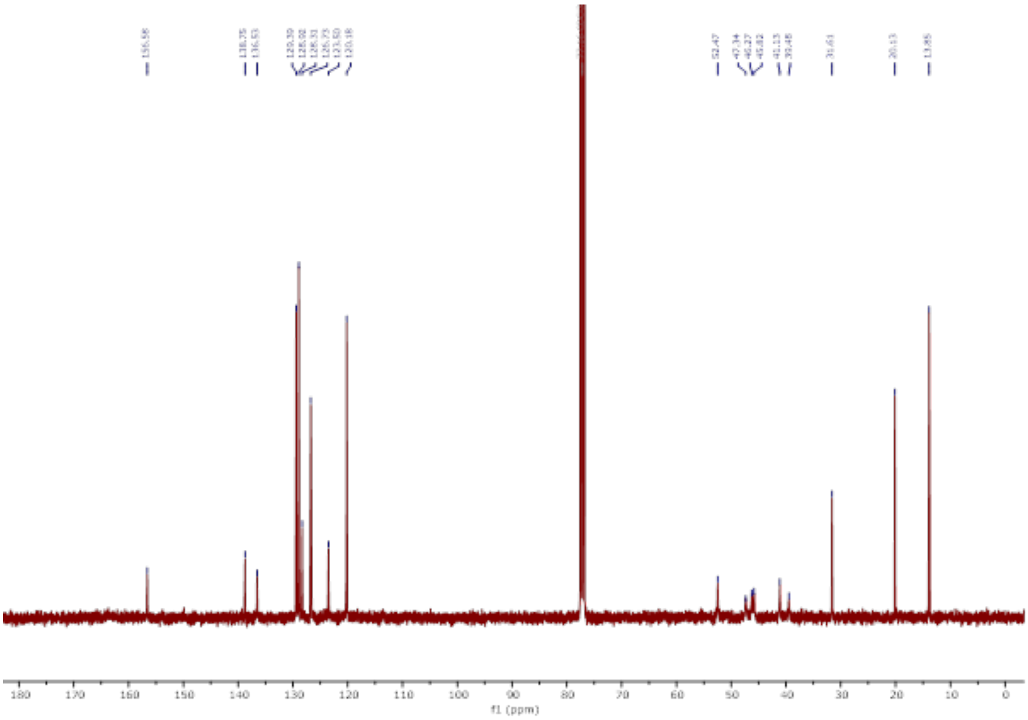

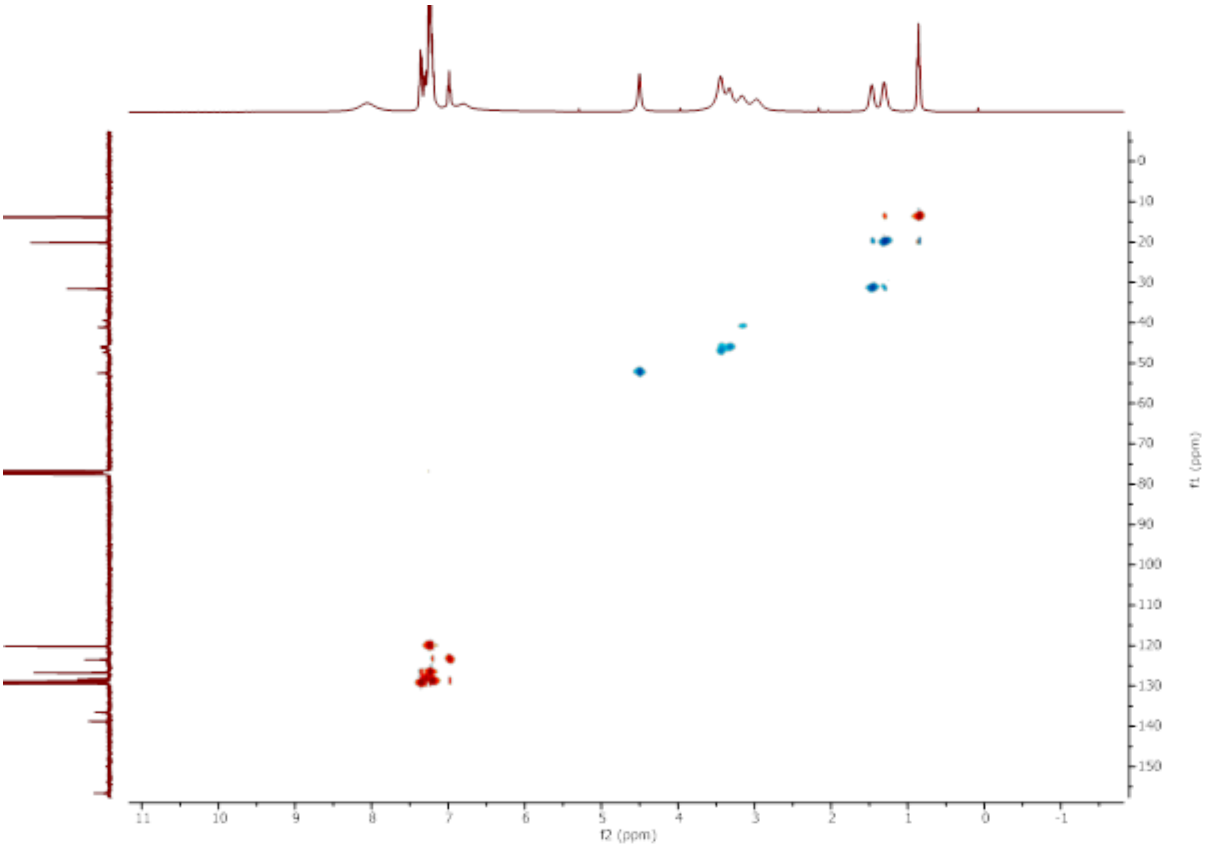

f2 (ppm)
f1 (ppm)

1b ***N*-(isopropylcarbamoyl)-*N*-benzyl-*N*''-(hexylcarbamoyl)-*N'*-(3,5-*bis*(trifluoromethyl)phenylcarbamoyl)diethylenetriamine (400 MHz, $CDCl_3$)**

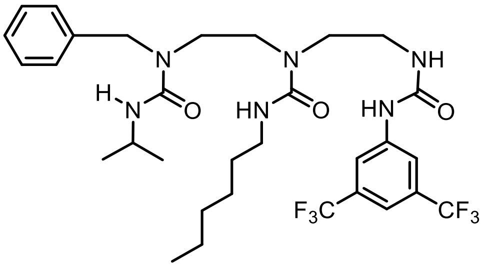

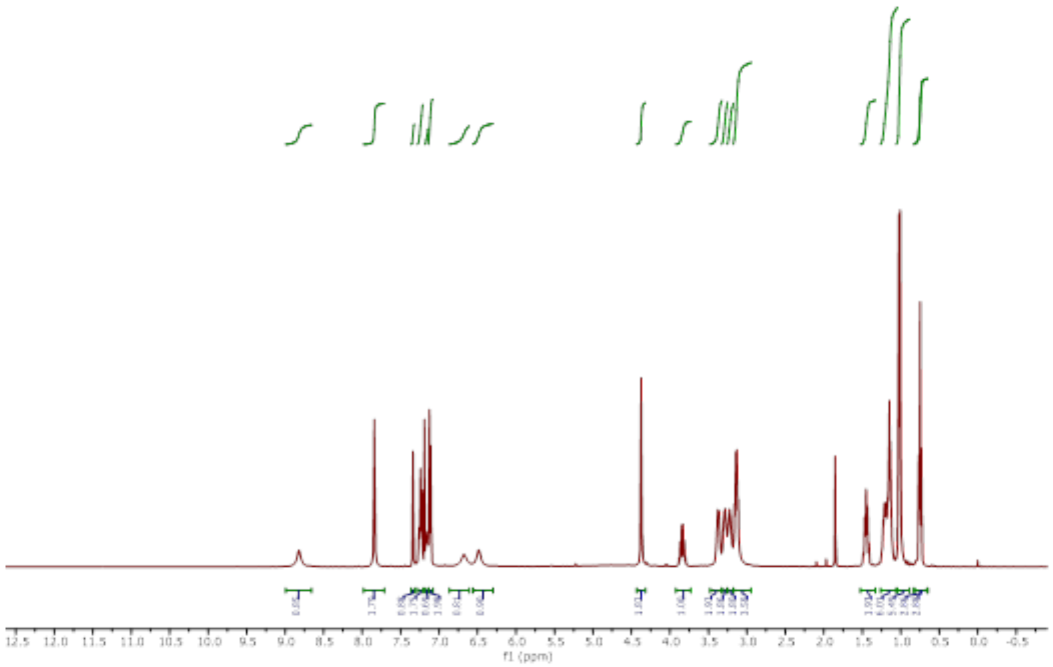

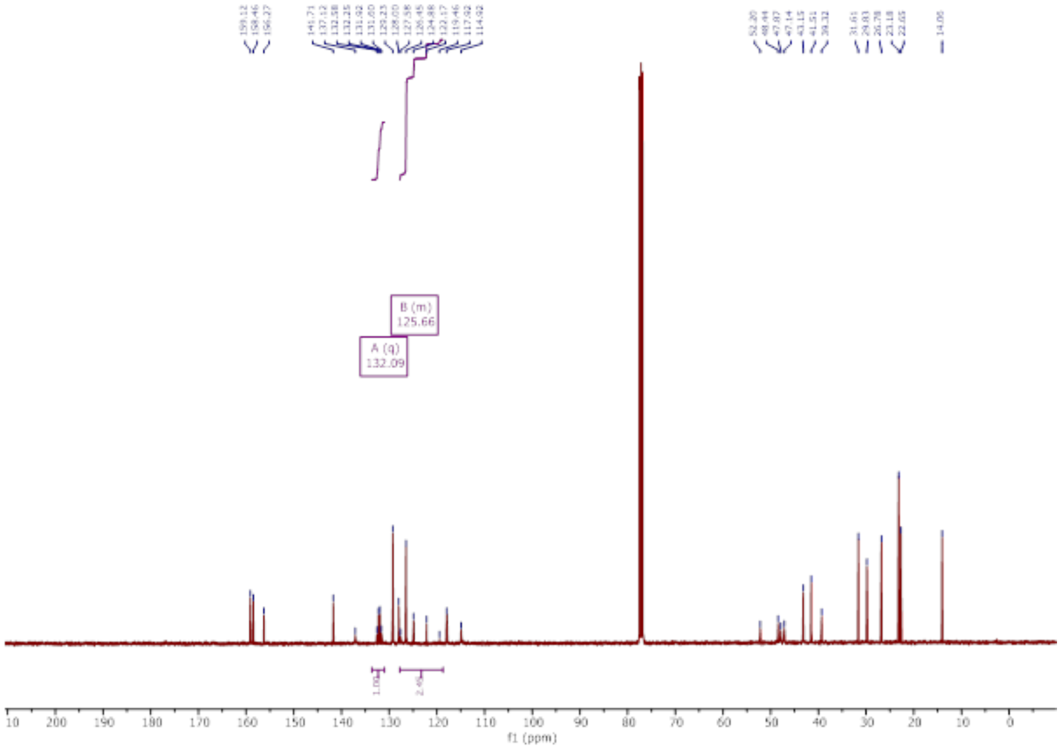

1c ***N*-(phenylcarbamoyl)-*N*-benzyl-*N*''-(butylcarbamoyl)-*N'*-(3,5-*bis*(trifluoromethyl)phenylcarbamoyl)diethylenetriamine (400 MHz, $CDCl_3$)**

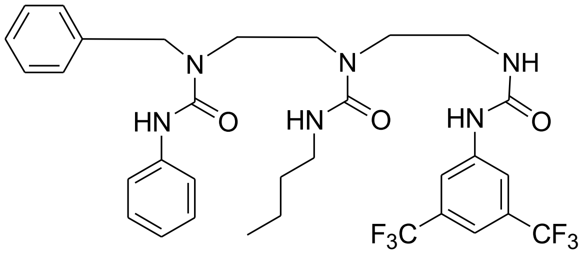

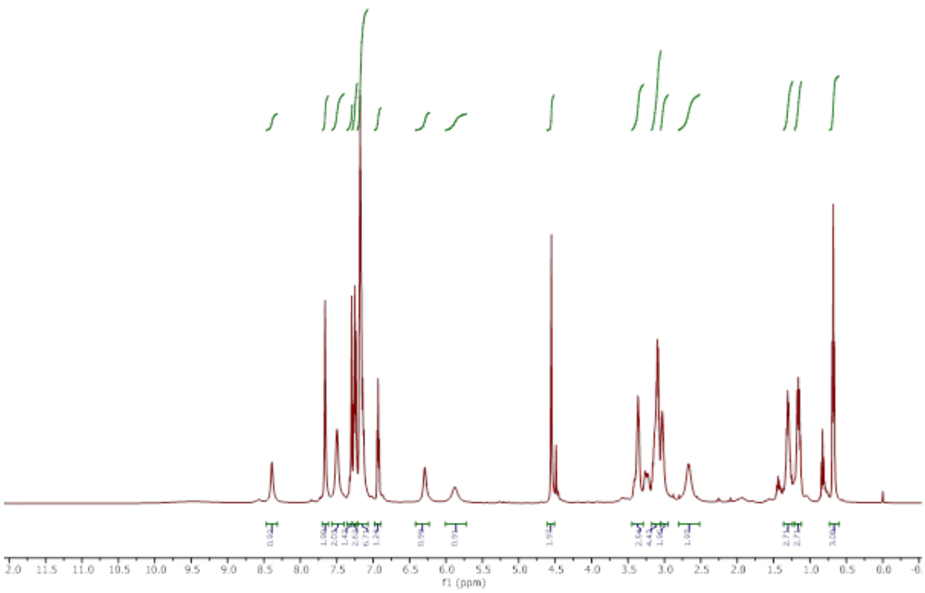

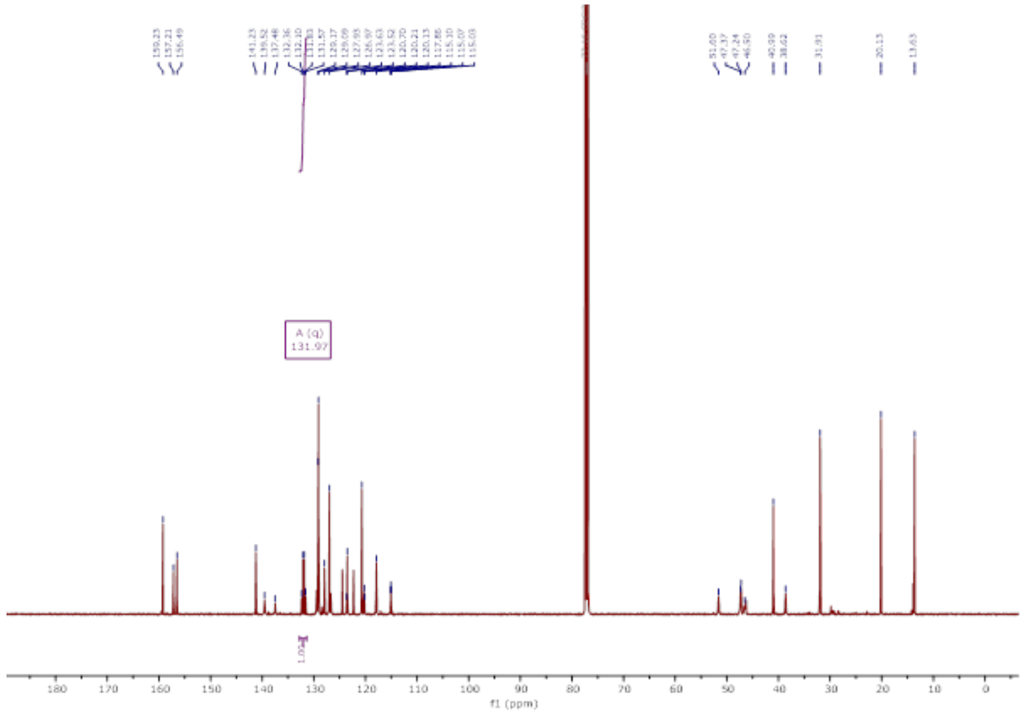

1aa *N*-benzyl-*N*'-(*tert*butoxycarbonyl)diethylenetriamine (400 MHz, $CDCl_3$)

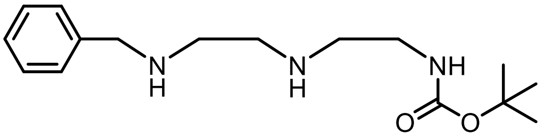

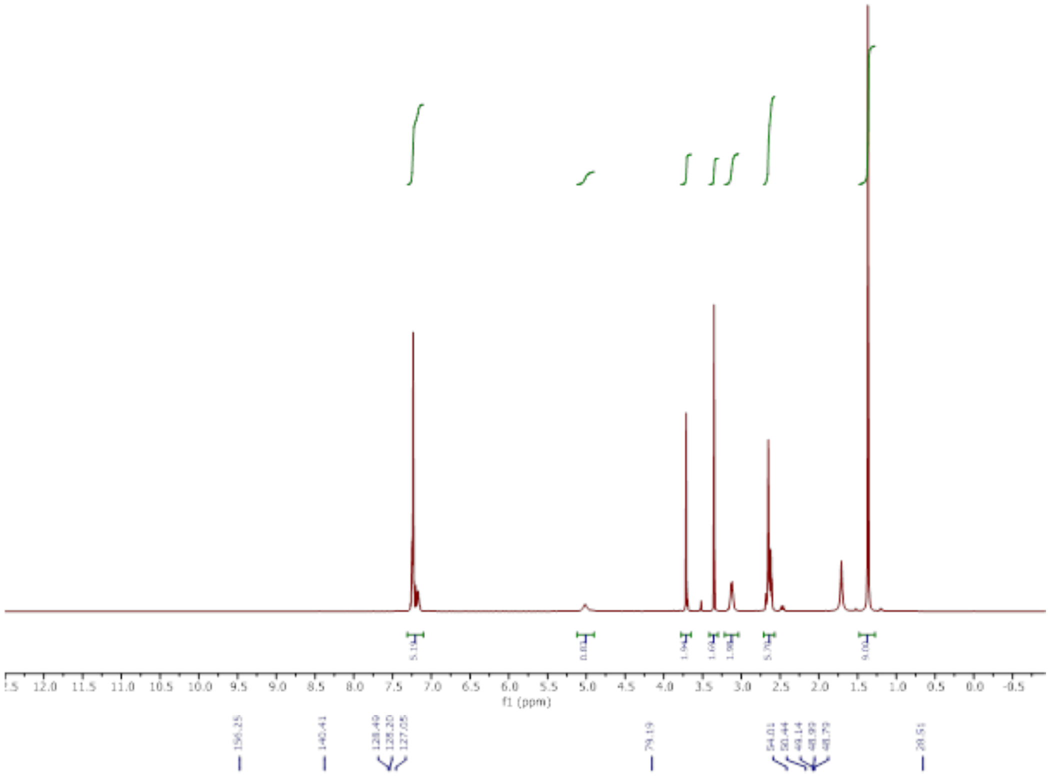

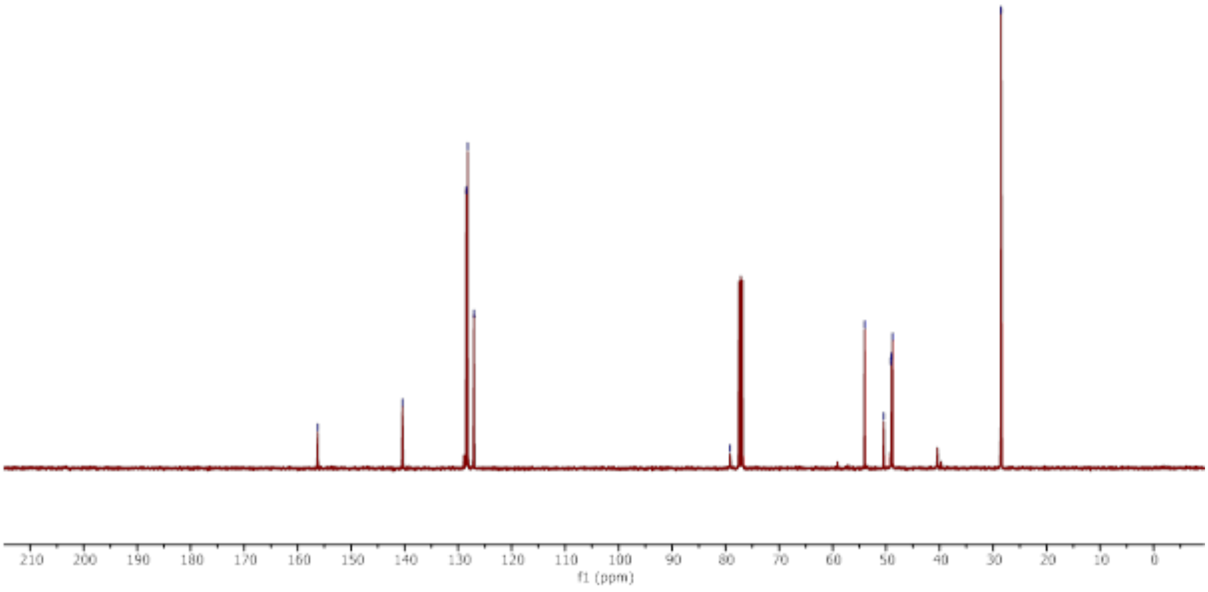

3aa *N*-ethyl-*N*'-(tertbutoxycarbonyl)diethylenetriamine (400 MHz, $CDCl_3$)

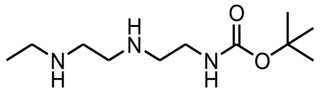

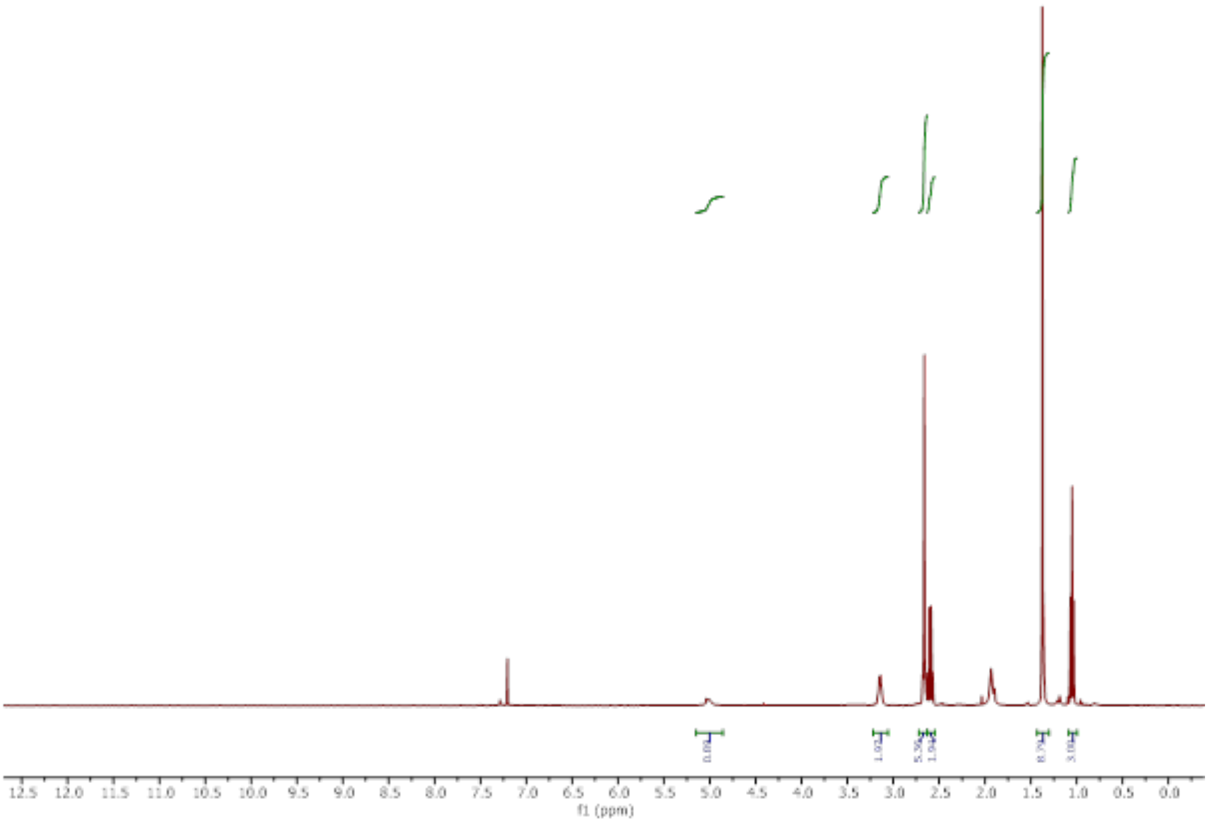

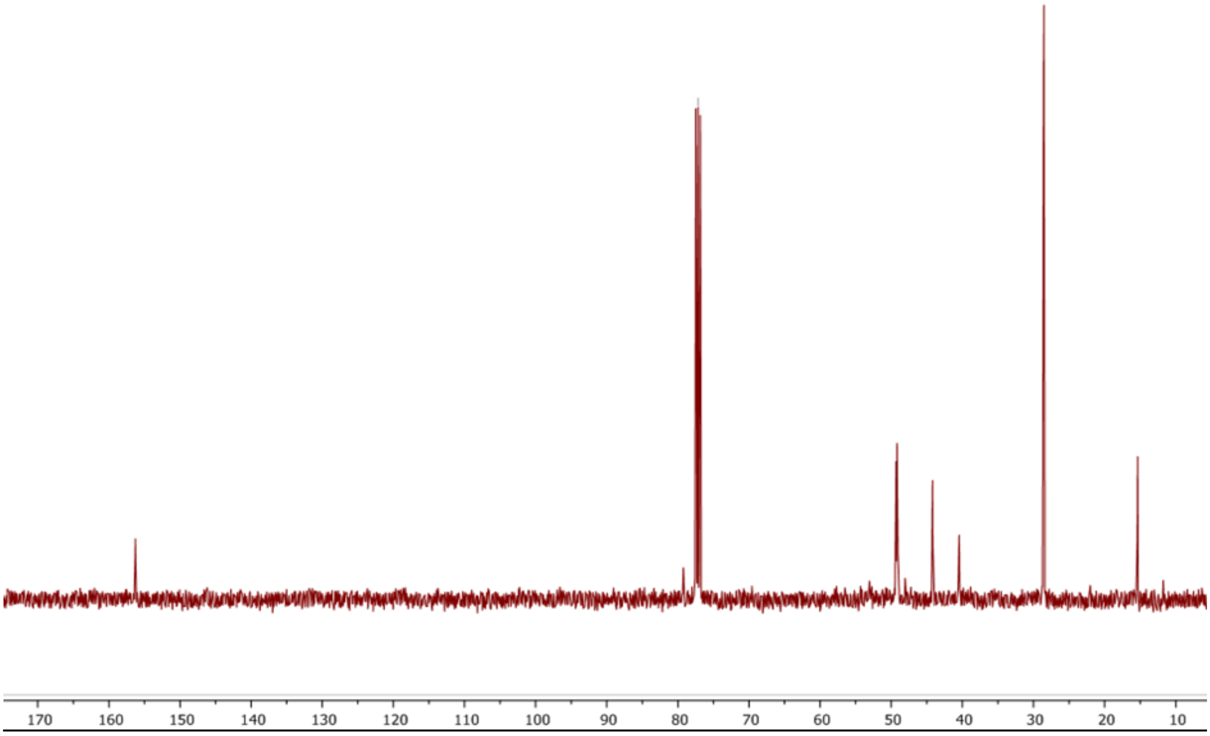

1ab *N,N''-bis*(hexylcarbamoyl)-*N*-benzyl-*N*'-(tertbutoxycarbonyl)diethylenetriamine (400 MHz, $CDCl_3$)

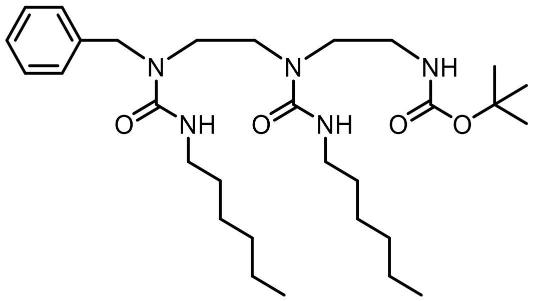

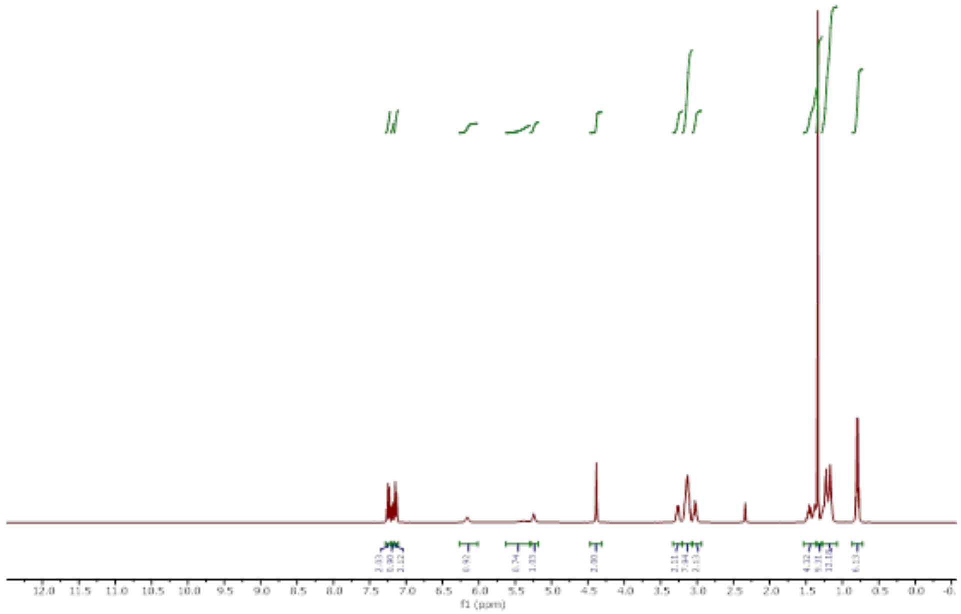

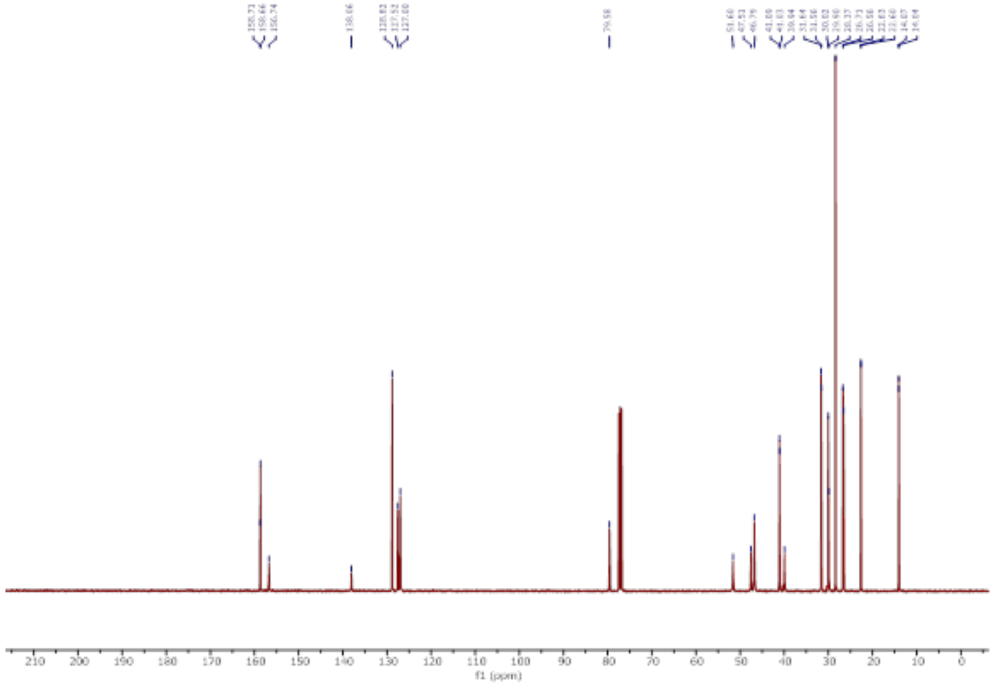

1db *N*-(phenylcarbamoyl)-*N*-benzyl-*N*'-(tertbutoxycarbonyl)-*N*''-(phenylcarbamoyl)diethylenetriamine (400 MHz, $CDCl_3$)

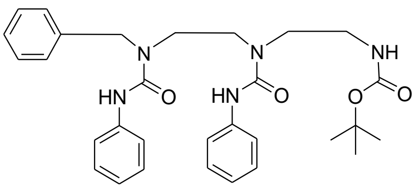

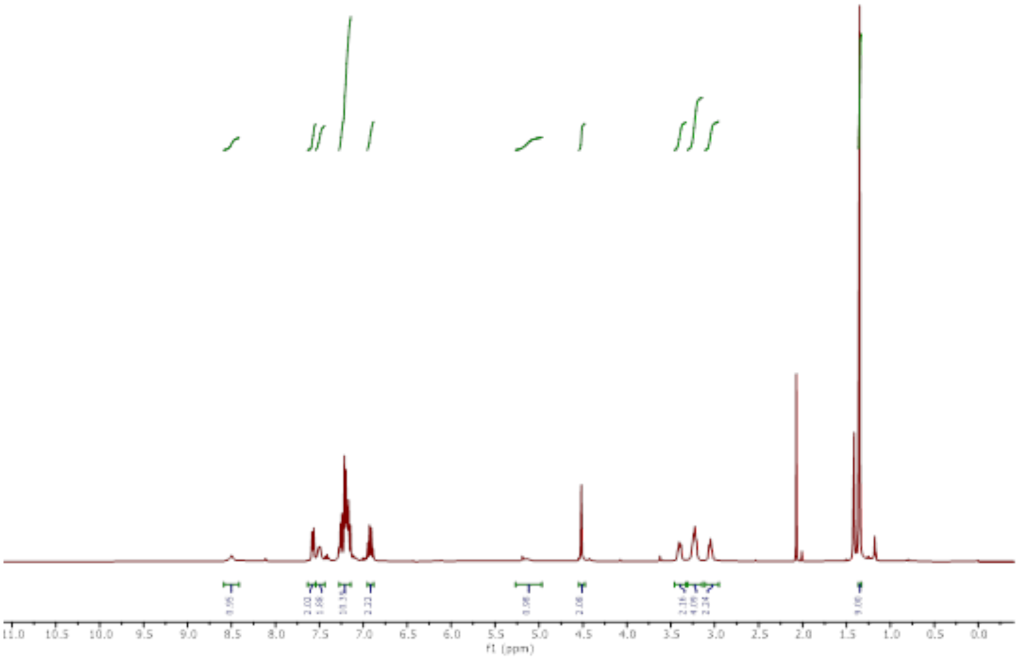

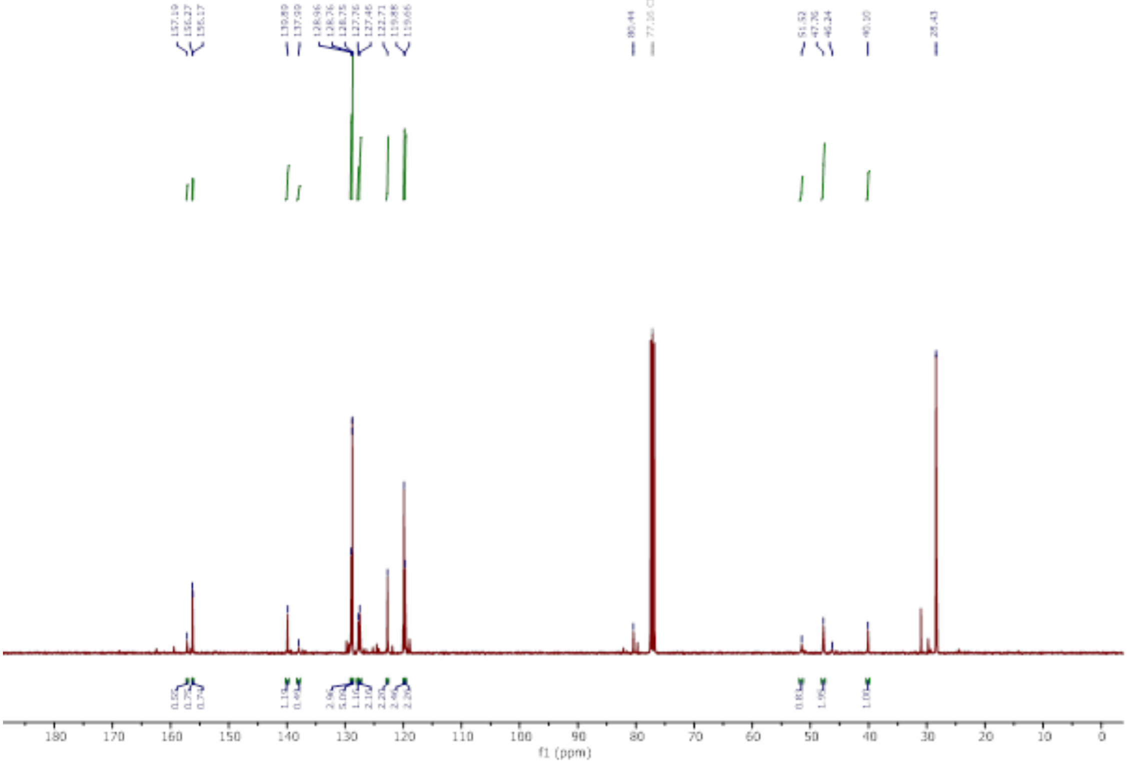

1eb *N,N''-bis*(4-butoxyphenylcarbamoyl)-*N*-benzyl-*N*'-(*tert*butoxycarbonyl)diethylenetriamine (400 MHz, $CDCl_3$)

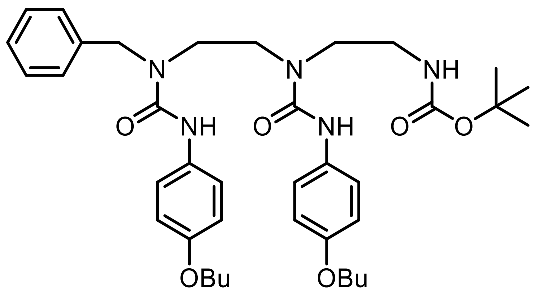

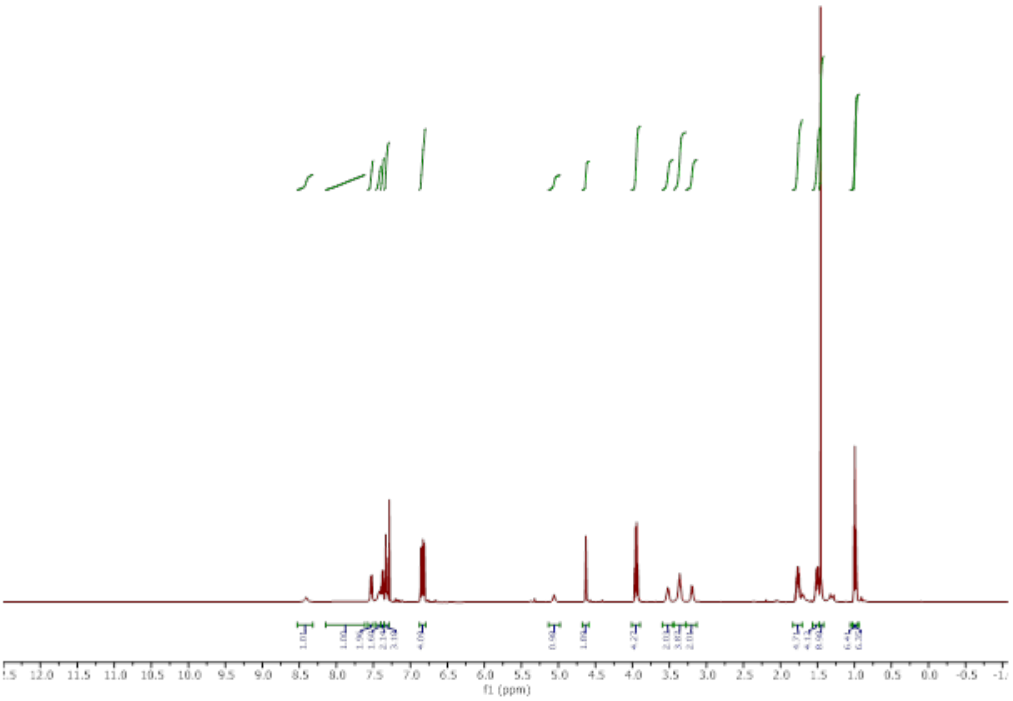

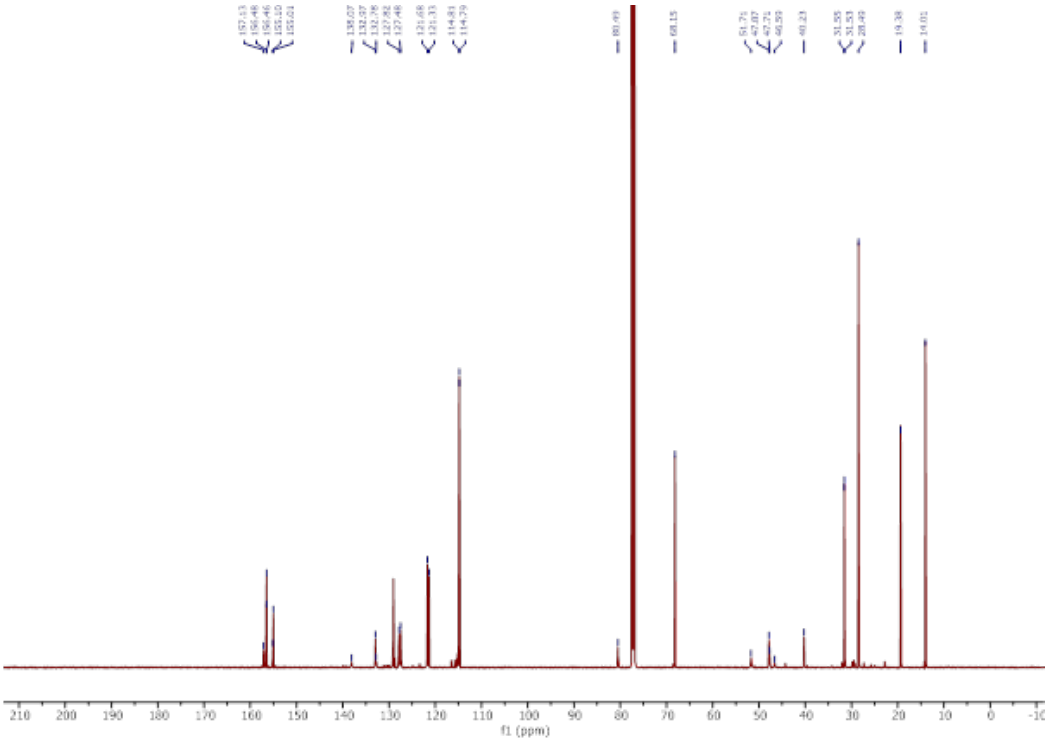

3ab *N*-ethyl-*N,N''-bis*(phenylcarbamoyl)-*N*'-(tertbutoxycarbonyl)diethylenetriamine (400 MHz, $CDCl_3$)

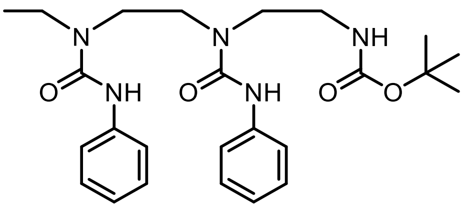

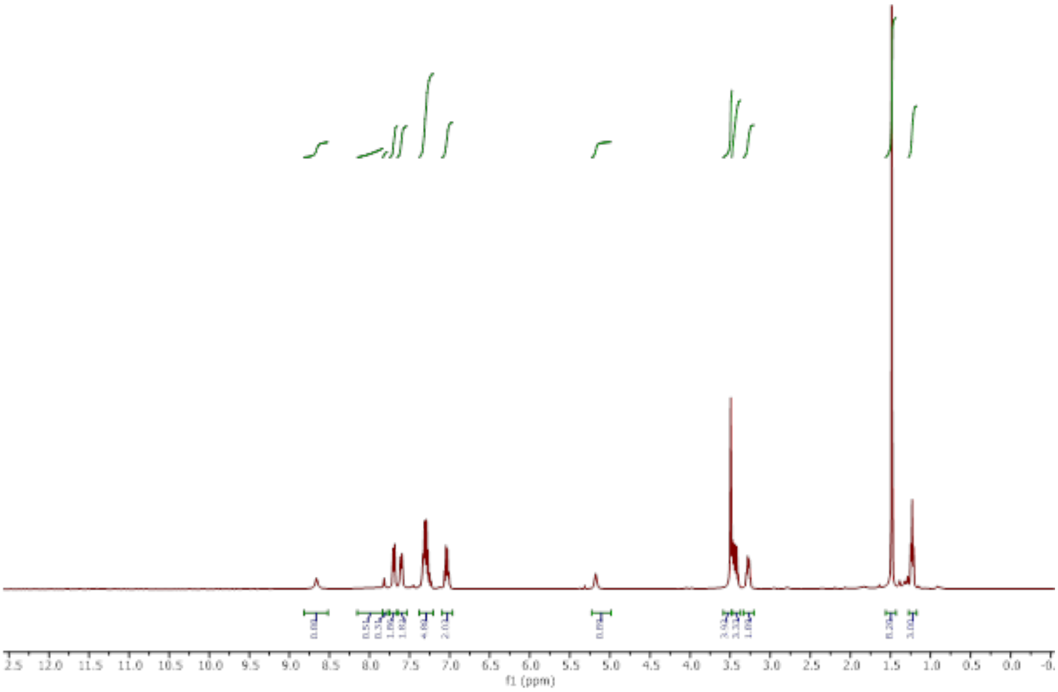

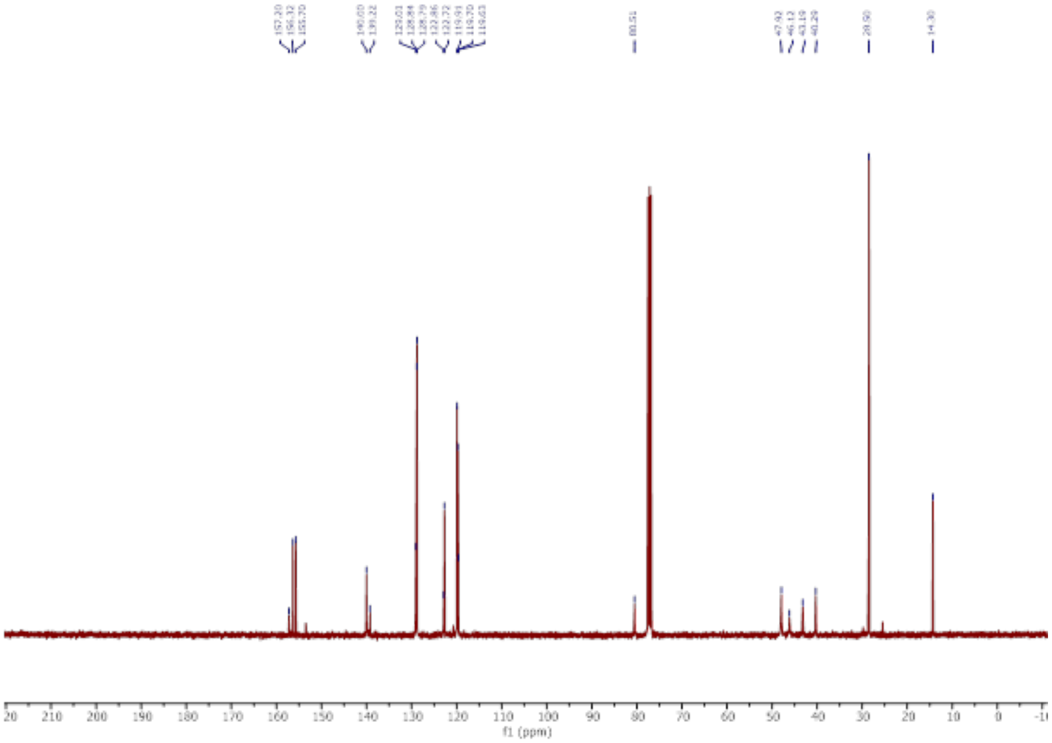

1ac *N,N''-bis*(hexylcarbamoyl)-*N*-benzyldiethylenetriamine, trifluoroacetic salt (400 MHz, $CDCl_3$)

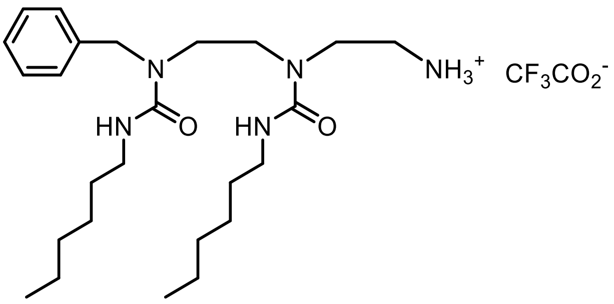

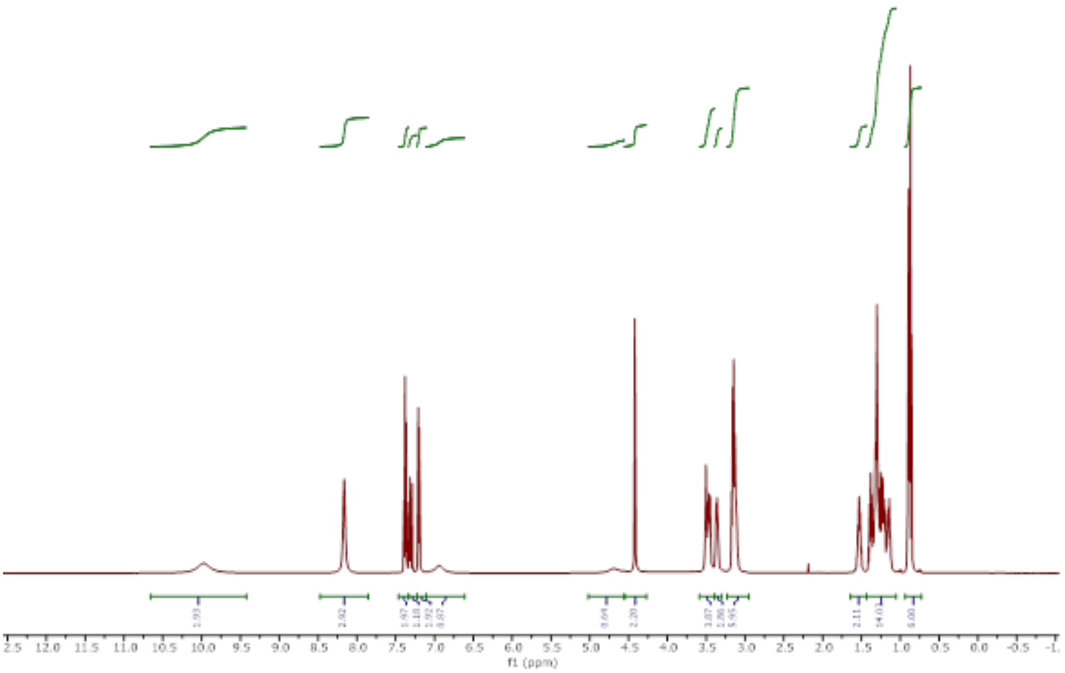

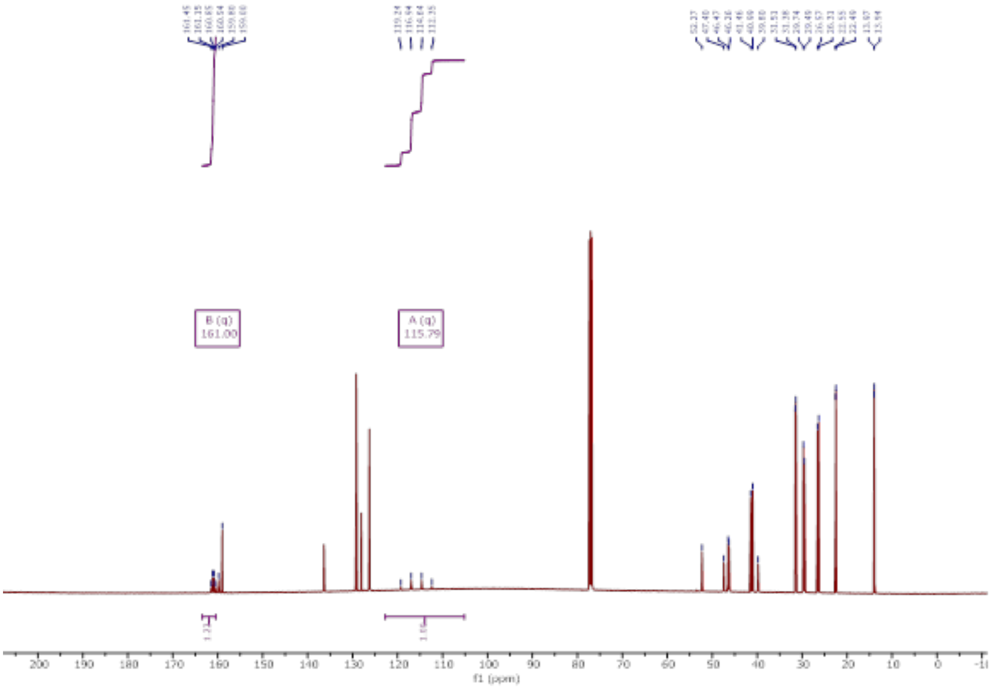

1dc *N*-(phenylcarbamoyl)-*N*-benzyl-*N*''-(phenylcarbamoyl)diethylenetriamine (400 MHz, $CDCl_3$)

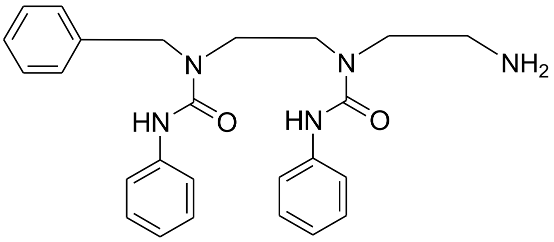

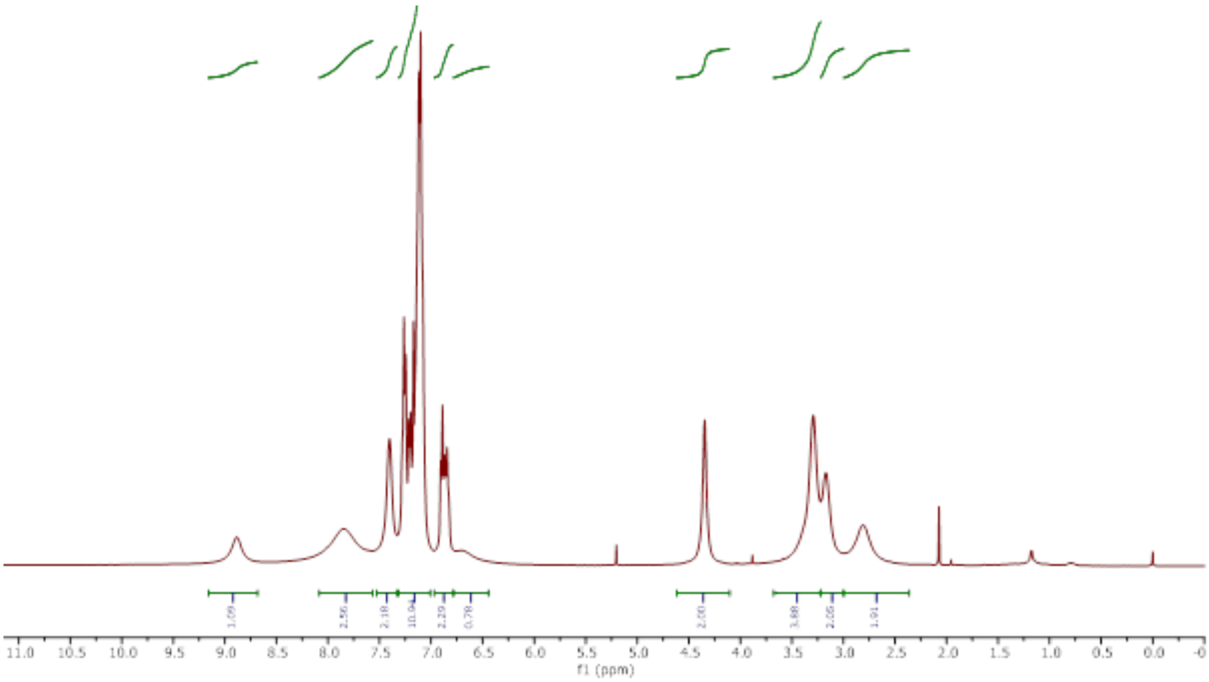

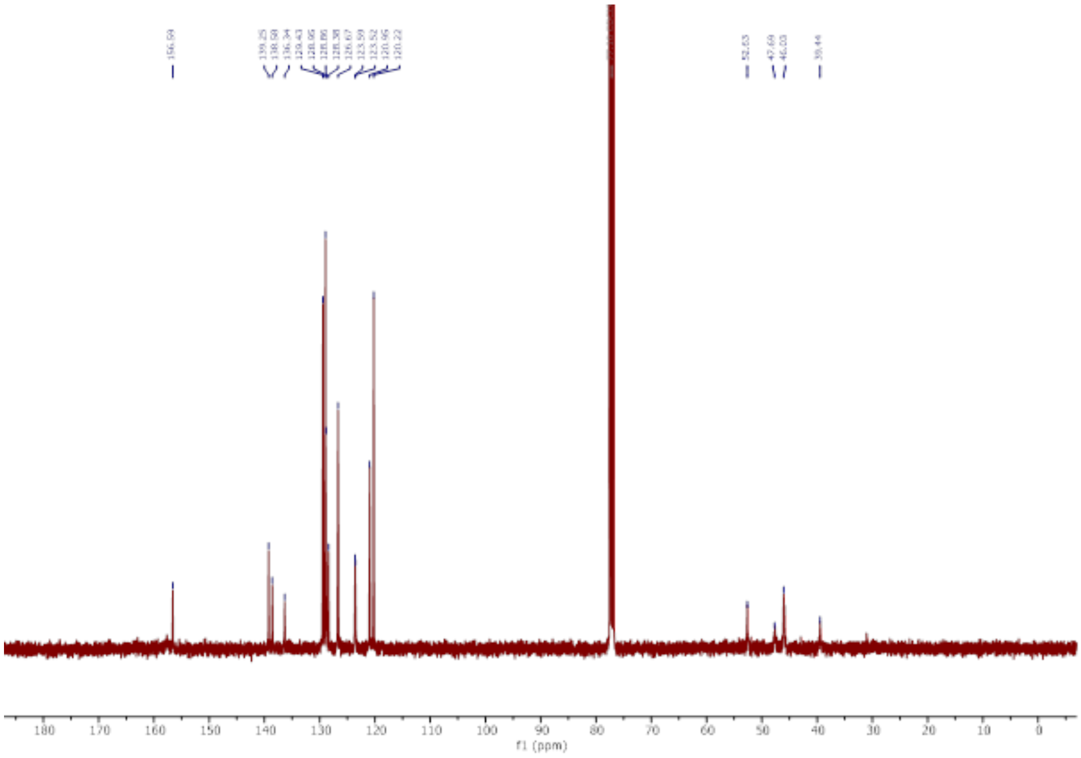

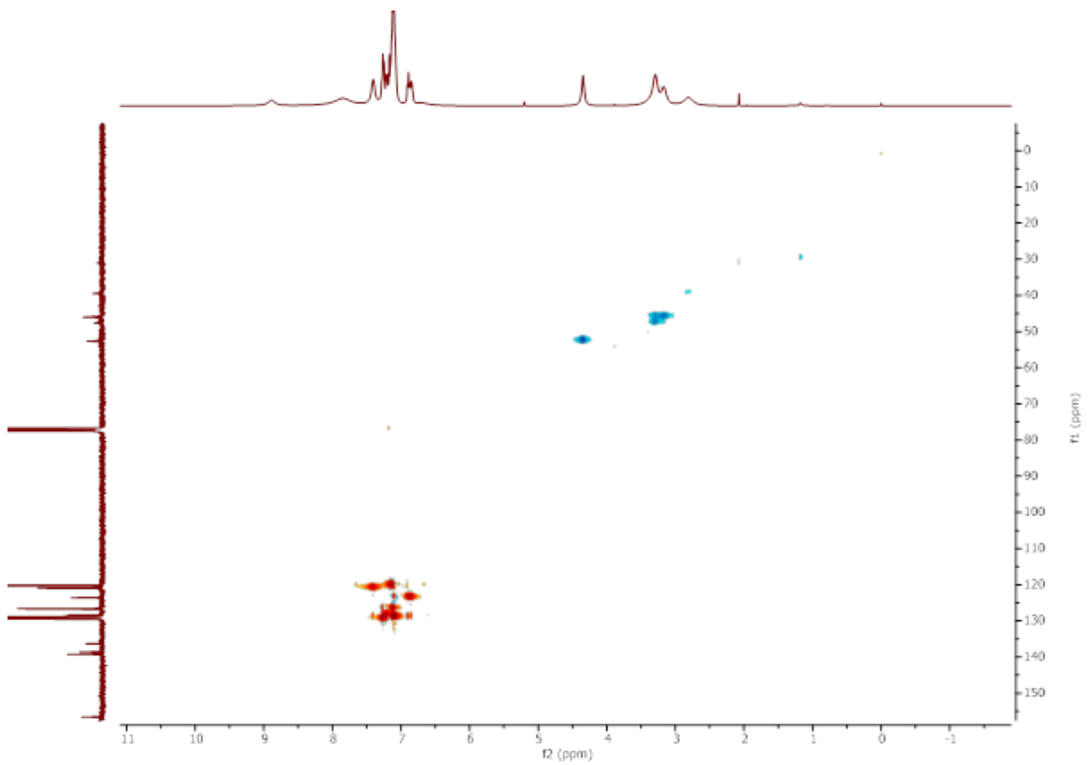

f2 (ppm)
f1 (ppm)

1ec *N,N''-bis*(4-butoxyphenylcarbamoyl)-*N*-benzyldiethylenetriamine, trifluoroacetic salt (400 MHz, $CDCl_3$)

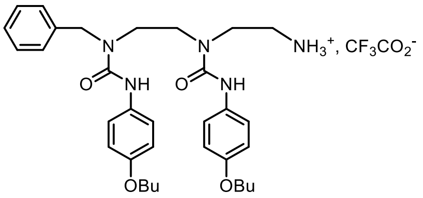

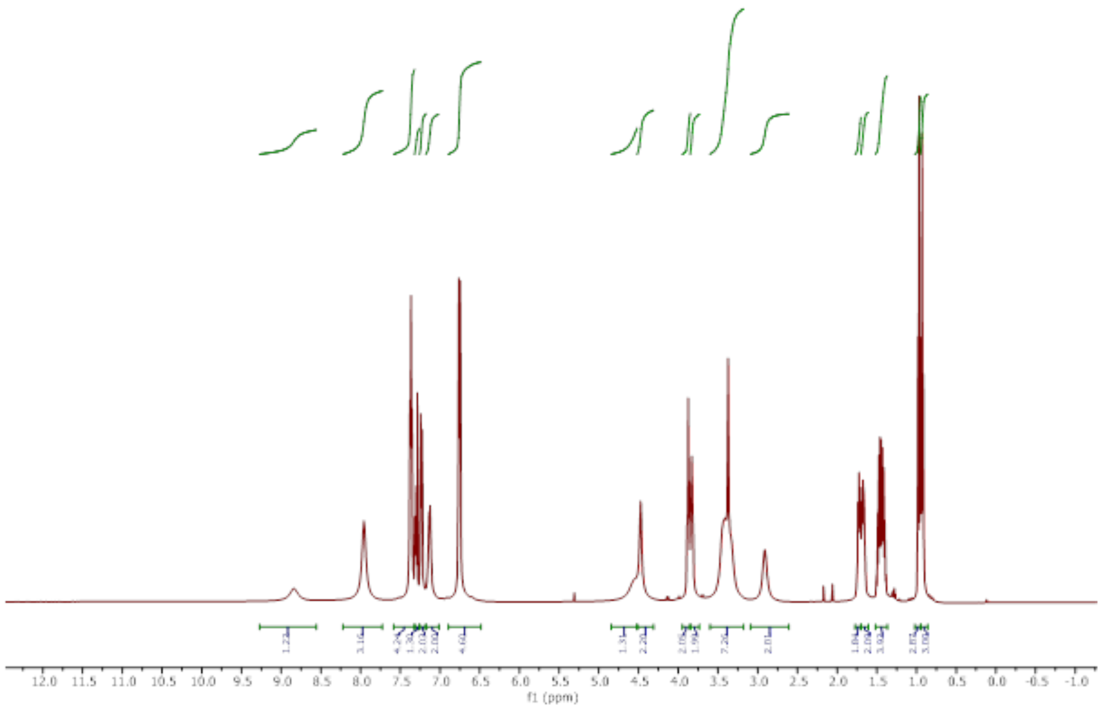

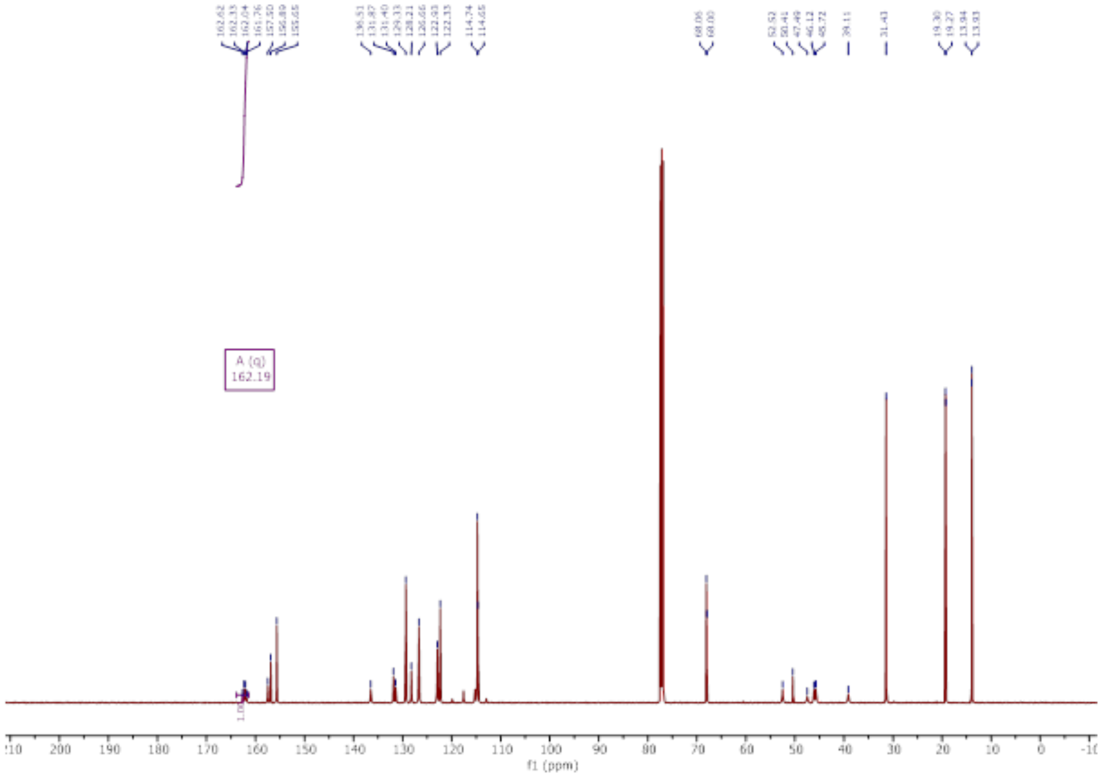

3ac *N,N''-bis*(phenylcarbamoyl)-*N*-ethyldiethylenetriamine, trifluoroacetic salt (400 MHz, $CDCl_3$)

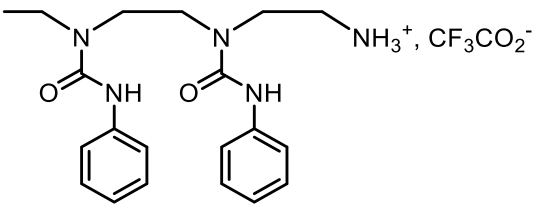

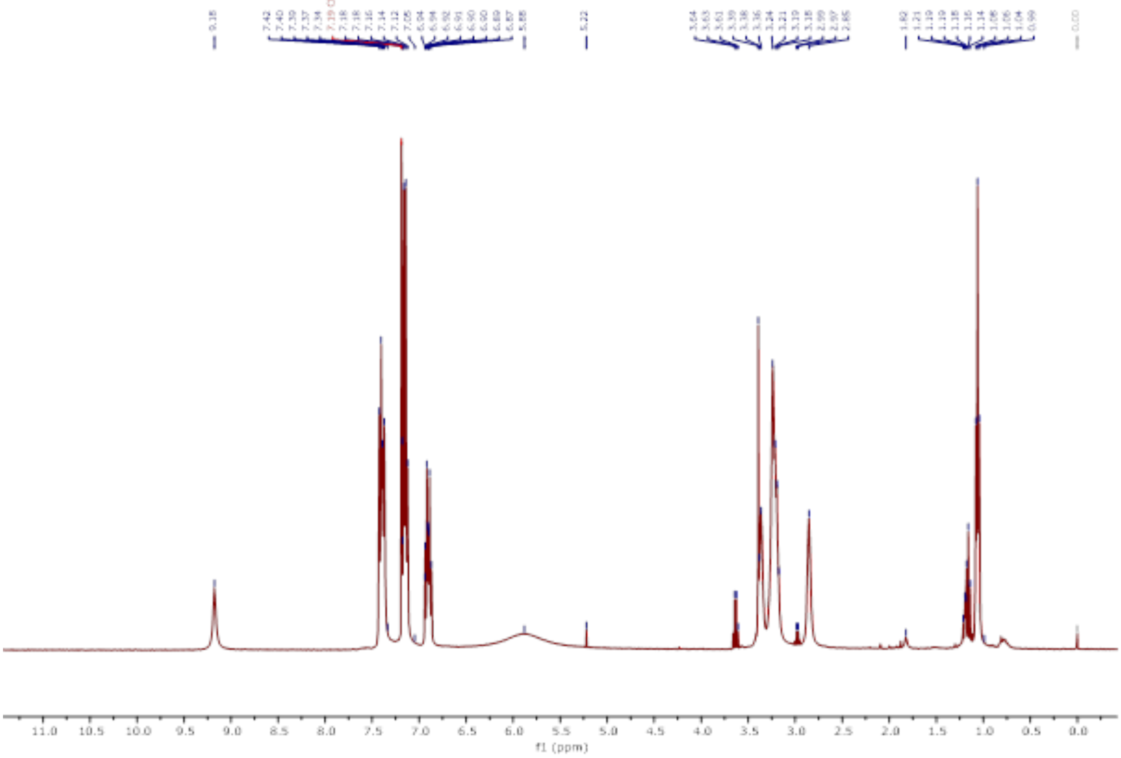

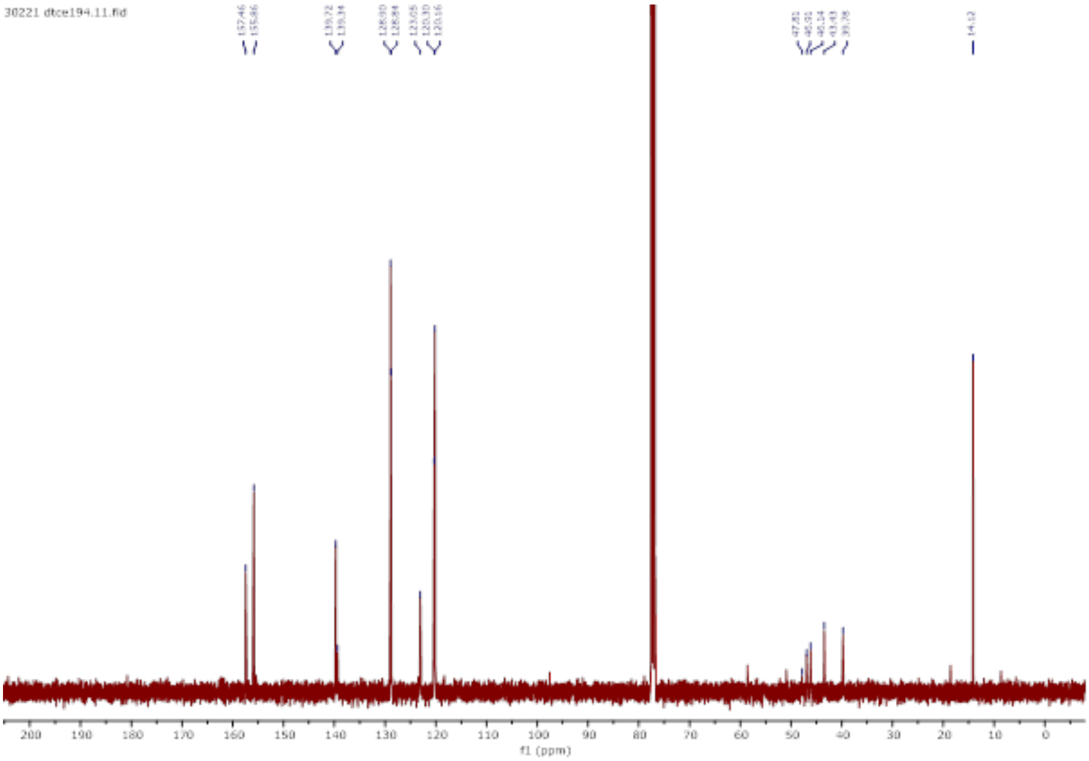

1a ***N,N''-bis*(hexylcarbamoyl)-*N*-benzyl-*N'*-(3,5-*bis*(trifluoromethyl)phenylcarbamoyl)diethylenetriamine (400 MHz, CD_2Cl_2)**

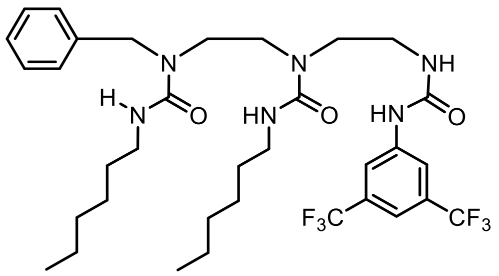

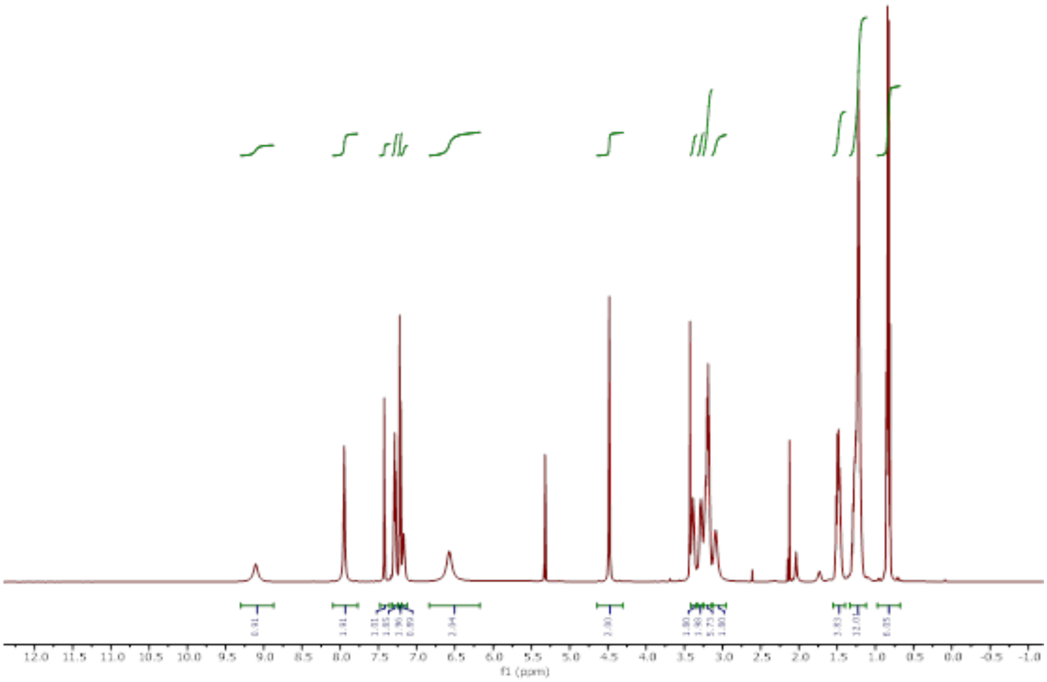

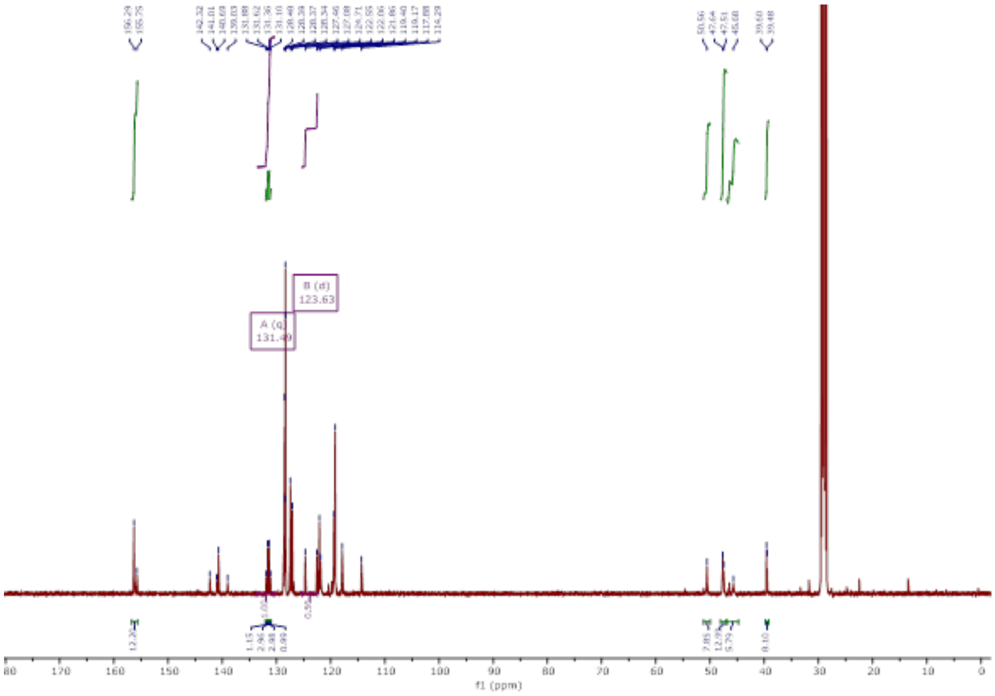

1d ***N*-(phenylcarbamoyl)-*N*-benzyl-*N*''-(phenylcarbamoyl)-*N'*-(3,5-*bis*(trifluoromethyl)phenylcarbamoyl)diethylenetriamine (400 MHz, acetone d6)**

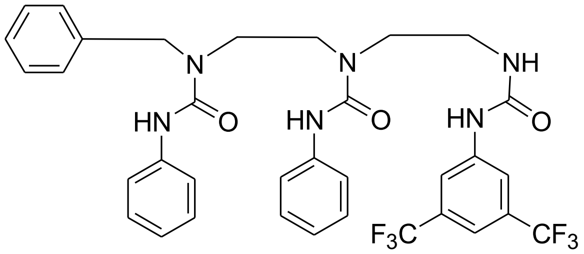

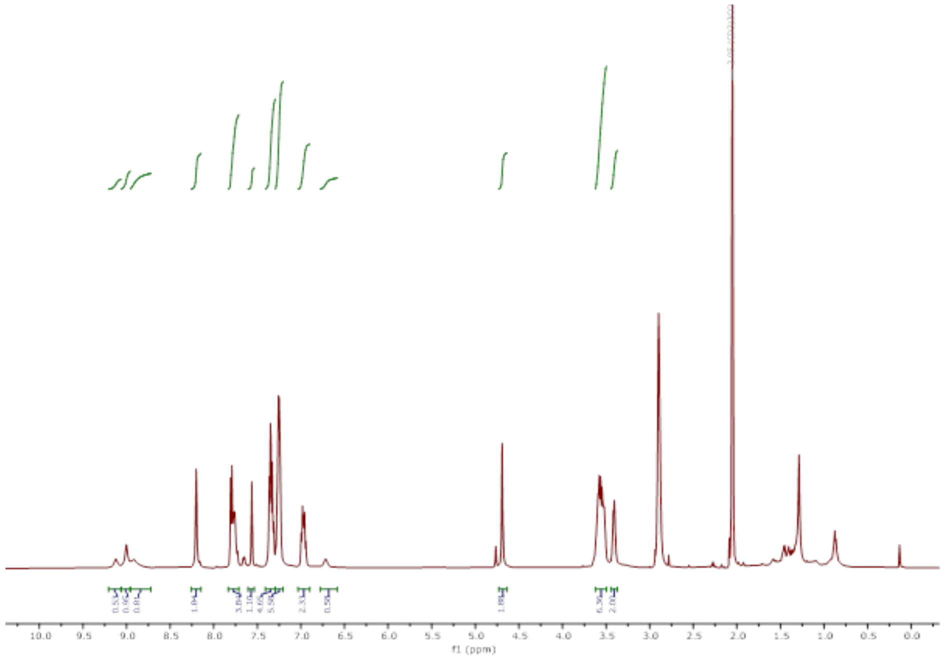

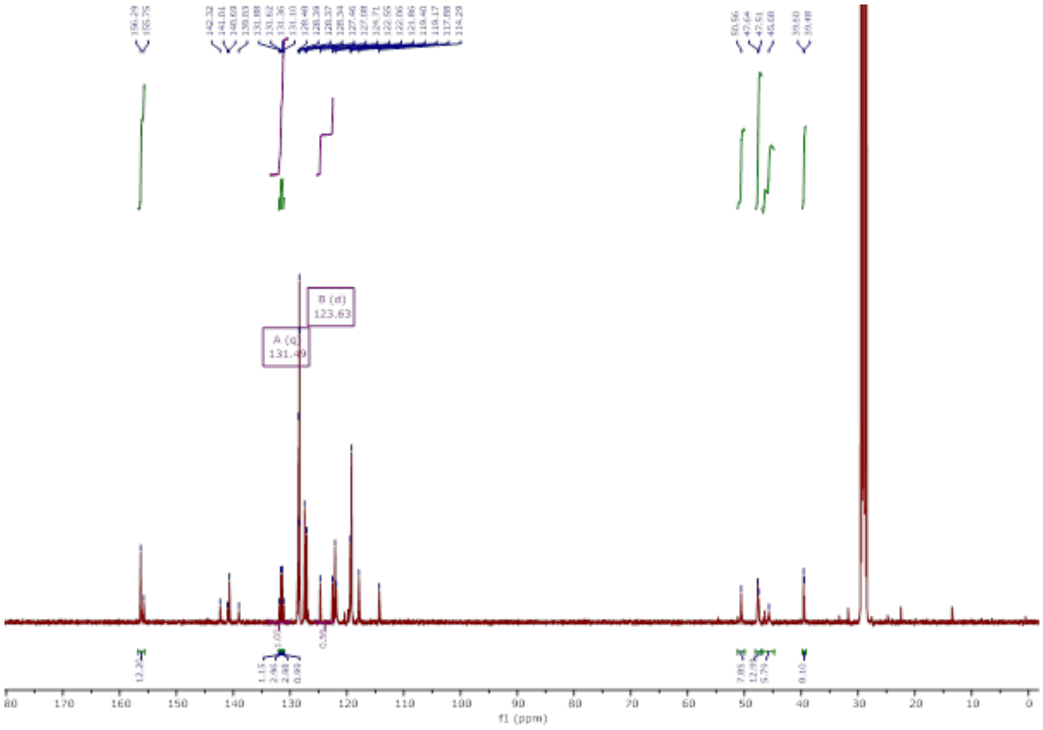

1e *N,N''-bis*(4-butoxyphenylcarbamoyl)-*N*-benzyl-*N'*-(3,5-*bis*(trifluoromethyl)phenylcarbamoyl) diethylenetriamine

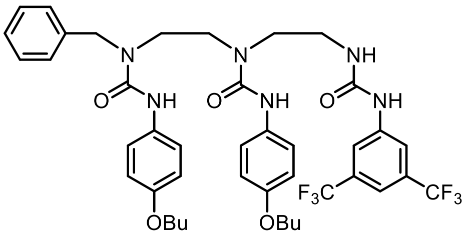

In $CDCl_3$

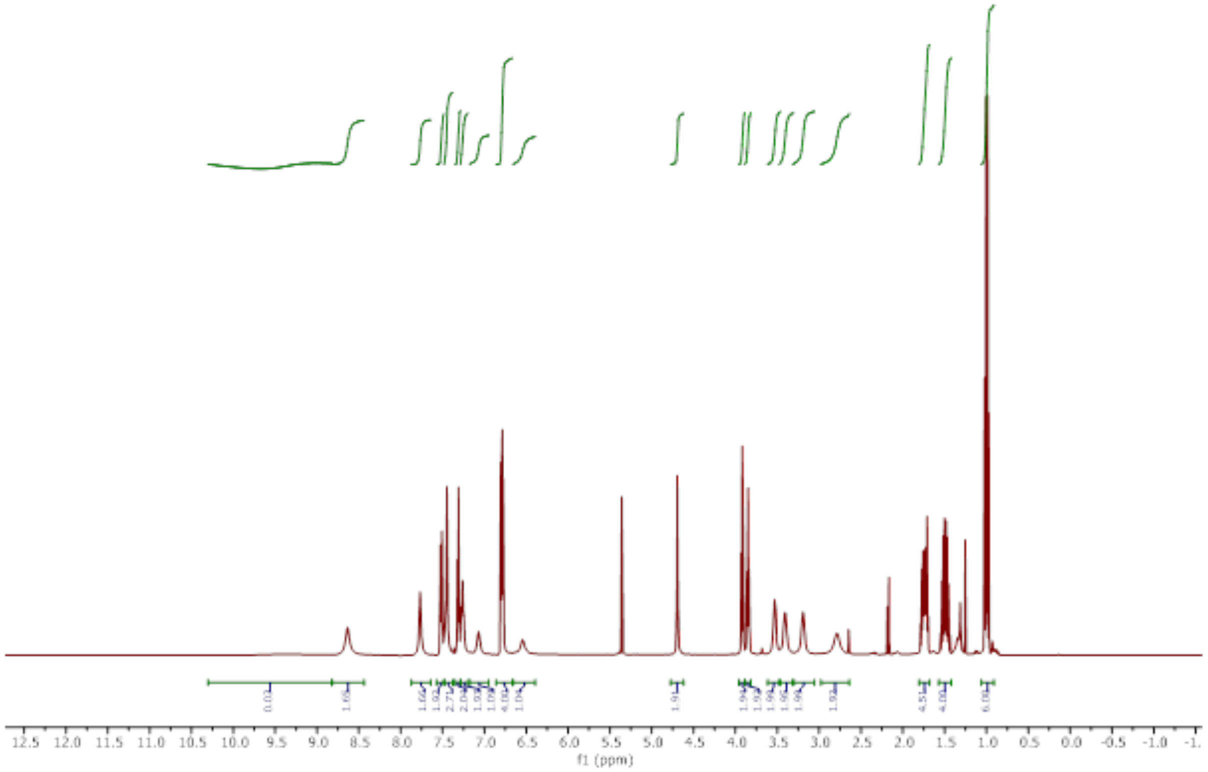

In CD_2Cl_2

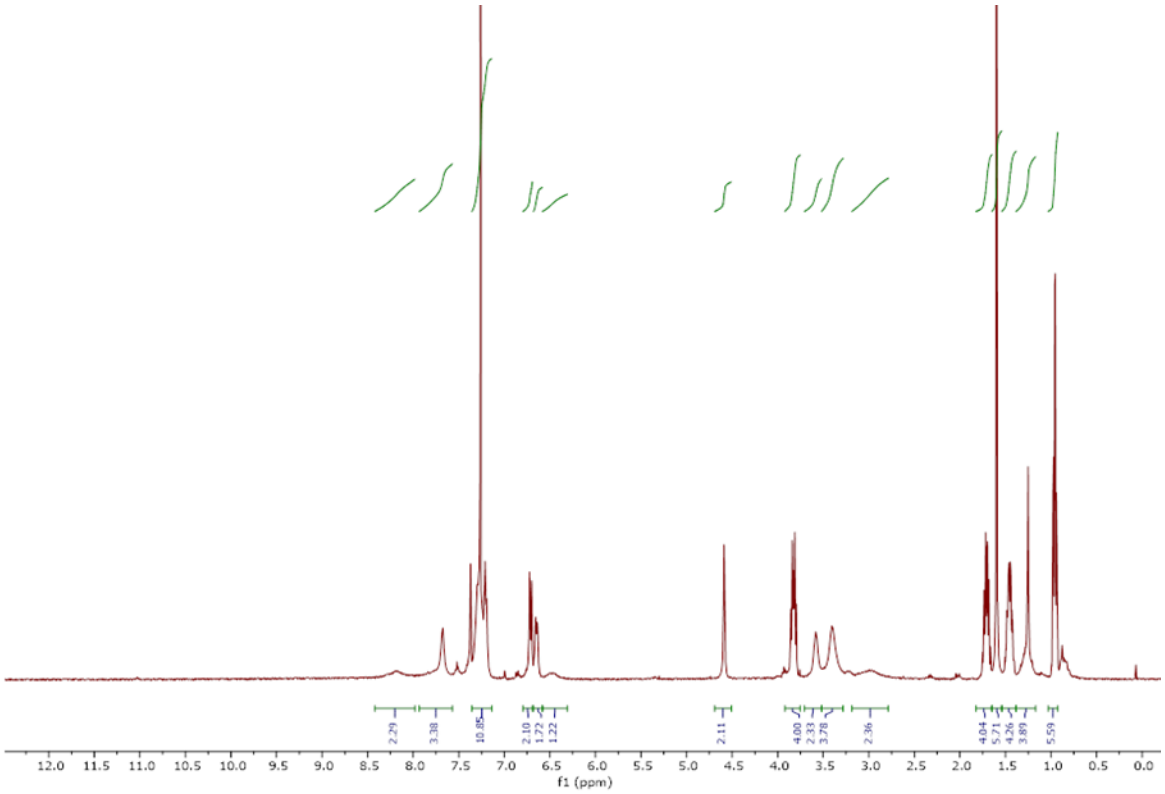

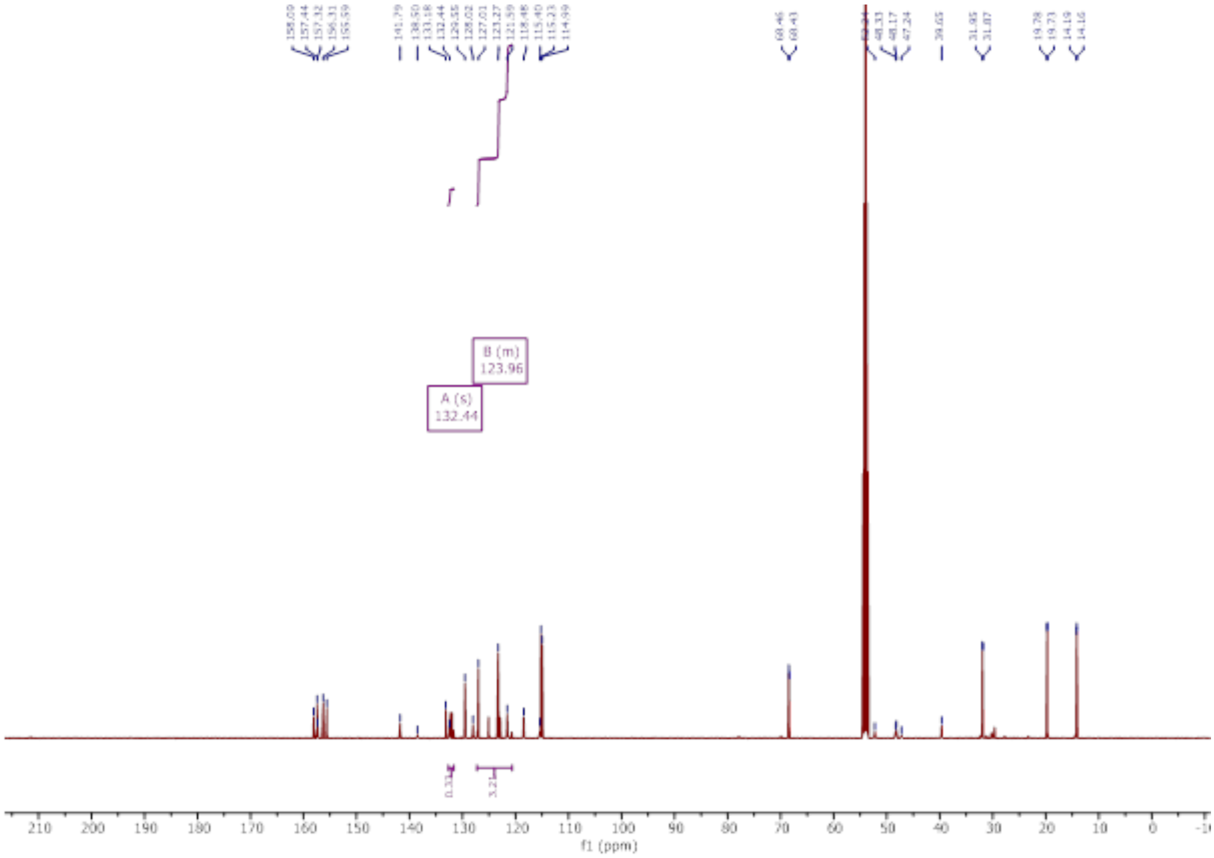

3a *N*-Ethyl-*N,N''*-*bis*(phenylcarbamoyl)-*N*'-(4-trifluoromethylphenylcarbamoyl)diethylenetriamine (400 MHz, CD_2Cl_2 +MeOH)

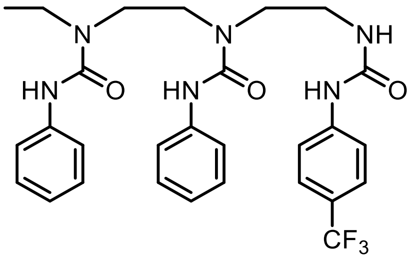

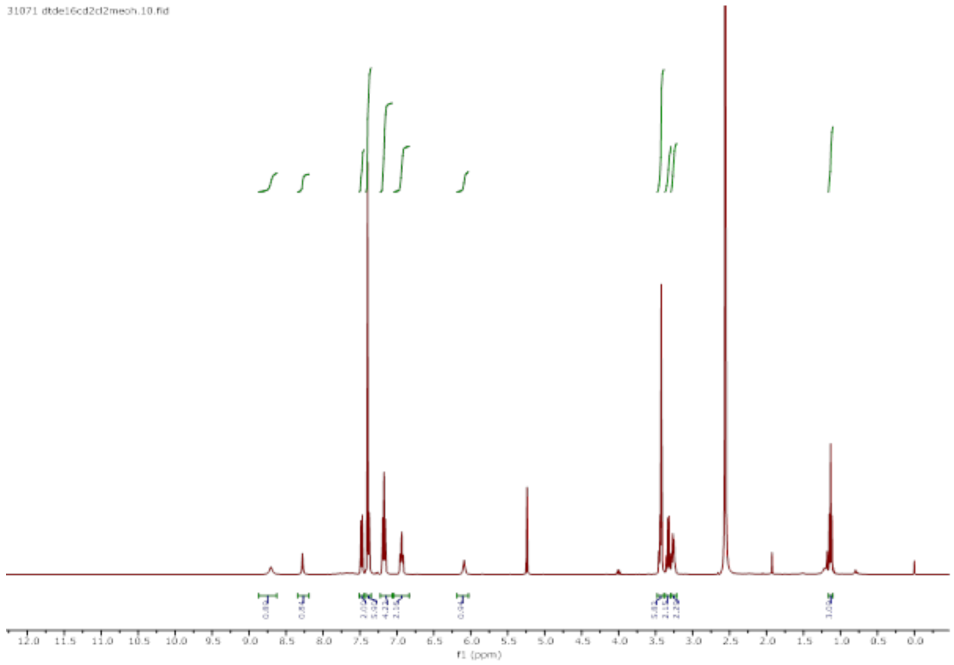

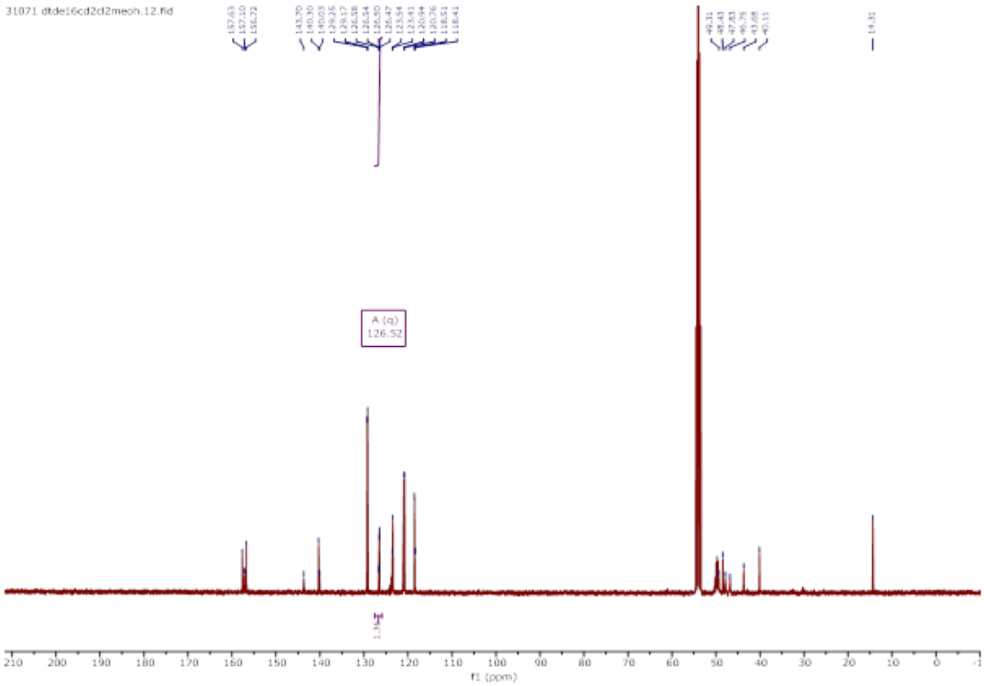

3b ***N*-Ethyl-*N,N''*-*bis*(phenylcarbamoyl)-*N'*-(3,5-*bis*(trifluoromethyl)phenylcarbamoyl)diethylenetriamine (400 MHz, DMSO d6)**

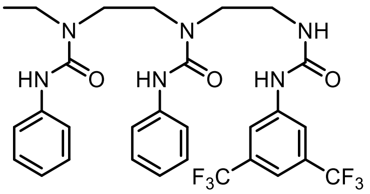

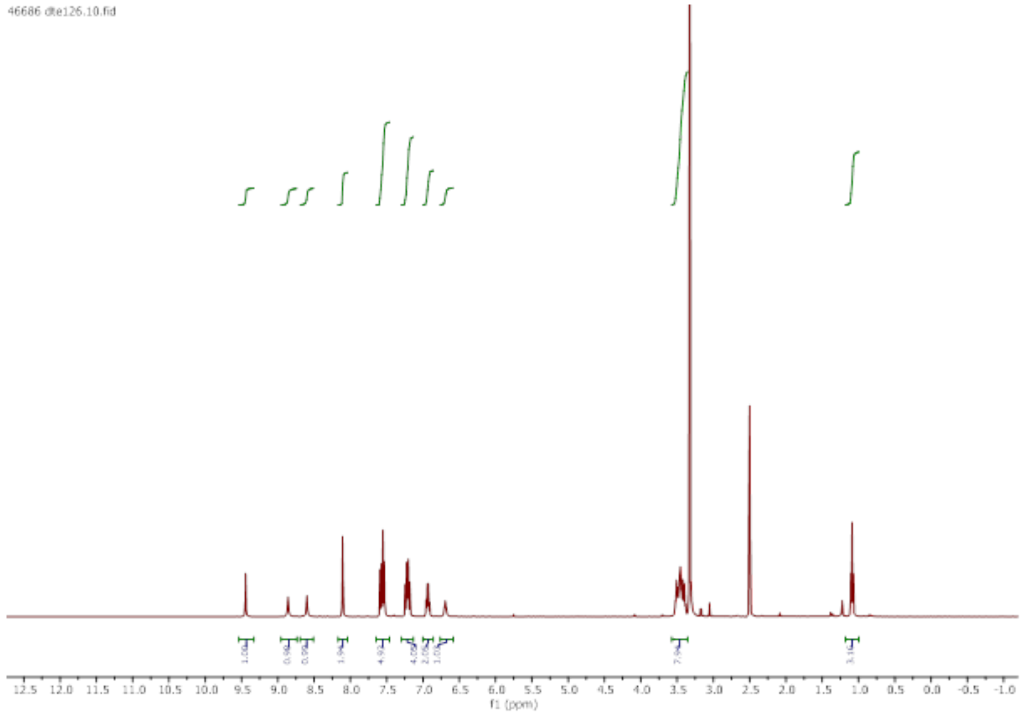

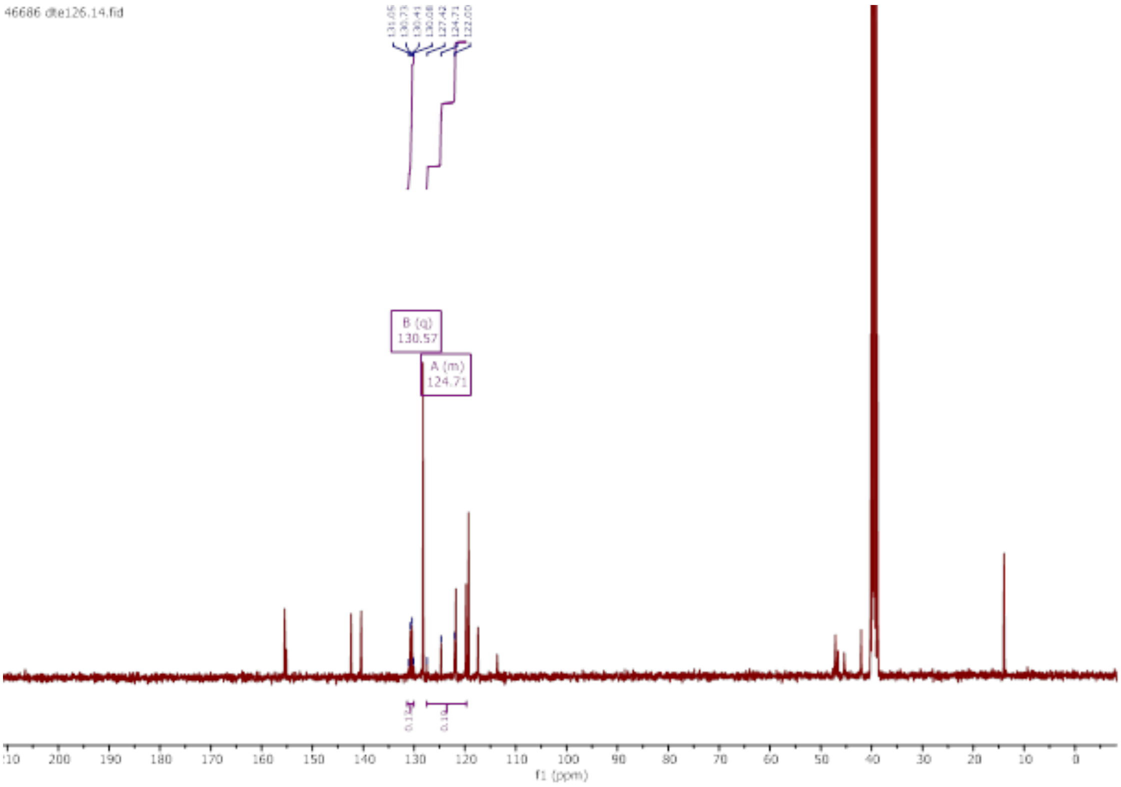

Compound 5ab: *N*,*N*''-*bis*(hexylcarbamoyl)-*N*-benzyl-*N*'-(tertbutoxycarbonyl)triethylenetetramine (400 MHz in $CDCl_3$)

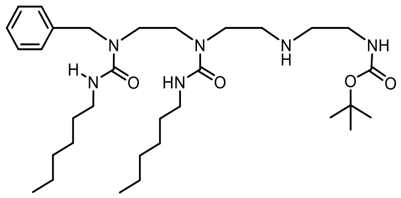

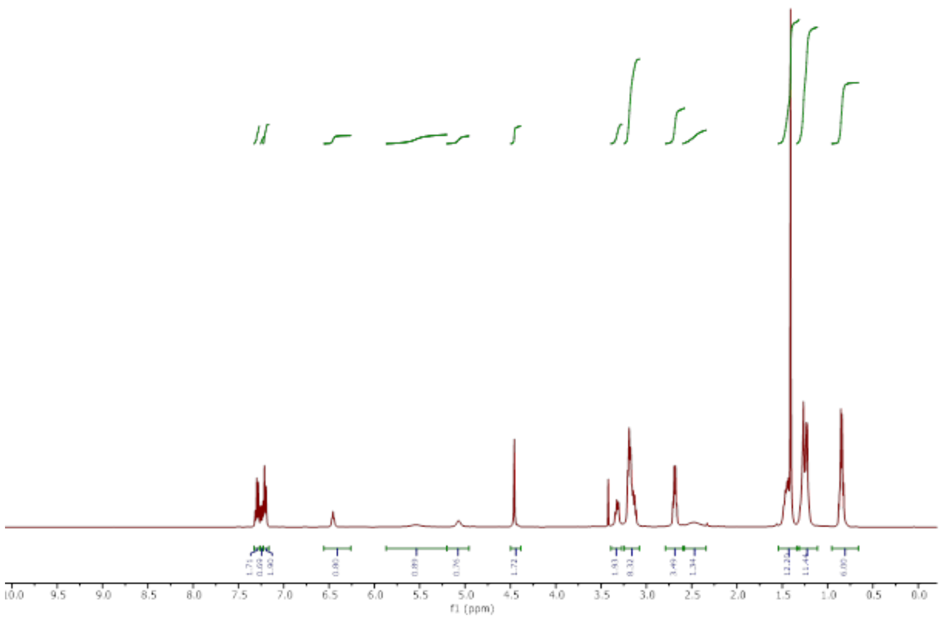

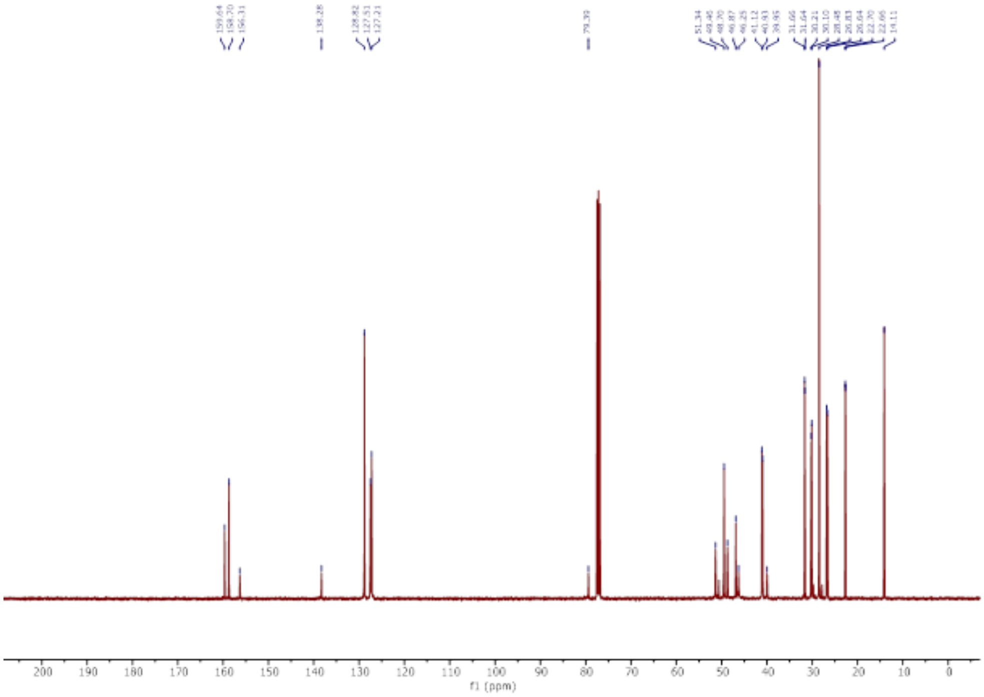

Compound 5ac: *N*,*N*'',*N*'''-tri(hexylcarbamoyl)-*N*-benzyl-*N*'-(*tert*butoxycarbonyl)triethylenetetramine (400 MHz in $CDCl_3$)

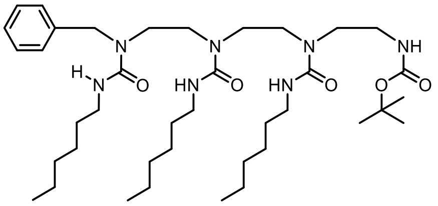

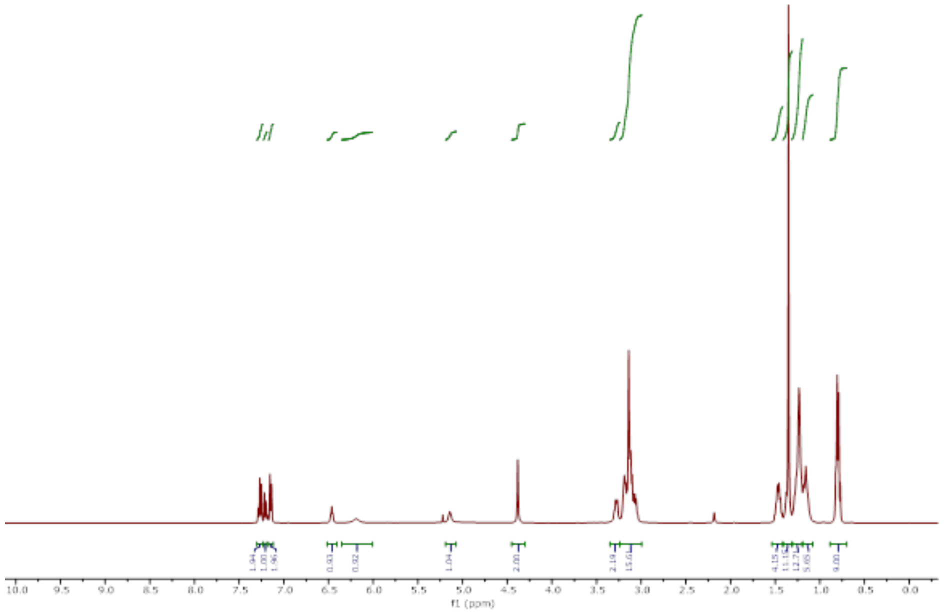

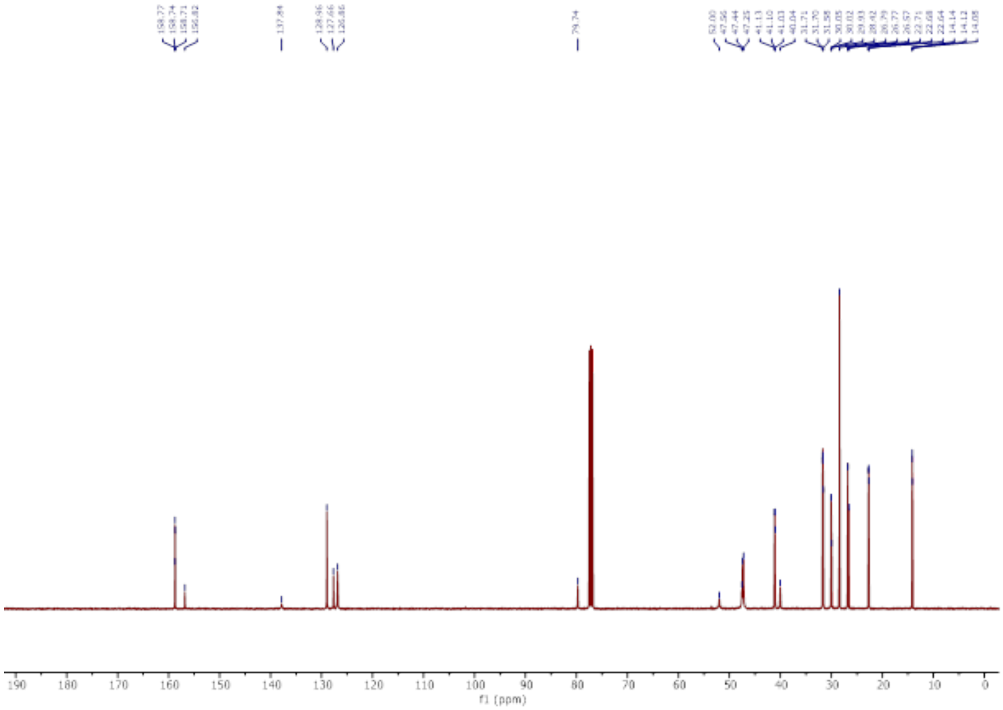

Compound 5bc: *N*'',*N*'''-*bis*(hexylcarbamoyl)-*N*-benzyl-*N*-(tertbutylcarbamoyl)-*N*'-(tertbutoxycarbonyl)triethylenetetramine (400 MHz in $CDCl_3$)

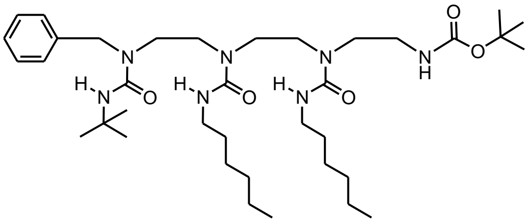

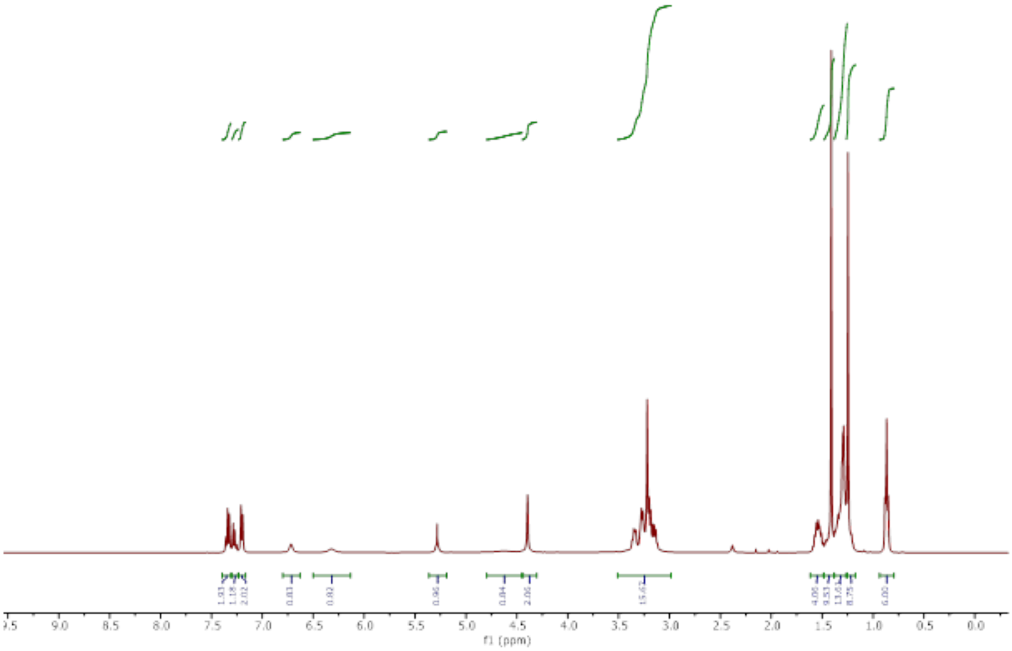

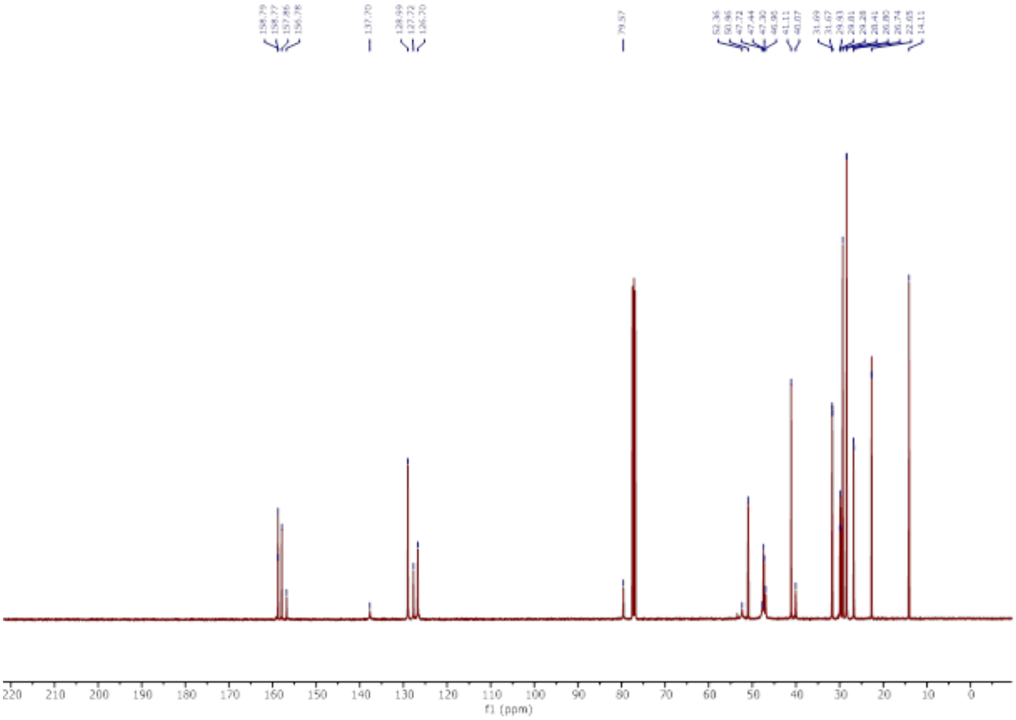

Compound 5ad: *N*-benzyl-*N*,*N*'',*N*'''-tri(hexylcarbamoyl)triethylenetetramine (400 MHz, $CDCl_3$)

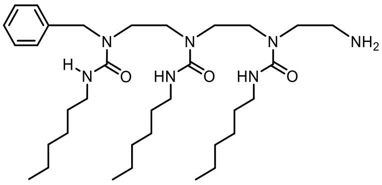

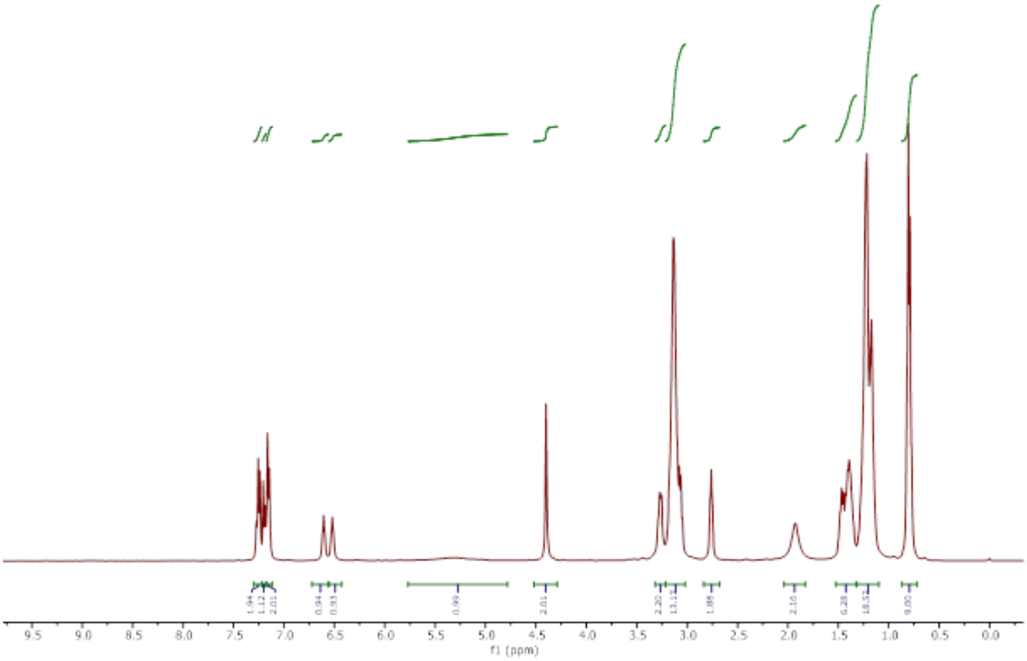

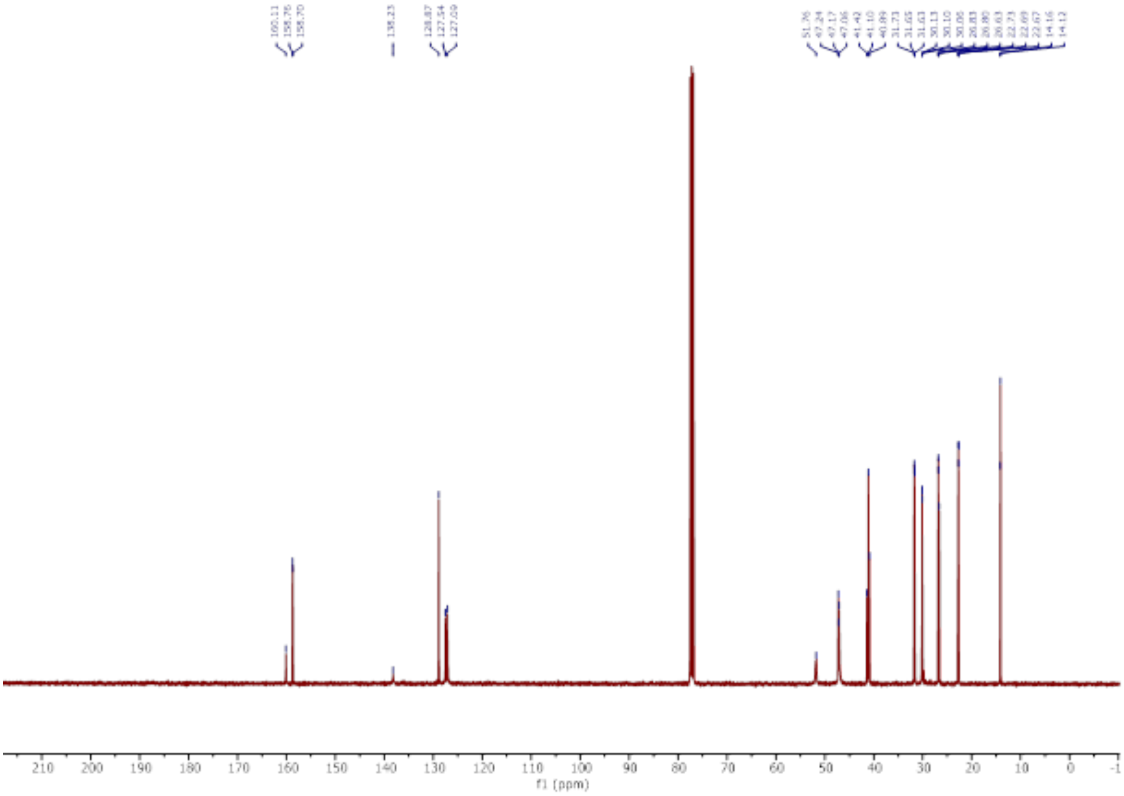

Compound 5bd: *N*-benzyl-*N*-(*tert*butylcarbamoyl)-*N*'',N’’’-*bis*(hexylcarbamoyl)triethylenetetramine (400 MHz, $CDCl_3$)

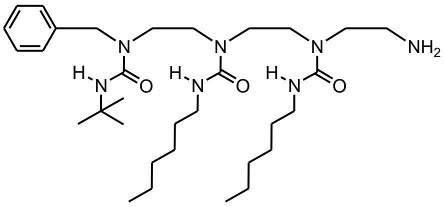

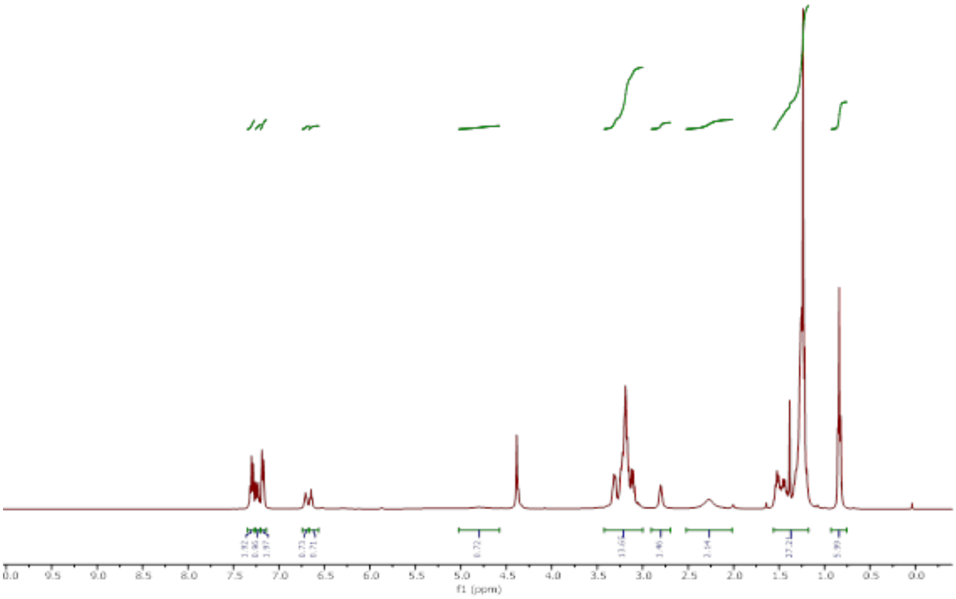

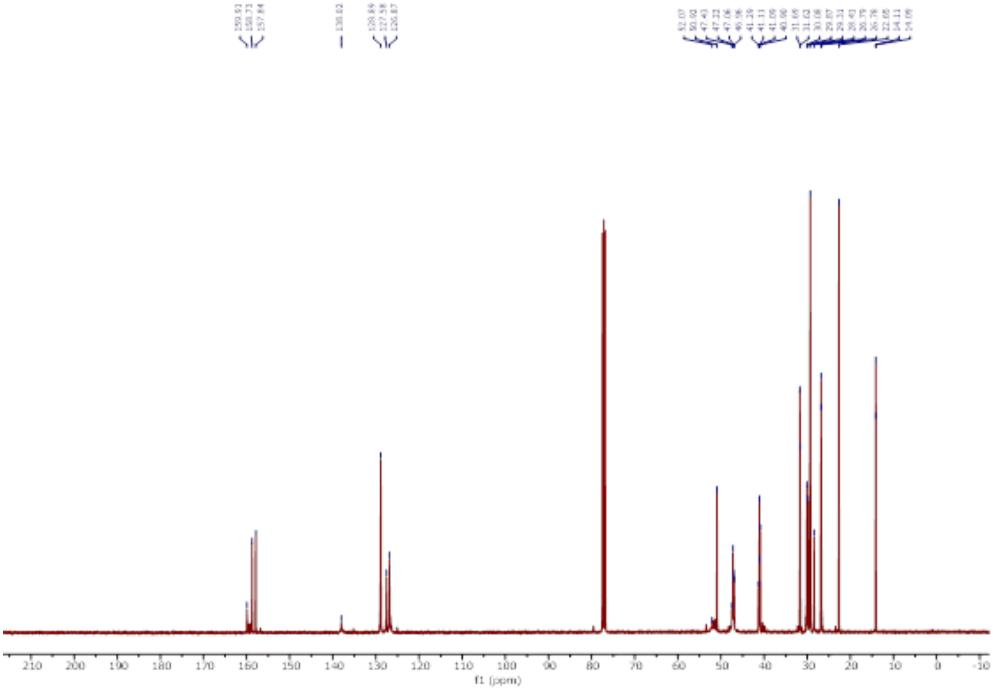

Compound 5a: *N*-benzyl-*N*,*N*'',N'''-tri(hexylcarbamoyl)-*N*'-(3,5-*bis*(trifluoromethyl)phenylcarbamoyl)triethylenetetramine (400 MHz in $CDCl_3$)

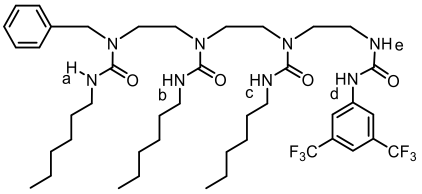

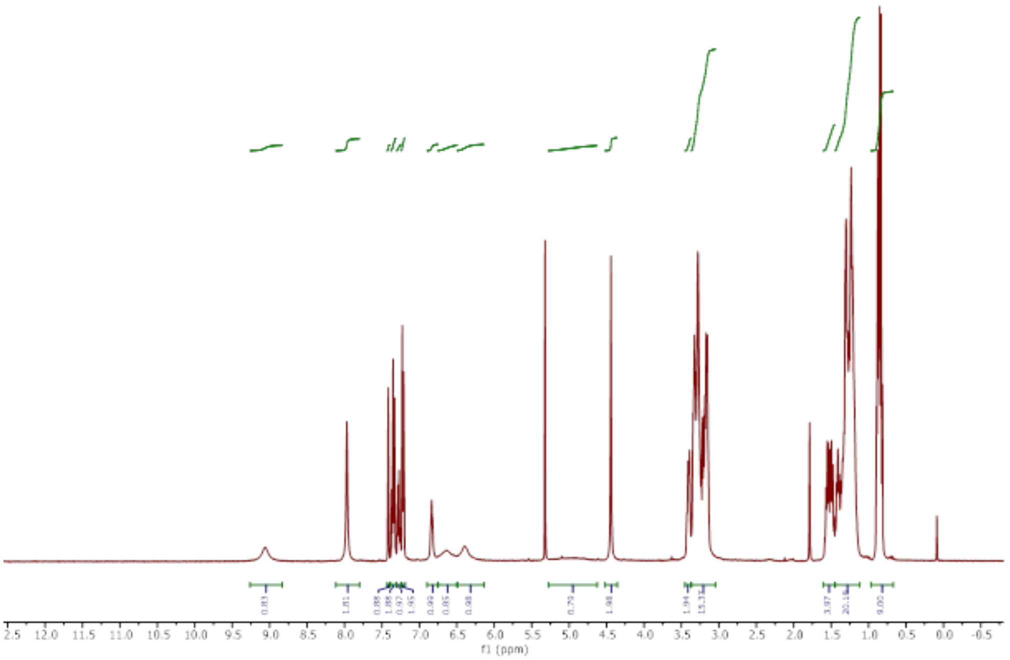

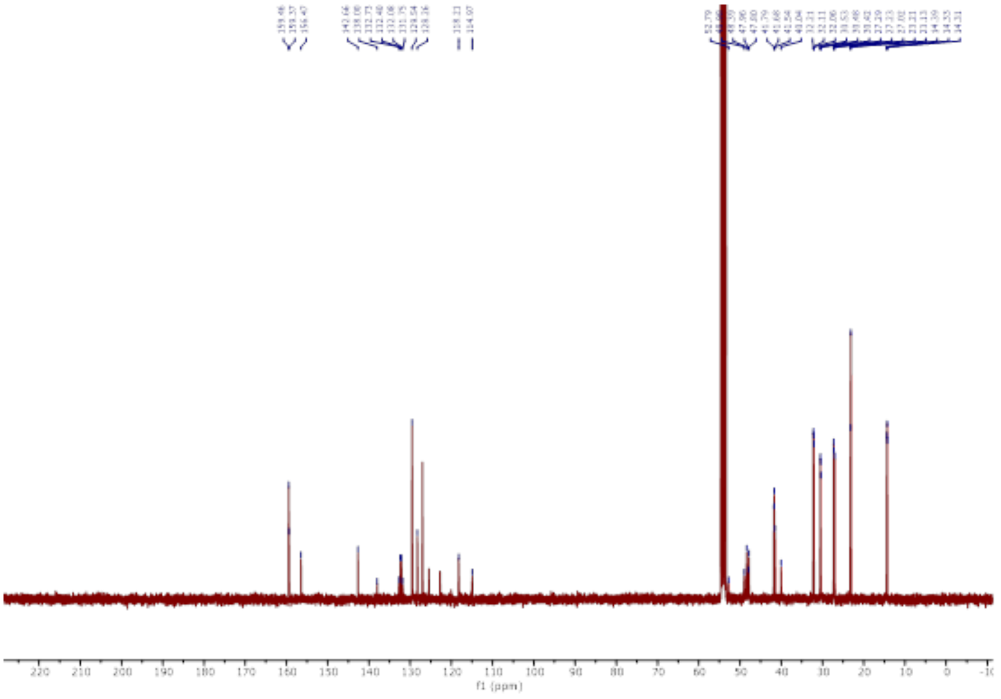

Compound 5b: *N*-benzyl-*N*-(*tert*butylcarbamoyl)-*N*'',*N*'''-*bis*(hexylcarbamoyl)-*N*'-(3,5-*bis*(trifluoromethyl)phenylcarbamoyl)triethylenetetramine (400 MHz in $CDCl_3$)

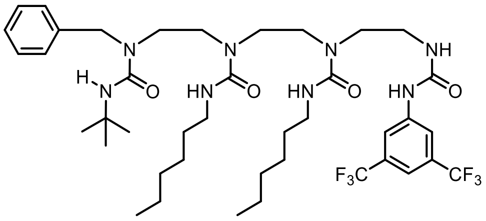

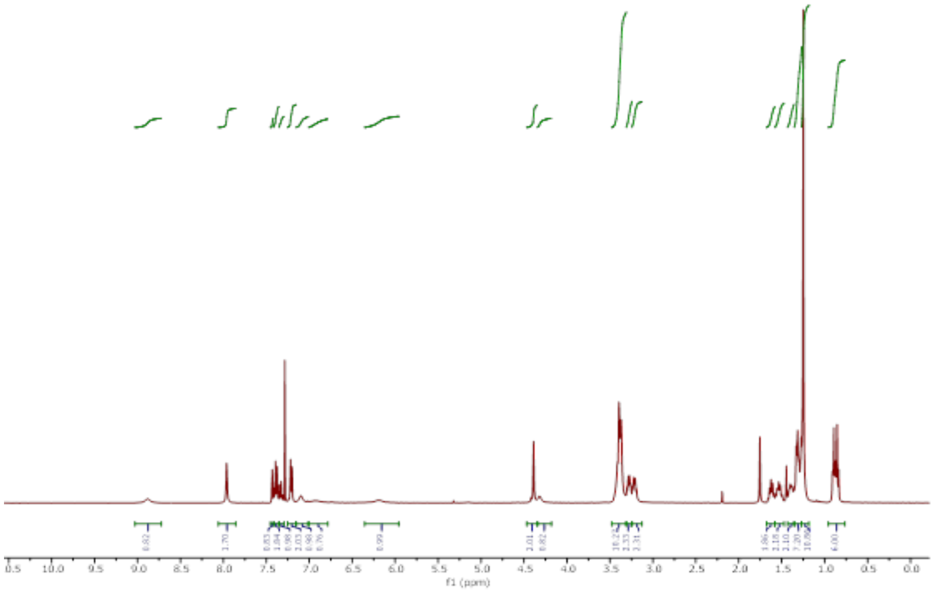

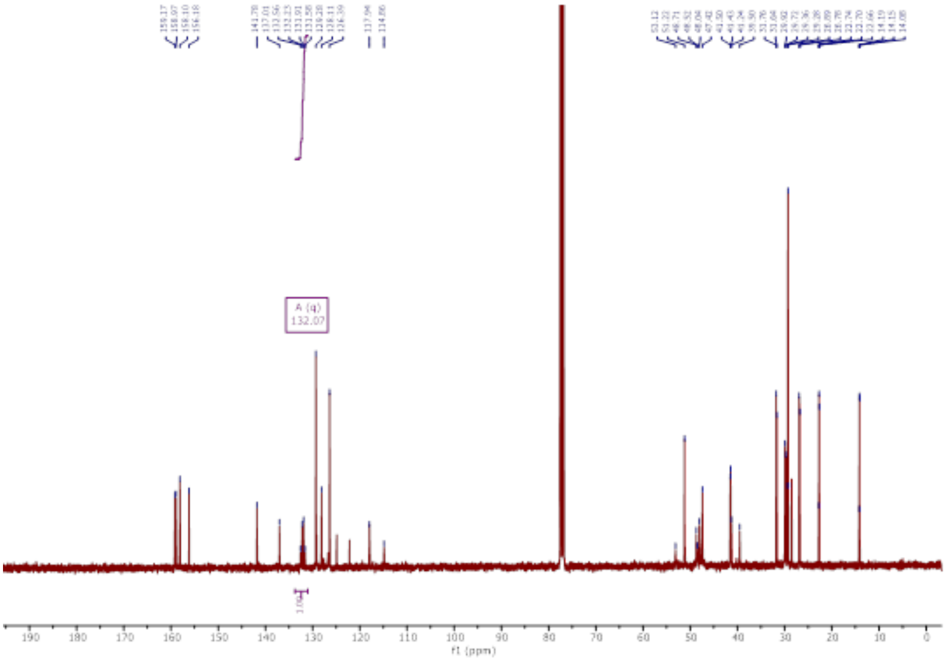

4. Crystal data and structure refinements

Single crystals suitable for X-ray diffraction analysis were grown by slow evaporation of an acetonitrile solution containing the compound. Data were collected on a dual source Rigaku FR-X rotating anode diffractometer using CuKα wavelength radiation (λ = 1.54184) or a Bruker Apex II Goniometer usine Mo-K_{α} radiation (λ = 0.717073 Å) at a temperature of 100(2) K, 150(2) K, 175(2) K or 200(2) K. Data for **1e**, **e136**, **f40** were found to be twinned with 2 components. Data were collected for compound **1a** at 100K using synchrotron radiation at beamline I19 at the Diamond Light Source with a Pilatus 2M detector.11 The data were either integrated using CrysAlisPro 1.71.40.14d and absorption correction was performed using empirical methods (SCALE3 ABSPACK) based upon symmetry-equivalent reflections combined with measurements at different azimuthal angles.10 or intensities were integrated using SAINT and absorption corrections based on equivalent reflections were applied using SADABS. Structures were solved using ShelXT, all of the structures were refined by full matrix least squares in ShelXL using Olex2. 11 All of the non hydrogen atoms were refined anisotropically. Hydrogen atoms were located geometrically and refined using a riding model. Disorder was modelled by setting the sum of the parts to 1 and refining the occupancies, restraints and constraints were applied to maintain sensible thermal and geometric parameters.

CCDC 2189961 (**1a**), 2189974 (**1b**), 2189976 (**1c**), 2189991 (**1d**), 2189996 (**1e**), 2189987 (**2a**), 2189988 (**2b**), 2189990 (**2c**), 2190005 (**3a**), 2189998 (**3b**), 2190003 (**4a**), 2190004 (**4b**), contain the supplementary crystallographic data for this paper. These data can be obtained free of charge via www.ccdc.cam.ac.uk/conts/retrieving.html (or from the Cambridge Crystallographic Data Centre, 12 Union Road, Cambridge CB2 1EZ, UK; fax: (+44)1223-336-033; or deposit@ccdc.cam.ac.uk).

1a

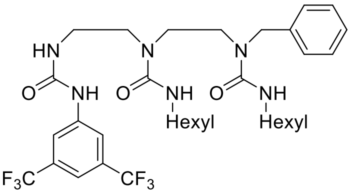

1b

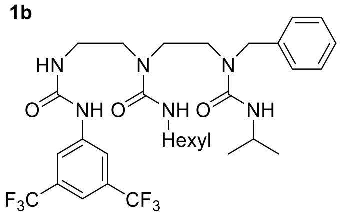

1c

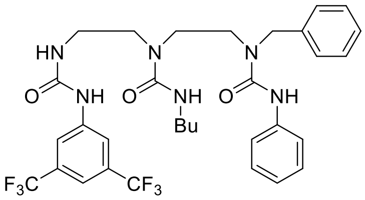

Identification code (**CCDC**)	**1a** (shelxt_a d69) (**2189961**)	**1b** (e67_bis)(**2189974**)	**1c** (s5812r g49)(**2189976**)
Empirical formula	$C_{34}H_{48}F_6N_6O_3$	$C_{31}H_{42}F_6N_6O_3$	$C_{32}H_{36}F_6N_6O_3$
Formula weight	702.78	660.70	666.67
Temperature/K	100(2)	100(2)	150(2)
Crystal system	triclinic	triclinic	triclinic
Space group	*P*-1	*P*-1	*P*-1
a/Å	13.5304(3)	14.5044(5)	14.5061(3)
b/Å	14.4783(3)	14.9547(5)	15.7206(4)
c/Å	19.2467(5)	17.2099(6)	17.2781(4)
α/°	86.952(1)	101.789(2)	92.442(1)
β/°	73.821(1)	111.249(2)	113.700(1)
γ/°	84.391(1)	97.465(2)	112.853(1)
Volume/Å^3	3602.39(16)	3319.6(2)	3231.16(13)
Z	4	4	4
ρ_{calc}g/cm^3	1.294	1.322	1.370
μ/mm^{-1}	0.891	0.109	0.970
F(000)	1488.0	1392.0	1392.0
Crystal size/mm^3	0.548 × 0.266 × 0.18	0.418 × 0.396 × 0.178	0.363 × 0.317 × 0.14
Radiation	Cu Kα (λ = 1.54184)	MoKα (λ = 0.71073)	Cu Kα (λ = 1.54184)
2θ range for data collection/	4.782 to 136.996	2.854 to 50.7	5.748 to 149.368
Index ranges	-16 ≤ h ≤ 16, -17 ≤ k ≤ 17, -23 ≤ l ≤ 23	-17 ≤ h ≤ 17, -18 ≤ k ≤ 17, -20 ≤ l ≤ 20	-18 ≤ h ≤ 18, -19 ≤ k ≤ 19, -21 ≤ l ≤ 21
Reflections collected	77150	48350	67927
Independent reflections	13215 [R_{int} = 0.0352, R_{sigma} = 0.0229]	12166 [R_{int} = 0.0572, R_{sigma} = 0.0526]	13186 [R_{int} = 0.0269, R_{sigma} = 0.0203]
Data/restraints/parameters	13215/66/1207	12166/328/949	13186/750/1053
Goodness-of-fit on F^2	1.022	1.010	1.020
Final R indexes [I>=2σ (I)]	R_1 = 0.0340, wR_2 = 0.0863	R_1 = 0.0528, wR_2 = 0.1276	R_1 = 0.0375, wR_2 = 0.0968
Final R indexes [all data]	R_1 = 0.0371, wR_2 = 0.0887	R_1 = 0.1011, wR_2 = 0.1565	R_1 = 0.0404, wR_2 = 0.0991
Largest diff. peak/hole / e Å^{-}	0.34/-0.28	0.66/-0.38	0.26/-0.28

Hydrogen Bonds for **1a** (2189961)

**D**	**H**	**A**	**d(D-H)/Å**	**d(H-A)/Å**	**d(D-A)/Å**	**D-H-A/°**
C6	H6	O1	0.949(15)	2.270(15)	2.8598(14)	119.6(11)
N1	H1	O6^1	0.870(17)	2.006(17)	2.8255(13)	156.6(14)
N2	H2A	O6^1	0.860(16)	2.050(16)	2.8386(13)	152.0(14)
N6	H6A	O2	0.865(17)	1.971(18)	2.8229(13)	167.9(15)
C40	H40	O4	0.952(15)	2.272(15)	2.8700(15)	120.1(11)

Hydrogen Bonds for **1a** (2189961)

D	H	A	d(D-H)/Å	d(H-A)/Å	d(D-A)/Å	D-H-A/°
N7	H7	O3	0.890(17)	1.828(17)	2.6844(13)	160.8(15)
N8	H8	O3	0.861(16)	2.079(16)	2.8410(13)	147.1(14)
N11	H11	O4	0.848(16)	2.085(16)	2.9247(13)	170.7(14)
N12	H12	O5	0.819(16)	2.145(17)	2.9529(14)	168.9(14)

Torsion Angles for **1a** (2189961).

A	B	C	D	Angle/˚	A	B	C	D	Angle/˚
C1	C2	C3	C4	-0.45(18)	C36	C35	N7	C43	176.01(11)
C1	C2	C3	C7	179.09(10)	C36	C37	C38	C39	-0.83(17)
C2	C1	C6	C5	-0.66(17)	C36	C37	C41	F7	59.5(4)
C2	C1	N1	C9	-168.97(11)	C36	C37	C41	F7A	38.1(8)
C2	C3	C4	C5	-0.50(17)	C36	C37	C41	F8	-61.8(4)
C2	C3	C7	F1	36.16(15)	C36	C37	C41	F8A	-78.8(7)
C2	C3	C7	F2	-83.52(13)	C36	C37	C41	F9	176.9(5)
C2	C3	C7	F3	156.15(11)	C36	C37	C41	F9A	162.5(7)
C3	C4	C5	C6	0.88(18)	C37	C38	C39	C40	0.80(17)
C3	C4	C5	C8	-177.71(11)	C37	C38	C39	C42	177.95(11)
C4	C3	C7	F1	-144.29(11)	C38	C37	C41	F7	-121.1(4)
C4	C3	C7	F2	96.03(13)	C38	C37	C41	F7A	-142.5(8)
C4	C3	C7	F3	-24.30(16)	C38	C37	C41	F8	117.6(4)
C4	C5	C6	C1	-0.30(18)	C38	C37	C41	F8A	100.6(7)
C4	C5	C8	F4	-63.71(14)	C38	C37	C41	F9	-3.7(5)
C4	C5	C8	F5	175.32(11)	C38	C37	C41	F9A	-18.1(7)
C4	C5	C8	F6	55.52(15)	C38	C39	C40	C35	0.27(17)
C6	C1	C2	C3	1.04(17)	C38	C39	C42	F10	12.39(17)
C6	C1	N1	C9	10.95(19)	C38	C39	C42	F11	-108.01(13)
C6	C5	C8	F4	117.65(12)	C38	C39	C42	F12	133.21(12)
C6	C5	C8	F5	-3.32(16)	C40	C35	C36	C37	1.29(17)
C6	C5	C8	F6	-123.12(12)	C40	C35	N7	C43	-4.60(19)
C7	C3	C4	C5	179.97(11)	C40	C39	C42	F10	-170.33(11)
C8	C5	C6	C1	178.28(11)	C40	C39	C42	F11	69.27(15)
C10	C11	N3	C12	93.45(12)	C40	C39	C42	F12	-49.51(15)
C10	C11	N3	C21	-79.25(14)	C41	C37	C38	C39	179.81(11)
C11	C10	N2	C9	-72.48(14)	C42	C39	C40	C35	-176.96(11)
C12	C13	N4	C14	-109.79(12)	C44	C45	N9	C46	-100.63(12)
C12	C13	N4	C28	72.51(14)	C44	C45	N9	C55	72.52(14)
C13	C12	N3	C11	-103.13(12)	C45	C44	N8	C43	69.84(14)
C13	C12	N3	C21	69.94(13)	C46	C47	N10	C48	88.58(12)
C14	C15	C16	C17	178.97(12)	C46	C47	N10	C62	-86.82(13)
C14	C15	C20	C19	-178.53(12)	C47	C46	N9	C45	95.37(12)
C15	C14	N4	C13	102.79(12)	C47	C46	N9	C55	-78.17(13)
C15	C14	N4	C28	-79.40(13)	C48	C49	C50	C51	178.52(12)

Torsion Angles for **1a** (2189961).

A	B	C	D	Angle/˚	A	B	C	D	Angle/˚
C15	C16	C17	C18	-0.1(2)	C48	C49	C54	C53	-178.86(11)
C16	C15	C20	C19	0.99(19)	C49	C48	N10	C47	80.24(13)
C16	C17	C18	C19	0.4(2)	C49	C48	N10	C62	-104.23(12)
C17	C18	C19	C20	0.1(2)	C49	C50	C51	C52	0.4(2)
C18	C19	C20	C15	-0.7(2)	C50	C49	C54	C53	-0.35(18)
C20	C15	C16	C17	-0.57(19)	C50	C51	C52	C53	-0.5(2)
C22	C23	C24	C25	177.54(11)	C51	C52	C53	C54	0.2(2)
C23	C22	N5	C21	151.98(11)	C52	C53	C54	C49	0.23(19)
C23	C24	C25	C26	176.89(11)	C54	C49	C50	C51	0.03(18)
C24	C25	C26	C27	175.64(13)	C56	C57	C58	C59	172.77(11)
C29	C30	C31	C32	-60.17(15)	C57	C56	N11	C55	-157.51(11)
C30	C29	N6	C28	119.62(12)	C57	C58	C59	C60	60.04(17)
C30	C31	C32	C33	176.79(11)	C58	C59	C60	C61	170.97(14)
C31	C32	C33	C34	-177.10(11)	C63	C64	C65	C66	62.65(16)
N1	C1	C2	C3	-179.04(11)	C64	C63	N12	C62	159.65(11)
N1	C1	C6	C5	179.42(11)	C64	C65	C66	C67	178.54(11)
N1	C9	N2	C10	178.53(10)	C65	C66	C67	C68	-67.31(16)
N2	C9	N1	C1	178.13(11)	N7	C35	C36	C37	-179.29(10)
N2	C10	C11	N3	162.57(10)	N7	C35	C40	C39	179.33(11)
N3	C12	C13	N4	-159.81(10)	N7	C43	N8	C44	179.28(10)
N3	C21	N5	C22	176.47(11)	N8	C43	N7	C35	177.29(11)
N4	C14	C15	C16	-178.24(11)	N8	C44	C45	N9	-159.46(10)
N4	C14	C15	C20	1.27(16)	N9	C46	C47	N10	160.17(9)
N4	C28	N6	C29	-170.76(11)	N9	C55	N11	C56	-171.75(10)
N5	C21	N3	C11	-2.08(17)	N10	C48	C49	C50	38.74(15)
N5	C21	N3	C12	-174.95(10)	N10	C48	C49	C54	-142.79(11)
N5	C22	C23	C24	-179.13(10)	N10	C62	N12	C63	179.63(10)
N6	C28	N4	C13	-1.31(17)	N11	C55	N9	C45	4.99(16)
N6	C28	N4	C14	-179.09(10)	N11	C55	N9	C46	178.35(10)
N6	C29	C30	C31	-59.19(14)	N11	C56	C57	C58	-178.44(11)
O1	C9	N1	C1	-1.1(2)	N12	C62	N10	C47	4.89(16)
O1	C9	N2	C10	-2.26(18)	N12	C62	N10	C48	-170.40(10)
O2	C21	N3	C11	179.31(11)	N12	C63	C64	C65	170.01(11)
O2	C21	N3	C12	6.43(16)	O4	C43	N7	C35	-1.76(19)
O2	C21	N5	C22	-4.93(18)	O4	C43	N8	C44	-1.67(18)
O3	C28	N4	C13	179.43(10)	O5	C55	N9	C45	-176.38(10)
O3	C28	N4	C14	1.65(16)	O5	C55	N9	C46	-3.01(16)
O3	C28	N6	C29	8.47(18)	O5	C55	N11	C56	9.65(17)
C35	C36	C37	C38	-0.21(17)	O6	C62	N10	C47	-172.78(10)
C35	C36	C37	C41	179.18(10)	O6	C62	N10	C48	11.93(16)
C36	C35	C40	C39	-1.31(17)	O6	C62	N12	C63	-2.73(18)

Hydrogen Bonds for 2189974 (compound **1b**)

D	H	A	d(D-H)/Å	d(H-A)/Å	d(D-A)/Å	D-H-A/°
N5	H5a	O4	0.8800	2.064(3)	2.852(3)	148.69(8)
N5	H5b	O4	0.8800	2.110(3)	2.852(3)	141.59(8)
C29	H29	O3	0.9500	2.241(3)	2.850(3)	121.05(9)
C60	H60	O6	0.9500	2.250(3)	2.864(3)	121.55(8)
N7	H7a	O5	0.79(3)	2.04(3)	2.816(3)	170(3)
N1	H1	O2	0.78(3)	2.09(3)	2.848(3)	164(3)
N12	H12	O1[1]	0.87(3)	1.96(3)	2.794(3)	160(3)
N6	H6	O4	0.80(3)	2.07(3)	2.817(3)	156(3)
N11	H11a	O1[1]	0.84(3)	2.11(3)	2.878(3)	153(3)

Torsion Angles for 2189974 (compound **1b**)

A	B	C	D	Angle/˚	A	B	C	D	Angle/˚
F1	C30	C26	C25	55.7(3)	F8	C61	C57	C58	109.0(3)
F1	C30	C26	C27	-122.6(2)	F9	C61	C57	C56	170.3(2)
F2	C30	C26	C25	-61.8(3)	F9	C61	C57	C58	-11.9(3)
F2	C30	C26	C27	119.8(2)	F10	C62	C59	C58	-13.5(4)
F3	C30	C26	C25	176.2(3)	F10	C62	C59	C60	165.4(3)
F3	C30	C26	C27	-2.1(3)	F11	C62	C59	C58	-134.2(3)
F4	C31	C28	C27	152.9(2)	F11	C62	C59	C60	44.7(3)
F4	C31	C28	C29	-28.3(3)	F12	C62	C59	C58	110.9(3)
F5	C31	C28	C27	31.1(3)	F12	C62	C59	C60	-70.2(3)
F5	C31	C28	C29	-150.0(2)	O4	C35	N7	C32	-5.9(4)
F6	C31	C28	C27	-87.5(3)	O4	C35	N8	C36	-21.9(4)
F6	C31	C28	C29	91.4(3)	O4	C35	N8	C43	166.9(3)
O1	C4	N1	C1	5.7(4)	O4	C35	N8A	C36	26.2(4)
O1	C4	N2	C5	18.5(3)	O4	C35	N8A	C43A	-163.6(13)
O1	C4	N2	C12	-168.5(3)	O5	C45	N9	C44	1.4(4)
O1	C4	N2A	C5	-27.2(3)	O5	C45	N9	C52	173.0(3)
O1	C4	N2A	C12A	165.3(6)	O5	C45	N10	C46	-1.0(4)
O2	C14	N3	C13	3.0(4)	O5	C45	N9A	C52	-161.1(10)
O2	C14	N3	C21	-172.6(4)	O5	C45	N9A	C44A	5.5(8)
O2	C14	N4	C15	-5.6(4)	O6	C54	N11	C53	0.6(3)
O2	C14	N3A	C13A	3.7(5)	O6	C54	N12	C55	12.7(3)
O2	C14	N3A	C21A	-179.2(6)	N7	C35	N8	C36	161.6(3)
O3	C23	N5	C22	8.2(4)	N7	C35	N8	C43	-9.6(4)
O3	C23	N5	C22A	-14.5(7)	N7	C35	N8A	C36	-172.9(11)
O3	C23	N6	C24	-5.1(4)	N7	C35	N8A	C43A	-2.7(9)
N1	C4	N2	C5	-168.0(3)	N8	C35	N8A	C36	-79.4(17)
N1	C4	N2	C12	5.0(4)	N8	C35	N8A	C43A	90.9(16)
N1	C4	N2A	C5	169.2(5)	N8	C36	C37	C38	-16.5(3)
N1	C4	N2A	C12A	1.7(5)	N8	C36	C37	C42	164.5(2)
N2	C4	N2A	C5	68.4(14)	N8	C36	N8A	C35	76.0(18)

Torsion Angles for 2189974 (compound **1b**)

A	B	C	D	Angle/˚	A	B	C	D	Angle/˚
N2	C4	N2A	C12A	-99.0(13)	N8	C36	N8A	C43A	-95.0(15)
N2	C5	C6	C7	-26.5(4)	N8	C43	C44	N9	-153.7(3)
N2	C5	C6	C11	154.2(3)	N9	C45	N10	C46	-175.0(3)
N2	C5	N2A	C4	-68.1(15)	N9	C45	N9A	C52	-65.8(16)
N2	C5	N2A	C12A	99.2(13)	N9	C45	N9A	C44A	100.9(13)
N2	C12	C13	N3	157.8(4)	N9	C52	C53	N11	161.7(3)
N3	C14	N4	C15	167.3(3)	N9	C52	N9A	C45	63.3(18)
N3	C14	N3A	C13A	-90.6(9)	N9	C52	N9A	C44A	-100.6(15)
N3	C14	N3A	C21A	86.6(9)	N10	C45	N9A	C52	39.0(4)
N3	C21	C22	N5	161.5(4)	N10	C45	N9A	C44A	-154.3(11)
N4	C14	N3A	C13A	161.0(5)	N10	C46	C47	C48	-61.3(3)
N4	C14	N3A	C21A	-21.8(6)	N11	C53	C52	N9A	163.9(5)
N4	C15	C16	C17	-71.4(3)	N11	C54	N12	C55	-167.3(2)
N5	C23	N6	C24	175.1(2)	N12	C55	C56	C57	-176.2(2)
N5	C22A	C21A	N3A	157.2(10)	N12	C55	C60	C59	176.2(3)
N6	C24	C25	C26	178.1(2)	C35	N8A	C36	C37	68(2)
N6	C24	C29	C28	-178.3(3)	C35	N8A	C43A	C44A	-68(2)
C4	N2A	C5	C6	117.2(7)	C36	C37	C38	C39	-179.9(3)
C4	N2A	C12A	C13A	78.6(9)	C36	C37	C42	C41	179.0(3)
C5	C6	C7	C8	-179.3(3)	C36	N8A	C43A	C44A	103.2(17)
C5	C6	C11	C10	179.6(2)	C37	C38	C39	C40	1.1(3)
C5	N2A	C12A	C13A	-87.8(9)	C37	C42	C41	C40	0.8(3)
C6	C7	C8	C9	-0.3(3)	C38	C39	C40	C41	-0.2(4)
C6	C11	C10	C9	-0.3(3)	C39	C40	C41	C42	-0.7(4)
C7	C8	C9	C10	0.3(4)	C45	N9A	C52	C53	-111.6(13)
C8	C9	C10	C11	-0.0(4)	C45	N9A	C44A	C43A	-76.2(15)
C14	N3A	C13A	C12A	67.3(8)	C46	C47	C48	C49	-172.2(3)
C14	N3A	C21A	C22A	-67.2(10)	C47	C48	C49	C50	-177.2(3)
C15	C16	C17	C18	175.7(2)	C48	C49	C50	C51	-178.1(3)
C16	C17	C18	C19	162.0(3)	C52	N9A	C44A	C43A	88.2(19)
C17	C18	C19	C20	174.2(3)	C55	C56	C57	C58	-0.7(3)
C24	C25	C26	C27	0.2(3)	C55	C56	C57	C61	177.1(3)
C24	C25	C26	C30	-178.2(3)	C55	C60	C59	C58	0.5(3)
C24	C29	C28	C27	0.3(3)	C55	C60	C59	C62	-178.3(3)
C24	C29	C28	C31	-178.5(3)	C56	C57	C58	C59	-0.3(3)
C25	C26	C27	C28	0.9(3)	C57	C58	C59	C60	0.4(3)
C26	C27	C28	C29	-1.2(3)	C57	C58	C59	C62	179.2(3)
C26	C27	C28	C31	177.7(3)	N2A	C12A	C13A	N3A	-161.1(7)
F7	C61	C57	C56	50.6(3)	C12A	C13A	N3A	C21A	-109.9(8)
F7	C61	C57	C58	-131.6(3)	C13A	N3A	C21A	C22A	109.8(10)
F8	C61	C57	C56	-68.8(3)					

Hydrogen Bonds for 2189976 (**1c**)

D	H	A	d(D-H)/Å	d(H-A)/Å	d(D-A)/Å	D-H-A/°
N1	H1	O6	0.852(18)	2.188(18)	2.9546(14)	149.5(15)
N2	H2A	O6	0.845(19)	1.962(19)	2.7691(14)	159.2(16)
N5	H5	O1	0.864(18)	2.136(18)	2.9920(13)	171.0(16)
N6	H6A	O2	0.850(16)	1.966(17)	2.8026(13)	167.9(14)
N7	H7	O3[1]	0.847(18)	2.351(18)	3.0759(13)	143.9(15)
N8	H8	O3[1]	0.88	2.11	2.909(4)	149.7
N8A	H8A	O3[1]	0.88	2.05	2.877(7)	155.6
N8B	H8B	O3[1]	0.88	1.92	2.768(7)	161.9
N11	H11	O4	0.877(19)	2.027(18)	2.8946(14)	170.2(16)
N12	H12	O5	0.880(18)	1.91(3)	2.782(18)	168.2(16)
N12	H12	O5A	0.880(18)	1.916(19)	2.773(6)	164.4(15)
N12	H12	O5B	0.880(18)	1.91(3)	2.786(19)	173.0(17)

Torsion Angles for 2189976 (compound **1c**)

A	B	C	D	Angle/˚	A	B	C	D	Angle/˚
C1	C2	C3	C4	-0.3(2)	C42B	C43B	N9B	C44B	-101.9(7)
C1	C2	C3	C7	-178.50(12)	C42B	C43B	N9B	C53B	44.7(9)
C2	C1	C6	C5	2.07(19)	C43	C42	N8	C41	-67.2(5)
C2	C1	N1	C9	-28.2(2)	C43A	C42A	N8A	C41	67.7(8)
C2	C3	C4	C5	1.44(19)	C43B	C42B	N8B	C41	69.6(8)
C2	C3	C7	F1	-46.47(17)	C44	C45	N10	C46	-81.8(6)
C2	C3	C7	F2	-168.02(12)	C44	C45	N10	C58	79.8(6)
C2	C3	C7	F3	73.06(15)	C44A	C45A	N10A	C46	-78.8(7)
C3	C4	C5	C6	-0.81(19)	C44A	C45A	N10A	C58	93.6(6)
C3	C4	C5	C8	179.90(12)	C44B	C45B	N10B	C46	104.5(4)
C4	C3	C7	F1	135.31(13)	C44B	C45B	N10B	C58	-79.2(5)
C4	C3	C7	F2	13.76(18)	C45	C44	N9	C43	-116.2(5)
C4	C3	C7	F3	-105.17(14)	C45	C44	N9	C53	77.5(6)
C4	C5	C6	C1	-0.94(19)	C45A	C44A	N9A	C43A	-84.9(7)
C4	C5	C8	F4	38.94(16)	C45A	C44A	N9A	C53A	67.1(7)
C4	C5	C8	F5	-80.37(15)	C45B	C44B	N9B	C43B	90.3(5)
C4	C5	C8	F6	159.03(12)	C45B	C44B	N9B	C53B	-55.3(6)
C6	C1	C2	C3	-1.46(19)	C46	C47	C48	C49	175.93(12)
C6	C1	N1	C9	151.77(12)	C46	C47	C52	C51	-176.26(12)
C6	C5	C8	F4	-140.36(12)	C47	C46	N10	C45	-94.6(4)
C6	C5	C8	F5	100.33(14)	C47	C46	N10	C58	102.49(16)
C6	C5	C8	F6	-20.27(17)	C47	C46	N10A	C45A	-84.7(3)
C7	C3	C4	C5	179.59(12)	C47	C46	N10A	C58	102.49(16)
C8	C5	C6	C1	178.34(12)	C47	C46	N10B	C45B	-107.2(4)
C10	C11	N3	C12	-103.02(12)	C47	C46	N10B	C58	76.2(5)
C10	C11	N3	C21	86.61(14)	C47	C48	C49	C50	0.8(2)
C11	C10	N2	C9	75.98(15)	C48	C47	C52	C51	0.37(19)

Torsion Angles for 2189976 (compound **1c**)

A	B	C	D	Angle/˚	A	B	C	D	Angle/˚
C12	C13	N4	C14	-99.60(12)	C48	C49	C50	C51	-0.3(2)
C12	C13	N4	C26	88.30(13)	C49	C50	C51	C52	-0.2(2)
C13	C12	N3	C11	-92.82(13)	C50	C51	C52	C47	0.1(2)
C13	C12	N3	C21	78.03(13)	C52	C47	C48	C49	-0.82(19)
C14	C15	C16	C17	177.57(11)	C54	C55	C56	C57	177.7(5)
C14	C15	C20	C19	-177.31(12)	C54A	C55A	C56A	C57A	-174.8(6)
C15	C14	N4	C13	81.79(13)	C55	C54	N11	C53	87.1(8)
C15	C14	N4	C26	-105.76(12)	C55A	C54A	N11	C53A	136.8(6)
C15	C16	C17	C18	-0.49(19)	C59	C60	C61	C62	1.3(2)
C16	C15	C20	C19	-0.19(19)	C60	C59	C64	C63	0.56(19)
C16	C17	C18	C19	0.0(2)	C60	C59	N12	C58	-42.09(18)
C17	C18	C19	C20	0.4(2)	C60	C61	C62	C63	0.3(2)
C18	C19	C20	C15	-0.3(2)	C61	C62	C63	C64	-1.6(2)
C20	C15	C16	C17	0.60(18)	C62	C63	C64	C59	1.1(2)
C22	C23	C24	C25	178.31(14)	C64	C59	C60	C61	-1.78(19)
C23	C22	N5	C21	-134.18(12)	C64	C59	N12	C58	141.30(13)
C27	C28	C29	C30	0.26(18)	N7	C33	C34	C35	-179.38(11)
C28	C27	C32	C31	-0.34(18)	N7	C33	C38	C37	-179.17(10)
C28	C27	N6	C26	-19.35(18)	N7	C41	N8	C42	-163.4(3)
C28	C29	C30	C31	-0.1(2)	N7	C41	N8A	C42A	179.8(5)
C29	C30	C31	C32	-0.3(2)	N7	C41	N8B	C42B	171.3(4)
C30	C31	C32	C27	0.5(2)	N8	C41	N7	C33	171.8(2)
C32	C27	C28	C29	-0.05(17)	N8	C42	C43	N9	159.5(3)
C32	C27	N6	C26	164.55(11)	N8A	C41	N7	C33	-165.9(4)
N1	C1	C2	C3	178.47(12)	N8A	C42A	C43A	N9A	-156.2(5)
N1	C1	C6	C5	-177.86(12)	N8B	C41	N7	C33	-158.6(4)
N1	C9	N2	C10	-177.66(11)	N8B	C42B	C43B	N9B	-149.6(7)
N2	C9	N1	C1	179.96(12)	N9	C44	C45	N10	-158.2(5)
N2	C10	C11	N3	-159.77(10)	N9	C53	N11	C54	-165.2(5)
N3	C12	C13	N4	-154.82(10)	N9A	C44A	C45A	N10A	-153.2(5)
N3	C21	N5	C22	173.84(11)	N9A	C53A	N11	C54A	-162.2(6)
N4	C14	C15	C16	28.87(16)	N9B	C44B	C45B	N10B	159.0(4)
N4	C14	C15	C20	-154.13(11)	N10	C46	C47	C48	151.58(13)
N4	C26	N6	C27	169.46(10)	N10	C46	C47	C52	-31.80(18)
N5	C21	N3	C11	-9.19(18)	N10	C58	N12	C59	166.60(13)
N5	C21	N3	C12	-179.84(11)	N10A	C46	C47	C48	151.58(13)
N5	C22	C23	C24	179.11(12)	N10A	C46	C47	C52	-31.80(18)
N6	C26	N4	C13	-3.70(16)	N10A	C58	N12	C59	166.60(13)
N6	C26	N4	C14	-175.74(10)	N10B	C46	C47	C48	149.8(3)
N6	C27	C28	C29	-176.07(10)	N10B	C46	C47	C52	-33.6(3)
N6	C27	C32	C31	175.94(11)	N10B	C58	N12	C59	-174.6(3)
O1	C9	N1	C1	0.0(2)	N11	C53	N9	C43	11.8(8)
O1	C9	N2	C10	2.33(19)	N11	C53	N9	C44	177.9(4)

Torsion Angles for 2189976 (compound **1c**)

A	B	C	D	Angle/˚	A	B	C	D	Angle/˚
O2	C21	N3	C11	170.48(11)	N11	C53A	N9A	C43A	-56.6(6)
O2	C21	N3	C12	-0.17(17)	N11	C53A	N9A	C44A	147.5(4)
O2	C21	N5	C22	-5.82(19)	N11	C53B	N9B	C43B	49.3(8)
O3	C26	N4	C13	175.68(11)	N11	C53B	N9B	C44B	-163.2(5)
O3	C26	N4	C14	3.64(17)	N11	C54	C55	C56	60.4(9)
O3	C26	N6	C27	-9.92(18)	N11	C54A	C55A	C56A	-178.7(6)
C33	C34	C35	C36	-1.62(18)	N12	C58	N10	C45	3.9(4)
C33	C34	C35	C39	176.60(11)	N12	C58	N10	C46	165.67(13)
C34	C33	C38	C37	-1.04(17)	N12	C58	N10A	C45A	-7.0(4)
C34	C33	N7	C41	28.76(19)	N12	C58	N10A	C46	165.67(13)
C34	C35	C36	C37	-0.83(18)	N12	C58	N10B	C45B	7.6(6)
C34	C35	C39	F7	32.29(17)	N12	C58	N10B	C46	-176.0(3)
C34	C35	C39	F8	-87.53(15)	N12	C59	C60	C61	-178.33(12)
C34	C35	C39	F9	153.68(12)	N12	C59	C64	C63	177.23(12)
C35	C36	C37	C38	2.38(17)	O4	C41	N7	C33	10.4(2)
C35	C36	C37	C40	179.45(11)	O4	C41	N8	C42	-1.8(5)
C36	C35	C39	F7	-149.45(12)	O4	C41	N8A	C42A	3.4(9)
C36	C35	C39	F8	90.74(15)	O4	C41	N8B	C42B	2.7(8)
C36	C35	C39	F9	-28.06(17)	O5	C53	N9	C43	-167.0(13)
C36	C37	C38	C33	-1.46(18)	O5	C53	N9	C44	-1.0(15)
C36	C37	C40	F10	15.86(16)	O5	C53	N11	C54	13.6(15)
C36	C37	C40	F11	-103.41(13)	O5A	C53A	N9A	C43A	150.3(5)
C36	C37	C40	F12	136.28(12)	O5A	C53A	N9A	C44A	-5.6(8)
C38	C33	C34	C35	2.54(17)	O5A	C53A	N11	C54A	-8.7(8)
C38	C33	N7	C41	-153.15(13)	O5B	C53B	N9B	C43B	-163.9(12)
C38	C37	C40	F10	-167.01(11)	O5B	C53B	N9B	C44B	-16.4(14)
C38	C37	C40	F11	73.73(14)	O6	C58	N10	C45	176.6(3)
C38	C37	C40	F12	-46.58(15)	O6	C58	N10	C46	-21.6(2)
C39	C35	C36	C37	-179.05(11)	O6	C58	N10A	C45A	165.8(3)
C40	C37	C38	C33	-178.54(11)	O6	C58	N10A	C46	-21.6(2)
C42	C43	N9	C44	110.7(4)	O6	C58	N10B	C45B	-161.3(4)
C42	C43	N9	C53	-83.8(5)	O6	C58	N10B	C46	15.2(5)
C42A	C43A	N9A	C44A	-81.9(6)	O6	C58	N12	C59	-6.0(2)
C42A	C43A	N9A	C53A	124.2(5)					

2a

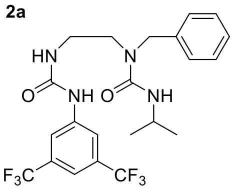

2b

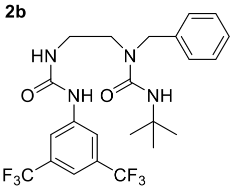

2c

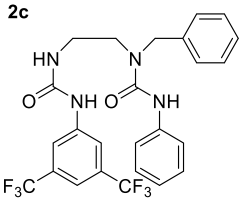

Identification code (**CCDC**)	**2a** (e153)(**2189987**)	**2b** (dte154 (1))(**2189988**)	**2c** (g37b)(**2189990**)
Empirical formula	$C_{22}H_{24}F_6N_4O_2$	$C_{23}H_{26}F_6N_4O_2$	$C_{25.33}H_{22.5}F_6N_{4.17}O_2$
Formula weight	490.45	504.48	524.46
Temperature/K	100(2)	100(2)	100(2)
Crystal system	trigonal	monoclinic	trigonal
Space group	*R*-3	$P2_1/n$	*R*-3
a/Å	31.7595(6)	18.0463(4)	31.6255(7)
b/Å	31.7595(6)	21.9267(6)	31.6255(7)
c/Å	24.6991(6)	18.6546(5)	12.2942(4)
α/°	90	90	90
β/°	90	105.3610(10)	90
γ/°	120	90	120
Volume/$Å^3$	21575.4(10)	7117.8(3)	10648.9(6)
Z	36	12	18
$\rho_{calc}g/cm^3$	1.359	1.412	1.472
μ/mm^{-1}	0.120	0.124	0.127
F(000)	9144.0	3144.0	4860.0
Crystal size/mm^3	0.577 × 0.455 × 0.241	0.474 × 0.426 × 0.26	0.525 × 0.339 × 0.26
Radiation	MoKα (λ = 0.71073)	MoKα (λ = 0.71073)	MoKα (λ = 0.71073)
2θ range for data collection/°	2.216 to 52.744	2.928 to 55.874	3.632 to 52.822
Index ranges	-38 ≤ h ≤ 39, -39 ≤ k ≤ 39, -23 ≤ l ≤ 30	-23 ≤ h ≤ 23, -28 ≤ k ≤ 21, -21 ≤ l ≤ 24	-39 ≤ h ≤ 39, -39 ≤ k ≤ 39, -15 ≤ l ≤ 15
Reflections collected	58951	64703	85699
Independent reflections	9812 [R_{int} = 0.0521, R_{sigma} = 0.0334]	17042 [R_{int} = 0.0684, R_{sigma} = 0.0646]	4853 [R_{int} = 0.0471, R_{sigma} = 0.0213]
Data/restraints/parameters	9812/598/852	17042/70/1015	4853/0/403
Goodness-of-fit on F^2	1.041	1.018	1.034
Final R indexes [I>=2σ (I)]	R_1 = 0.0507, wR_2 = 0.1223	R_1 = 0.0533, wR_2 = 0.1138	R_1 = 0.0397, wR_2 = 0.0839
Final R indexes [all data]	R_1 = 0.0682, wR_2 = 0.1346	R_1 = 0.1034, wR_2 = 0.1395	R_1 = 0.0518, wR_2 = 0.0909
Largest diff. peak/hole / e $Å^{-3}$	0.48/-0.29	0.51/-0.36	0.25/-0.26

Hydrogen Bonds for 2189987 (compound **2a**)

**D**	**H**	**A**	**d(D-H)/Å**	**d(H-A)/Å**	**d(D-A)/Å**	**D-H-A/°**
N5	H5	$O1^1$	0.85(3)	2.01(3)	2.812(2)	155(3)

Torsion Angles for 2189987 (compound **2a**)

A	B	C	D	Angle/˚	A	B	C	D	Angle/˚
N2	C5	C6	C1AA	73.0(3)	C25	N6	C24	O3	4.8(5)
N2	C5	C6	C7	-106.9(3)	C25	N6	C24	N5	-175.2(3)
N2	C11	C12	N3	-162.9(2)	C25	N6	C32	C33	94.9(4)
N4	C14	C15	C16	-178.7(3)	C25	C26	C27	C28	179.7(4)
C0AA	C14	C15	C16	-0.1(4)	C25	C26	C31	C30	179.8(3)
C0AA	C18	C20	F4	152.7(3)	C26	C27	C28	C29	0.8(7)
C0AA	C18	C20	F5	32.1(4)	C27	C26	C31	C30	-1.3(6)
C0AA	C18	C20	F6	-88.3(4)	C27	C28	C29	C30	-1.9(7)
C0AA	C18	C20	F4A	81.2(10)	C28	C29	C30	C31	1.4(7)
C0AA	C18	C20	F5A	-33.0(9)	C29	C30	C31	C26	0.3(6)
C0AA	C18	C20	F6A	-155.4(9)	C31	C26	C27	C28	0.8(6)
C1AA	C6	C7	C8	0.8(4)	C32	N6	C24	O3	-179.4(3)
C2	N1	C4	O1	3.5(4)	C32	N6	C24	N5	0.7(5)
C2	N1	C4	N2	-177.2(2)	C32	N6	C25	C26	-82.5(4)
C4	N1	C2	C1	-161.4(3)	C33	N7	C34	O4	-9.4(5)
C4	N1	C2	C3	76.2(3)	C33	N7	C34	N8	172.0(3)
C4	N2	C5	C6	-97.3(3)	C34	N7	C33	C32	-70.5(4)
C4	N2	C11	C12	78.1(3)	C34	N8	C35	C36	-2.5(4)
C5	N2	C4	O1	-8.5(4)	C34	N8	C35	C40	176.5(3)
C5	N2	C4	N1	172.2(2)	C35	N8	C34	O4	2.4(5)
C5	N2	C11	C12	-93.3(3)	C35	N8	C34	N7	-179.0(3)
C5	C6	C7	C8	-179.4(3)	C35	N8	C34A	O4A	-12.8(19)
C6	C1AA	C10	C9	0.3(4)	C35	N8	C34A	N7A	170.5(8)
C6	C7	C8	C9	1.1(5)	C35	C36	C37	C38	0.2(4)
C7	C8	C9	C10	-2.3(5)	C35	C36	C37	C41	-176.5(3)
C8	C9	C10	C1AA	1.6(5)	C36	C35	C40	C39	0.2(4)
C10	C1AA	C6	C5	178.7(3)	C36	C37	C38	C39	-0.7(4)
C10	C1AA	C6	C7	-1.5(4)	C36	C37	C41	F7	-30.2(5)
C11	N2	C4	O1	-179.6(2)	C36	C37	C41	F8	89.2(4)
C11	N2	C4	N1	1.0(4)	C36	C37	C41	F9	-150.3(5)
C11	N2	C5	C6	74.3(3)	C36	C37	C41	F7A	-115.5(11)
C12	N3	C13	O2	4.8(4)	C36	C37	C41	F9A	135.3(15)
C12	N3	C13	N4	-175.7(2)	C36	C37	C41	F8A	-9.3(14)
C13	N3	C12	C11	73.0(3)	C37	C38	C39	C40	1.0(4)
C13	N4	C14	C0AA	-179.5(3)	C37	C38	C39	C42	-179.6(3)
C13	N4	C14	C15	-0.9(4)	C38	C37	C41	F7	153.1(4)
C14	N4	C13	O2	-2.2(4)	C38	C37	C41	F8	-87.5(4)
C14	N4	C13	N3	178.3(2)	C38	C37	C41	F9	32.9(6)
C14	C0AA	C18	C17	-0.4(4)	C38	C37	C41	F7A	67.7(11)
C14	C0AA	C18	C20	178.2(2)	C38	C37	C41	F9A	-41.4(16)
C14	C15	C16	C17	-0.7(4)	C38	C37	C41	F8A	173.9(13)
C14	C15	C16	C19	-179.3(3)	C38	C39	C40	C35	-0.8(4)
C15	C16	C17	C18	1.0(4)	C38	C39	C42	F10	10.4(4)

Torsion Angles for 2189987 (compound **2a**)

A	B	C	D	Angle/˚	A	B	C	D	Angle/˚
C15	C16	C19	F1	70.9(6)	C38	C39	C42	F11	129.8(3)
C15	C16	C19	F2	-47.8(5)	C38	C39	C42	F12	-109.0(3)
C15	C16	C19	F3	-158.8(5)	C40	C35	C36	C37	0.1(4)
C15	C16	C19	F3A	167.9(6)	C40	C39	C42	F10	-170.2(2)
C15	C16	C19	F1A	53.6(7)	C40	C39	C42	F11	-50.8(4)
C15	C16	C19	F2A	-66.6(6)	C40	C39	C42	F12	70.5(3)
C16	C17	C18	C0AA	-0.4(4)	C41	C37	C38	C39	175.9(3)
C16	C17	C18	C20	-179.0(2)	C42	C39	C40	C35	179.8(3)
C17	C16	C19	F1	-107.7(6)	N5A	C24A	N6A	C25A	-5(2)
C17	C16	C19	F2	133.6(5)	N5A	C24A	N6A	C32A	178.1(14)
C17	C16	C19	F3	22.6(6)	C22A	N5A	C24A	O3A	2(3)
C17	C16	C19	F3A	-10.7(6)	C22A	N5A	C24A	N6A	172.8(13)
C17	C16	C19	F1A	-125.0(6)	C34A	N8	C35	C36	-169.9(7)
C17	C16	C19	F2A	114.8(6)	C34A	N8	C35	C40	9.1(8)
C17	C18	C20	F4	-28.8(4)	C34A	N7A	C33A	C25A	78.9(16)
C17	C18	C20	F5	-149.3(3)	O4A	C34A	N7A	C33A	-1(2)
C17	C18	C20	F6	90.2(4)	C24A	N5A	C22A	C21A	77.9(18)
C17	C18	C20	F4A	-100.2(10)	C24A	N5A	C22A	C23A	-160.4(15)
C17	C18	C20	F5A	145.6(9)	C24A	N6A	C25A	C33A	84.9(19)
C17	C18	C20	F6A	23.2(10)	C24A	N6A	C32A	C26A	-88.2(18)
C18	C0AA	C14	N4	179.3(2)	O3A	C24A	N6A	C25A	166.4(14)
C18	C0AA	C14	C15	0.6(4)	O3A	C24A	N6A	C32A	-11(2)
C19	C16	C17	C18	179.5(3)	N6A	C25A	C33A	N7A	-161.9(11)
N6	C25	C26	C27	136.9(4)	N6A	C32A	C26A	C31A	-138.4(12)
N6	C25	C26	C31	-44.3(5)	N6A	C32A	C26A	C27A	45.8(17)
N6	C32	C33	N7	160.7(3)	C25A	N6A	C32A	C26A	94.8(18)
N8	C35	C36	C37	179.1(3)	C32A	N6A	C25A	C33A	-98.3(16)
N8	C35	C40	C39	-178.9(2)	C32A	C26A	C31A	C30A	-175.7(14)
N8	C34A	N7A	C33A	176.5(10)	C32A	C26A	C27A	C28A	175.9(13)
C22	N5	C24	O3	-3.7(5)	C26A	C31A	C30A	C29A	0.0
C22	N5	C24	N6	176.2(3)	C31A	C26A	C27A	C28A	0.0
C24	N5	C22	C21	163.3(4)	C31A	C30A	C29A	C28A	0.0
C24	N5	C22	C23	-75.4(4)	C30A	C29A	C28A	C27A	0.0
C24	N6	C25	C26	93.5(4)	C29A	C28A	C27A	C26A	0.0
C24	N6	C32	C33	-81.1(4)	C27A	C26A	C31A	C30A	0.0

Hydrogen Bonds for 2189988 (compound **2b**)

D	H	A	d(D-H)/Å	d(H-A)/Å	d(D-A)/Å	D-H-A/°
C50	H50	O5	0.9500	2.150(3)	2.780(3)	122.57(7)
N5	H5a	O6	0.84(3)	2.08(3)	2.900(3)	165(3)

Torsion Angles for 2189988 (compound **2b**)

A	B	C	D	Angle/˚	A	B	C	D	Angle/˚
F1	C7	C2	C1	134.7(2)	N7	C39	C40	C45	-22.2(2)
F1	C7	C2	C3	-47.4(3)	C01A	C24	C25	C26	1.0(3)
F2	C7	C2	C1	13.9(3)	C01A	C24	C25	C29	-176.2(2)
F2	C7	C2	C3	-168.3(2)	C01A	C28	C27	C26	0.5(3)
F3	C7	C2	C1	-106.6(2)	C01A	C28	C27	C30	178.1(2)
F3	C7	C2	C3	71.2(3)	C24	C25	C26	C27	-1.2(3)
F4	C8	C4	C3	32.6(3)	C25	C26	C27	C28	0.4(3)
F4	C8	C4	C5	-152.1(2)	C25	C26	C27	C30	-177.1(2)
F5	C8	C4	C3	-86.7(2)	C39	C40	C41	C42	-179.55(19)
F5	C8	C4	C5	88.6(2)	C39	C40	C45	C44	179.4(2)
F6	C8	C4	C3	154.7(2)	C40	C41	C42	C43	0.0(3)
F6	C8	C4	C5	-29.9(3)	C40	C45	C44	C43	0.2(2)
O1	C9	N1	C6	7.1(3)	C41	C42	C43	C44	-1.1(3)
O1	C9	N2	C10	-5.0(3)	C42	C43	C44	C45	0.9(3)
O2	C12	N3	C11	161.1(2)	F13	C51	C47	C46	87.8(2)
O2	C12	N3	C17	-4.2(3)	F13	C51	C47	C48	-89.6(2)
O2	C12	N4	C13	-15.5(3)	F14	C51	C47	C46	-32.4(3)
N1	C6	C1	C2	-179.9(2)	F14	C51	C47	C48	150.2(2)
N1	C6	C5	C4	178.8(2)	F15	C51	C47	C46	-152.0(2)
N1	C9	N2	C10	174.8(2)	F15	C51	C47	C48	30.6(3)
N2	C10	C11	N3	-161.20(18)	F16	C52	C49	C48	25.8(3)
N3	C12	N4	C13	165.1(2)	F16	C52	C49	C50	-155.5(2)
N3	C17	C18	C19	-22.8(2)	F17	C52	C49	C48	-94.7(2)
N3	C17	C18	C23	157.58(19)	F17	C52	C49	C50	84.1(2)
C1	C2	C3	C4	-0.9(3)	F18	C52	C49	C48	145.4(2)
C1	C6	C5	C4	-1.6(3)	F18	C52	C49	C50	-35.9(3)
C2	C3	C4	C5	-0.2(3)	O5	C53	N9	C019	7.5(4)
C2	C3	C4	C8	174.9(2)	O5	C53	N10	C54	-0.0(4)
C3	C4	C5	C6	1.5(3)	O5	C53	N10A	C54A	7.0(6)
C17	C18	C19	C20	-178.6(3)	O6	C56	N11	C55	-150.4(3)
C17	C18	C23	C22	-179.9(2)	O6	C56	N11	C61	5.9(3)
C18	C19	C20	C21	-1.2(3)	O6	C56	N11	C55A	161.3(6)
C18	C23	C22	C21	-1.7(3)	O6	C56	N12	C57	16.2(3)
C19	C20	C21	C22	-0.1(4)	N9	C019	C46	C47	-179.4(2)
C20	C21	C22	C23	1.5(4)	N9	C019	C50	C49	178.5(2)
F7	C29	C25	C24	96.5(2)	N9	C53	N10	C54	-174.5(3)
F7	C29	C25	C26	-80.8(2)	N9	C53	N10A	C54A	158.4(7)
F8	C29	C25	C24	-22.2(2)	N10	C53	N10A	C54A	-103.8(8)
F8	C29	C25	C26	160.5(2)	N10	C54	C55	N11	159.0(2)
F9	C29	C25	C24	-142.9(2)	N11	C56	N12	C57	-163.8(2)
F9	C29	C25	C26	39.8(3)	N11	C61	C62	C63	23.9(3)
F10	C30	C27	C26	98.4(2)	N11	C61	C62	C67	-156.7(2)
F10	C30	C27	C28	-79.2(2)	N11	C55A	C54A	N10A	-164.1(7)

Torsion Angles for 2189988 (compound **2b**)

A	B	C	D	Angle/˚	A	B	C	D	Angle/˚
F11	C30	C27	C26	-20.0(3)	C019	C46	C47	C48	1.0(3)
F11	C30	C27	C28	162.4(2)	C019	C46	C47	C51	-176.4(2)
F12	C30	C27	C26	-141.3(2)	C019	C50	C49	C48	0.8(3)
F12	C30	C27	C28	41.1(3)	C019	C50	C49	C52	-177.9(2)
O3	C31	N5	C01A	4.0(3)	C46	C47	C48	C49	-1.6(3)
O3	C31	N6	C32	1.8(3)	C47	C48	C49	C50	0.7(3)
O4	C34	N7	C33	175.8(2)	C47	C48	C49	C52	179.4(2)
O4	C34	N7	C39	-6.5(3)	C53	N10A	C54A	C55A	65.8(11)
O4	C34	N8	C35	1.2(3)	C61	C62	C63	C64	179.6(2)
N5	C01A	C24	C25	178.5(2)	C61	C62	C67	C66	-179.4(2)
N5	C01A	C28	C27	-179.2(2)	C62	C63	C64	C65	0.0(3)
N5	C31	N6	C32	-178.7(2)	C62	C67	C66	C65	-0.5(3)
N6	C32	C33	N7	163.50(17)	C63	C64	C65	C66	-0.5(3)
N7	C34	N8	C35	-178.79(18)	C64	C65	C66	C67	0.7(3)
N7	C39	C40	C41	158.45(18)					

Hydrogen Bonds for 2189990 (compound **2c**)

D	H	A	d(D-H)/Å	d(H-A)/Å	d(D-A)/Å	D-H-A/°
N2	H2A	O2[1]	0.857(18)	1.979(19)	2.8202(16)	166.7(16)
N4	H4A	O1	0.854(19)	2.108(19)	2.9473(16)	167.4(17)

Torsion Angles for 2189990 (compound **2c**)

A	B	C	D	Angle/˚	A	B	C	D	Angle/˚
C1	C2	C3	C4	-0.6(2)	C13	C14	C15	C16	0.4(2)
C1	C2	C3	C7	-178.25(14)	C14	C13	C18	C17	1.6(2)
C2	C1	C6	C5	-0.6(2)	C14	C15	C16	C17	0.6(2)
C2	C1	N1	C9	24.3(2)	C15	C16	C17	C18	-0.5(2)
C2	C3	C4	C5	-0.2(2)	C16	C17	C18	C13	-0.6(2)
C2	C3	C7	F4	-141.50(15)	C18	C13	C14	C15	-1.5(2)
C2	C3	C7	F5	98.36(17)	C20	C21	C22	C23	-1.1(2)
C2	C3	C7	F6	-20.9(2)	C21	C20	C25	C24	-0.3(2)
C3	C4	C5	C6	0.6(2)	C21	C20	N4	C19	139.45(15)
C3	C4	C5	C8	-178.80(14)	C21	C22	C23	C24	-0.2(2)
C4	C3	C7	F4	40.8(2)	C22	C23	C24	C25	1.2(2)
C4	C3	C7	F5	-79.35(19)	C23	C24	C25	C20	-1.0(2)
C4	C3	C7	F6	161.39(14)	C25	C20	C21	C22	1.3(2)
C4	C5	C6	C1	-0.2(2)	C25	C20	N4	C19	-43.3(2)
C4	C5	C8	F1	-154.42(14)	N1	C1	C2	C3	179.50(14)
C4	C5	C8	F2	85.83(18)	N1	C1	C6	C5	-179.17(13)
C4	C5	C8	F3	-34.4(2)	N1	C9	N2	C10	171.03(13)

Torsion Angles for 2189990 (compound **2c**)

A	B	C	D	Angle/˚	A	B	C	D	Angle/˚
C6	C1	C2	C3	1.0(2)	N2	C9	N1	C1	-179.61(14)
C6	C1	N1	C9	-157.12(14)	N2	C10	C11	N3	-159.61(12)
C6	C5	C8	F1	26.2(2)	N3	C12	C13	C14	70.33(18)
C6	C5	C8	F2	-93.53(18)	N3	C12	C13	C18	-108.98(15)
C6	C5	C8	F3	146.24(14)	N3	C19	N4	C20	177.71(13)
C7	C3	C4	C5	177.50(14)	N4	C19	N3	C11	1.9(2)
C8	C5	C6	C1	179.16(13)	N4	C19	N3	C12	172.41(12)
C10	C11	N3	C12	-88.01(16)	N4	C20	C21	C22	178.67(14)
C10	C11	N3	C19	82.83(17)	N4	C20	C25	C24	-177.52(14)
C11	C10	N2	C9	79.14(17)	O1	C9	N1	C1	0.8(2)
C12	C13	C14	C15	179.19(14)	O1	C9	N2	C10	-9.4(2)
C12	C13	C18	C17	-179.07(14)	O2	C19	N3	C11	-179.67(13)
C13	C12	N3	C11	70.05(17)	O2	C19	N3	C12	-9.1(2)
C13	C12	N3	C19	-101.08(15)	O2	C19	N4	C20	-0.8(2)

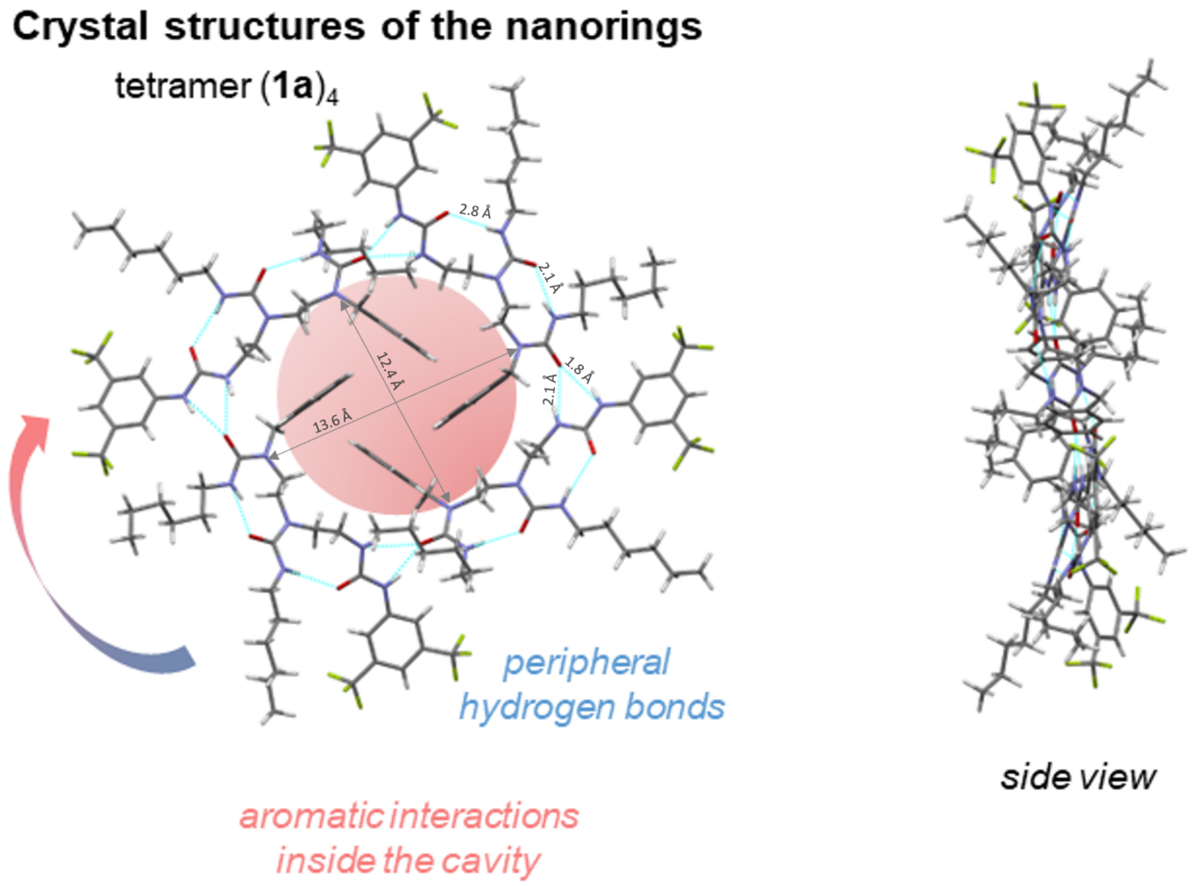

Figure SI 1. Nanorings formed by head-to-tail arrangement of **1a** in the crystal structure.

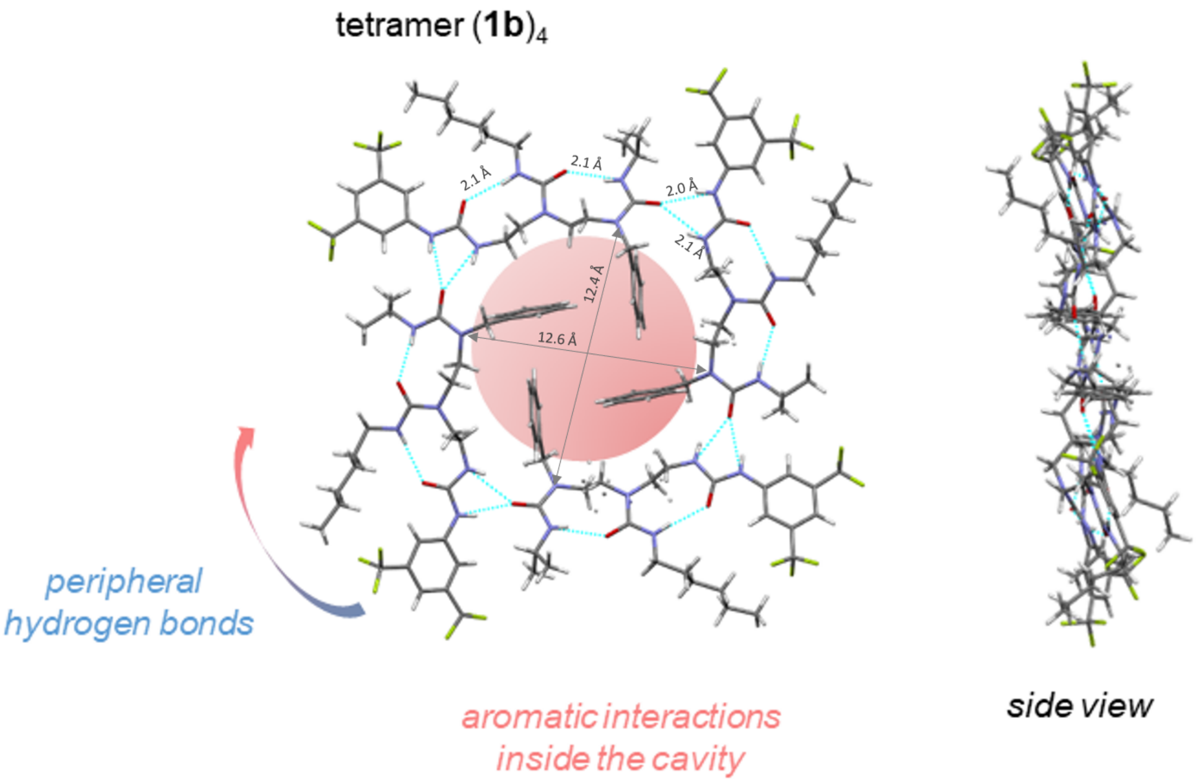

Figure SI 2. Nanorings formed by head-to-tail arrangement of **1b** in the crystal structure.

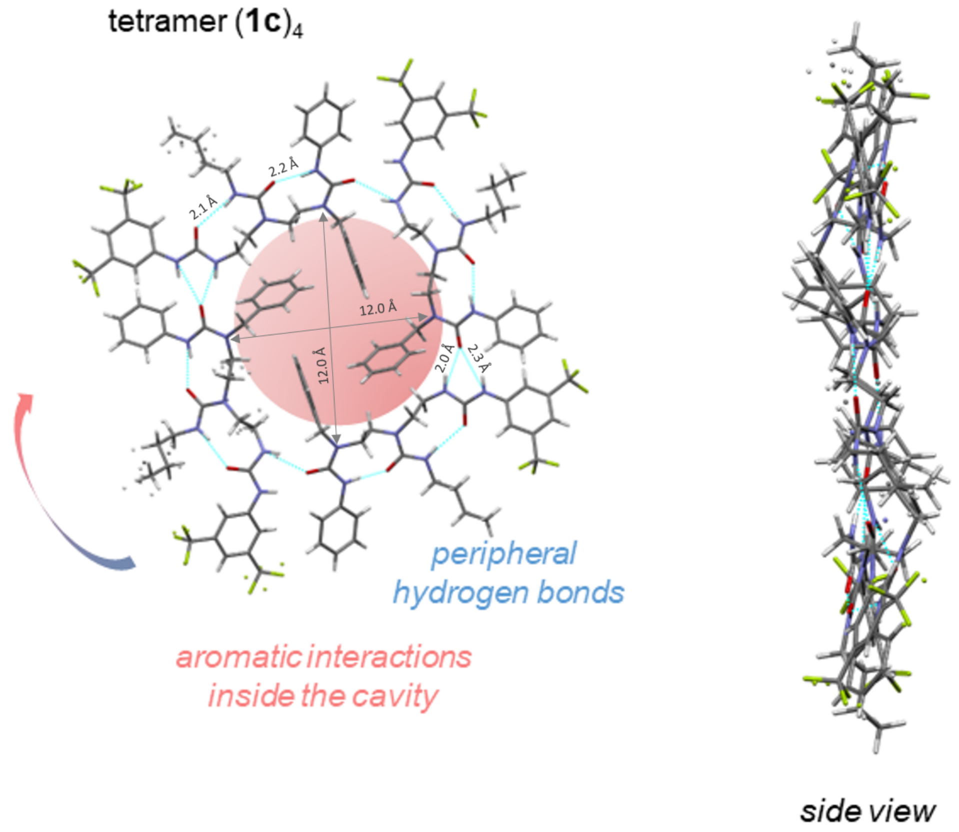

Figure SI 3. Nanorings formed by head-to-tail arrangement of **1c** in the crystal structure.

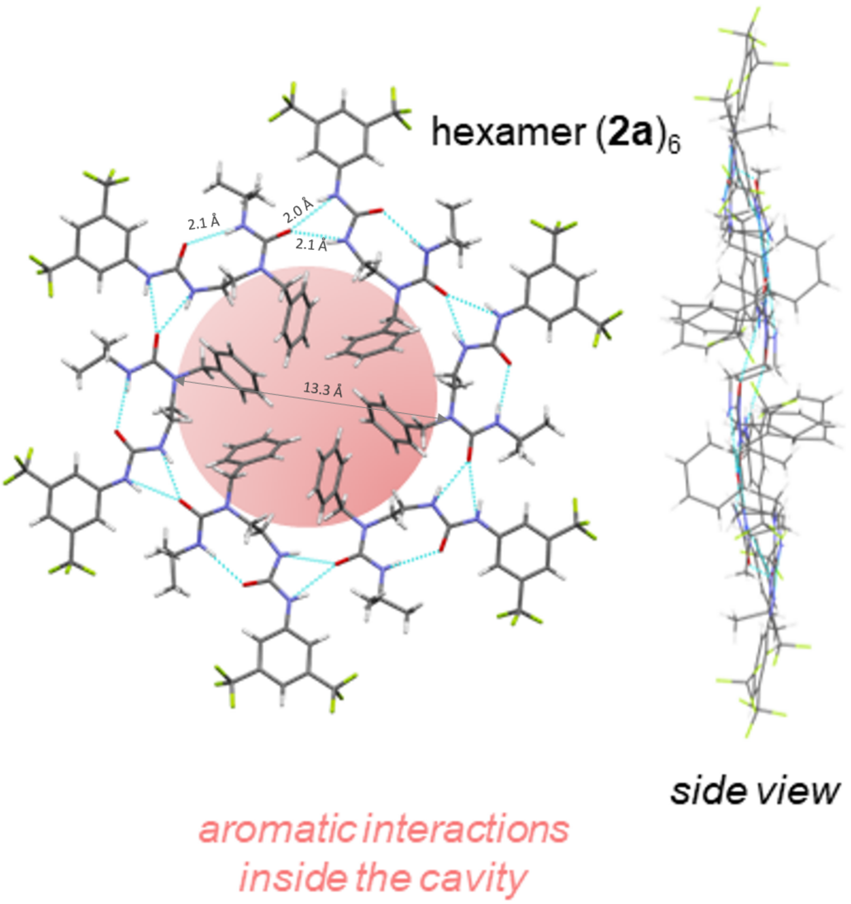

Figure SI 4. Nanorings formed by head-to-tail arrangement of **2a** in the crystal structure.

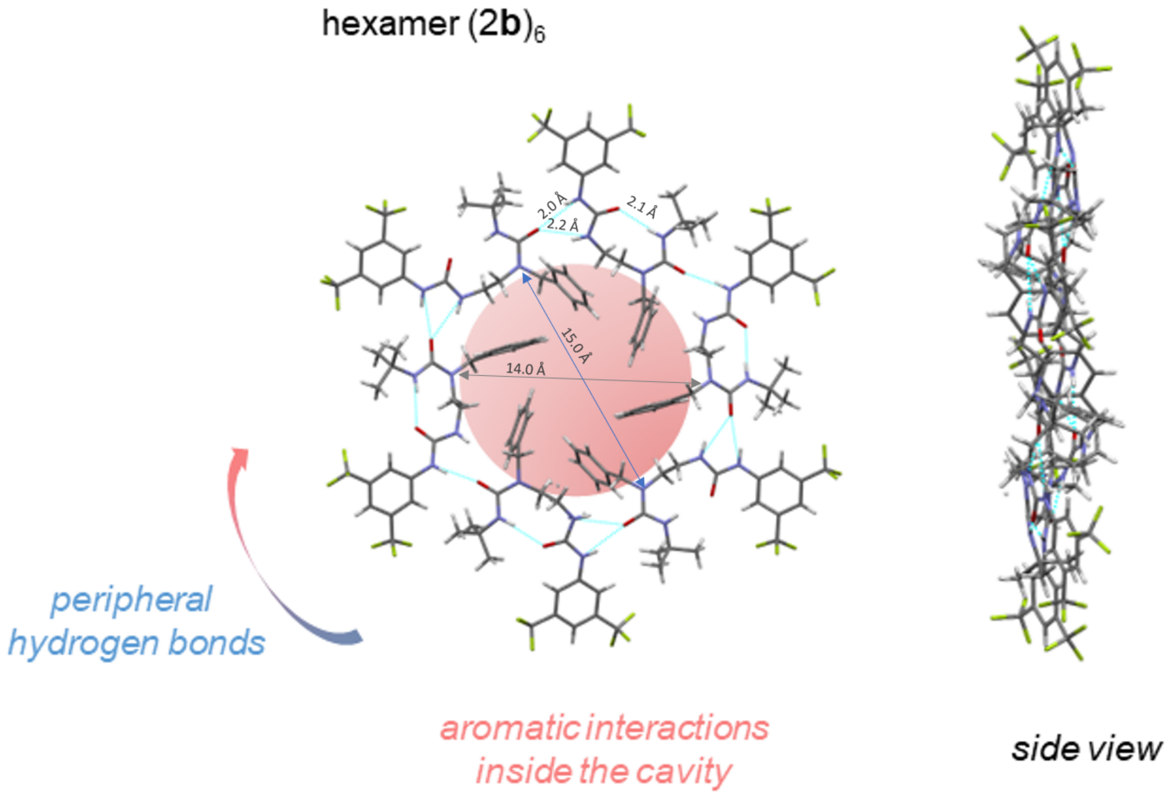

Figure SI 5. Nanorings formed by head-to-tail arrangement of **2b** in the crystal structure.

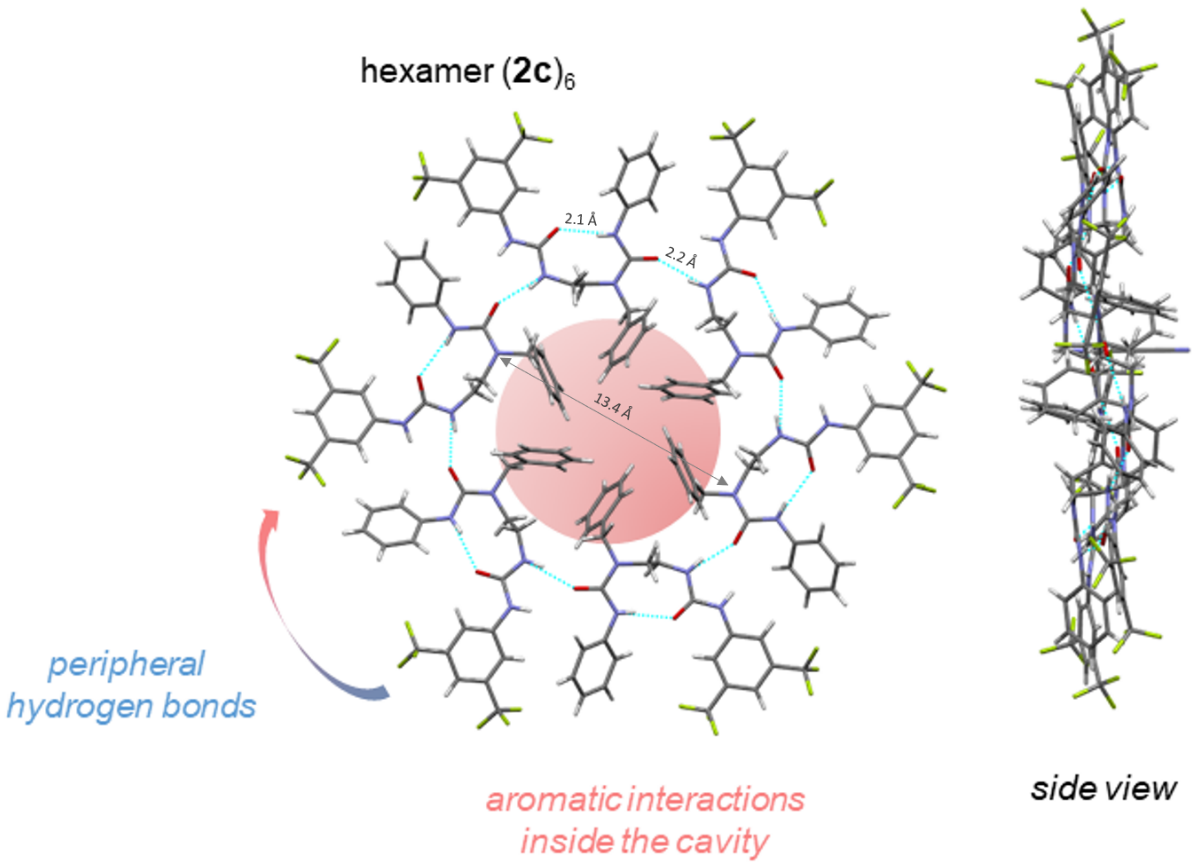

Figure SI 6. Nanorings formed by head-to-tail arrangement of **2c** in the crystal structure.

1d

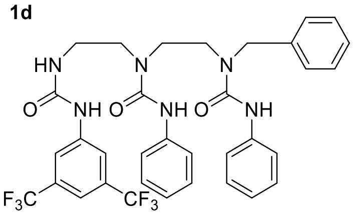

1e

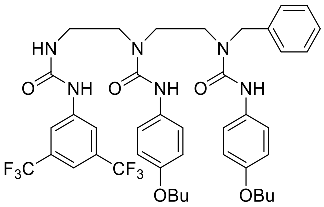

Identification code (**CCDC**)	**1d** (g48) (**2189991**)	**1e** (m_d70_c1_col1__twin1_hklf4 (**2189996**)
Empirical formula	$C_{34}H_{32}F_6N_6O_3$	$C_{42}H_{47.5}F_6N_6O_5$
Formula weight	686.65	830.36
Temperature/K	100(2)	100(2)
Crystal system	monoclinic	triclinic
Space group	*C*2/*c*	*P*-1
a/Å	18.9800(6)	9.9573(5)
b/Å	20.3627(6)	19.5693(11)
c/Å	16.4981(5)	20.9138(11)
α/°	90	84.902(4)
β/°	92.891(3)	89.962(4)
γ/°	90	87.554(4)
Volume/Å^3	6368.2(3)	4055.4(4)
Z	8	4

ρ_{calc}g/cm^3	1.432	1.360
μ/mm^{-1}	0.117	0.100
F(000)	2848.0	1742.0
Crystal size/mm^3	0.3 × 0.26 × 0.25	0.04 × 0.04 × 0.02
Radiation	Mo Kα (λ = 0.71073)	Synchrotron (λ = 0.6889)
2θ range for data collection/°	6.714 to 58.568	3.79 to 49.22
Index ranges	-24 ≤ h ≤ 24, -26 ≤ k ≤ 27, -22 ≤ l ≤ 22	-11 ≤ h ≤ 11, -23 ≤ k ≤ 23, -25 ≤ l ≤ 25
Reflections collected	25400	40914
Independent reflections	7599 [R_{int} = 0.0424, R_{sigma} = 0.0487]	40914 [R_{int} = ?, R_{sigma} = 0.0395]
Data/restraints/parameters	7599/203/508	40914/0/1070
Goodness-of-fit on F^2	1.045	1.508
Final R indexes [I>=2σ (I)]	R_1 = 0.0520, wR_2 = 0.1086	R_1 = 0.1231, wR_2 = 0.3821
Final R indexes [all data]	R_1 = 0.0762, wR_2 = 0.1217	R_1 = 0.1602, wR_2 = 0.4043
Largest diff. peak/hole / e Å^{-3}	0.36/-0.33	0.92/-0.64

Hydrogen Bonds for 2189991 (compound **1d**)

D	H	A	d(D-H)/Å	d(H-A)/Å	d(D-A)/Å	D-H-A/°
C2	H2	O23	0.9500	2.207(2)	2.814(2)	120.74(6)
N9	H9	O17^1	0.8800	2.028(2)	2.807(2)	147.03(7)
N9A	H9A	O17^1	0.8800	1.961(15)	2.766(15)	151.3(5)

Torsion Angles for 2189991 (compound **1d**)

A	B	C	D	Angle/˚	A	B	C	D	Angle/˚
F25	C24	C5	C4	45.70(19)	C13	N12A	C11A	C10A	-73.1(15)
F25	C24	C5	C6	-136.72(15)	C2	C3	C4	C5	0.4(2)
F26	C24	C5	C4	-72.64(18)	C2	C3	C28	F30	170.3(7)
F26	C24	C5	C6	104.94(16)	C2	C3	C28	F31	55.2(4)
O17	C16	N18	C38	-2.3(2)	C2	C3	C28	F31A	81.4(14)
O17	C16	N15	C14	173.91(16)	C2	C3	C28	F29A	-49.3(8)
O17	C16	N15	C19	5.7(2)	C2	C3	C28	F30A	-153.3(11)
N18	C16	N15	C14	-6.6(2)	C2	C1	N7	C8	-12.4(2)
N18	C16	N15	C19	-174.82(15)	C2	C1	C6	C5	0.9(2)
N18	C38	C43	C42	178.86(15)	C43	C38	C39	C40	0.4(2)
N18	C38	C39	C40	-179.94(18)	C43	C42	C41	C40	-0.1(2)
O21	C20	N22	C44	12.3(2)	N15	C19	C32	C37	-58.67(19)
O21	C20	N12	C13	14.6(2)	N15	C19	C32	C33	117.90(19)
O21	C20	N12	C11	-173.74(18)	C3	C4	C5	C6	-0.1(2)
O21	C20	N12A	C13	-45.8(3)	C3	C4	C5	C24	177.43(16)
O21	C20	N12A	C11A	163.2(11)	C1	N7	C8	N9	172.1(2)
O23	C8	N7	C1	-6.0(3)	C1	N7	C8	N9A	-151.5(9)

Torsion Angles for 2189991 (compound **1d**)

A	B	C	D	Angle/˚	A	B	C	D	Angle/˚
O23	C8	N9	C10	-0.5(2)	C1	C6	C5	C4	-0.5(2)
O23	C8	N9A	C10A	18.5(9)	C1	C6	C5	C24	-178.06(16)
N22	C44	C45	C46	-178.95(16)	N7	C8	N9	C10	-178.70(16)
N22	C44	C49	C48	178.04(17)	N7	C8	N9A	C10A	166.4(11)
N22	C20	N12	C13	-167.67(17)	N12	C20	N12A	C11A	-89.9(14)
N22	C20	N12	C11	4.0(2)	N12	C11	C10	N9	159.25(17)
N22	C20	N12A	C13	161.6(7)	C19	C32	C37	C36	176.93(19)
N22	C20	N12A	C11A	10.6(8)	C19	C32	C33	C34	-176.2(2)
C14	C13	N12	C20	-84.37(18)	C38	C39	C40	C41	0.8(2)
C14	C13	N12	C11	103.34(17)	C44	C45	C46	C47	0.6(2)
C14	C13	N12A	C20	-30.3(3)	C44	C49	C48	C47	1.1(2)
C14	C13	N12A	C11A	122.5(12)	C20	N12A	C11A	C10A	76.2(17)
C14	N15	C19	C32	-67.96(16)	C39	C40	C41	C42	-1.0(2)
F27	C24	C5	C4	166.98(16)	C32	C37	C36	C35	-0.3(3)
F27	C24	C5	C6	-15.4(2)	C32	C33	C34	C35	-1.1(3)
F29	C28	C3	C2	-59.7(4)	C48	C47	C46	C45	-0.9(2)
F29	C28	C3	C4	119.6(4)	C37	C36	C35	C34	-0.3(3)
C13	N12	C20	N12A	-74.7(12)	N9	C8	N9A	C10A	-89.9(14)
C13	N12	C11	C10	83.4(2)	C8	N9A	C10A	C11A	50.9(18)
C13	N12A	C20	N12	61.1(11)	N12A	C11A	C10A	N9A	-161.6(14)

Hydrogen Bonds for 2189996 (compound **1e**)

D	H	A	d(D-H)/Å	d(H-A)/Å	d(D-A)/Å	D-H-A/°
N6	H6	O9	0.8800	1.985(5)	2.826(5)	159.33(13)
N12	H12	$O4^1$	0.8800	1.958(5)	2.803(5)	160.63(13)

Torsion Angles for 2189996 (compound **1e**)

A	B	C	D	Angle/˚	A	B	C	D	Angle/˚
F1	C1	C2	C3	-59.0(5)	C13	C14	C15	C16	-55.2(7)
F1	C1	C2	C42	123.0(4)	F7	C43	C44	C45	58.1(5)
F4	C41	C40	C42	22.2(6)	F7	C43	C44	C84	-124.3(4)
F4	C41	C40	C39	-159.3(4)	F8	C43	C44	C45	-60.5(5)
F2	C1	C2	C3	179.8(4)	F8	C43	C44	C84	117.1(4)
F2	C1	C2	C42	1.8(5)	F9	C43	C44	C45	-179.5(4)
F3	C1	C2	C3	60.0(5)	F9	C43	C44	C84	-1.9(5)
F3	C1	C2	C42	-118.0(4)	F10	C83	C82	C81	-75.0(6)
F5	C41	C40	C42	142.9(4)	F10	C83	C82	C84	105.0(5)
F5	C41	C40	C39	-38.7(5)	F11	C83	C82	C81	162.1(5)
F6	C41	C40	C42	-97.9(4)	F11	C83	C82	C84	-17.9(7)
F6	C41	C40	C39	80.5(5)	F12	C83	C82	C81	40.3(6)

Torsion Angles for 2189996 (compound **1e**)

A	B	C	D	Angle/˚	A	B	C	D	Angle/˚
O1	C5	N1	C4	3.9(6)	F12	C83	C82	C84	-139.7(5)
O1	C5	N2	C6	6.7(5)	O6	C47	N8	C48	-5.4(5)
O2	C8	N4	C9	-7.9(5)	O6	C47	N7	C46	-3.5(6)
O2	C8	N3	C19	-4.8(5)	O9	C63	N11	C62	6.2(5)
O2	C8	N3	C7	-175.3(4)	O9	C63	N11	C74	171.5(4)
O4	C21	N5	C20	-7.0(4)	O9	C63	N12	C64	7.3(5)
O4	C21	N5	C32	-169.3(4)	O7	C50	N9	C61	5.6(5)
O4	C21	N6	C22	-7.4(5)	O7	C50	N9	C49	173.7(4)
O5	C25	C30	C31	-175.8(5)	O7	C50	N10	C51	4.8(5)
O5	C25	C24	C23	176.7(4)	O10	C67	C72	C73	178.3(4)
O5	C26	C27	C28	175.3(6)	O10	C67	C66	C65	-179.1(4)
O3	C12	C11	C10	-179.2(5)	O10	C68	C69	C70	-172.8(4)
O3	C12	C17	C18	179.2(5)	O8	C54	C53	C52	174.7(5)
O3	C13	C14	C15	-52.0(6)	O8	C54	C59	C60	-174.7(5)
N1	C5	N2	C6	-173.2(3)	O8	C55	C56	C57	-174.0(5)
N1	C4	C3	C2	179.0(4)	N11	C63	N12	C64	-171.4(3)
N1	C4	C39	C40	-178.9(4)	N11	C62	C61	N9	179.2(3)
N4	C8	N3	C19	176.1(4)	N11	C74	C75	C76	-157.5(4)
N4	C8	N3	C7	5.6(5)	N11	C74	C75	C80	21.9(5)
N4	C9	C10	C11	-179.8(4)	N9	C50	N10	C51	-172.4(4)
N4	C9	C18	C17	179.7(5)	N9	C49	C48	N8	56.7(4)
N3	C19	C20	N5	-175.0(3)	N8	C47	N7	C46	179.0(4)
N3	C7	C6	N2	-56.7(4)	N12	C64	C65	C66	180.0(4)
N5	C21	N6	C22	170.0(3)	N12	C64	C73	C72	179.3(4)
N5	C32	C33	C34	159.5(4)	N7	C46	C81	C82	179.8(4)
N5	C32	C33	C38	-22.7(4)	N7	C46	C45	C44	179.6(4)
N6	C22	C23	C24	-176.9(4)	N10	C51	C52	C53	-177.9(4)
N6	C22	C31	C30	177.7(4)	N10	C51	C60	C59	178.0(5)
C9	C10	C11	C12	0.8(6)	C64	C65	C66	C67	0.5(5)
C9	C18	C17	C12	-0.7(6)	C64	C73	C72	C67	1.3(5)
C22	C23	C24	C25	-1.2(5)	C51	C52	C53	C54	1.2(5)
C22	C31	C30	C25	-0.5(6)	C51	C60	C59	C54	-1.3(6)
C1	C2	C3	C4	-177.8(4)	C46	C81	C82	C84	0.0(6)
C1	C2	C42	C40	177.3(4)	C46	C81	C82	C83	-179.9(5)
C23	C24	C25	C30	-1.8(5)	C46	C45	C44	C43	178.8(4)
C31	C30	C25	C24	2.6(5)	C46	C45	C44	C84	1.3(5)
C10	C11	C12	C17	0.9(6)	C81	C82	C84	C44	-0.3(6)
C33	C34	C35	C36	-1.5(6)	C79	C80	C75	C74	178.7(4)
C33	C38	C37	C36	0.4(5)	C79	C80	C75	C76	-1.9(5)
C4	C3	C2	C42	0.2(5)	C79	C78	C77	C76	0.2(6)
C4	C39	C40	C42	-0.6(6)	C74	C75	C76	C77	-179.1(4)
C4	C39	C40	C41	-179.0(5)	C45	C44	C84	C82	-0.4(6)
C40	C42	C2	C3	-0.6(5)	C43	C44	C84	C82	-177.9(5)

Torsion Angles for 2189996 (compound **1e**)

**A**	**B**	**C**	**D**	**Angle/˚**	**A**	**B**	**C**	**D**	**Angle/˚**
C34	C35	C36	C37	1.4(6)	C53	C54	C59	C60	3.5(6)
C11	C12	C17	C18	-1.0(6)	C68	C69	C70	C71	-64.9(5)
C26	C27	C28	C29	-176.4(7)	C55	C56	C57	C58	179.7(6)

3b

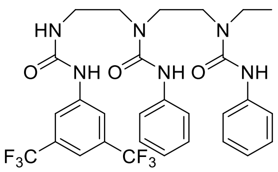

4a

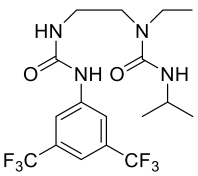

4b

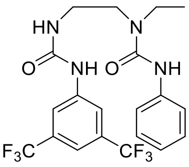

Identification code (**CCDC**)	**3b** (dte126) (**2189998**)	**4a** (s5784l_auto) (**2190003**)	**4b** (g32a) (**2190004**)
Empirical formula	$C_{29}H_{30}F_6N_6O_3$	$C_{17}H_{22}F_6N_4O_2$	$C_{20}H_{20}F_6N_4O_2$
Formula weight	624.59	428.38	462.40
Temperature/K	100(2)	200(2)	100(2)
Crystal system	monoclinic	monoclinic	monoclinic
Space group	$P2_1/c$	$P2_1/n$	$P2_1/c$
a/Å	10.2730(3)	11.6426(4)	13.7401(12)
b/Å	19.1589(4)	11.0107(4)	14.0361(11)
c/Å	15.2648(4)	16.8030(5)	11.5499(10)
α/°	90	90	90
β/°	101.0383(13)	108.437(3)	113.558(3)
γ/°	90	90	90
Volume/Å^3	2948.82(13)	2043.46(12)	2041.8(3)
Z	4	4	4
ρ_{calc}g/cm^3	1.407	1.392	1.504
μ/mm^{-1}	0.119	1.141	0.136
F(000)	1296.0	888.0	952.0
Crystal size/mm^3	0.42 × 0.382 × 0.207	0.558 × 0.377 × 0.328	0.553 × 0.426 × 0.19
Radiation	MoKα (λ = 0.71073)	CuKα (λ = 1.54184)	MoKα (λ = 0.71073)
2θ range for data collection/°	3.45 to 54.968	8.17 to 136.498	3.234 to 51.352
Index ranges	-13 ≤ h ≤ 13, -23 ≤ k ≤ 24, -19 ≤ l ≤ 19	-14 ≤ h ≤ 13, -13 ≤ k ≤ 13, -20 ≤ l ≤ 17	-16 ≤ h ≤ 16, -17 ≤ k ≤ 17, -13 ≤ l ≤ 14
Reflections collected	39105	12890	59432
Independent reflections	6760 [R_{int} = 0.0407, R_{sigma} = 0.0280]	3712 [R_{int} = 0.0279, R_{sigma} = 0.0290]	3866 [R_{int} = 0.1093, R_{sigma} = 0.0507]
Data/restraints/parameters	6760/356/534	3712/181/344	3866/169/323

Goodness-of-fit on F^2	1.012	1.048	1.037
Final R indexes [I>=2σ (I)]	R_1 = 0.0438, wR_2 = 0.1010	R_1 = 0.0509, wR_2 = 0.1300	R_1 = 0.0423, wR_2 = 0.0909
Final R indexes [all data]	R_1 = 0.0645, wR_2 = 0.1132	R_1 = 0.0606, wR_2 = 0.1367	R_1 = 0.0727, wR_2 = 0.1042
Largest diff. peak/hole / e $Å^{-3}$	0.35/-0.34	0.34/-0.28	13.7401(12)

Hydrogen Bonds for 2189998 (compound **3b**)

D	H	A	d(D-H)/Å	d(H-A)/Å	d(D-A)/Å	D-H-A/°
C6	H6	O1	0.9500	2.2607(18)	2.8704(18)	121.21(5)
C18	H18	O2A	0.9500	2.25(3)	2.86(3)	121.6(5)
N1	H1	$O3^1$	0.834(19)	2.111(19)	2.8823(17)	153.6(17)
N2	H2a	$O3^1$	0.86(2)	1.94(2)	2.745(2)	156.3(19)

Torsion Angles for for 2189998 (compound **3b**)

A	B	C	D	Angle/˚	A	B	C	D	Angle/˚
C1	C2	C3	C4	-0.82(19)	C12	N4	C13	C14	156.1(4)
C1	C2	C3	C7	178.30(16)	C12	N4	C13	C18	-22.4(7)
C1	C6	C5	C4	-0.17(18)	C12	N4	C13	N4A	-102(8)
C1	C6	C5	C8	176.40(15)	C12A	N3A	C19A	C20A	-81.0(14)
C1	N1	C9	N2	-172.47(19)	C12A	N4A	C13	C14	-179(4)
C1	N1	C9	N2A	-140.6(7)	C12A	N4A	C13	C18	-6(7)
C1	N1	C9	O1	8.9(2)	C12A	N4A	C13	N4	100(10)
C2	C3	C4	C5	-0.50(19)	C13	C14	C15	C16	0.2(2)
C2	C3	C7	F1	-3.0(2)	C13	C18	C17	C16	0.4(2)
C2	C3	C7	F1A	161.0(10)	C14	C15	C16	C17	-0.4(2)
C2	C3	C7	F2	-122.4(2)	C15	C16	C17	C18	0.2(2)
C2	C3	C7	F2A	45.2(10)	C19	C20	N5	C27	74.85(17)
C2	C3	C7	F3	119.9(2)	C19	C20	N5	C28	-84.80(16)
C2	C3	C7	F3A	-73.9(12)	C19A	C20A	N5A	C27	-57.5(14)
C3	C4	C5	C6	1.00(19)	C19A	C20A	N5A	C28A	107.4(13)
C3	C4	C5	C8	-175.52(15)	C20	N5	C27	N5A	-86.4(8)
C4	C5	C8	F4	94.80(16)	C20	N5	C27	N6	7.5(2)
C4	C5	C8	F5	-23.81(16)	C20	N5	C27	O3	-174.91(17)
C4	C5	C8	F6	-146.32(16)	C20	N5	C28	C29	-78.02(19)
C9	N2	C10	C11	-74.1(2)	C20A	N5A	C27	N5	83.2(14)
C9	N2A	C10A	C11A	50.7(13)	C20A	N5A	C27	N6	-18.6(15)
C10	C11	N3	C12	-84.49(18)	C20A	N5A	C27	O3	-168.1(11)
C10	C11	N3	C19	92.91(16)	C20A	N5A	C28A	C29A	-75.0(16)
C10A	C11A	N3A	C12A	78.0(15)	C21	C22	C23	C24	-0.8(2)
C10A	C11A	N3A	C19A	-99.8(12)	C21	C26	C25	C24	-0.11(19)
C11	N3	C12	N4	1.5(3)	C21	N6	C27	N5	-176.86(17)
C11	N3	C12	O2	-178.9(2)	C21	N6	C27	N5A	-141.9(6)

Torsion Angles for for 2189998 (compound **3b**)

A	B	C	D	Angle/˚	A	B	C	D	Angle/˚
C11	N3	C19	C20	-103.91(17)	C21	N6	C27	O3	5.52(19)
C11A	N3A	C12A	N4A	-4(3)	C22	C23	C24	C25	-0.3(3)
C11A	N3A	C12A	O2A	-177(2)	C23	C24	C25	C26	0.8(2)
C11A	N3A	C19A	C20A	96.9(13)	C27	N5	C28	C29	121.9(2)
C12	N3	C19	C20	73.66(18)	C27	N5A	C28A	C29A	90.5(15)

Hydrogen Bonds for 2190003 (compound **4a**)

D	H	A	d(D-H)/Å	d(H-A)/Å	d(D-A)/Å	D-H-A/°
N1	H1	O2[1]	0.831(15)	1.970(16)	2.787(2)	167(2)

Torsion Angles for 2190003 (compound **4a**)

A	B	C	D	Angle/˚	A	B	C	D	Angle/˚
N1	C6	C5	C4	-178.18(18)	C8	C4	C5	C6	-179.42(19)
N1	C6	C1	C2	178.15(19)	C11	N3	C12	O2	-178.44(16)
N3	C11	C10	N2	-162.01(15)	C11	N3	C12	N4	1.9(3)
C2	C3	C4	C8	179.55(19)	C11	N3	C16	C17	79.9(2)
C2	C3	C4	C5	0.6(3)	C7	C2	C3	C4	178.8(2)
C13	N4	C12	O2	5.4(3)	C7	C2	C1	C6	-179.1(2)
C13	N4	C12	N3	-174.94(18)	C16	N3	C12	O2	-2.9(3)
C9	N1	C6	C5	-170.8(2)	C16	N3	C12	N4	177.49(17)
C9	N1	C6	C1	10.9(3)	C16	N3	C11	C10	-98.1(2)
C9	N2	C10	C11	75.5(2)	C6	N1	C9	O1	3.6(4)
C3	C2	C7	F2	128.5(5)	C6	N1	C9	N2	-177.0(2)
C3	C2	C7	F1	-111.6(5)	C5	C4	C8	F6	-42.6(3)
C3	C2	C7	F3	9.4(6)	C5	C4	C8	F4	-159.0(2)
C3	C2	C7	F1A	-146.1(5)	C5	C4	C8	F5	78.7(3)
C3	C2	C7	F2A	92.5(6)	C5	C4	C8	F6A	-93.6(9)
C3	C2	C7	F3A	-30.7(5)	C5	C4	C8	F4A	146.5(7)
C3	C2	C7	F2B	159.7(6)	C5	C4	C8	F5A	26.3(7)
C3	C2	C7	F1B	-64.5(6)	C5	C6	C1	C2	-0.1(3)
C3	C2	C7	F3B	48.6(5)	C1	C2	C3	C4	-0.5(3)
C3	C2	C1	C6	0.3(3)	C1	C2	C7	F2	-52.1(5)
C3	C4	C8	F6	138.4(2)	C1	C2	C7	F1	67.8(5)
C3	C4	C8	F4	22.1(3)	C1	C2	C7	F3	-171.3(6)
C3	C4	C8	F5	-100.3(3)	C1	C2	C7	F1A	33.2(5)
C3	C4	C8	F6A	87.4(9)	C1	C2	C7	F2A	-88.2(6)
C3	C4	C8	F4A	-32.5(7)	C1	C2	C7	F3A	148.6(4)
C3	C4	C8	F5A	-152.7(7)	C1	C2	C7	F2B	-20.9(7)
C3	C4	C5	C6	-0.5(3)	C1	C2	C7	F1B	114.9(6)
C12	N4	C13	C15	106.5(2)	C1	C2	C7	F3B	-132.1(5)
C12	N4	C13	C14	-130.7(2)	C1	C6	C5	C4	0.2(3)

Torsion Angles for 2190003 (compound **4a**)

A	B	C	D	Angle/˚	A	B	C	D	Angle/˚
C12	N3	C11	C10	77.5(2)	C10	N2	C9	O1	-6.0(3)
C12	N3	C16	C17	-95.8(2)	C10	N2	C9	N1	174.59(18)

Hydrogen Bonds for 2190004 (compound **4b**)

D	H	A	d(D-H)/Å	d(H-A)/Å	d(D-A)/Å	D-H-A/°
N4	H4	O1	0.87(2)	2.04(2)	2.894(2)	168.7(18)
N1	H1	O2[1]	0.90(2)	1.92(2)	2.784(2)	160.3(19)

Torsion Angles for 2190004 (compound **4b**)

A	B	C	D	Angle/˚	A	B	C	D	Angle/˚
F6	C8	C4	C3	164.41(16)	N3	C12	N4	C13	-178.24(15)
F6	C8	C4	C5	-17.38(19)	N4	C13	C14	C15	179.54(16)
F1	C7	C2	C3	-93.9(10)	N4	C13	C18	C17	179.85(17)
F1	C7	C2	C1	85.7(10)	C17	C16	C15	C14	-1.5(2)
F2	C7	C2	C3	154.7(10)	C17	C18	C13	C14	-1.8(2)
F2	C7	C2	C1	-25.7(10)	C3	C4	C5	C6	-0.1(2)
F4	C8	C4	C3	43.70(19)	C3	C2	C1	C6	-0.5(2)
F4	C8	C4	C5	-138.10(16)	C3	C2	C7	F1A	-106.7(5)
F5	C8	C4	C3	-75.12(18)	C3	C2	C7	F2B	162.6(6)
F5	C8	C4	C5	103.09(17)	C3	C2	C7	F3A	7.3(7)
F3	C7	C2	C3	33.8(16)	C3	C2	C7	F1B	-62.1(4)
F3	C7	C2	C1	-146.5(16)	C3	C2	C7	F2A	143.9(7)
O2	C12	N3	C11	173.37(16)	C3	C2	C7	F3B	46.4(3)
O2	C12	N3	C19	-8.9(2)	C1	C2	C7	F1A	72.9(5)
O2	C12	N4	C13	1.2(2)	C1	C2	C7	F2B	-17.8(6)
O1	C9	N2	C10	3.0(2)	C1	C2	C7	F3A	-173.0(7)
O1	C9	N1	C6	1.6(2)	C1	C2	C7	F1B	117.5(4)
N2	C9	N1	C6	-177.32(15)	C1	C2	C7	F2A	-36.5(7)
N2	C10	C11	N3	-152.19(15)	C1	C2	C7	F3B	-134.0(3)
N1	C6	C1	C2	179.77(17)	C1	C6	C5	C4	-0.6(2)
N1	C6	C5	C4	-179.36(18)	C15	C14	C13	C18	1.1(2)

3a

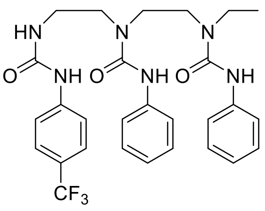

Identification code (**CCDC**)	**3a** (d16) (**2190005**)
Empirical formula	$C_{29}H_{32}Cl_3F_3N_6O_3$

Formula weight	675.95
Temperature/K	100(2)
Crystal system	monoclinic
Space group	*P2₁/n*
a/Å	11.9677(5)
b/Å	20.0581(8)
c/Å	14.2211(5)
α/°	90
β/°	111.923(2)
γ/°	90
Volume/Å^3	3166.9(2)
Z	4
ρ_{calc}g/cm^3	1.418
μ/mm^{-1}	0.348
F(000)	1400.0
Crystal size/mm^3	0.435 × 0.392 × 0.256
Radiation	MoKα (λ = 0.71073)
2θ range for data collection/°	3.812 to 56.328
Index ranges	-15 ≤ h ≤ 15, -12 ≤ k ≤ 26, -18 ≤ l ≤ 18
Reflections collected	29238
Independent reflections	7652 [R_{int} = 0.0670, R_{sigma} = 0.0704]
Data/restraints/parameters	7652/0/414
Goodness-of-fit on F^2	1.048
Final R indexes [I>=2σ (I)]	R_1 = 0.0531, wR_2 = 0.1228
Final R indexes [all data]	R_1 = 0.0875, wR_2 = 0.1392
Largest diff. peak/hole / e Å^{-3}	0.68/-0.63

Hydrogen Bonds for 2190005 (compound **3a**)

D	H	A	d(D-H)/Å	d(H-A)/Å	d(D-A)/Å	D-H-A/°
N5	H5	O1^1	0.85(3)	2.01(3)	2.812(2)	155(3)

Torsion Angles for 2190005 (compound **3a**)

A	B	C	D	Angle/˚	A	B	C	D	Angle/˚
F1	C28	C25	C24	69.4(3)	N3	C19	C20	N5	165.27(19)
F1	C28	C25	C26	-112.3(2)	N4	C13	C14	C15	178.2(2)
F2	C28	C25	C24	-49.5(3)	N4	C13	C18	C17	-178.0(2)
F2	C28	C25	C26	128.9(2)	N5	C21	N6	C22	176.82(19)
F3	C28	C25	C24	-170.8(2)	N6	C22	C23	C24	-175.8(2)
F3	C28	C25	C26	7.5(3)	N6	C22	C27	C26	175.5(2)
O1	C3	N1	C2	-7.9(3)	C4	C5	C6	C7	-1.0(3)
O1	C3	N1	C10	176.8(2)	C4	C9	C8	C7	-1.1(3)

Torsion Angles for 2190005 (compound **3a**)

A	B	C	D	Angle/˚	A	B	C	D	Angle/˚
O1	C3	N2	C4	-0.9(3)	C5	C6	C7	C8	2.3(3)
O2	C12	N3	C11	2.1(3)	C6	C7	C8	C9	-1.2(3)
O2	C12	N3	C19	179.4(2)	C13	C14	C15	C16	0.1(3)
O2	C12	N4	C13	16.3(3)	C13	C18	C17	C16	-0.6(3)
O3	C21	N5	C20	2.6(3)	C14	C15	C16	C17	0.6(3)
O3	C21	N6	C22	-4.0(3)	C15	C16	C17	C18	-0.4(3)
N1	C3	N2	C4	177.66(19)	C22	C23	C24	C25	0.1(3)
N1	C10	C11	N3	-163.33(18)	C22	C27	C26	C25	0.5(3)
N2	C4	C5	C6	-178.6(2)	C23	C24	C25	C26	-2.1(3)
N2	C4	C9	C8	179.8(2)	C23	C24	C25	C28	176.3(2)
N3	C12	N4	C13	-164.17(19)	C24	C25	C26	C27	1.8(3)

Polymeric chains/helices

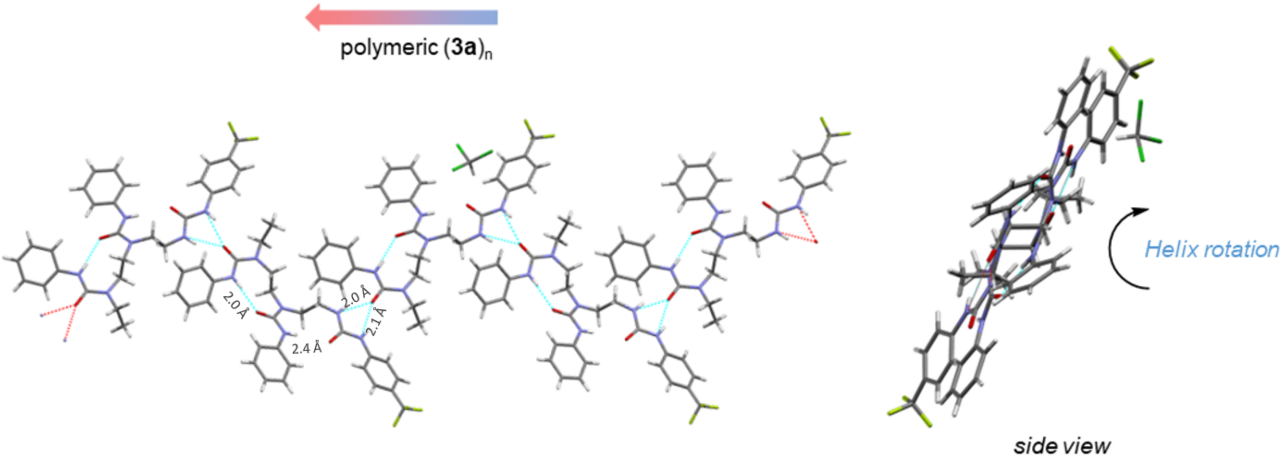

Figure SI 7. Supramolecular chains formed by head-to-tail arrangement of **3a** in the crystal structure.

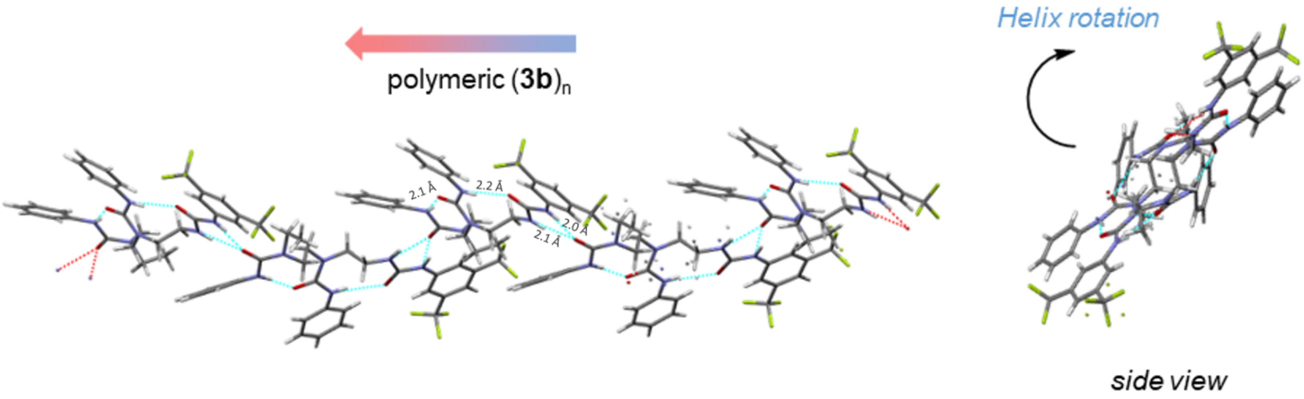

Figure SI 8. Supramolecular chains formed by head-to-tail arrangement of **3b** in the crystal structure.

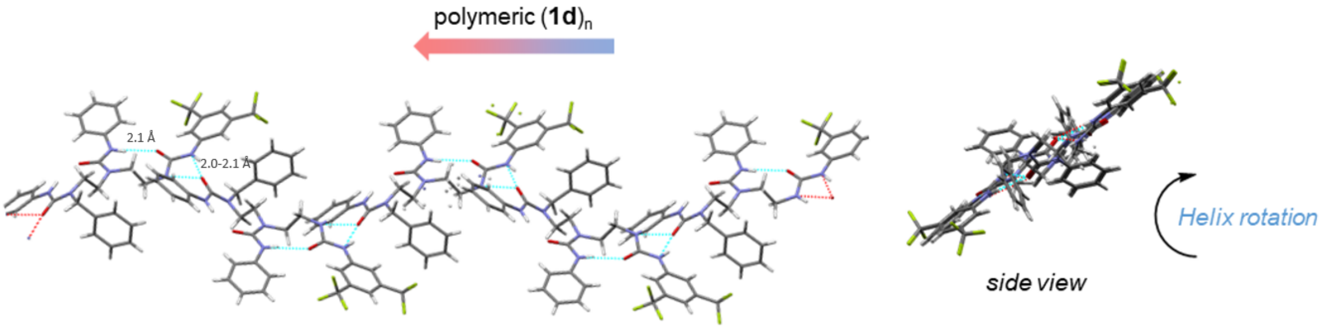

Figure SI 9. Supramolecular chains formed by head-to-tail arrangement of **1d** in the crystal structure.

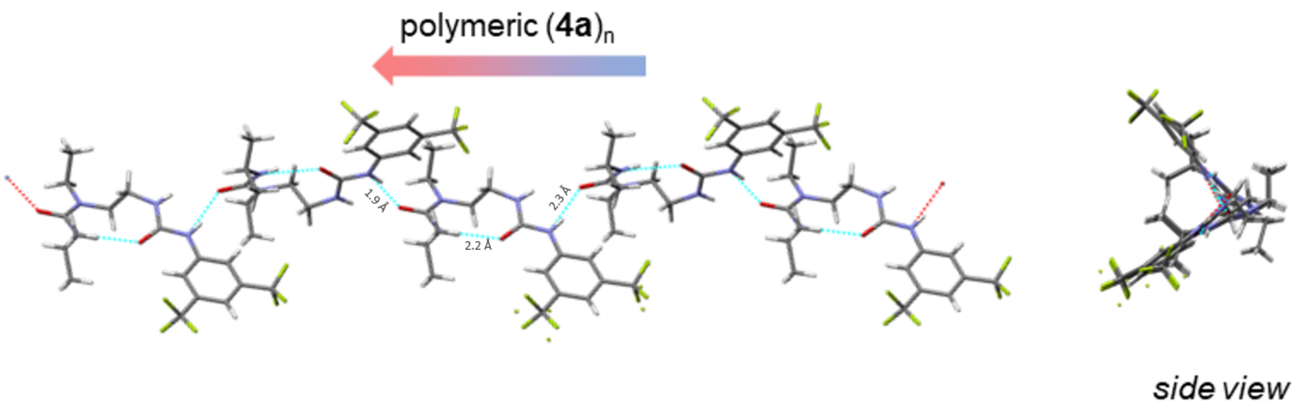

Figure SI 10. Supramolecular chains formed by head-to-tail arrangement of **4a** in the crystal structure.

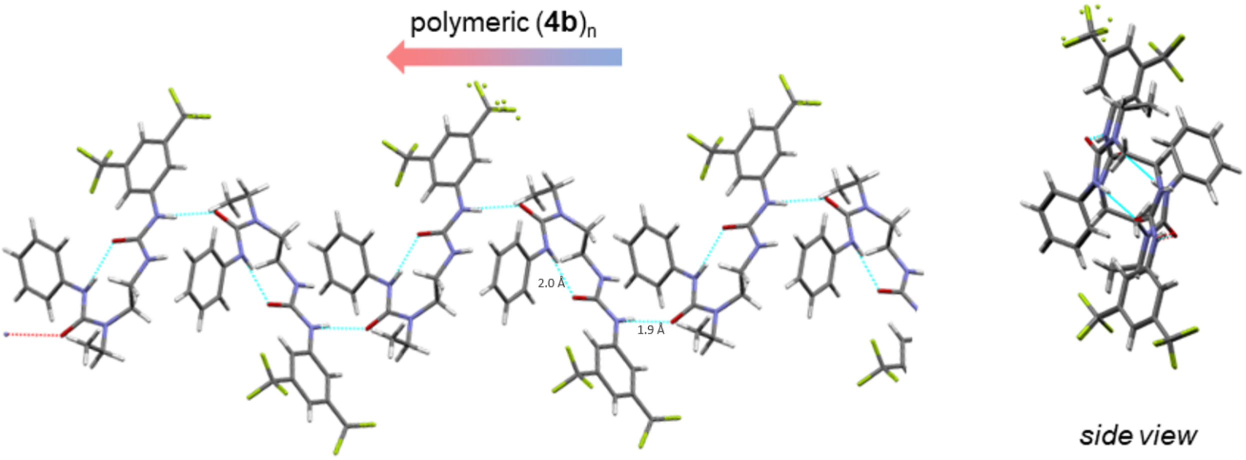

Figure SI 11. Supramolecular chains formed by head-to-tail arrangement of **4b** in the crystal structure.

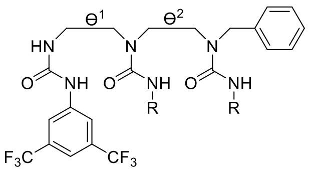

Molecule	Θ^1 (°)	Θ^2 (°)
**1a**	162	159
**1b**[a]	162/161	154/158
**1c**[a]	160/156	160/155
**1d**[a]	159/172	165/163

**2a**	162	161
**2b**	159	163
**2c**	160	
**3a**[a]	163/165	
**3b**	162	
**4a**	162	
**4b**	152	

a) Twin structure

Table SI 1. Measured torsion angles for N-C-C-N bonds of ethylene bridge in the crystal structures.

6. NMR studies

6.1 NMR Spectrometer Data and Pulse Sequences

NMR experiments in this section were performed on a Bruker Avance III HD 500 Cryo equipped with 5 mm DCH 13C–1H/D Cryo Probe (500 MHz). The temperature in the VT experiments was controlled by Bruker BCU II unit. TMS or solvent residual peak (5.32 ppm for CD_2Cl_2) were used as references for ^{1}H and ^{13}C chemical shifts. The spectra were plotted in Mestrenova 14.2.1 or TopSpin 4.1.4 using standard processing methods.

Standard Bruker pulse sequences with the following parameters were used throughout the work.

<u>^{1}H-NMR:</u> zg30; Relaxation delay = 2 s, Acquisition time = 4.36 s, SW = 30.0 ppm, TD = 64k, NS = 8 – 32.

<u>^{13}C{^{1}H}-NMR:</u> Pulse program: zgpg30; Relaxation delay = 1.00 s, Acquisition time = 1.90 s, SW = 248.0 ppm, TD = 120k, NS = 512.

<u>1D selective NOESY:</u> Pulse program: selnogpzs with 180° Gaussian shaped pulse; Relaxation delay = 2.0 s, Acquisition time = 3.21 s, SW = 20.0 ppm, TD = 64k, NS = 128, mixing time D8 = 300 ms.

<u>1D selective ROESY:</u> Pulse program: selrogp with 180° Gaussian shaped pulse; Relaxation delay = 2.0 s, Acquisition time = 3.28 s, SW = 20.0 ppm, TD = 64k, NS = 64 - 128, spinlock time P15 = 200 ms.

<u>^{1}H,^{1}H-COSY:</u> pulse program: cosygpmfppqf; Relaxation delay = 0.92 s, Acquisition time = 0.18 s (F2), SW = 12.0 ppm (F2), 12.0 ppm (F1); TD = 4k (F2), 256 (F1), NS = 2.

<u>^{1}H,^{13}C-HSQC:</u> pulse program: hsqcedetgpsp.3; Relaxation delay = 0.8 s, Acquisition time = 0.07 s (F2), SW = 14.0 ppm (F2), 165 ppm (F1); TD = 1k (F2), 256 (F1), NS = 2, cnst2 = 145 Hz.

<u>^{1}H,^{13}C-HMBC:</u> Pulse program: hmbcetgpl3nd; Relaxation delay = 1.5 s, Acquisition time = 0.27 s, SW = 15.0 ppm (F2), 240 ppm (F1), TD = 4k (F2), 512 (F1), NS = 4, cnst13 = 8 Hz.

<u>^{1}H,^{1}H-ROESY:</u> Pulse program: roesyadjsphpp; Relaxation delay = 2.5 s, Acquisition time = 0.14 s, SW = 15 ppm (F2), 15 ppm (F1), TD =6k (F2), 256 (F1), NS = 8, spinlock time = 200 ms.

<u>^{1}H DOSY:</u> Pulse program ledbpgp2s. Relaxation delay = 2 s, Acquisition time = 2.18 s, SW = 30.0 ppm, 16 experiments with variable gradient strength (5 – 95 %), DS = 4, NS = 16, P30 = δ/2 = 1.8 ms, D20 = 10 – 40 ms.

<u>^{1}H DOSY with convection compensation:</u> Pulse program dstebpgp3s. Relaxation delay = 5 s, Acquisition time = 2.18 s, SW = 30.0 ppm, 16 experiments with variable gradient strength (5 – 95 % using quadratic ramp), DS = 4, NS = 16, P30 = δ/2 = 1.8 ms, D20 = 20 – 30 ms.

6.2 Structural Idenfication

Assignment of Chemical Shifts

^{1}H NMR (500 MHz, CD_2Cl_2, 248 K):

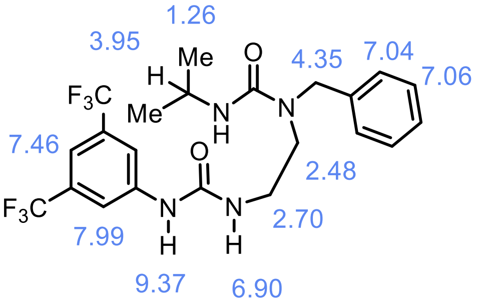

$^{13}C\{^{1}H\}$ NMR (125 MHz, CD_2Cl_2, 248 K):

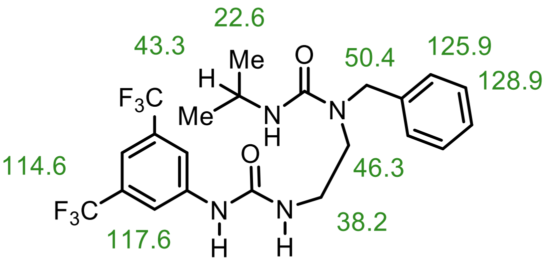

Figure SI 12. Assignment of ^{1}H and ^{13}C NMR shifts (500 MHz, CD_2Cl_2, 32 mM) of **2a** at 248 K.

^{1}H NMR (500 MHz,acetone-d_6, 298 K):

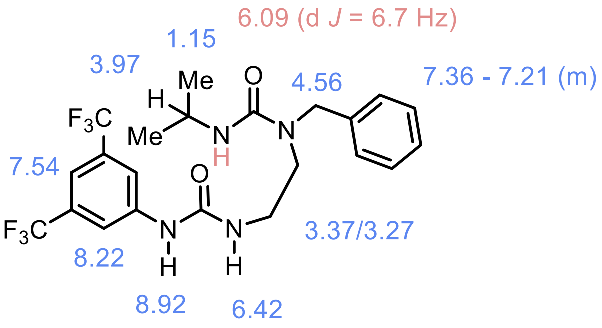

Figure SI 13. Assignment of ^{1}H NMR shifts (500 MHz, acetone-d_6, 32 mM) of **2a** at 298 K.

^{1}H NMR (500 MHz, CD_2Cl_2, 258 K):

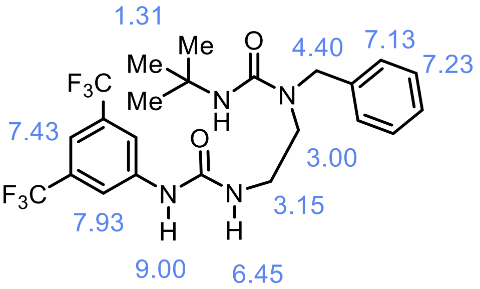

$^{13}C\{^{1}H\}$ NMR (125 MHz, CD_2Cl_2, 258 K):

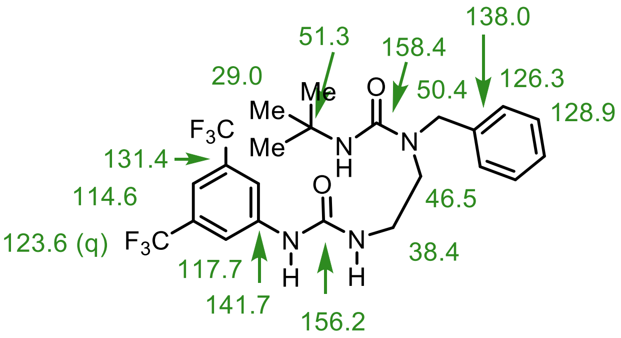

Figure SI 14. Assignment of ^{1}H and ^{13}C NMR shifts (500 MHz, CD_2Cl_2, 52 mM) of **2b** at 258 K.

6.3 NMR Spectra at variable temperature and varying solvents

Compound 2a

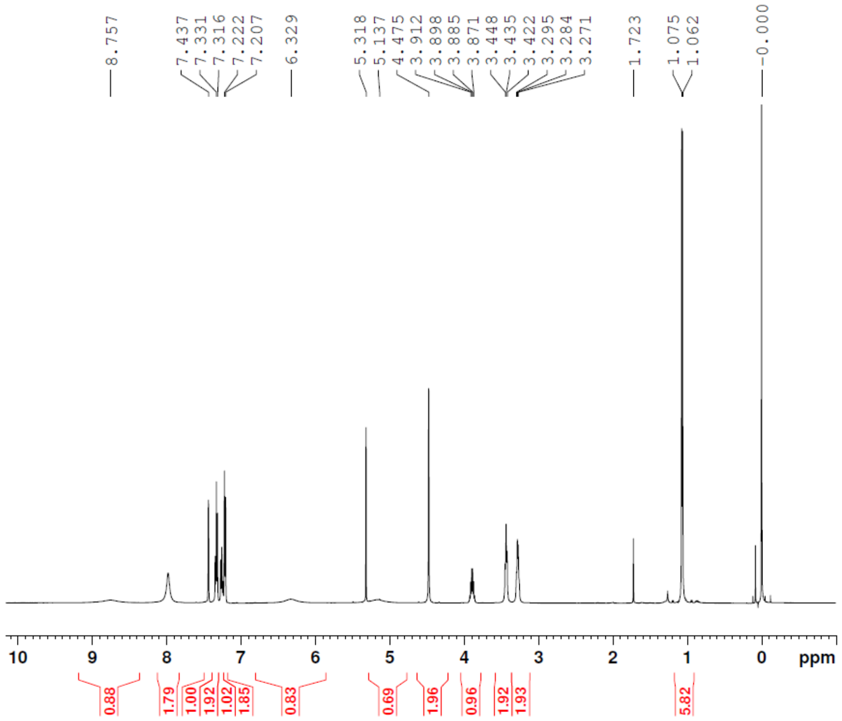

Figure SI 15. ^{1}H NMR spectrum (500 MHz, CD_2Cl_2, 32 mM) of **2a** at 298 K.

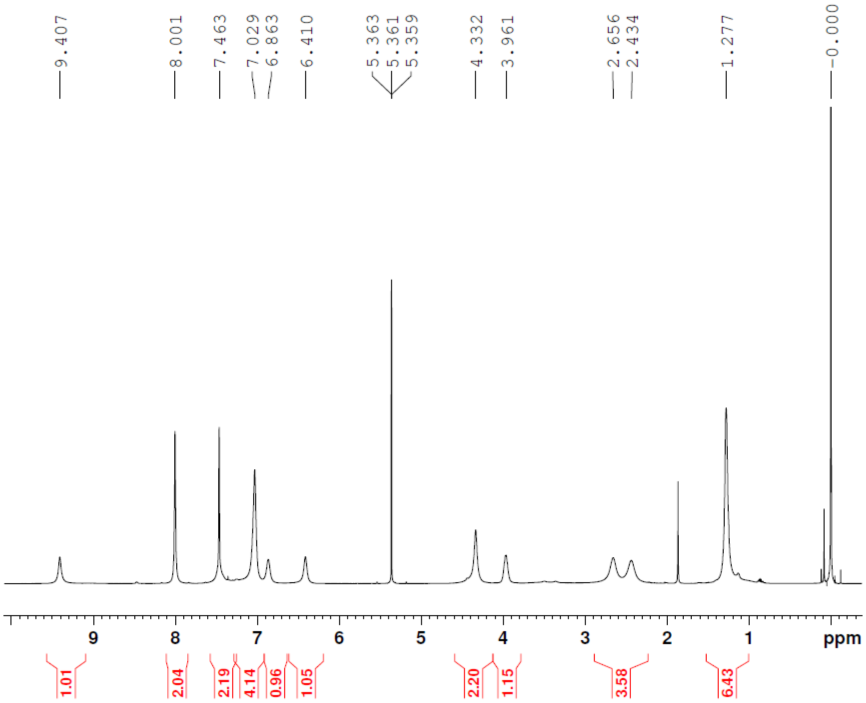

Figure SI 16. ^{1}H NMR spectrum (500 MHz, CD_2Cl_2, 32 mM) of **2a** at 248 K.

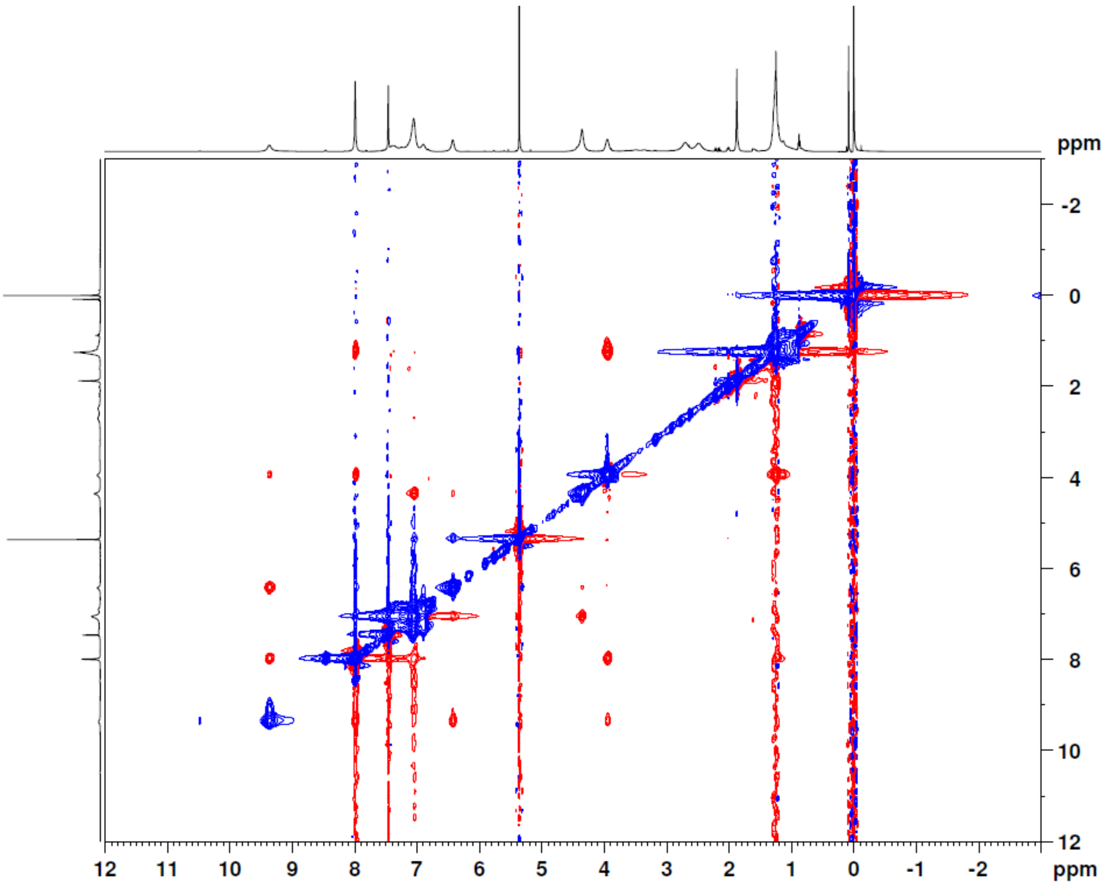

Figure SI 17. 2D ROESY spectrum (500 MHz, CD_2Cl_2) of **2a** at 248 K.

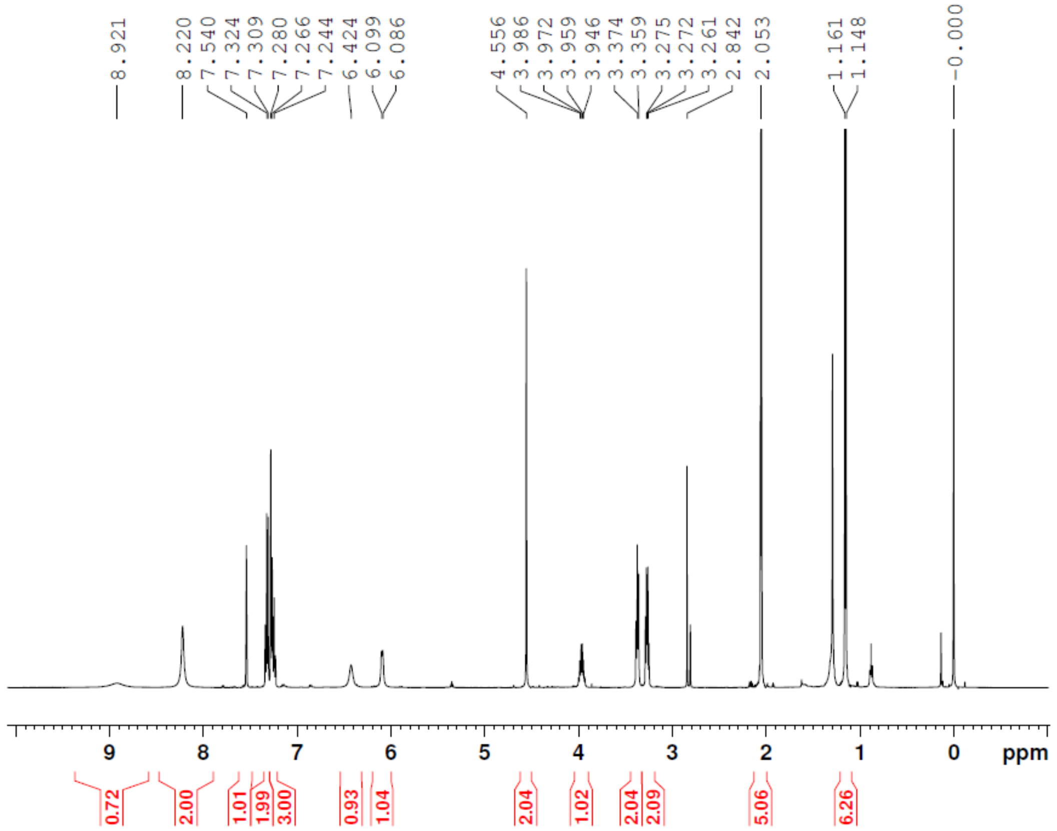

Figure SI 18. ^{1}H NMR spectrum (500 MHz, acetone-d_6, 25mM) of **2a** at 298 K.

Compound 2b

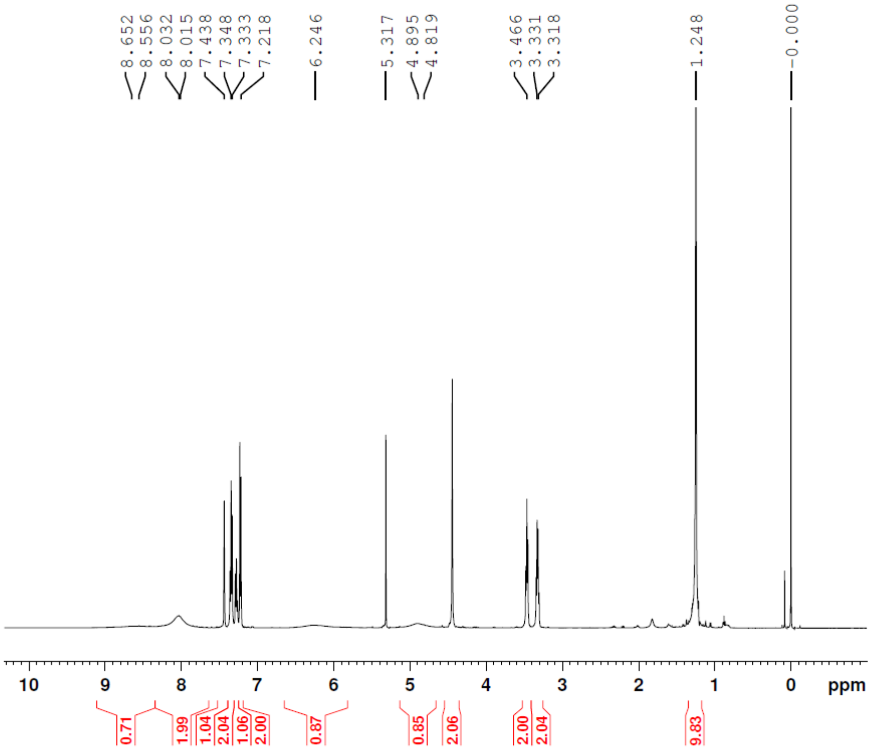

Figure SI 19. ^{1}H NMR spectrum (500 MHz, CD_2Cl_2, 52 mM) of **2b** at 298 K.

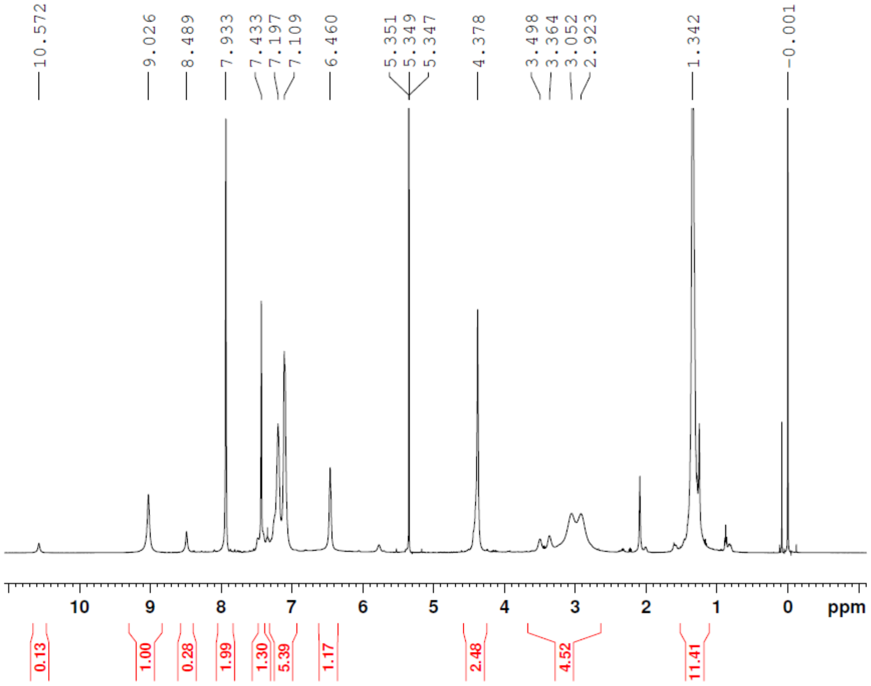

Figure SI 20. ^{1}H NMR spectrum (500 MHz, CD_2Cl_2, 52 mM) of **2b** at 258 K.

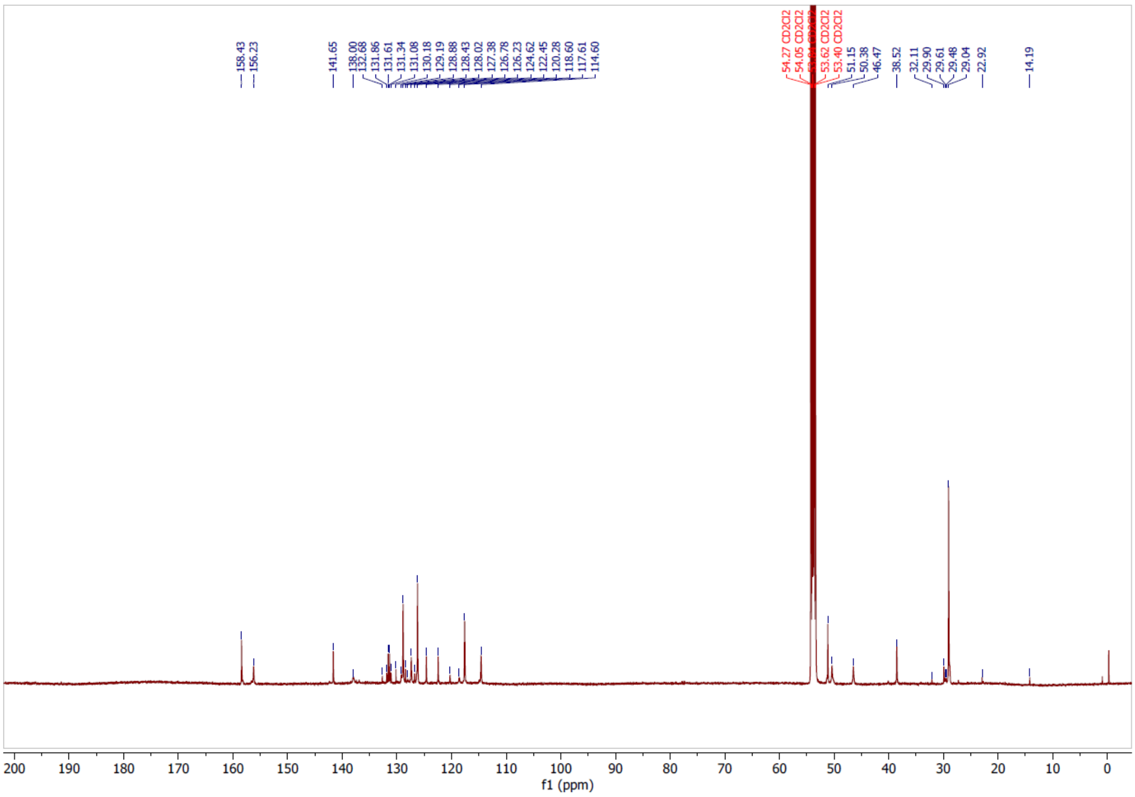

Figure SI 21. ^{13}C NMR spectrum (126 MHz, CD_2Cl_2, 52 mM) of **2b** at 258 K.

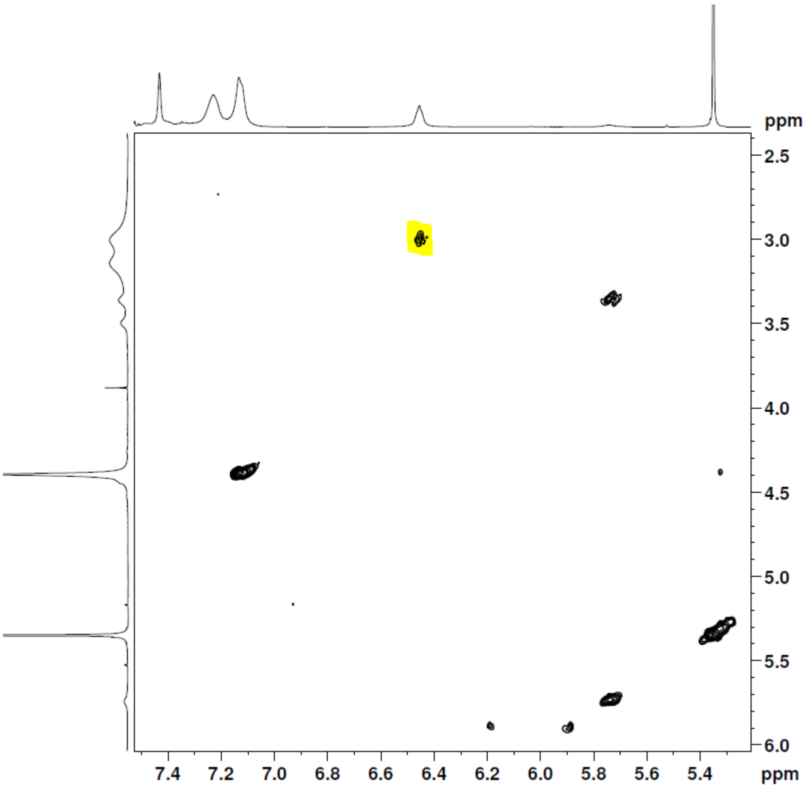

Figure SI 22. ^{1}H,^{1}H-COSY spectrum (500 MHz, CD_2Cl_2, 52 mM) of **2b** at 258 K, showing a correlation (in yellow) between the upfield aryl urea NH (δ_H 6.45 ppm) and ethylene bridge (δ_H 3.00 ppm).

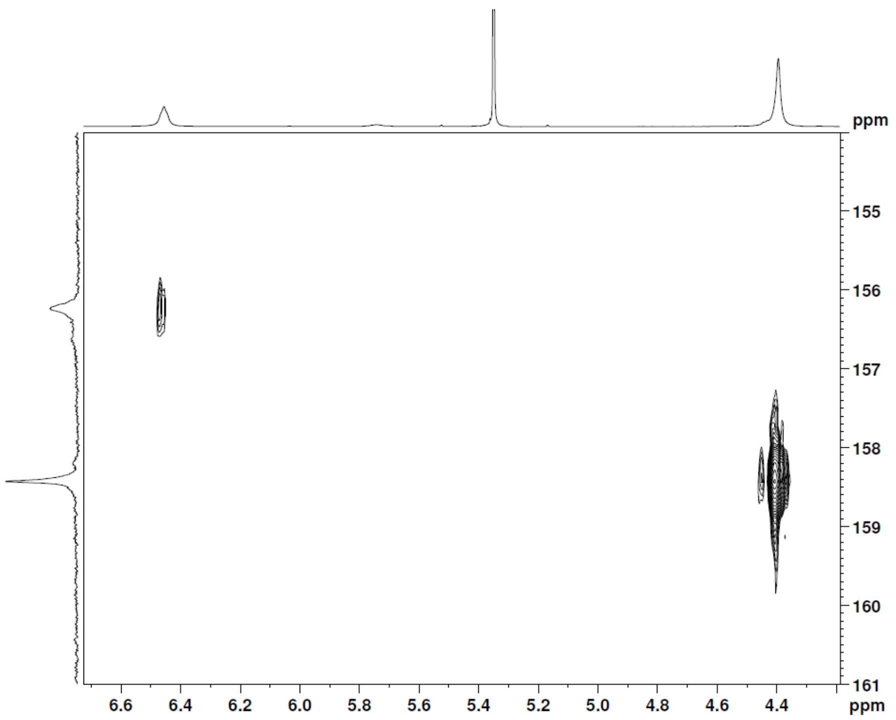

Figure SI 23. ^{1}H, ^{13}C-HMBC spectrum (CD_2Cl_2, 52 mM) of **2b** at 258 K, showing a correlation between the upfield aryl urea NH (δ_H 6.45 ppm) aryl urea carbonyl (δ_C 156.2 ppm); as well as a correlation between benzylic protons (δ_H 4.40 ppm) and alkyl urea carbonyl (δ_C 158.4 ppm)

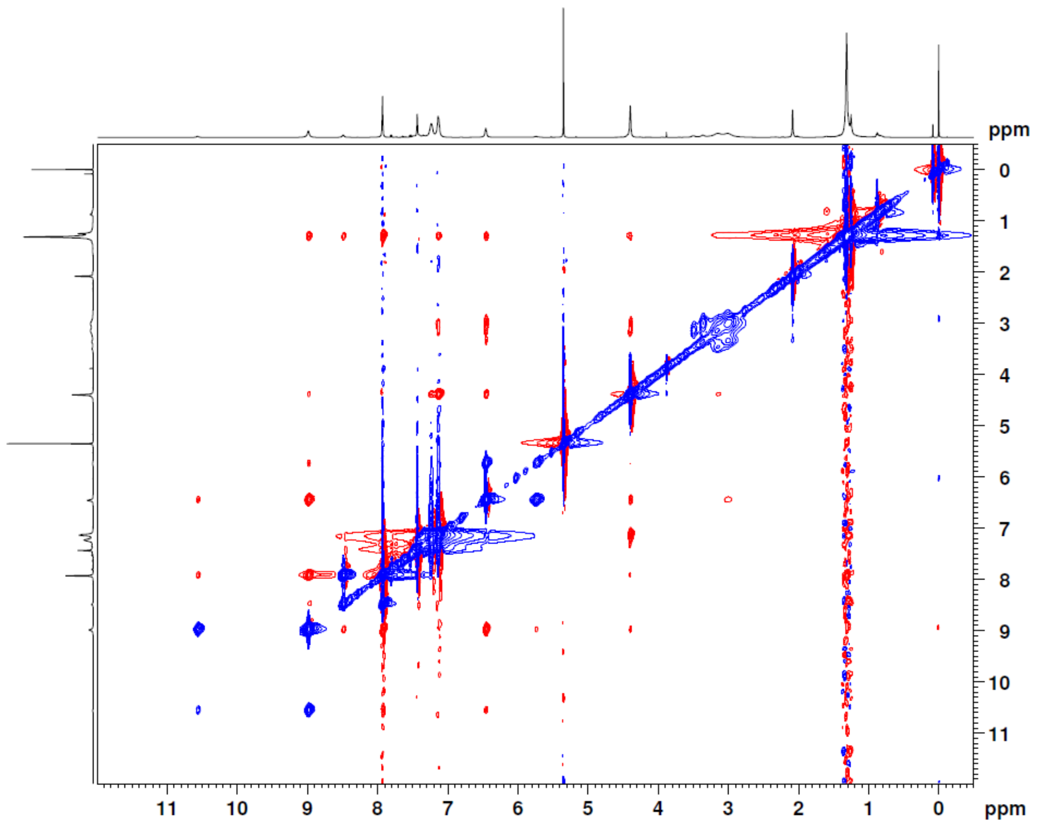

Figure SI 24. 2D ROESY spectrum (500 MHz, CD_2Cl_2) of **2b** at 258 K.

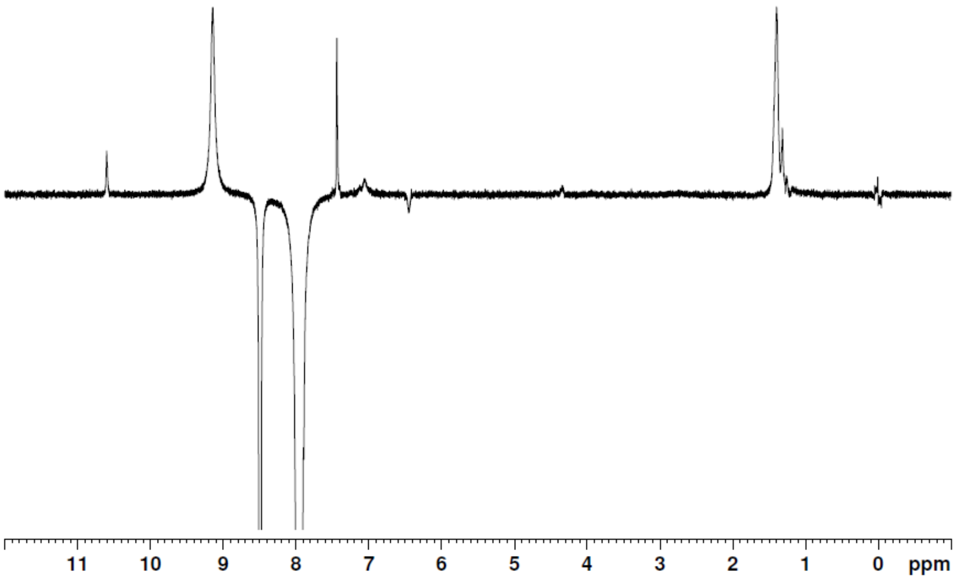

Figure SI 25. 1D ROESY spectrum (500 MHz, CD_2Cl_2) of **2b** at 248 K. Signal at 7.93 ppm was irradiated (aryl urea CH), showing exchange with a intra H-bonded monomer, as well as through-space correlations with aryl urea NH and *t*-Bu group.

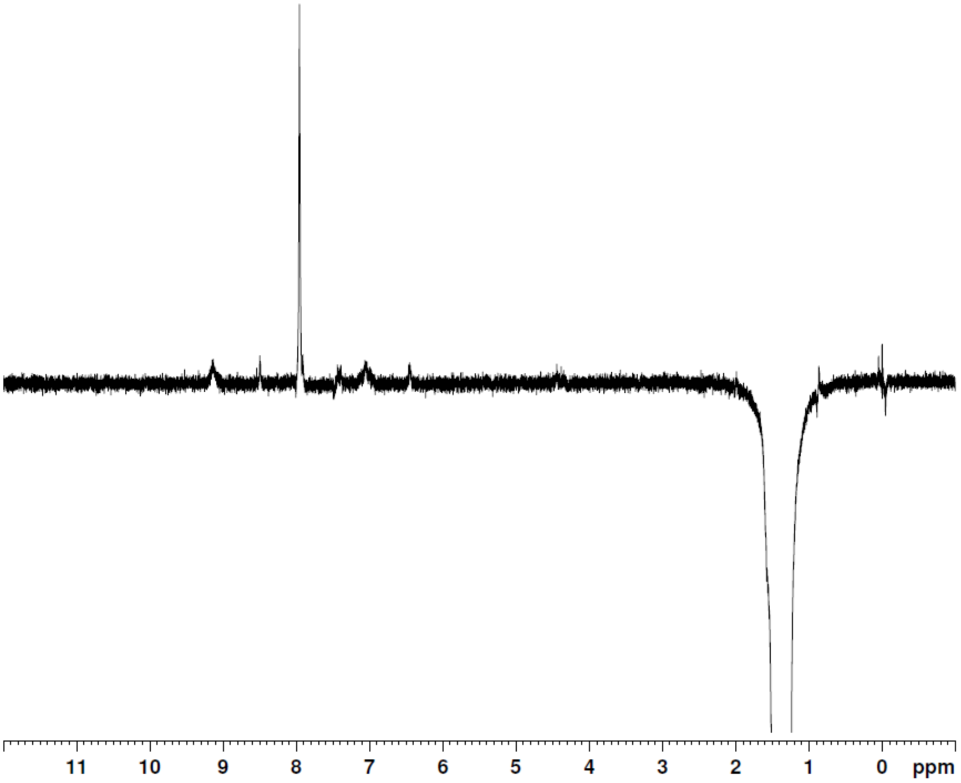

Figure SI 26. 1D ROESY spectrum (500 MHz, CD_2Cl_2) of **2b** at 248 K. Signal at 1.25 ppm was irradiated (*t*-Bu group), showing through-space correlation with aryl urea NHs and $(CF_3)_2C_6H_3$-aryl ring.

6.4 Variable Temperature (VT) NMR

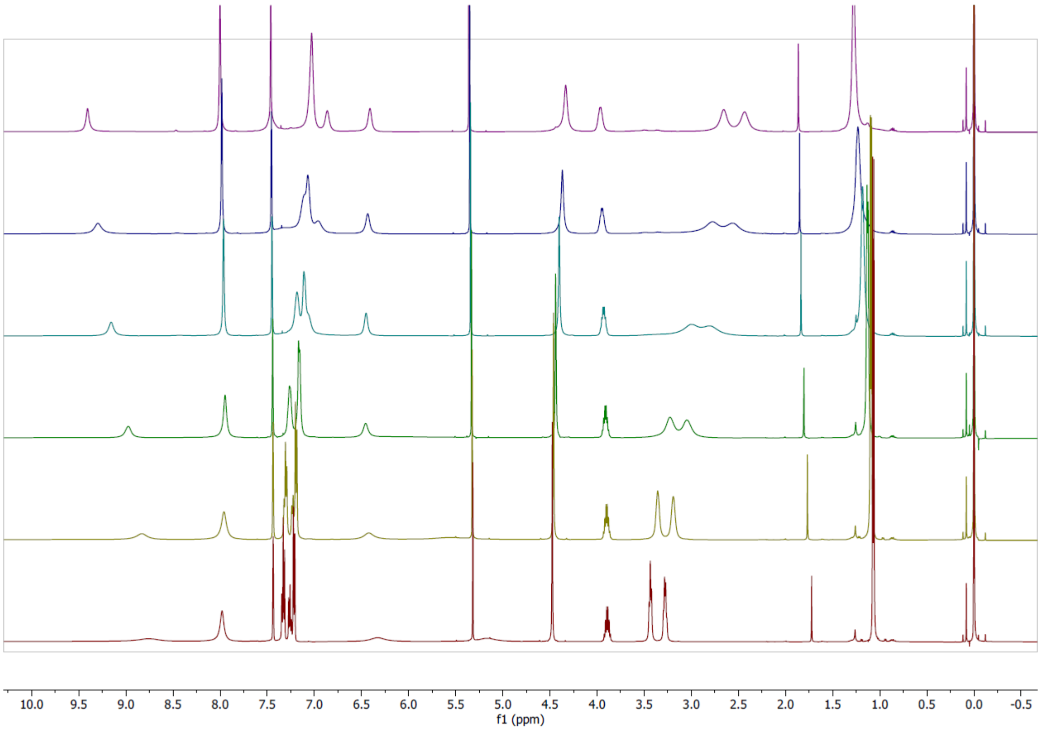

Figure SI 27. Variable temperature ^{1}H NMR spectra of **2a** (32 mM, CD_2Cl_2) at different temperatures (bottom: 298 K, top: 248 K in 10 K increments).

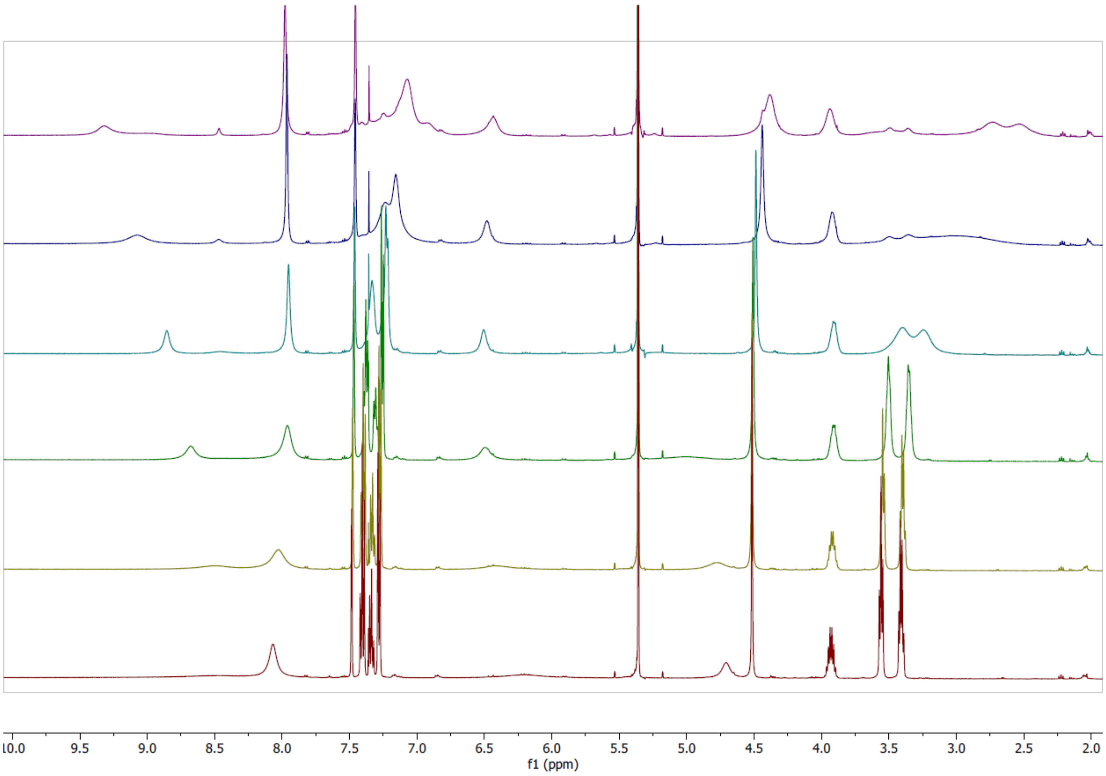

Figure SI 28. Variable temperature ^{1}H NMR spectra of **2a** (6.4 mM, CD_2Cl_2) at different temperatures (bottom: 298 K, top: 248 K in 10 K increments).

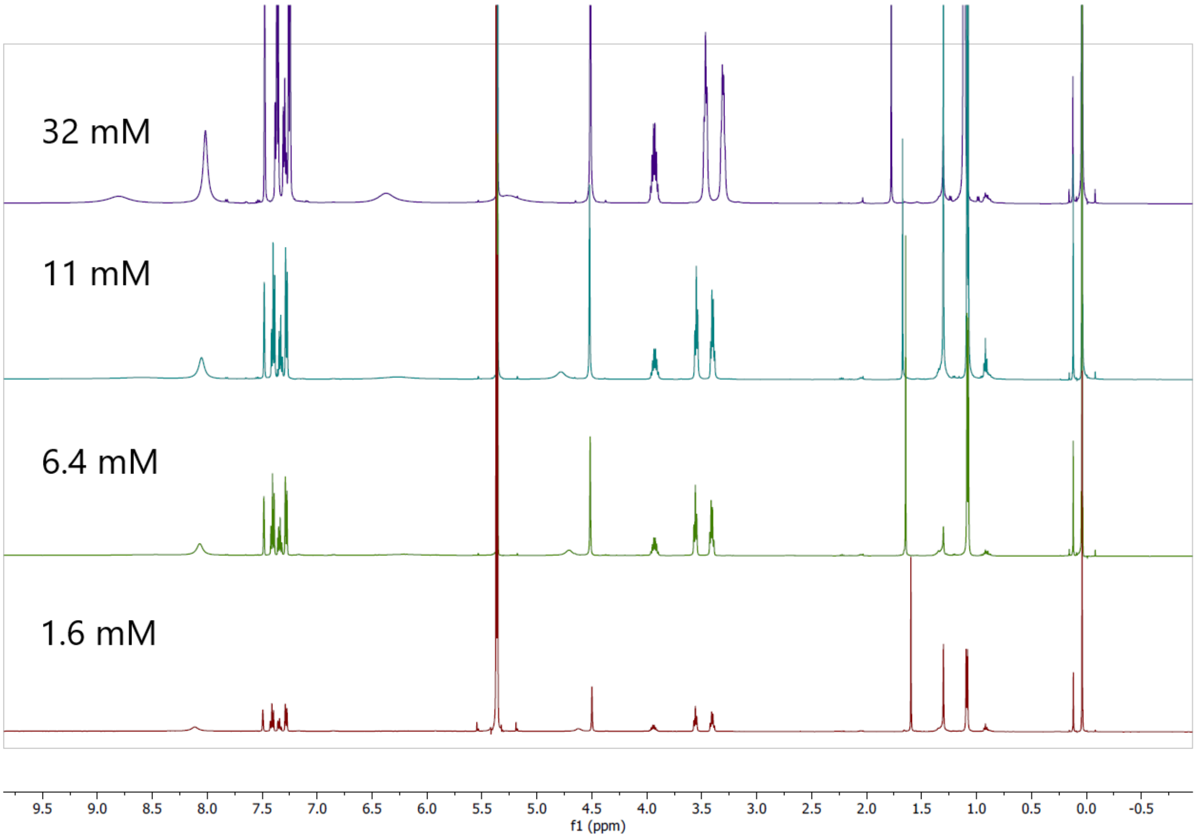

Figure SI 29. ^{1}H NMR spectra of **2a** (298 K, CD_2Cl_2) at different concentrations.

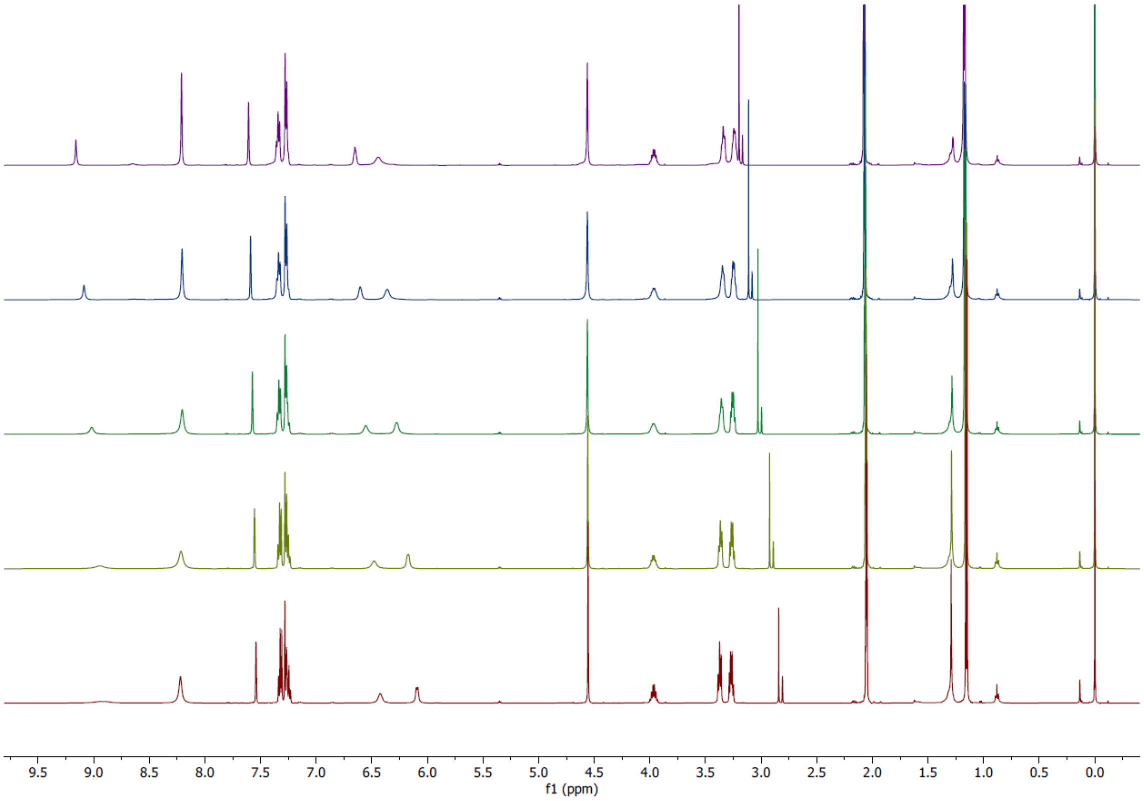

Figure SI 30. Variable temperature ^{1}H NMR spectra of **2a** (25 mM, acetone-d_6) at different temperatures (bottom: 298 K, top: 258 K in 10 K increments).

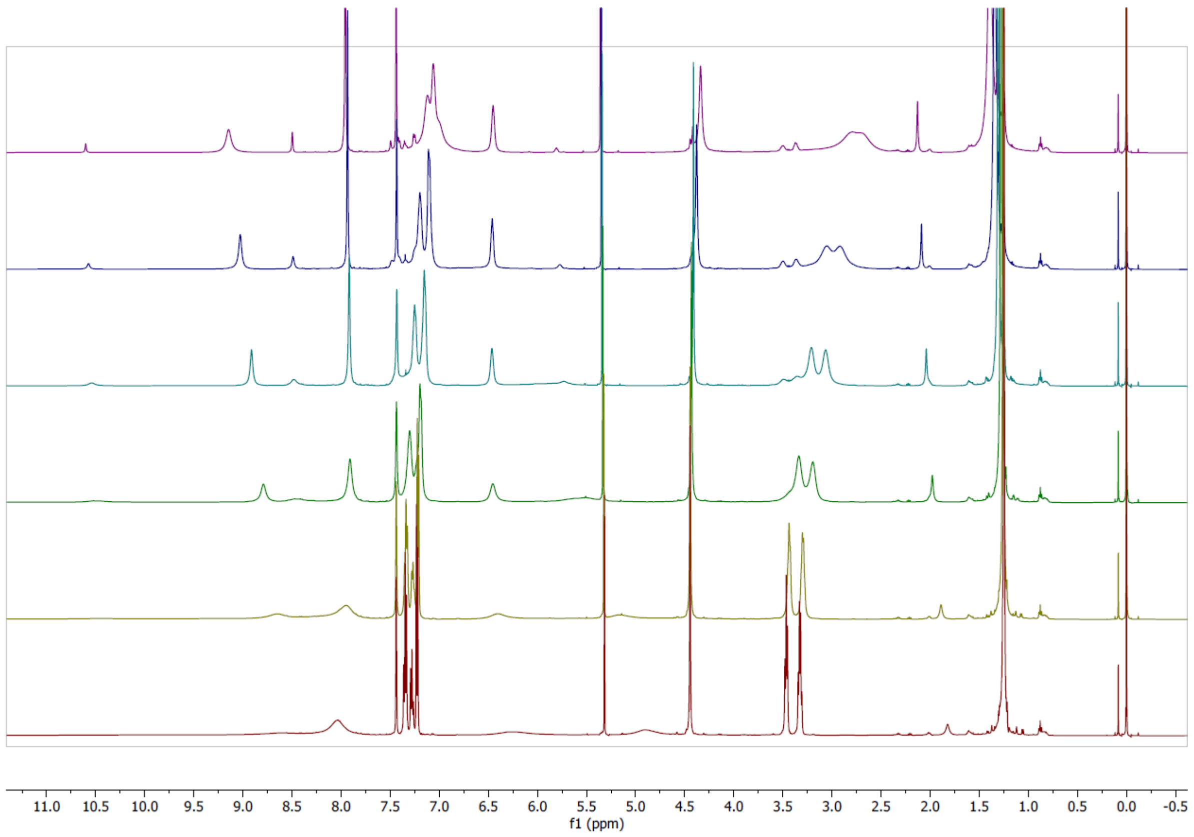

Figure SI 31. Variable temperature ^{1}H NMR spectra of **2b** (52 mM, CD_2Cl_2) at different temperatures (bottom: 298 K, top: 248 K in 10 K increments).

6.5 DOSY Experiments

The ^{1}H diffusion measurements[2] were performed in CD_2Cl_2 or acetone-d_6 with the DSTE (double stimulated echo) pulse sequence using LED, convection compensation, and bipolar gradients, developed by Müller and Jerschow.[3] For the *z*-gradient strengths, values of 100, -13.17, -17.13 and -15.37% were used. Tetramethylsilane (TMS) was added as reference for the ^{1}H chemical shifts and for temperature and was used for viscosity corrections of the diffusion coefficients of the analytes.[4] The solvent residual peak represented additional internal reference. Diffusion delay (d20) was optimized for the urea molecules. Concentration-dependent DOSY measurements were done at 288 K, further away from the boiling point of DCM.

Signal intensities were fitted to the Stejskal-Tanner equation in Mestrenova 14.2.1 (or TopSpin 4.1.4 T1/T2 relaxation module for the ledbpgp2s pulse sequence). Typically, a well-separated benzylic CH_2 peak (singlet) of the urea was chosen for the fitting. In concentrated samples, the TMS peak was often distorted.

^{1}H DOSY: Pulse program ledbpgp2s. Relaxation delay = 2 s, Acquisition time = 2.18 s, SW = 30.0 ppm, 16 experiments with variable gradient strength (5 – 95%), DS = 4, NS = 16, P30 = δ/2 = 1.8 ms, D20 = 10 – 40 ms.

^{1}H DOSY with convection compensation: Pulse program dstebpgp3s. Relaxation delay = 5 s, Acquisition time = 2.18 s, SW = 30.0 ppm, 16 experiments with variable gradient strength (5 – 95% using quadratic ramp), DS = 4, NS = 16, P30 = δ/2 = 1.8 ms, D20 = 20 – 30 ms.

The estimated molecular weight of the urea was calculated:[5]

$$M_A = \left(\frac{D_{ref}}{D_A}\right)^{1.72} M_{ref}$$

Hydrodynamic radius was calculated using viscosity correction from the standard as follows:

$$c(Chen) = \frac{6F}{1 + 0.695(\frac{r_{solv}}{r_{ref}})^{2.234}} ; F = 1\ for\ spheres$$

$$\eta = \frac{kT(1 + 0.695\left(\frac{r_{solv}}{r_{ref}}\right)^{2.234})}{6\pi D_{ref} r_{ref}}$$

$$D[m^2/s] = \frac{kT}{Fc\pi\eta \boldsymbol{r_A}}$$

r_{solv} = 2.46 Å; r_{ref} = 2.96 Å.

Diffusion Data

Concentration (288 K)	***D(TMS)***	***D($CHDCl_2$)***
32 mM	2.47E-09	3.28E-09
11 mM	2.45E-09	3.29E-09
6.4 mM	2.48E-09	3.35E-09
1.3 mM	2.45E-09	3.36E-09
Lit. (298 K)[5]	2.37E-9	3.47E-9

Table SI2. Diffusion coefficients of TMS and $CHDCl_2$ (500 MHz, CD_2Cl_2, 288 K) at different sample concentrations showing the reliability of the convection-compensated pulse sequence and reproducibility of the data.

**[urea] [mM]**	***T* [K]**	**D (urea) [m^2s^{-1}]**	**D (TMS) [m^2s^{-1}]**	**viscosity**	**MW** [g·mol^{-1}]	**r_A** [Å]	**D ($CHDCl_2$) [m^2s^{-1}]**	**viscosity**	**MW** [g·mol^{-1}]	**r_A** [Å]
32	**288**	6.82E-10	2.47E-09	4.21E-14	806.9	10.72	3.28E-09	4.43E-14	1280.4	10.19
18	**288**	7.89E-10	2.43E-09	4.28E-14	610.6	9.12	3.26E-09	4.46E-14	986.1	8.75
11	**288**	8.41E-10	2.45E-09	4.24E-14	554.9	8.62	3.29E-09	4.42E-14	897.6	8.29
6.4	**288**	8.94E-10	2.48E-09	4.19E-14	510.1	8.21	3.35E-09	4.34E-14	833.5	7.94
1.3	**288**	9.52E-10	2.45E-09	4.24E-14	448.3	7.62	3.36E-09	4.32E-14	752.0	7.48

Table SI 3. Diffusion data for **2a** in CD_2Cl_2 at 288 K for different concentrations.

[urea] [mM]	*T* [K]	D (urea) [m²s⁻¹]	D (TMS) [m²s⁻¹]	viscosity	MW [g·mol⁻¹]	r_A [Å]	D ($CHDCl_2$) [m²s⁻¹]	viscosity	MW [g·mol⁻¹]	r_A [Å]
6.4	**288**	8.94E-10	2.48E-09	4.19E-14	510.1	8.21	3.35E-09	4.34E-14	833.5	7.94
6.4	**278**	7.43E-10	2.17E-09	4.63E-14	557.3	8.64	2.86E-09	4.90E-14	873.0	8.15
6.4	**268**	6.02E-10	1.88E-09	5.15E-14	625.3	9.24	2.49E-09	5.43E-14	987.9	8.76
6.4	**258**	4.71E-10	1.68E-09	5.54E-14	786.0	10.56	2.22E-09	5.86E-14	1236.7	9.99
6.4	**248**	3.50E-10	1.40E-09	6.4E-14	957.2	11.84	1.85E-09	6.76E-14	1506.2	11.20

Table SI 4. Diffusion data for **2a** in CD_2Cl_2 for different temperatures.

[urea] [mM]	*T* [K]	r_A [Å]	V [Å³]	*N*
6.4	**288**	7.94	2095.52	1.26
6.4	**278**	8.15	2271.44	1.36
6.4	**268**	8.76	2818.24	1.69
6.4	**258**	9.99	4170.29	2.50
6.4	**248**	11.20	5881.40	3.53

Table SI 5. Extracted hydrodynamic radius of **2a** in CD_2Cl_2 at different temperatures, volume of the corresponding sphere ($4/3\pi r^3$), and aggregation number *N* (V/V^0), where V^0 is the calculated volume of the monomer using the extrapolated radius (7.35 Å).

[urea] [mM]	*T* [K]	MW [g·mol⁻¹]	*N*	*N(N-1)*
1.3	**288**	751.96	1.53	0.82
6.4	**288**	833.53	1.70	1.19
11	**288**	897.58	1.83	1.52
32	**288**	1280.38	2.61	4.21

Table SI 6. Extracted molecular mass of **2a** in CD_2Cl_2 at different concentrations. Aggregation number $N = MW/490$ g.mol⁻¹.

[urea] [mM]	*T* [K]	r_A [Å]	V [Å³]	*N*	*N(N-1)*
1.3	**288**	7.62	1851.6	1.11	0.12
6.4	**288**	8.21	2319.0	1.39	0.55
11	**288**	8.62	2685.8	1.61	0.99
18	**288**	9.12	3173.6	1.90	1.72
32	**288**	10.72	5160.6	3.10	6.50

Table SI 7. Extracted hydrodynamic radius of **2a** in CD_2Cl_2 (referenced to TMS) at different concentrations. Aggregation number *N* (V/V^0), where V^0 (1666 Å³) is the calculated volume of the monomer using the extrapolated radius (7.35 Å).

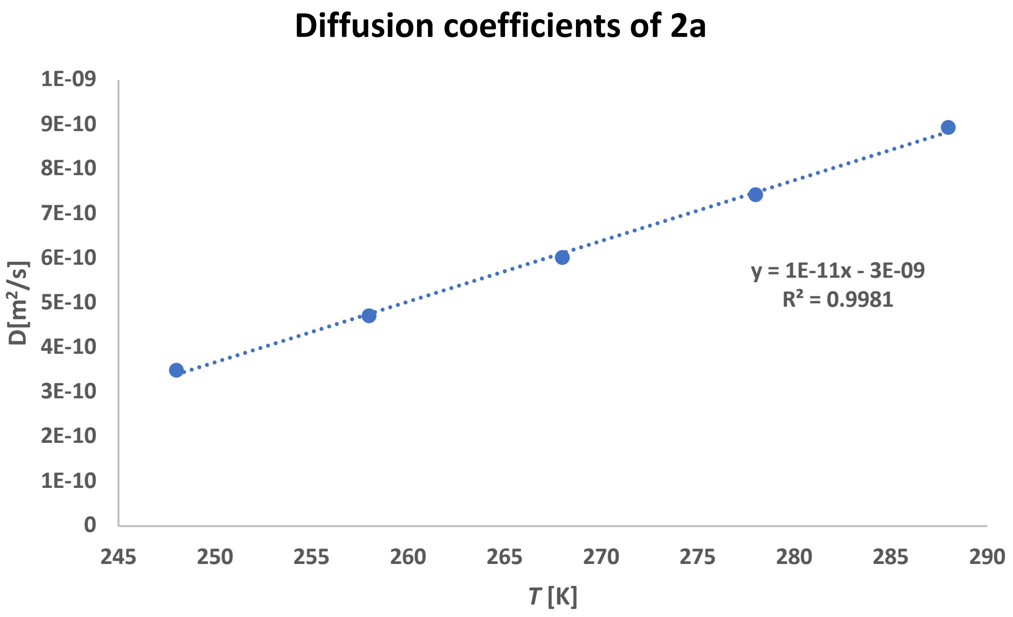

Figure SI 32. Diffusion coefficients of **2a** (6.4 mM, CD_2Cl_2) at different temperatures showing a linear dependence.

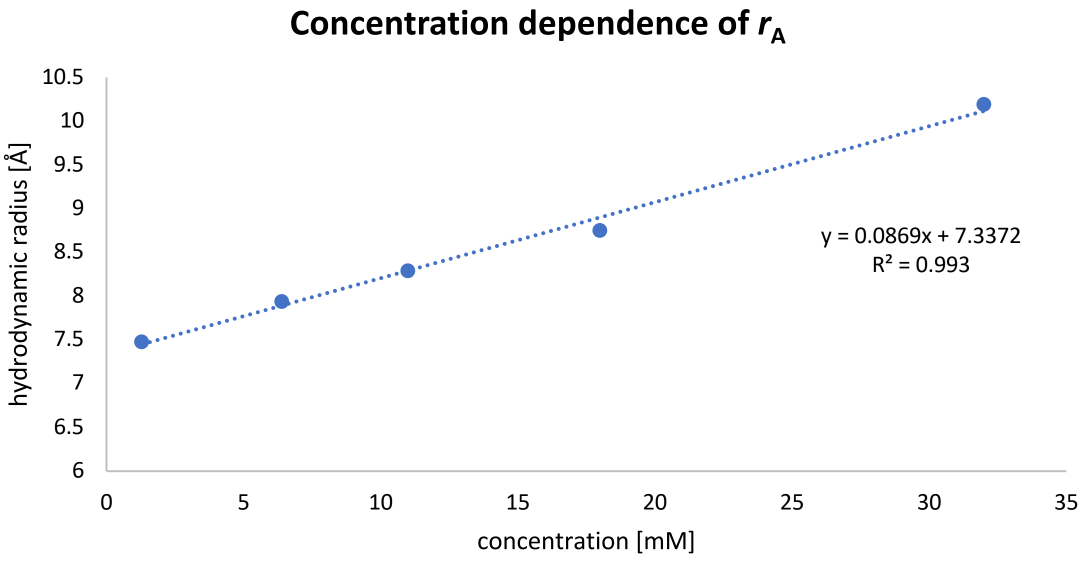

Figure SI 33. Concentration dependence of extracted hydrodynamic radii of **2a** in CD_2Cl_2 at 288 K.

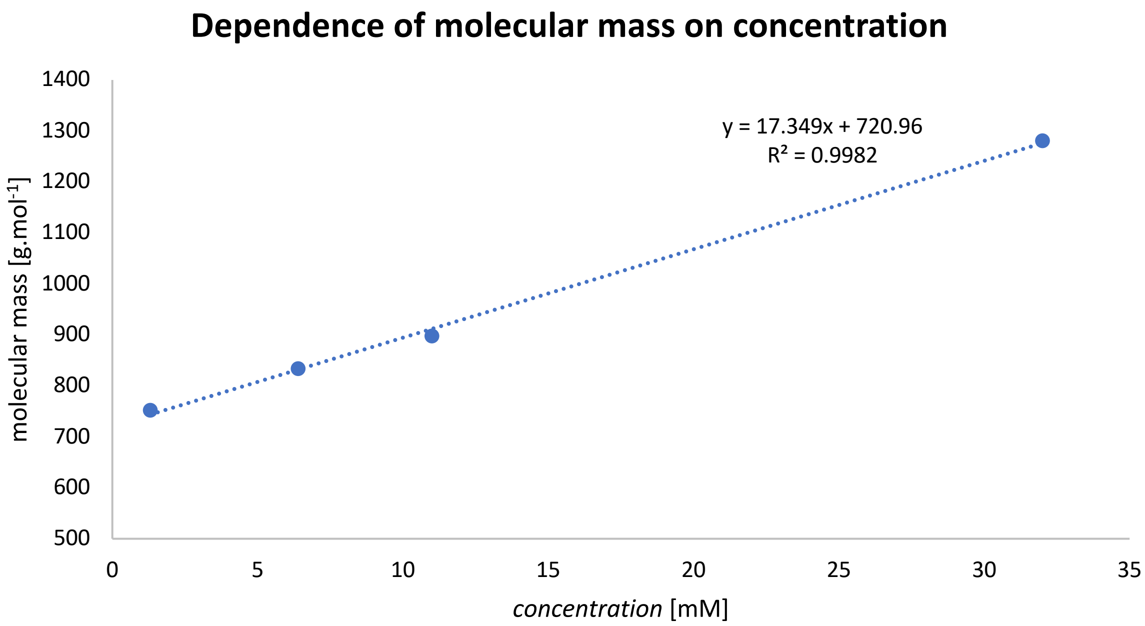

Figure SI 34. Concentration dependence of extracted molecular mass of **2a** in CD_2Cl at 288 K.

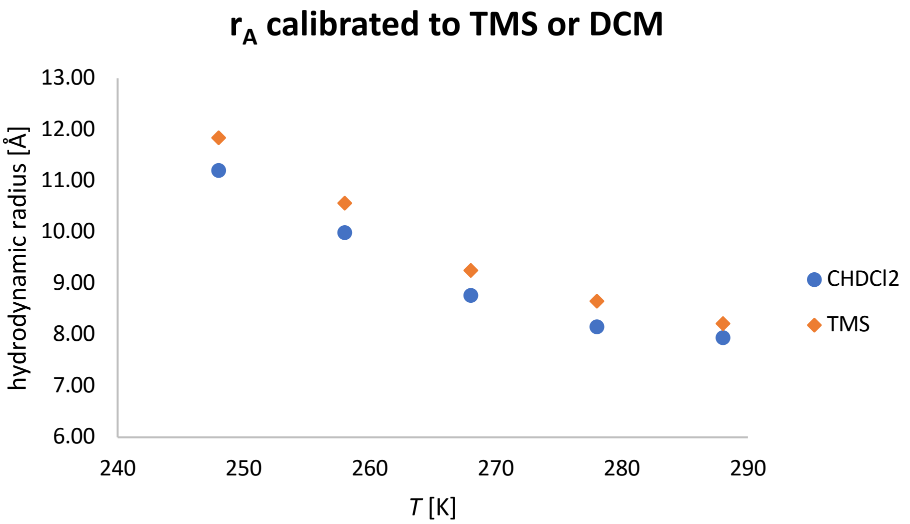

Figure SI 35. Dependence of extracted hydrodynamic radii of **2a** on temperature in CD_2Cl_2 at 6.4 mM concentration.

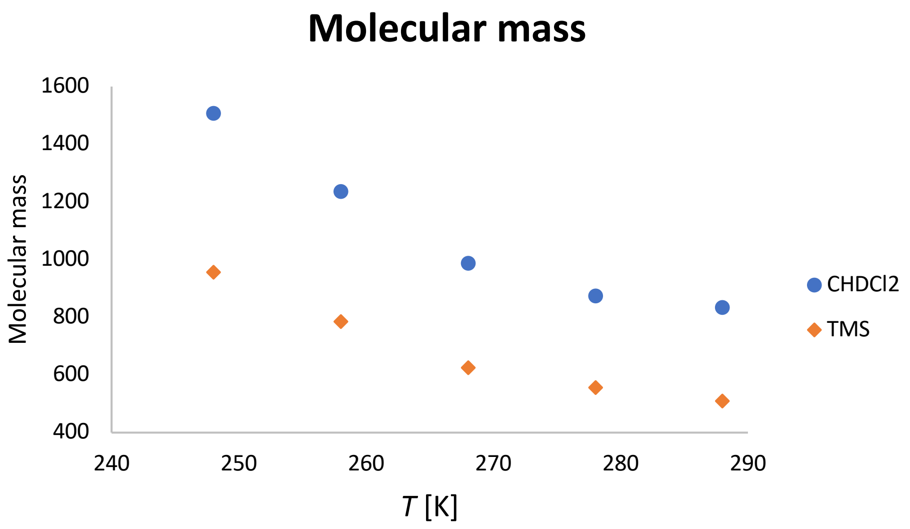

Figure SI 36. Dependence of extracted molecular mass of **2a** on temperature in CD_2Cl_2 at 6.4 mM concentration.

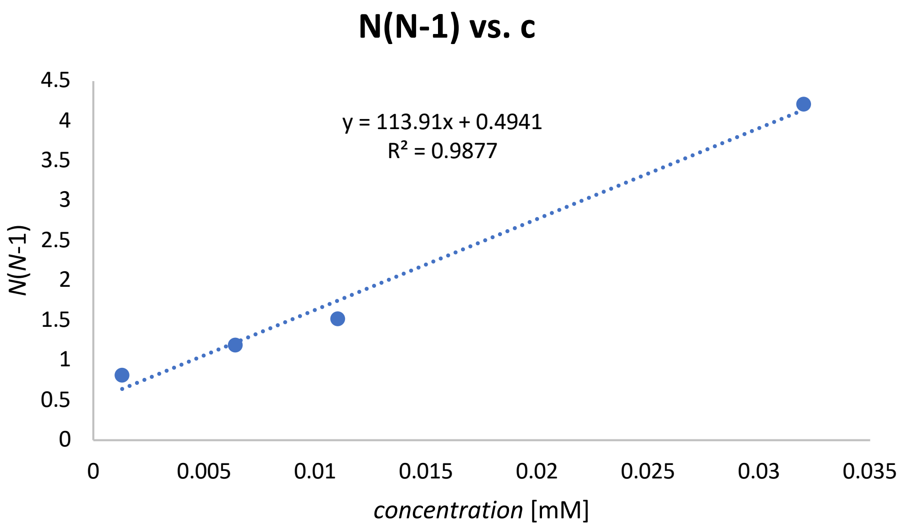

Figure SI 37. Extraction of association constant by Equal K model in CD_2Cl_2 based on molecular mass, showing a K = 114 M^{-1} (data are presented in Table **SI 6**). N is the aggregation number.[6]

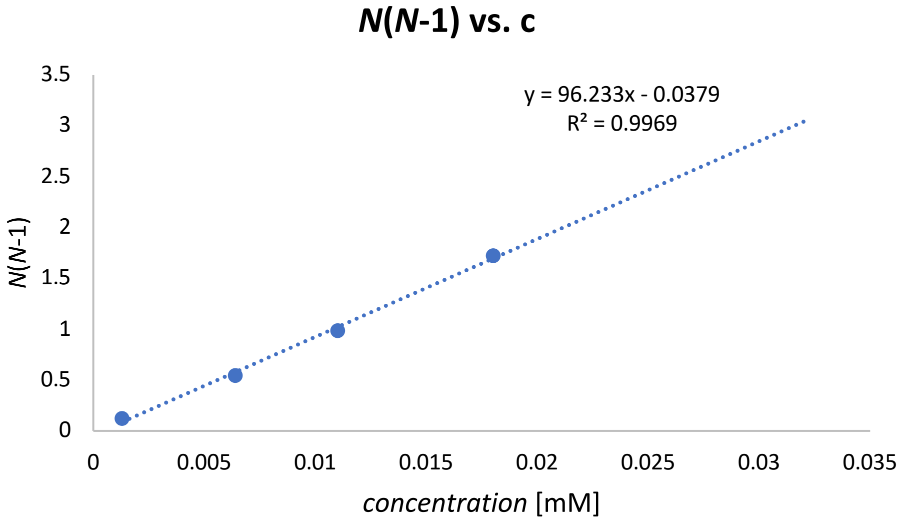

Figure SI 38. Extraction of association constant by Equal K model in CD_2Cl_2 based on calculated volume, showing a K = 96 M^{-1} (data are presented in **Table SI** 7). N is the aggregation number.[6]

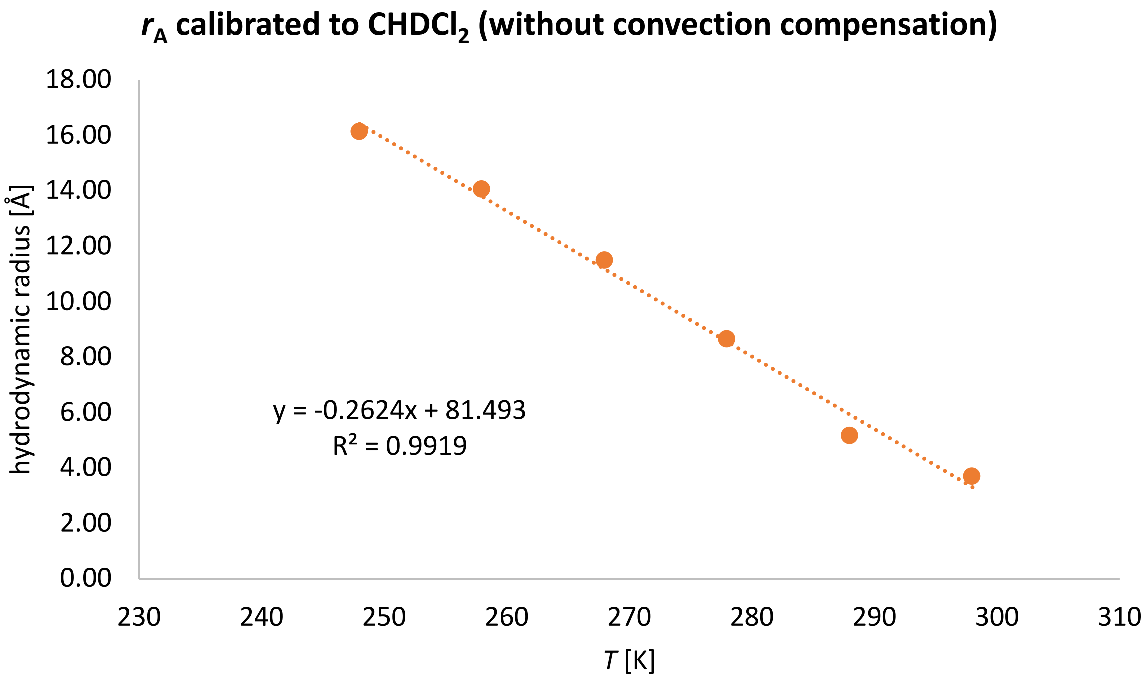

Figure SI2. Initial results using ledbpgp2s pulse sequence <u>without convection compensation</u>. Dependence of extracted hydrodynamic radii of **2a** on temperature in CD_2Cl_2 at 32 mM concentration.

[urea] [mM]	*T* [K]	D (urea) [m^2s^{-1}]	D (TMS) [m^2s^{-1}]	viscosity	MW [g·mol-1]	r_A [Å]
25	**288**	1.20E-09	3.19E-09	3.26E-14	474.0	7.87
25	**278**	1.00E-09	2.74E-09	3.66E-14	499.3	8.11
25	**268**	8.51E-10	2.36E-09	4.1E-14	509.8	8.21
25	**258**	7.20E-10	2.01E-09	4.63E-14	515.6	8.26

Table SI8. Diffusion data for **2a** acetone-d_6 for different temperatures.

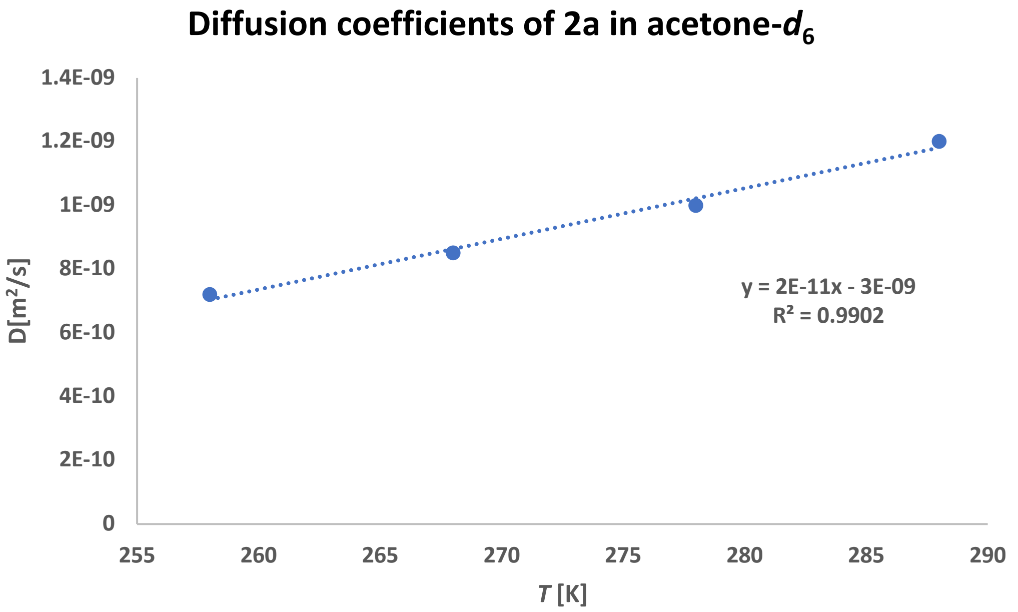

Figure SI 40. Dependence of diffusion coefficients of **2a** in acetone-d_6 on temperature.

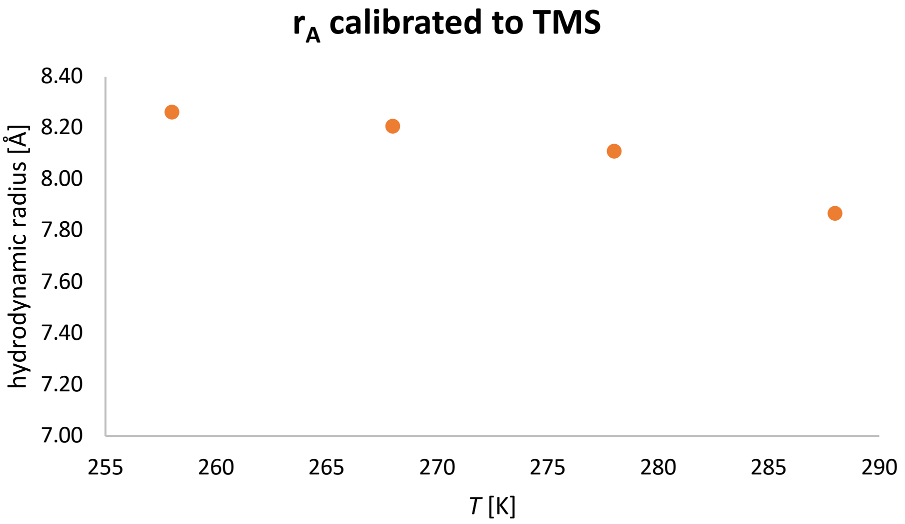

Figure SI 41. Temperature dependence of extracted hydrodynamic radius of **2a** in acetone-d_6.

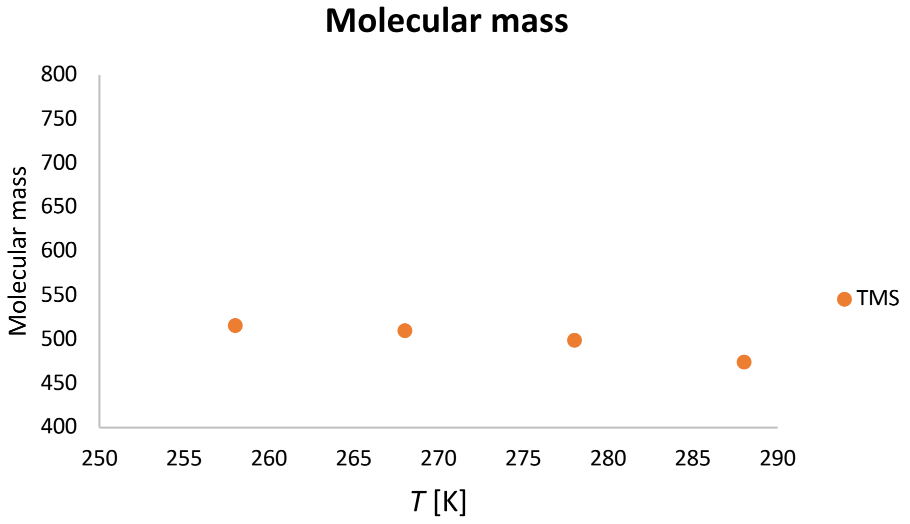

Figure SI 42. Temperature dependence of extracted molecular mass of **2a** in acetone-d_6 at different temperatures. Only a constant mass corresponding to monomeric urea was observed.

[urea] [mM]	*T* [K]	D (urea) [m²s⁻¹]	D (TMS) [m²s⁻¹]	viscosity	MW [g·mol-1]	r_A [Å]	D ($CHDCl_2$) [m²s⁻¹]	viscosity	MW [g·mol-1]	r_A [Å]
52	**288**	8.05E-10	2.57E-09	4.05E-14	649.5	9.45	3.03E-09	3.03E-09	840.0	7.97
52	**278**	6.16E-10	2.14E-09	4.69E-14	751.1	10.28	2.52E-09	2.52E-09	969.3	8.67
52	**268**	4.73E-10	1.84E-09	5.26E-14	912.4	11.51	2.18E-09	2.18E-09	1190.0	9.76
52	**258**	3.85E-10	1.67E-09	5.58E-14	1100.4	12.84	2.03E-09	2.03E-09	1499.8	11.17
52	**248**	2.89E-10	1.43E-09	6.26E-14	1380.1	14.65	1.79E-09	1.79E-09	1978.4	13.12

Table SI 9. Diffusion data for **2b** in CD_2Cl_2 for different temperatures.

[urea] [mM]	*T* [K]	r_A [Å]	V [Å³]	*N*
52	**288**	7.97	2123.8	1.00
52	**278**	8.67	2726.6	1.28
52	**268**	9.76	3899.0	1.84
52	**258**	11.17	5838.2	2.75
52	**248**	13.12	9463.1	4.46

Table SI 10. Aggregation of **2b** in CD_2Cl_2 at different temperatures. Estimation of the aggregation number on the assumption that monomer is predominant at 288 K.

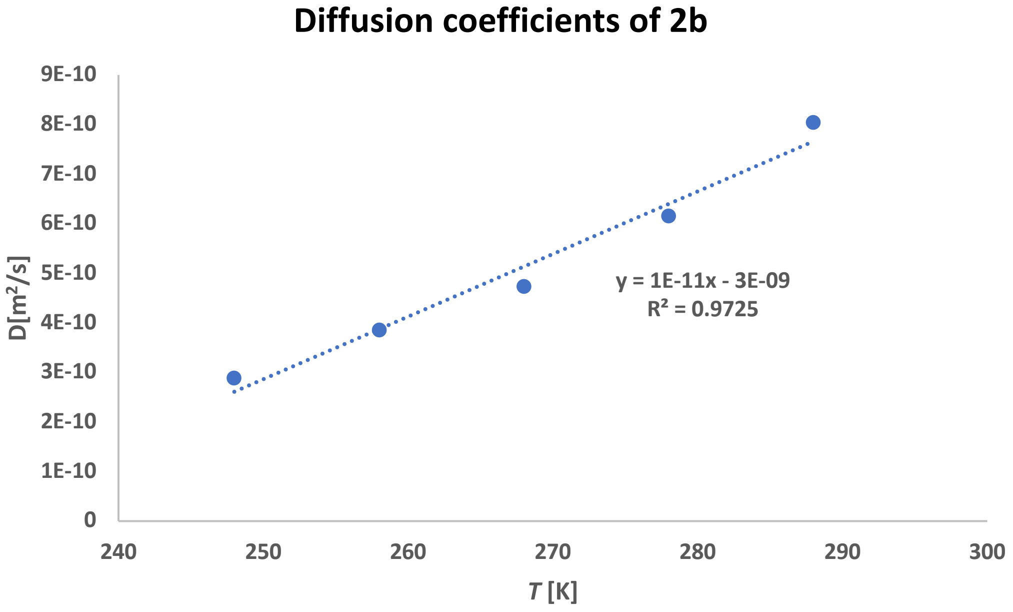

Figure SI 43. Dependence of diffusion coefficients of **2b** on temperature in CD_2Cl_2.

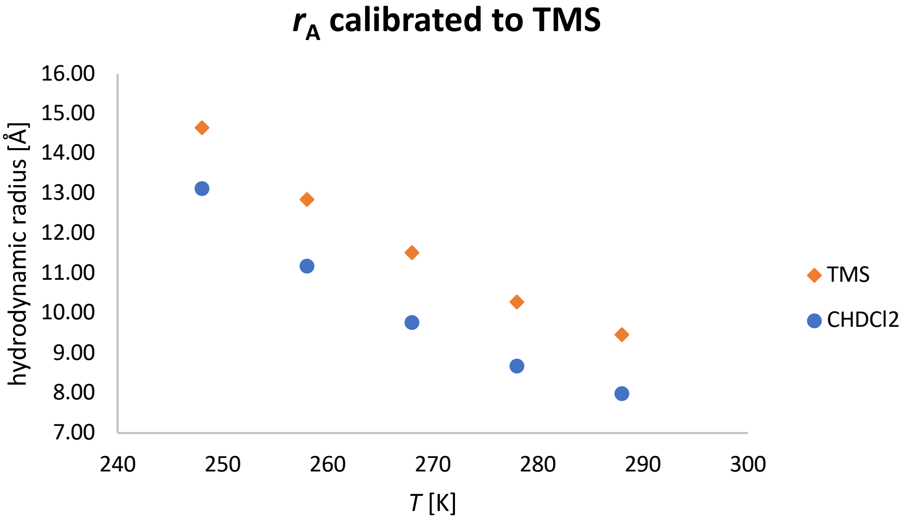

Figure SI 44. Dependence of extracted hydrodynamic radius of **2b** on temperature in CD_2Cl_2.

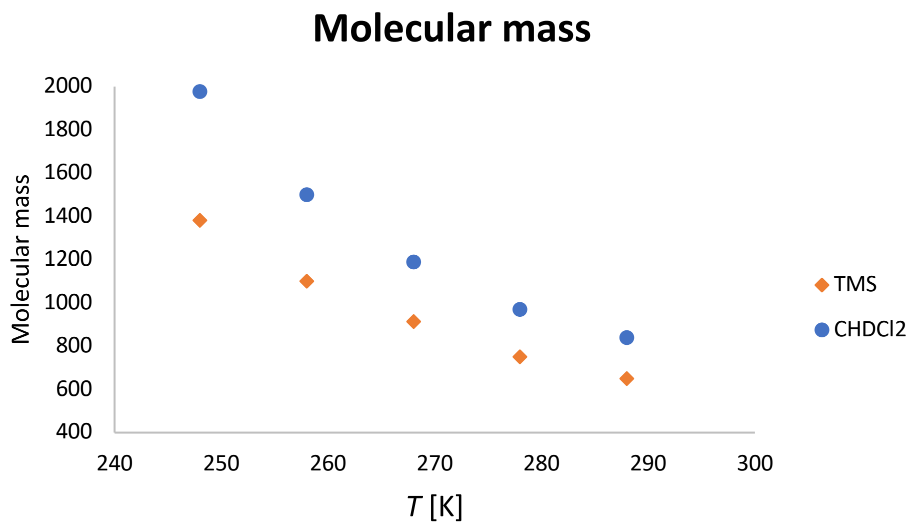

Figure SI 45. Dependence of extracted molecular mass of **2b** on temperature in CD_2Cl_2.

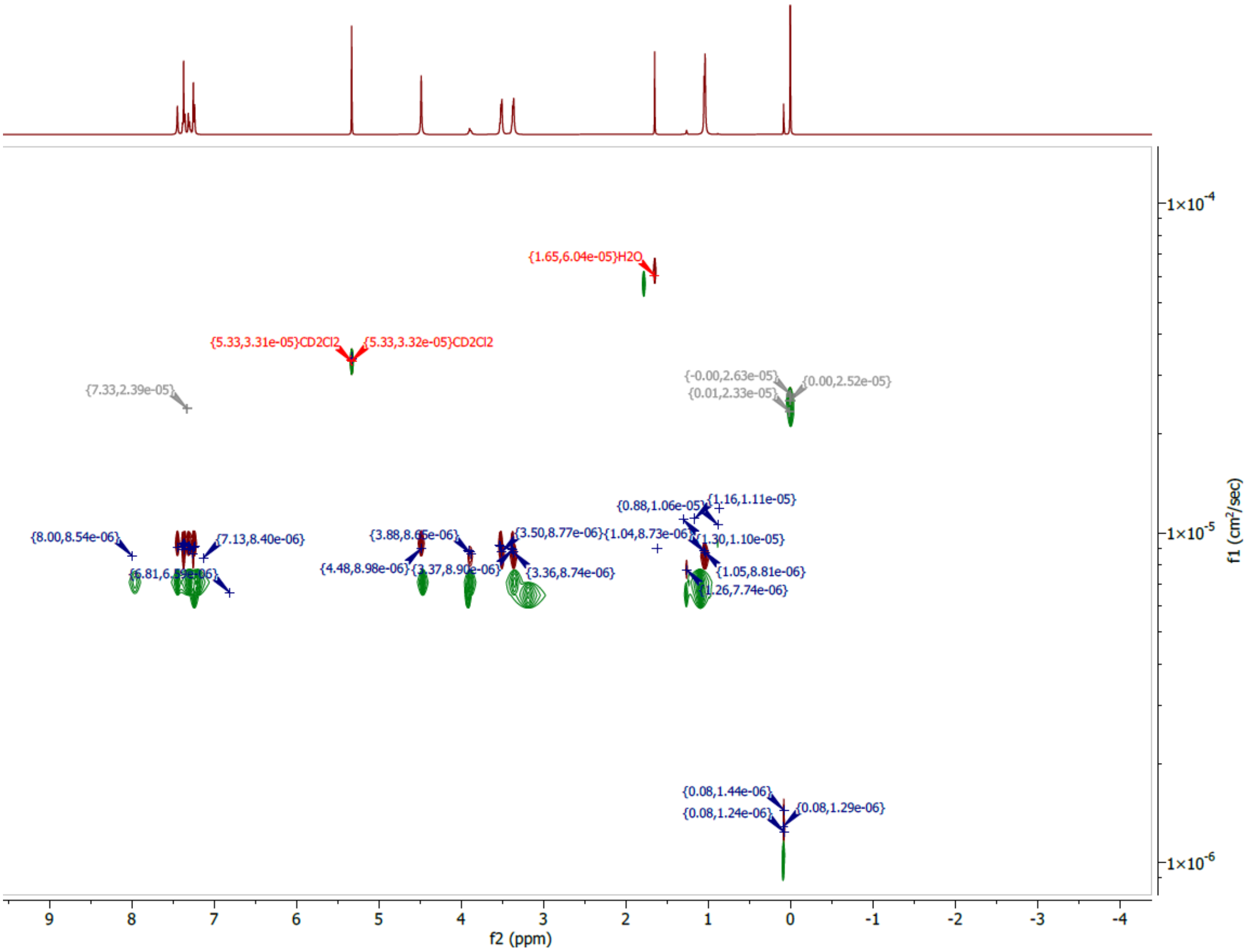

Figure SI 46. Overlaid 2D DOSY spectra of **2a** in CD_2Cl_2 at 32mM concentration (maroon) and 6.4 mM (green).

7. Computational studies

Geometry Optimization

Density functional theory (DFT) calculations were performed with Gaussian 16.[7] The geometries were optimized using the hybrid B3LYP functional[8,9] with D3(BJ) dispersion correction[10] and the split-valence plus polarization def2-SVP basis set.[11] All DFT calculations were conducted with the *ultrafine* integration grid. Frequency calculations were performed at the same level of theory as for geometry optimizations to verify the stationary points as minima (no imaginary frequencies) as well as to obtain thermal Gibbs free energy corrections at 298 K. Thermochemical free energy corrections were then recomputed at 298 K and concentration 1.0 mol.L^{-1} using the GoodVibes software package with Grimme's entropy corrections using quasi-rigid rotor harmonic approximation (qRRHO)[12] applied to all frequencies below 100 cm^{-1}.[13]

The hydrogen atom positions in the nanoring crystal structure were refined by B97-3c/def2-mTZVP[14] in ORCA 4.2.0 computational software.[15]

Single-Point Calculations

To refine the computed energy, single point calculations were performed in Gaussian 16 at B3LYP-D3(BJ)/def2-TZVPP/SMD(CH_2Cl_2) level of theory. SMD implicit solvation model for dichloromethane was used.[16]

HFLD

Hartree-Fock London Dispersion calculations[17] were conducted in Orca 4.2.0 with def2-TZVP basis, Rijcosx approximation, and NormalPNO settings (T_{CutPNO} 3.3x10^{-7}). T_{CutPairs} was set to 10^{-5}.

DLPNO-CCSD(T) Calculations

The DLPNO-CCSD(T) single-point energy calculations[18,19] were conducted in Orca 4.2.0 with def2-TZVP basis set, Rijcosx approximation, and LoosePNO (T_{CutPNO} 1x10^{-6}) settings.

The T1 diagnostics value was <0.02 in all cases. Local energy decomposition of total and interaction energy was performed according to Bistoni et al.[20,21] Triples corrections were added to the dispersive and non-dispersive components of the correlation energy.

Gibbs Free Energies

The ΔG value is obtained by adding the corresponding free energy corrections at temperature T calculated at the B3LYP-D3(BJ)/def2-SVP level, to ΔE, calculated at the single-point calculation at B3LYP-D3(BJ)/def2-TZVPP/SMD(CH_2Cl_2) level with SMD solvation correction. G^0_{sol} = +1.89 kcal·mol^{-1} is added for each compound in the dimerization equilibrium to capture the stoichiometric effect (two molecules of reactant and one molecule of product).

Computed Energies

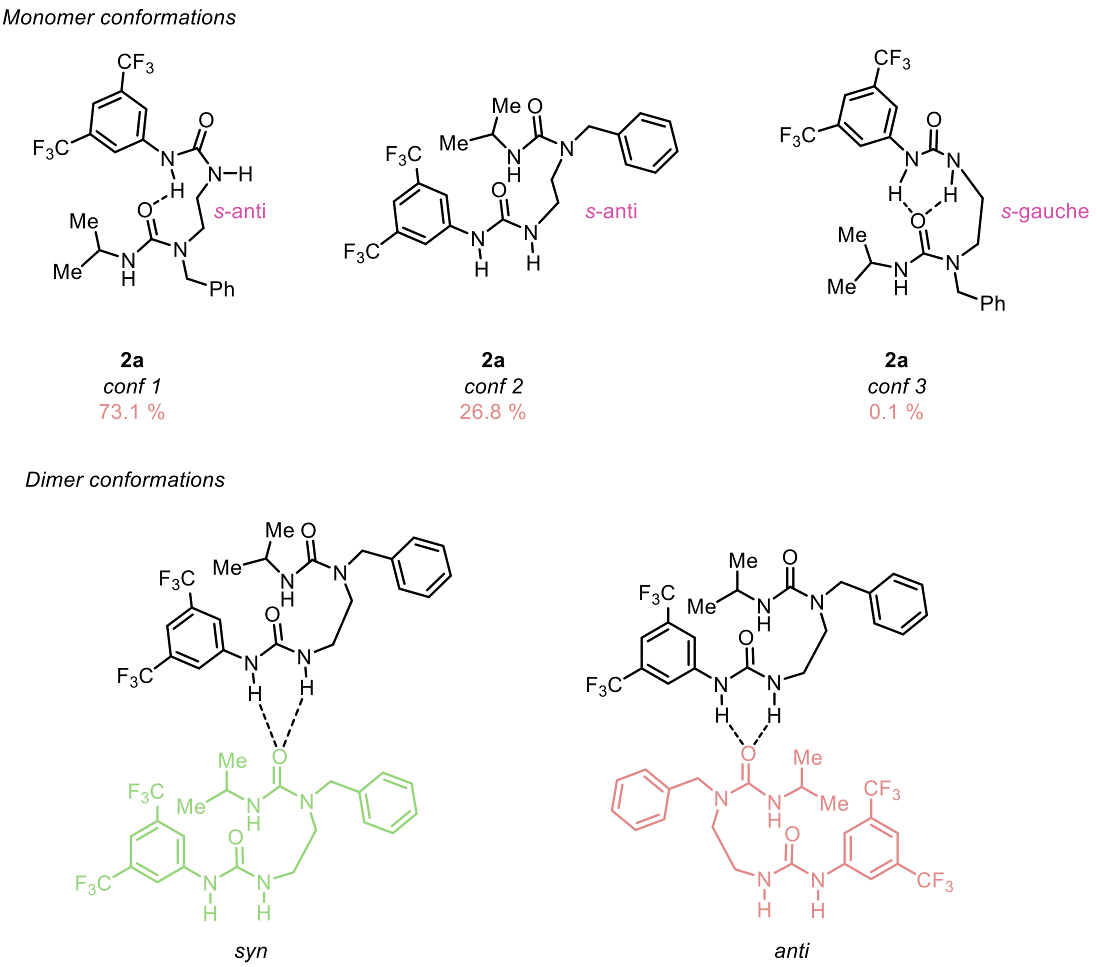

Figure SI 47. Conformations of monomer and dimer of **2a**.

Compound	B3LYP/SVP	G_{corr}	B3LYP/TZVPP/SMD	ΔG_{corr}	ΔGsolv [kJ·mol^{-1}]
Monomer_conf1	-1820.190302	0.394113	-1822.30344	-1821.909324	0.00
Monomer_conf2	-1820.184624	0.392742	-1822.30112	-1821.908376	2.49
Monomer_conf3	-1820.197741	0.398004	-1822.30081	-1821.902808	17.11
***syn*-dimer**	-3640.418776	0.812674	-3644.62946	-3643.816786	0.00
***anti*-dimer**	-3640.423258	0.815768	-3644.62560	-3643.811142	14.82
***syn*-dimer (2b)**	-3718.999395	0.867004			

Table SI 1. Computed Gibbs free energies (DCM, 298 K, 1 M) of **2a** based on B3LYP-D3(BJ)/def2-TZVPP/SMD(dichloromethane) single point calculations.

Gibbs free energies of the dimerization process were computed as follows:

$$\Delta G_{solv} = \Delta G_{solv}(dimer) - 2 * \Delta G_{solv}(monomer) - \Delta G^{0}_{solv}$$

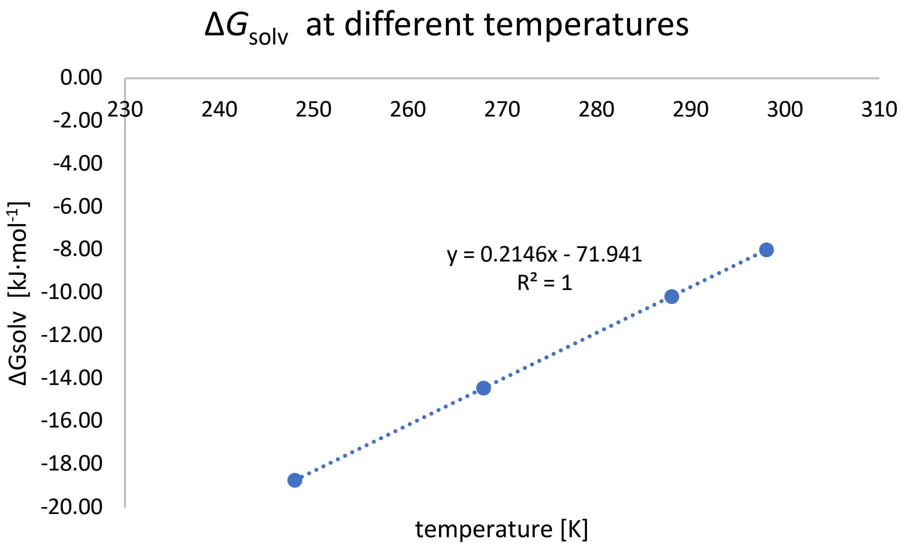

Figure SI 48. Computed Gibbs free energies (DCM, 1 M) of the dimerization process in the temperature range 298 – 248 K based on *monomer_conf2*, showing increasing association with decreasing temperature.

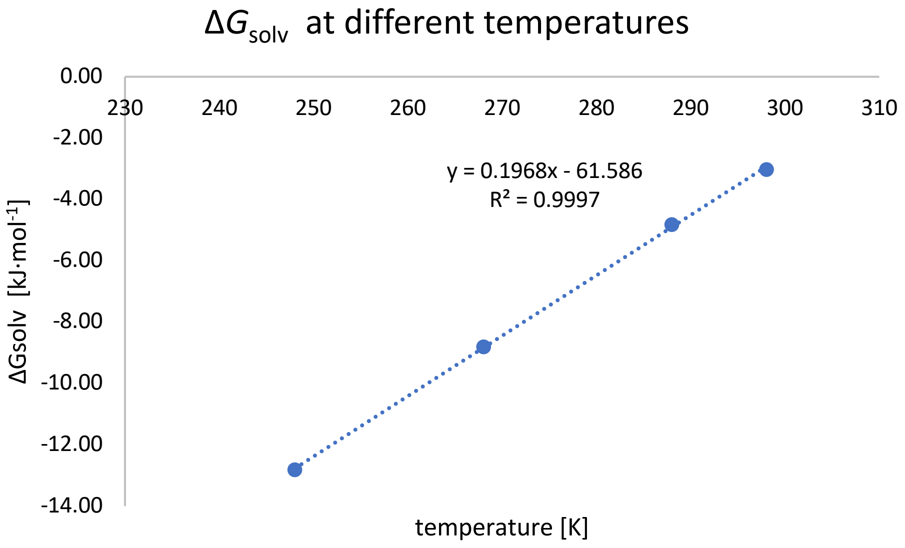

Figure SI 49. Computed Gibbs free energies (DCM, 1 M) of the dimerization process in the temperature range 298 – 248 K based on *monomer_conf1*, showing increasing association with decreasing temperature.

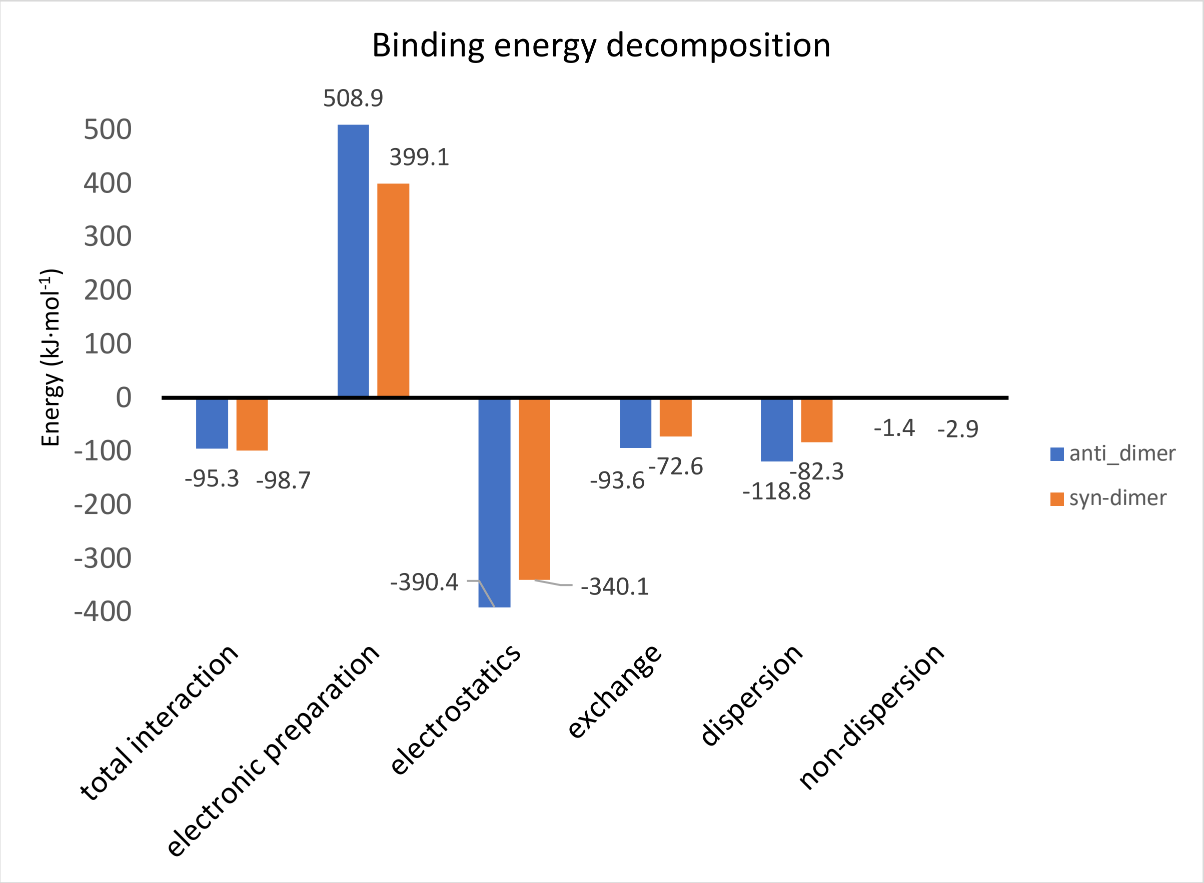

Figure SI 50. Decomposition of the binding energy of **2a** dimers at DLPNO-CCSD(T)/def2-TZVP/LoosePNO calculations (energies in kJ·mol^{-1}). The *syn*-dimer has slightly higher interaction energy despite weaker interactions than the *anti*-dimer.

**structure**	**dispersion interaction** [kJ·mol^{-1}]
syn-dimer **2a**	-75.2
syn-dimer **2b**	-86.1
anti-dimer **2a**	-109.5

Table SI 12. Computed dispersion interactions between monomers in the dimer structures based on HFLD/def2-TZVP/NormalPNO/TCutPairs(1x10^{-5}) calculations. In agreement with the CCSD(T) calculations, the *anti*-dimer has stronger dispersion interaction between the monomers. The change of an *i*-Pr group to a *t*-Bu group slightly increased the interaction in the *syn*-dimer.

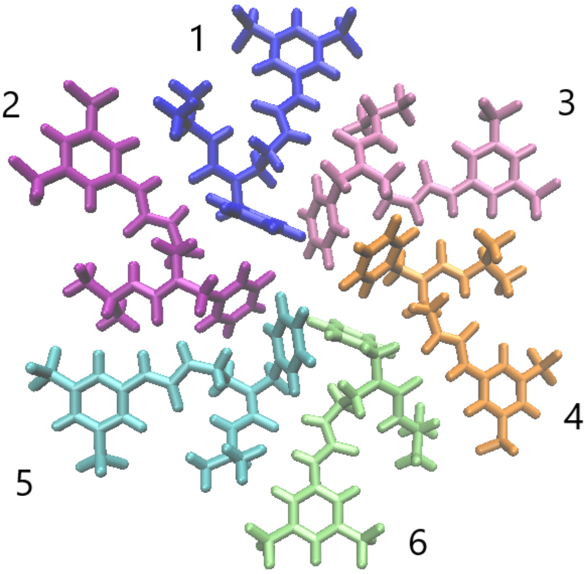

Pair	Dispersion interaction	[kJ·mol⁻¹]
2,1	-0.007893	-20.72
3,1	-0.008421	-22.11
3,2	0.000000	0.00
4,1	-0.000071	-0.19
4,2	0.000000	0.00
4,3	-0.008076	-21.20
5,1	-0.000071	-0.19
5,2	-0.007873	-20.67
5,3	0.000000	0.00
5,4	-0.000071	-0.19
6,1	0.000000	0.00
6,2	-0.000071	-0.19
6,3	-0.000071	-0.19
6,4	-0.007871	-20.66
6,5	-0.007868	-20.66

Table SI 13. Computed dispersion interactions between monomers **2a** in the cyclic hexamer structure based on HFLD/def2-TZVP(-f)/NormalPNO calculation. The energies are apparently lower due to a smaller basis set and looser settings used.

Computed NMR Chemical Shifts

NMR chemical shieldings and chemical shifts were calculated in ORCA 4.2.0 using TPSS-D3BJ/pcSseg-3/SMD(CH_2Cl_2) level of theory. Chemical shift scaling was done for NH and aromatic protons according to a published procedure.[22]

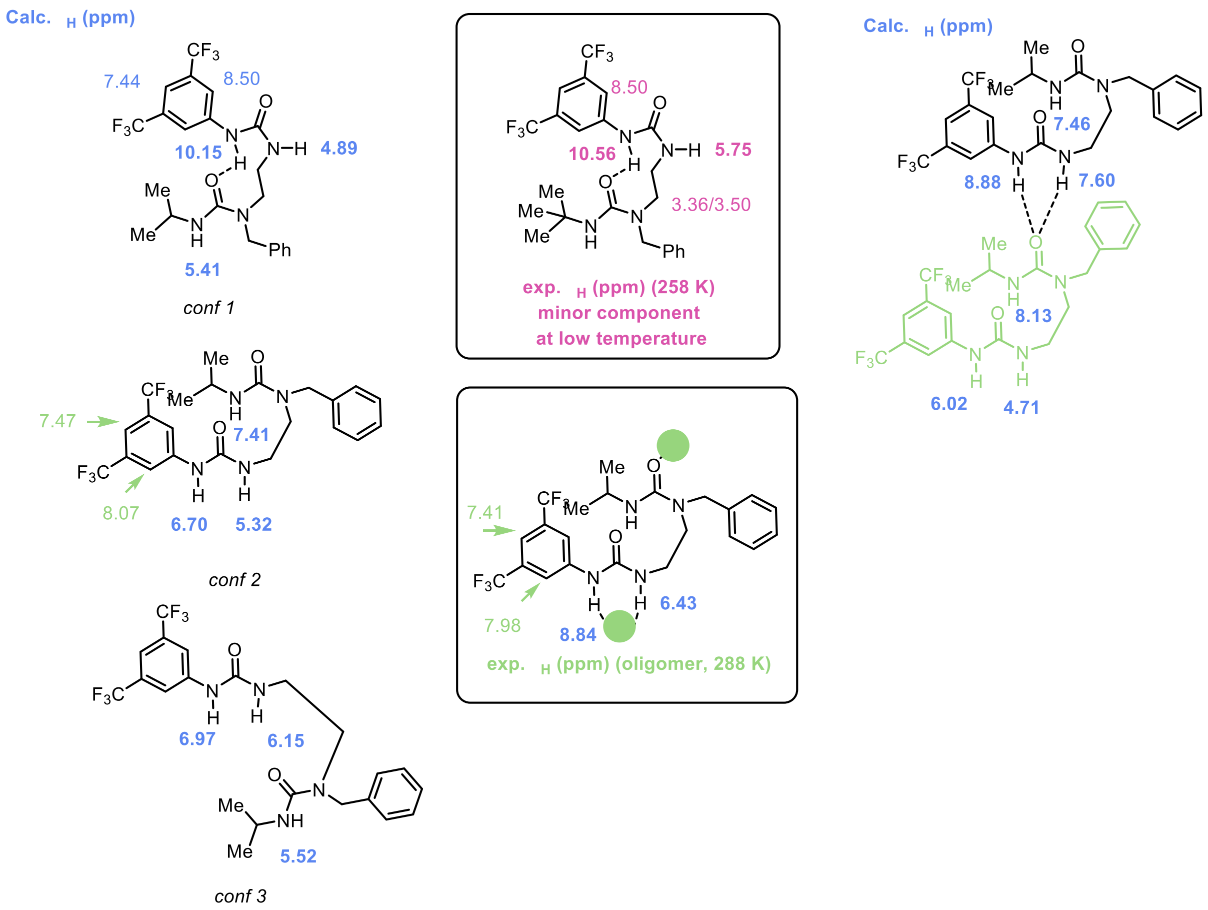

Figure SI 51. Computed ^{1}H chemical shifts of monomer (left) and dimer (right) conformers of **2a**. Experimental chemical shifts are shown for the minor component **2b** at 258 K (assigned as *conf1*, top inset), as well as major component of **2a** at 288 K (bottom inset).

Noncovalent Interaction (NCI) Plots

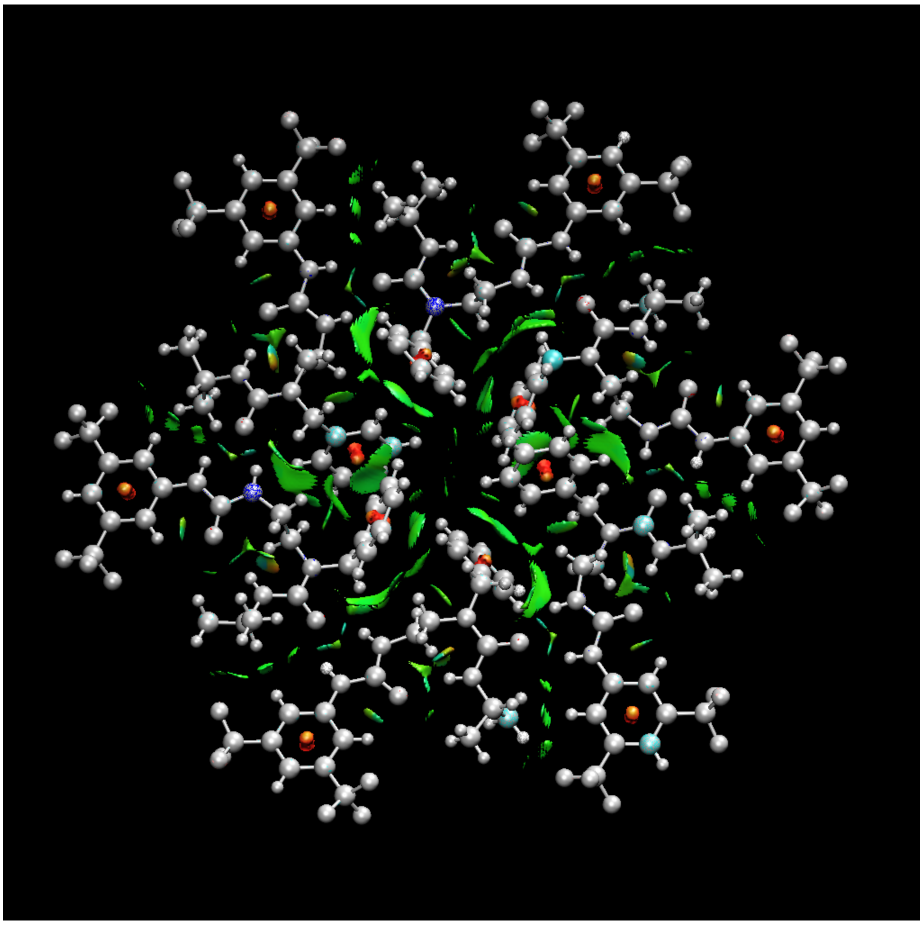

Figure SI 52. Noncovalent interaction plot (NCIPlot)[23],[24] of **2a** hexamer.

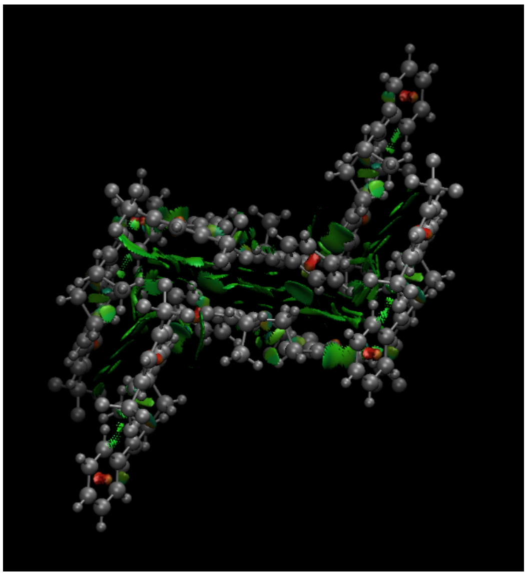

Figure SI 53. Noncovalent interaction plot (NCI) of **4b** polymeric structure **(**two antiparallel chains).

Coordinates of Optimized Structures

Method: B3LYP-D3(BJ)/def2-SVP

Monomer_conf2 **2a**

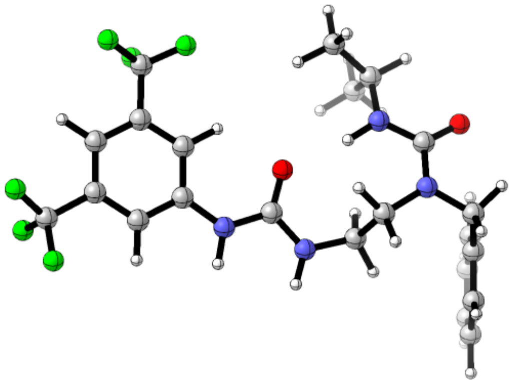

F 4.2104590000 2.7463260000 1.5380030000
F 3.0863320000 3.0788820000 -0.2796330000
F 5.2366380000 2.8059750000 -0.3651580000
F 7.0712980000 -1.6976490000 -0.7003440000
F 5.7304760000 -3.4035800000 -0.7024130000
F 6.4170860000 -2.5651830000 1.1691330000
O -4.7726950000 2.3623800000 -0.6107700000
O -0.2004800000 0.2616080000 0.0401970000
N -2.5165580000 2.0284140000 -0.5425540000
H -1.7580460000 1.3503800000 -0.5019760000
N -3.9504320000 0.3664450000 -1.3683620000
N -1.1538160000 -1.7928330000 -0.0789820000
H -1.0327620000 -2.7884080000 -0.2124230000
N 1.1565640000 -1.5940420000 -0.0089790000
H 1.1577320000 -2.6066470000 -0.0223330000
C 3.5438130000 -1.8451860000 -0.0599270000
H 3.4105770000 -2.9263430000 -0.1370630000
C -0.9571390000 3.9024360000 -0.3850920000
H -0.0900770000 3.2482390000 -0.1941050000
H -0.7580220000 4.8704230000 0.1004080000
H -1.0279010000 4.0663780000 -1.4710810000
C -5.9616520000 -0.4580030000 0.8545220000
H -5.8261630000 0.6187520000 0.9826350000
C -2.2469760000 3.2829130000 0.1538890000
H -3.0954670000 3.9430560000 -0.0752460000
C -2.1939830000 3.0635790000 1.6698870000
H -3.1480650000 2.6479340000 2.0285330000
H -2.0100240000 4.0100750000 2.2032040000
H -1.3852620000 2.3599800000 1.9284300000
C -3.7996630000 1.6406650000 -0.8150010000
C -5.3070100000 -0.1315610000 -1.5612440000
H -5.3483930000 -0.6901320000 -2.5107940000
H -5.9527750000 0.7510740000 -1.6474300000
C -5.7743590000 -1.0144170000 -0.4220180000

C -5.9580000000 -2.3914390000 -0.5941510000
H -5.8196690000 -2.8319430000 -1.5862190000
C -6.3119280000 -3.2070140000 0.4868150000
H -6.4531100000 -4.2805450000 0.3371960000
C -6.4856090000 -2.6474060000 1.7544850000
H -6.7614290000 -3.2808000000 2.6011440000
C -6.3132150000 -1.2693540000 1.9335690000
H -6.4582340000 -0.8256590000 2.9218050000
C -2.8586320000 -0.5769060000 -1.4476060000
H -1.9659800000 -0.0731840000 -1.8479470000
H -3.1251850000 -1.3572530000 -2.1780540000
C -2.4963510000 -1.2313960000 -0.0930730000
H -2.5641310000 -0.4690780000 0.6958730000
H -3.2100480000 -2.0249360000 0.1627650000
C -0.0752530000 -0.9582360000 -0.0155530000
C 2.4248480000 -0.9995940000 0.0140800000
C 2.6243690000 0.3885170000 0.0986570000
H 1.7653260000 1.0493300000 0.1531670000
C 3.9223450000 0.8988650000 0.1016490000
C 5.0383510000 0.0641170000 0.0233880000
H 6.0457690000 0.4784300000 0.0095490000
C 4.8330820000 -1.3149290000 -0.0515730000
C 4.1135240000 2.3904970000 0.2440580000
C 6.0187420000 -2.2477900000 -0.0763670000

Monomer_conf1 **2a** (*E,Z*-aryl urea)

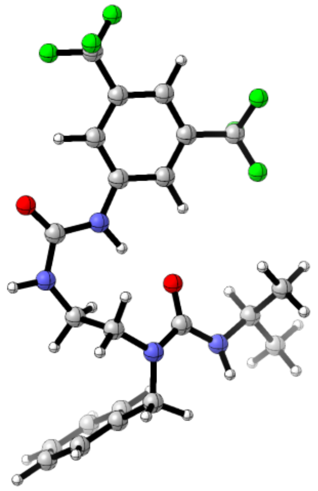

F -5.8447620000 -2.2068830000 1.1603200000
F -5.4097210000 -2.9600460000 -0.8181280000
F -6.6156380000 -1.1745220000 -0.5777960000
F -4.4076530000 3.1570610000 0.2873480000
F -3.2912370000 3.0651730000 -1.5615560000
F -2.2402880000 3.2574810000 0.3179870000
O 1.1187300000 0.8729460000 0.2734710000
O -1.0491590000 -3.6513340000 0.1890460000
N 2.8562090000 2.3157660000 0.4324590000
H 3.6742900000 2.5697450000 0.9718890000
N 3.0126820000 0.1164110000 1.2769360000
N 1.1115150000 -2.9627350000 0.2395480000

H 1.3343430000 -3.9463510000 0.3228590000
N -0.5920740000 -1.3955890000 0.0219490000
H 0.1410050000 -0.6809860000 0.0593560000
C -1.9816390000 0.5422110000 -0.1001260000
H -1.0718930000 1.1411250000 -0.0770240000
C 3.0305870000 4.4565240000 -0.7115500000
H 3.6732000000 4.8949170000 0.0720680000
H 2.4696850000 5.2817370000 -1.1743510000
H 3.6797660000 4.0057950000 -1.4778110000
C 5.0961380000 -0.1202290000 -1.0040340000
H 4.5341380000 0.7934850000 -1.2126740000
C 2.0720290000 3.4256040000 -0.1219570000
H 1.4674850000 2.9884030000 -0.9303110000
C 1.1190890000 4.0308740000 0.9116890000
H 0.4400270000 3.2630770000 1.3071090000
H 0.5014000000 4.8189510000 0.4541200000
H 1.6808270000 4.4774810000 1.7496030000
C 2.2758820000 1.0971230000 0.6486360000
C 4.4636080000 0.1413190000 1.4312760000
H 4.7146130000 -0.3188640000 2.3996170000
H 4.8137870000 1.1808920000 1.4940390000
C 5.1907600000 -0.5855340000 0.3168070000
C 5.9092770000 -1.7581710000 0.5745490000
H 5.9847430000 -2.1301980000 1.6003010000
C 6.5229210000 -2.4611250000 -0.4677100000
H 7.0789650000 -3.3768240000 -0.2532920000
C 6.4198690000 -1.9943560000 -1.7796140000
H 6.8949230000 -2.5431450000 -2.5960060000
C 5.7065730000 -0.8196780000 -2.0450520000
H 5.6257940000 -0.4491620000 -3.0696660000
C 2.3424740000 -1.1677540000 1.4578220000
H 1.3470930000 -0.9804840000 1.8822240000
H 2.9087730000 -1.7459730000 2.2027300000
C 2.1765640000 -1.9885780000 0.1555890000
H 1.9934810000 -1.2920810000 -0.6775850000
H 3.1100440000 -2.5141170000 -0.0867870000
C -0.2479950000 -2.7303780000 0.1510210000
C -1.8797960000 -0.8622750000 -0.0513670000
C -3.0562730000 -1.6302250000 -0.0766180000
H -2.9837240000 -2.7142880000 -0.0432880000
C -4.2962430000 -0.9871630000 -0.1398110000
C -4.4034800000 0.4033120000 -0.1845580000
H -5.3783630000 0.8862670000 -0.2317580000
C -3.2267970000 1.1586440000 -0.1656510000
C -5.5440800000 -1.8332360000 -0.0990330000
C -3.2937360000 2.6591470000 -0.2761960000

Monomer_conf3 **2a**

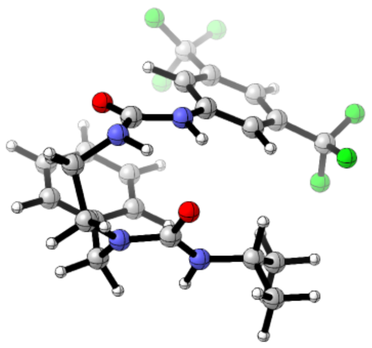

F -3.4489860000 -3.1804480000 0.9261470000
F -3.3702510000 -2.5535930000 -1.1431250000
F -4.9741340000 -1.8599780000 0.1319390000
F -2.7697500000 2.9903250000 -1.2945630000
F -2.1652840000 3.7764550000 0.6289030000
F -4.1849960000 3.0378600000 0.3382910000
O 3.0469140000 1.5419290000 1.3330450000
O 0.9587410000 -2.3964140000 1.4330370000
N 2.4035730000 1.8869000000 -0.8103370000
H 2.5534050000 1.6682740000 -1.7858380000
N 3.6535370000 -0.0147840000 -0.2387540000
N 2.6153010000 -1.0648310000 2.2945800000
H 2.8120960000 -0.0819430000 2.4790710000
N 0.5361260000 -0.1678020000 1.8727830000
H 1.0430030000 0.7066230000 1.9893460000
C -1.2020430000 1.2446200000 1.0234680000
H -0.5688380000 2.1085810000 1.2264540000
C 0.6025730000 3.3786730000 -1.4941520000
H 0.9813060000 3.4878150000 -2.5252110000
H 0.0471520000 4.2939710000 -1.2449870000
H -0.1075190000 2.5396220000 -1.4750270000
C 0.9070180000 -0.9100030000 -1.8132450000
H 0.8089250000 0.1730520000 -1.8889510000
C 1.7490960000 3.1596350000 -0.5107700000
H 1.3423910000 3.0542380000 0.5057120000
C 2.7475350000 4.3193860000 -0.4941280000
H 3.5394210000 4.1262930000 0.2439300000
H 2.2457150000 5.2623380000 -0.2258550000
H 3.2123060000 4.4506260000 -1.4856820000
C 3.0420550000 1.1593200000 0.1527950000
C 3.4260110000 -0.6425500000 -1.5440000000
H 4.3043810000 -1.2699840000 -1.7519440000
H 3.4301400000 0.1277760000 -2.3324150000
C 2.1728250000 -1.4921160000 -1.6431360000
C 2.2615280000 -2.8860940000 -1.5426280000
H 3.2412090000 -3.3558370000 -1.4160230000
C 1.1154730000 -3.6818930000 -1.5917610000
H 1.2031090000 -4.7662660000 -1.4943150000

C -0.1386250000 -3.0920350000 -1.7527930000
H -1.0418970000 -3.7044940000 -1.7777410000
C -0.2384710000 -1.7037590000 -1.8705100000
H -1.2172110000 -1.2393950000 -1.9980870000
C 4.4792850000 -0.7234910000 0.7381620000
H 4.9589560000 0.0349660000 1.3740250000
H 5.2794020000 -1.2490150000 0.1927650000
C 3.7287590000 -1.7232610000 1.6371740000
H 3.3257000000 -2.5698670000 1.0648600000
H 4.4449300000 -2.1309030000 2.3720370000
C 1.3405960000 -1.3124320000 1.8335980000
C -0.7391040000 -0.0467010000 1.3322760000
C -1.5722980000 -1.1471280000 1.0662130000
H -1.2141060000 -2.1493420000 1.2822220000
C -2.8248550000 -0.9382750000 0.4884000000
C -3.2857020000 0.3410460000 0.1709230000
H -4.2690440000 0.4856840000 -0.2756610000
C -2.4550970000 1.4309190000 0.4455880000
C -3.6597290000 -2.1362140000 0.1134550000
C -2.8967150000 2.8125390000 0.0388080000

Syn-dimer **2a**

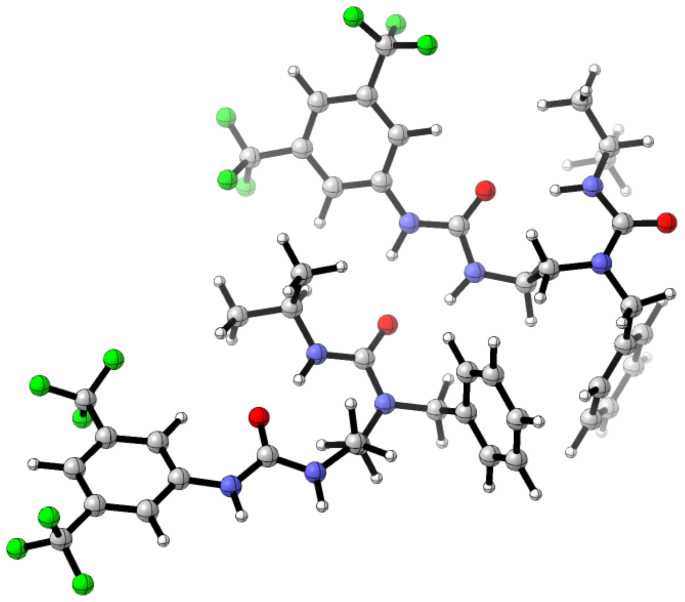

F -5.1771570000 6.5138780000 -0.5458890000
F -5.1052080000 5.0956670000 -2.1778850000
F -3.8009880000 6.8283790000 -2.1832880000
F 0.0225000000 7.0250120000 0.9536960000
F 1.1107150000 5.2629760000 0.3156080000
F 0.3171870000 5.3675260000 2.3219990000
O -8.2997990000 -2.3933200000 -1.0688200000
O -4.7439970000 1.1691830000 -0.2137650000
N -7.1347890000 -0.4318580000 -1.0128310000
H -6.2401330000 0.0298990000 -0.8480090000
N -6.0219280000 -2.4733480000 -1.2873950000
N -3.3926210000 -0.5093680000 0.5340920000
H -2.4959020000 -0.7239830000 0.9702520000
N -2.6214580000 1.6487890000 0.5649730000
H -1.8050780000 1.2049020000 0.9929960000
C -1.4230480000 3.6975970000 0.8320810000
H -0.6958360000 3.1612180000 1.4403870000
C -8.1755450000 1.7493420000 -1.3257400000
H -7.2907190000 2.2799810000 -0.9371590000
H -9.0620320000 2.3663520000 -1.1118370000
H -8.0678120000 1.6605940000 -2.4175320000
C -6.6102060000 -4.3317340000 1.1569340000
H -7.5699520000 -3.8463440000 0.9630630000
C -8.3070830000 0.3703250000 -0.6797110000
H -9.1718680000 -0.1583250000 -1.1062620000
C -8.4953300000 0.4602500000 0.8390720000
H -8.6078100000 -0.5471900000 1.2675750000
H -9.3933780000 1.0451980000 1.0959660000
H -7.6223610000 0.9467010000 1.3052940000
C -7.2272750000 -1.7925390000 -1.1116010000
C -6.0546390000 -3.9300590000 -1.2723230000
H -5.3434070000 -4.3088710000 -2.0248920000
H -7.0693770000 -4.2233750000 -1.5681110000
C -5.7142120000 -4.5033290000 0.0885100000
C -4.4812100000 -5.1207400000 0.3293270000
H -3.7717990000 -5.2488500000 -0.4914910000
C -4.1397340000 -5.5538960000 1.6150580000
H -3.1719010000 -6.0311620000 1.7855950000

C -5.0317650000 -5.3724670000 2.6741990000
H -4.7672540000 -5.7079460000 3.6801800000
C -6.2703870000 -4.7626680000 2.4396560000
H -6.9745060000 -4.6233230000 3.2639020000
C -4.7427330000 -1.8251630000 -1.0953420000
H -4.7139090000 -0.8842770000 -1.6635750000
H -3.9631880000 -2.4710870000 -1.5225380000
C -4.4125970000 -1.5261160000 0.3850840000
H -5.3369330000 -1.2087760000 0.8929630000
H -4.0651280000 -2.4368170000 0.8889060000
C -3.6702230000 0.7948970000 0.2603030000
C -2.5393940000 3.0152030000 0.3236880000
C -3.4903330000 3.7409060000 -0.4205530000
H -4.3655080000 3.2281940000 -0.8086330000
C -3.2914470000 5.1006880000 -0.6545850000
C -2.1577240000 5.7727610000 -0.1849410000
H -2.0051820000 6.8300830000 -0.3963350000
C -1.2273120000 5.0508320000 0.5617720000
C -4.3474730000 5.8817660000 -1.3976490000
C 0.0485130000 5.6891370000 1.0432390000
F 6.4630920000 2.5468300000 0.6460550000
F 7.8061540000 1.9352540000 2.2264910000
F 8.5940870000 2.9112960000 0.4631400000
F 11.5928070000 -0.8969130000 -0.6837770000
F 10.7008710000 -2.7564570000 -1.3584680000
F 10.6934970000 -1.0183550000 -2.6462990000
O -0.7242250000 -0.3303080000 1.4941620000
O 4.0511390000 -1.0234280000 0.0739620000
N 1.5081550000 -0.1040610000 1.1147610000
H 2.3862930000 -0.5380930000 0.8250760000
N 0.5741870000 -2.2121320000 1.4988000000
N 3.6645720000 -3.2058620000 -0.3948430000
H 4.0388280000 -4.1257320000 -0.5881450000
N 5.8266510000 -2.3642940000 -0.4948290000
H 6.0788030000 -3.2744150000 -0.8609290000
C 8.1441870000 -1.8510270000 -0.8695650000
H 8.2765300000 -2.8451250000 -1.3017090000
C 2.7689300000 1.9883750000 1.2696880000
H 3.5692740000 1.6559580000 0.5900740000
H 2.6880780000 3.0822260000 1.1895870000
H 3.0630720000 1.7348970000 2.2994920000
C -1.2925920000 -3.0643010000 -0.8037800000
H -1.4038610000 -1.9803010000 -0.7321040000
C 1.4326970000 1.3431680000 0.9061230000
H 0.6673720000 1.7098900000 1.6055750000
C 1.0042070000 1.6801080000 -0.5250880000
H 0.0179280000 1.2485770000 -0.7512360000
H 0.9441120000 2.7688480000 -0.6666130000
H 1.7367100000 1.2777100000 -1.2435720000
C 0.4028840000 -0.8454620000 1.3652840000
C -0.5924540000 -3.0792650000 1.6259260000
H -0.3998260000 -3.8161250000 2.4219960000
H -1.4319930000 -2.4571120000 1.9575100000
C -0.9226280000 -3.7981360000 0.3336550000
C -0.7990060000 -5.1885050000 0.2303980000
H -0.5115360000 -5.7690320000 1.1116070000
C -1.0400790000 -5.8402470000 -0.9837520000
H -0.9474270000 -6.9270120000 -1.0466950000
C -1.4036790000 -5.1010940000 -2.1119970000
H -1.5940420000 -5.6060170000 -3.0617000000
C -1.5306700000 -3.7104100000 -2.0174660000
H -1.8218800000 -3.1277820000 -2.8945160000
C 1.8608790000 -2.8486900000 1.2959360000

H 2.6421890000 -2.2499840000 1.7849630000
H 1.8471740000 -3.8222490000 1.8091750000
C 2.2335820000 -3.0442870000 -0.1929200000
H 1.8914100000 -2.1672040000 -0.7615860000
H 1.7206800000 -3.9186020000 -0.6132100000
C 4.4880430000 -2.1255720000 -0.2499790000
C 6.8858050000 -1.4482390000 -0.3945970000
C 6.7445790000 -0.1683420000 0.1634840000
H 5.7761290000 0.1551710000 0.5304160000
C 7.8506450000 0.6795190000 0.2320890000
C 9.1027810000 0.2876600000 -0.2418100000
H 9.9569490000 0.9612430000 -0.1835280000
C 9.2366880000 -0.9890440000 -0.7926010000
C 7.6783990000 2.0284830000 0.8905390000
C 10.5642410000 -1.4180940000 -1.3687920000

Anti-dimer **2a**

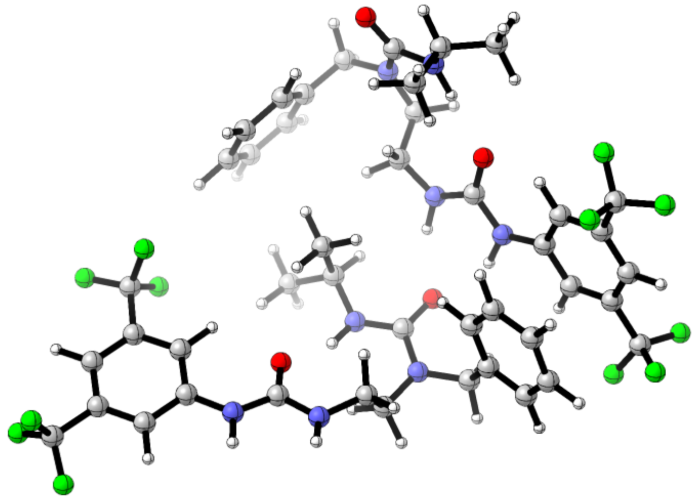

F 6.0373420000 -1.0727050000 3.4142230000
F 6.8473380000 0.7755160000 2.6323580000
F 8.0462680000 -1.0316050000 2.6089990000
F 7.5710660000 -3.5681930000 -2.1017530000
F 5.5017740000 -4.1690160000 -2.2981160000
F 6.6413040000 -4.7375100000 -0.5416940000
O 1.4871490000 6.5711520000 1.2480610000
O 3.5995950000 2.1675210000 0.0125170000
N 2.9629360000 4.8467820000 1.0086170000
H 3.1988950000 3.9908010000 0.5053410000
N 1.5951450000 5.5089850000 -0.7756880000
N 1.9646970000 1.8556130000 -1.5465200000
H 1.4966560000 1.1492660000 -2.1163890000
N 3.5374380000 0.2135630000 -1.2113550000
H 2.9466120000 -0.3041330000 -1.8649800000
C 5.0017530000 -1.6762290000 -1.2772990000
H 4.5078320000 -1.9924140000 -2.1959500000
C 4.8168250000 4.5026750000 2.5596010000
H 5.0349220000 3.4955570000 2.1675150000
H 5.1238900000 4.5262640000 3.6168860000
H 5.4313700000 5.2256790000 2.0020600000
C -1.3702860000 5.2885230000 0.1751880000
H -0.7849040000 5.5897910000 1.0469680000
C 3.3311180000 4.8354580000 2.4201610000
H 3.1434770000 5.8525250000 2.7930020000
C 2.4467660000 3.8581100000 3.2039580000
H 1.3875340000 4.1418750000 3.1076960000
H 2.7059790000 3.8549130000 4.2751490000
H 2.5727030000 2.8340440000 2.8147760000
C 1.9888680000 5.6899930000 0.5510720000
C 0.4639210000 6.2917840000 -1.2544230000
H 0.6339480000 6.5430420000 -2.3138450000
H 0.4605010000 7.2231450000 -0.6734010000
C -0.8777210000 5.5961310000 -1.1053010000
C -1.6373810000 5.2309660000 -2.2234330000
H -1.2669250000 5.4685260000 -3.2251330000
C -2.8642020000 4.5728580000 -2.0746740000
H -3.4482330000 4.3048140000 -2.9587610000

C -3.3418990000 4.2614190000 -0.7997150000
H -4.2896070000 3.7320630000 -0.6782320000
C -2.5886030000 4.6237010000 0.3239160000
H -2.9597050000 4.3886440000 1.3247230000
C 2.0205250000 4.3630160000 -1.5527780000
H 3.1009660000 4.2096830000 -1.4233190000
H 1.8673450000 4.5859430000 -2.6201960000
C 1.2684870000 3.0704230000 -1.1707690000
H 1.0950620000 3.0847130000 -0.0827900000
H 0.2825590000 3.0612220000 -1.6481160000
C 3.0679790000 1.4650580000 -0.8497680000
C 4.6030030000 -0.4829200000 -0.6491390000
C 5.2532180000 -0.0909170000 0.5319540000
H 4.9534210000 0.8299230000 1.0226720000
C 6.2459920000 -0.9061740000 1.0765960000
C 6.6347070000 -2.0975770000 0.4655000000
H 7.4019980000 -2.7295800000 0.9110710000
C 5.9992710000 -2.4702160000 -0.7221390000
C 6.8050950000 -0.5497150000 2.4287490000
C 6.4254770000 -3.7363610000 -1.4171660000
F -6.0247350000 2.0016910000 0.6957200000
F -6.1607160000 1.3666710000 -1.3629710000
F -7.9610920000 1.8905320000 -0.2729930000
F -10.5353080000 -2.1114090000 0.8222520000
F -9.5251590000 -3.9363350000 1.4203240000
F -9.7415550000 -2.3044590000 2.8240420000
O 1.1396530000 -0.7424620000 -2.5037000000
O -3.2101090000 -1.8314110000 -0.4238490000
N -1.0939990000 -0.8999580000 -2.0543400000
H -1.8228430000 -1.4346340000 -1.5823240000
N 0.2783200000 -2.7753290000 -1.9001120000
N -2.4518910000 -3.7568130000 0.4911590000
H -2.6100750000 -4.5142390000 1.1435260000
N -4.6663080000 -3.0899810000 0.8127070000
H -4.8040170000 -3.9794550000 1.2774030000
C -7.0397120000 -2.7998350000 1.1116880000
H -7.1135180000 -3.8433900000 1.4241640000
C -2.7114010000 0.7986020000 -2.7370360000
H -3.5361720000 0.3149650000 -2.1906050000
H -2.9167900000 1.8775020000 -2.7663600000
H -2.7044750000 0.4096910000 -3.7664330000
C 2.1677100000 -2.3548190000 0.3646980000
H 1.5471430000 -1.4939230000 0.1134640000
C -1.3718530000 0.5363170000 -2.0491460000
H -0.5782550000 1.0170790000 -2.6387560000
C -1.3489700000 1.0704380000 -0.6130250000
H -0.3648460000 0.9005440000 -0.1507800000
H -1.5661270000 2.1463420000 -0.5805950000
H -2.1083270000 0.5499760000 -0.0094530000
C 0.1515680000 -1.4209320000 -2.1661820000
C 1.6049060000 -3.3699500000 -1.8968570000
H 1.5360030000 -4.3865460000 -2.3151910000
H 2.2235570000 -2.7821820000 -2.5901110000
C 2.2786670000 -3.4215950000 -0.5378120000
C 3.0994460000 -4.5056060000 -0.2016240000
H 3.2015790000 -5.3411210000 -0.8991110000
C 3.8232770000 -4.5095130000 0.9930490000
H 4.4814300000 -5.3491350000 1.2249170000
C 3.7313450000 -3.4268050000 1.8710460000
H 4.3242610000 -3.4039460000 2.7875360000
C 2.8924490000 -2.3544130000 1.5576940000
H 2.8275780000 -1.4987660000 2.2331530000
C -0.8276420000 -3.5795570000 -1.4134570000

H -1.7435850000 -3.3138200000 -1.9595630000
H -0.6198390000 -4.6314380000 -1.6599440000
C -1.0813250000 -3.4349310000 0.1064140000
H -0.8604840000 -2.3979010000 0.3995520000
H -0.4005230000 -4.0805120000 0.6758400000
C -3.4302850000 -2.8337000000 0.2516990000
C -5.7976160000 -2.2588520000 0.7408630000
C -5.7349980000 -0.9180040000 0.3332810000
H -4.7828840000 -0.4860980000 0.0436730000
C -6.9012060000 -0.1519020000 0.2871240000
C -8.1370750000 -0.6830390000 0.6509180000
H -9.0408730000 -0.0773280000 0.6022250000
C -8.1906250000 -2.0153180000 1.0726560000
C -6.7716090000 1.2838290000 -0.1655840000
C -9.5059020000 -2.5962520000 1.5324980000

Syn-dimer **2b**

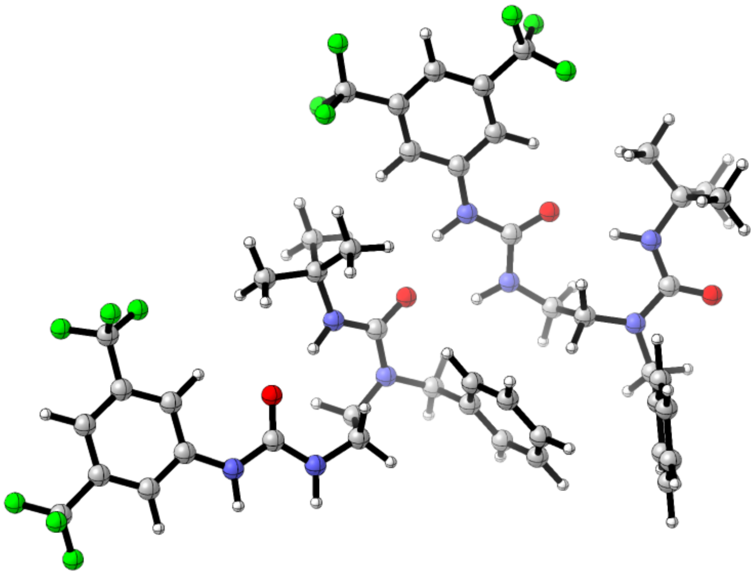

F 0.8202150000 7.3460500000 0.3544630000
F -0.3477570000 5.6238610000 0.9547270000
F -0.0761060000 6.0581380000 -1.1437120000
F 5.2269390000 6.1298080000 2.1775500000
F 6.1248960000 5.8726060000 0.2272260000
F 6.1390720000 4.2363940000 1.6413410000
O 4.7444040000 0.9460700000 -0.9030580000
O 7.9467520000 -2.9300980000 -0.1694550000
N 2.5933410000 1.7709480000 -1.0048380000
N 3.0474060000 -0.4199920000 -1.5503100000
N 5.6844140000 -2.8376710000 -0.4475990000
N 6.9209190000 -0.8820600000 -0.0750220000
C 1.6927620000 3.9347320000 -0.5240640000
H 0.7604720000 3.6204510000 -0.9872130000
C 1.7882340000 5.2075510000 0.0361030000
C 2.9776150000 5.6486200000 0.6168900000
H 3.0513420000 6.6429380000 1.0544930000
C 4.0715840000 4.7776080000 0.6203710000
C 3.9901610000 3.4931110000 0.0846970000
H 4.8456820000 2.8255190000 0.1055970000
C 2.7825060000 3.0504890000 -0.4890650000
C 0.5534800000 6.0692590000 0.0489850000
C 5.3939090000 5.2514420000 1.1720180000
C 3.5467730000 0.7755210000 -1.1344440000
C 3.9500320000 -1.5420170000 -1.7058100000
H 4.8334390000 -1.2108380000 -2.2727240000
H 3.4428300000 -2.3143100000 -2.3015110000
C 4.4286290000 -2.1236290000 -0.3570610000
H 4.5321220000 -1.3002050000 0.3629920000
H 3.6650210000 -2.7989350000 0.0508790000
C 6.9318570000 -2.2398920000 -0.2313480000
C 8.1377790000 -0.0901120000 0.1962800000
C 7.6851630000 1.3675920000 0.3604210000
H 7.1952930000 1.7378850000 -0.5520370000
H 8.5527870000 2.0090780000 0.5738320000
H 6.9714550000 1.4701500000 1.1921620000
C 9.1247820000 -0.1994060000 -0.9790510000
H 9.4270830000 -1.2451130000 -1.1225050000

H 10.0227800000 0.4097080000 -0.7883250000
H 8.6539190000 0.1638460000 -1.9065840000
C 8.7986600000 -0.5678150000 1.4993330000
H 8.0903800000 -0.4782810000 2.3383710000
H 9.6807240000 0.0518530000 1.7251720000
H 9.1108430000 -1.6160250000 1.4126210000
C 5.6494770000 -4.2698850000 -0.6439530000
H 6.6667500000 -4.5757410000 -0.9283150000
H 4.9771180000 -4.5164360000 -1.4851030000
C 5.2211480000 -5.0695050000 0.5739650000
C 5.2300050000 -4.5131570000 1.8578310000
H 5.5487430000 -3.4772990000 1.9863490000
C 4.8346130000 -5.2690260000 2.9655730000
H 4.8468420000 -4.8175040000 3.9608160000
C 4.4300520000 -6.5969650000 2.8055410000
H 4.1223080000 -7.1882710000 3.6713050000
C 4.4304700000 -7.1660530000 1.5269800000
H 4.1226320000 -8.2059800000 1.3905550000
C 4.8209780000 -6.4050520000 0.4239620000
H 4.8137080000 -6.8526870000 -0.5745400000
H 2.0365840000 -0.5205850000 -1.6262500000
H 1.6426570000 1.5151010000 -1.2791580000
H 6.1136120000 -0.3393430000 -0.3826760000
F -11.2904390000 -1.8578060000 2.1142760000
F -11.1700420000 -2.9341000000 0.2420920000
F -12.0323540000 -0.9437020000 0.3004070000
F -7.8573080000 2.7224230000 1.6550620000
F -9.1075630000 2.9514990000 -0.0949760000
F -6.9681890000 2.6648710000 -0.3159520000
O -4.4830720000 -0.8722750000 -0.2024090000
O 0.2184390000 0.2750850000 -1.6985480000
N -6.2467720000 -2.3261360000 0.0184730000
N -4.0887930000 -3.0916090000 -0.3872980000
N -1.1011160000 -1.5427200000 -2.0558090000
N -1.9962590000 0.4135530000 -1.1336810000
C -7.3080170000 -1.4247330000 0.1974320000
C -8.5931050000 -1.9588240000 0.3809370000
H -8.7445980000 -3.0403290000 0.3814650000
C -9.6909280000 -1.1158380000 0.5491840000
C -9.5359270000 0.2717150000 0.5341760000
H -10.3976330000 0.9288010000 0.6472380000
C -8.2545680000 0.7958520000 0.3565080000
C -7.1435800000 -0.0299580000 0.1880420000
H -6.1562620000 0.3942270000 0.0412940000
C -11.0542070000 -1.7143940000 0.7980710000
C -8.0459360000 2.2918990000 0.3953240000
C -4.9157320000 -2.0222250000 -0.1910910000
C -2.6696810000 -2.8495780000 -0.5938970000
H -2.1282680000 -3.7959210000 -0.4658630000
H -2.3083550000 -2.1646460000 0.1868750000
C -2.3764940000 -2.2239900000 -1.9764870000
H -2.4002870000 -3.0046060000 -2.7524180000
H -3.1782660000 -1.5152070000 -2.2242620000
C -0.9129330000 -0.2392430000 -1.6210200000
C -1.9917260000 1.8226810000 -0.6718260000
C -1.0635340000 1.9814730000 0.5447400000
H -1.4278600000 1.3610970000 1.3786240000
H -1.0384770000 3.0288040000 0.8792600000
H -0.0392820000 1.6684770000 0.3102580000
C -3.4304360000 2.1535370000 -0.2502850000
H -4.1323470000 2.0276240000 -1.0886300000
H -3.4846500000 3.1985710000 0.0865090000
H -3.7630170000 1.5061160000 0.5738080000

C -1.5887260000 2.7481080000 -1.8307310000
H -0.5993270000 2.4877130000 -2.2261380000
H -1.5742950000 3.7965650000 -1.4991550000
H -2.3142220000 2.6586360000 -2.6537370000
C 0.0644810000 -2.3258730000 -2.4347270000
H 0.8119720000 -1.6296830000 -2.8403830000
H -0.2217620000 -2.9925360000 -3.2629260000
C 0.6582520000 -3.1519200000 -1.3065910000
C 1.1563790000 -4.4369440000 -1.5611830000
H 1.1026430000 -4.8435780000 -2.5755040000
C 1.7178020000 -5.2030700000 -0.5361120000
H 2.1119440000 -6.1983160000 -0.7490260000
C 1.7802680000 -4.6984590000 0.7650630000
H 2.2345480000 -5.2914870000 1.5600090000
C 1.2841060000 -3.4188850000 1.0282940000
H 1.3445390000 -3.0091280000 2.0391330000
C 0.7345300000 -2.6482250000 0.0007350000
H 0.3713560000 -1.6425500000 0.2181400000
H -4.4478350000 -4.0365250000 -0.4276500000
H -6.5030700000 -3.3053090000 0.0566020000
H -2.8464770000 -0.1057010000 -0.9161950000

Method: B97-3c/def2-mTZVP (hydrogen atoms only)

Hexamer nanoring (**2a**)$_6$

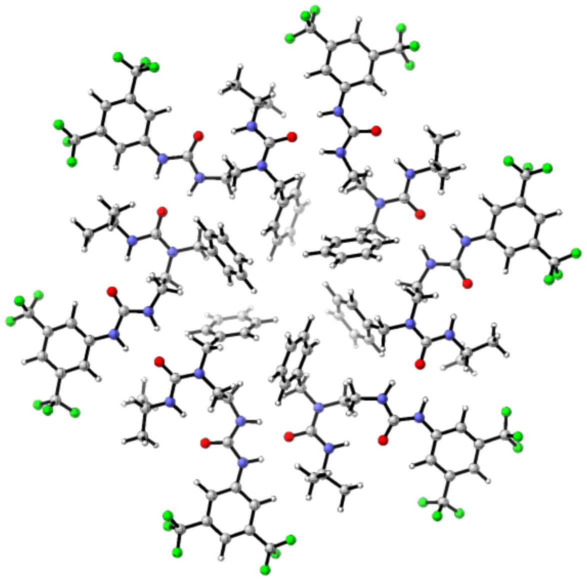

F 7.9355350000 21.5029340000 7.5677950000
F 8.5672030000 20.9964220000 9.5313750000
F 6.5837370000 21.5734370000 9.2868510000
F 3.3831000000 17.9543600000 9.1243270000
F 4.0568020000 15.9667000000 8.8472040000
F 3.6293540000 17.1862670000 7.1874230000
O 15.9572120000 17.3187290000 8.7607720000
O 10.8541760000 17.4366090000 7.8575200000
N 13.7816230000 17.9277800000 8.4732710000
H 12.8173270000 17.6582270000 8.3110460000
N 14.3332340000 15.7449200000 9.0430800000
N 10.9140970000 15.1765200000 7.6384380000
H 10.3722880000 14.3183010000 7.6303430000

N 8.9052070000 16.1974760000 7.8896260000
H 8.5634980000 15.2455120000 7.8197760000
C 6.6090620000 16.7656270000 8.1516800000
H 6.3754290000 15.7176430000 8.0368260000
C 12.9126160000 20.1710900000 8.2974130000
H 12.0980810000 19.8566520000 7.6483050000
H 13.1539770000 21.2049190000 8.0615480000
H 12.5611330000 20.1285470000 9.3262750000
C 16.1651330000 14.3508490000 6.8325130000
H 16.5379320000 15.3658220000 6.8642340000
C 14.1384110000 19.2881870000 8.0946350000
H 14.9262030000 19.6135160000 8.7727930000
C 14.6286650000 19.3535910000 6.6665340000
H 15.4978810000 18.7164120000 6.5179660000
H 14.9065960000 20.3713490000 6.3979470000
H 13.8441940000 19.0292170000 5.9834460000
C 14.7456960000 17.0120500000 8.7541010000
C 15.3129190000 14.6677290000 9.1880650000
H 14.9842390000 14.0271050000 10.0056830000
H 16.2507220000 15.1136340000 9.4944660000
C 15.4890960000 13.8333960000 7.9400200000
C 14.9805450000 12.5413030000 7.8693790000
H 14.4411450000 12.1315080000 8.7114300000
C 15.1562930000 11.7771070000 6.7272930000
H 14.7513710000 10.7739120000 6.6957530000
C 15.8540710000 12.2755660000 5.6593050000
H 16.0005530000 11.6730390000 4.7721200000
C 16.3504020000 13.5767450000 5.6993180000
H 16.8812860000 13.9813590000 4.8479550000
C 12.9277800000 15.3704360000 9.0319640000
H 12.3455540000 16.1348020000 9.5406870000
H 12.8224050000 14.4581360000 9.6119990000
C 12.3662830000 15.1488560000 7.6226320000
H 12.7387670000 15.9146260000 6.9462110000
H 12.6891550000 14.1878220000 7.2338030000
C 10.2727160000 16.3497480000 7.8029340000
C 7.9422890000 17.1794910000 8.0909220000
C 8.2336470000 18.5322410000 8.2615930000
H 9.2530740000 18.8703590000 8.2332730000
C 7.1869840000 19.4271230000 8.4846250000
C 5.8698040000 19.0261760000 8.5290810000
H 5.0782900000 19.7354710000 8.7086860000
C 5.5911500000 17.6750660000 8.3633500000
C 7.5352590000 20.8805030000 8.6476400000
C 4.1764140000 17.1927720000 8.3806380000
F 22.5898540000 22.2154480000 8.8982720000
F 22.4670390000 21.4151500000 6.9346940000
F 21.9750160000 23.4213900000 7.1792140000
F 17.2404840000 24.3836840000 7.3417280000
F 15.8559700000 22.8064120000 7.6188500000
F 16.6984180000 23.7863770000 9.2786320000
O 22.9770670000 13.1763690000 7.7053060000
O 20.5276330000 17.6546690000 8.6085490000
N 22.4167240000 15.3650100000 7.9928020000
H 21.7013820000 16.0655810000 8.1549550000
N 20.8021200000 13.7958710000 7.4229920000
N 18.6002990000 16.4727310000 8.8276270000
H 17.5861640000 16.5124650000 8.8357330000
N 18.4800280000 18.7229590000 8.5764370000
H 17.4847290000 18.5427280000 8.6454260000
C 17.8239890000 20.9955530000 8.3143800000
H 16.7996570000 20.6738400000 8.4294150000
C 23.9249850000 17.2392470000 8.1686630000

H 23.2445480000 17.7872880000 8.8169890000
H 24.9407130000 17.5471290000 8.4057650000
H 23.7136760000 17.5222800000 7.1395140000
C 20.5107630000 11.5123670000 9.6335610000
H 21.5761250000 11.6969790000 9.6018490000
C 23.7732660000 15.7362260000 8.3714420000
H 24.4488570000 15.2165670000 7.6932860000
C 24.0750310000 15.3443580000 9.7995450000
H 23.9557250000 14.2732400000 9.9482890000
H 25.0960190000 15.6106390000 10.0676710000
H 23.4031720000 15.8629770000 10.4828360000
C 22.1057170000 14.0722330000 7.7119740000
C 20.3590870000 12.4088420000 7.2780070000
H 19.6398200000 12.3730950000 6.4604970000
H 21.2140980000 11.8195710000 6.9715680000
C 19.7246200000 11.8391030000 8.5260520000
C 18.3513580000 11.6334750000 8.5966900000
H 17.7265250000 11.8955040000 7.7548080000
C 17.7774180000 11.0991760000 9.7387760000
H 16.7061020000 10.9489140000 9.7704070000
C 18.5579830000 10.7441140000 10.8067660000
H 18.1094450000 10.3161120000 11.6940170000
C 19.9330020000 10.9648670000 10.7667540000
H 20.5488210000 10.7073610000 11.6181220000
C 19.7750810000 14.8257880000 7.4341060000
H 20.1464180000 15.7121800000 6.9257140000
H 18.9323050000 14.4615920000 6.8534830000
C 19.3024340000 15.2012690000 8.8434350000
H 20.1520840000 15.2617120000 9.5195680000
H 18.6319320000 14.4410820000 9.2326810000
C 19.2956540000 17.6147970000 8.6631320000
C 18.8490200000 20.0478770000 8.3751400000
C 20.1662160000 20.4719290000 8.2044720000
H 20.9687310000 19.7581200000 8.2328310000
C 20.4178750000 21.8258070000 7.9814390000
C 19.4120540000 22.7660440000 7.9369790000
H 19.6305160000 23.8061630000 7.7573460000
C 18.1026330000 22.3318110000 8.1027080000
C 21.8506760000 22.2508810000 7.8184270000
C 16.9775840000 23.3158610000 8.0854180000
F 1.2254300000 8.4556640000 8.8982430000
F 1.9799200000 8.7494500000 6.9346660000
F 0.4884760000 7.3202250000 7.1791840000
F 2.0223700000 2.7388540000 7.3417060000
F 4.0805840000 2.3284660000 7.6188330000
F 2.8106820000 2.5680660000 9.2786110000
O 8.8598970000 13.3105390000 7.7052870000
O 6.2062900000 8.9501180000 8.6085290000
N 7.2446490000 11.7309480000 7.9927820000
H 6.9958990000 10.7612550000 8.1559460000
N 9.4108670000 11.1172280000 7.4229770000
N 8.1935450000 7.8719670000 8.8276130000
H 8.6660100000 6.9737370000 8.8365840000
N 6.3049280000 6.6426950000 8.5764200000
H 6.9585020000 5.8707710000 8.6456610000
C 4.6648230000 4.9382510000 8.3143610000
H 5.4556550000 4.2120380000 8.4294790000
C 4.8673830000 12.1000210000 8.1686370000
H 4.7324270000 11.2371910000 8.8174910000
H 4.0927840000 12.8259150000 8.4048190000
H 4.7283240000 11.7747210000 7.1396780000
C 11.5341140000 12.0066600000 9.6335500000
H 10.8413170000 12.8368080000 9.6021010000

C 6.2448950000 12.7201390000 8.3714190000
H 6.3568280000 13.5649960000 7.6932220000
C 6.4333790000 13.1774110000 9.7995210000
H 7.4200730000 13.6110240000 9.9481680000
H 5.6913540000 13.9273210000 10.0682210000
H 6.3216290000 12.3357290000 10.4824290000
C 8.5197320000 12.1079950000 7.7119560000
C 10.8335860000 11.4270640000 7.2779950000
H 11.2240630000 10.8220360000 6.4604420000
H 10.9165750000 12.4622820000 6.9718920000
C 11.6442250000 11.1624710000 8.5260420000
C 12.5089340000 10.0760050000 8.5966820000
H 12.5945040000 9.4039680000 7.7547000000
C 13.2586190000 9.8461090000 9.7387700000
H 13.9246240000 8.9935830000 9.7705120000
C 13.1758280000 10.6996310000 10.8067590000
H 13.7707640000 10.5251530000 11.6939990000
C 12.2971400000 11.7800550000 10.7667450000
H 12.2121400000 12.4420760000 11.6181320000
C 9.0324530000 9.7128280000 7.4340910000
H 8.0792870000 9.5911760000 6.9254350000
H 9.7693760000 9.1651780000 6.8536580000
C 8.9435960000 9.1157640000 8.8434210000
H 8.4664560000 9.8212490000 9.5197090000
H 9.9372740000 8.9148910000 9.2324370000
C 6.8568090000 7.9031290000 8.6631150000
C 4.9730190000 6.2997920000 8.3751200000
C 3.9471820000 7.2284910000 8.2044490000
H 4.1641320000 8.2804080000 8.2327900000
C 2.6488600000 6.7694950000 7.9814140000
C 2.3375010000 5.4283100000 7.9369550000
H 1.3274860000 5.0974620000 7.7573570000
C 3.3682690000 4.5114350000 8.1026870000
C 1.5643350000 7.7978000000 7.8183990000
C 3.0785810000 3.0450890000 8.0853970000
F 9.1696470000 -3.8790920000 7.5677940000
F 9.2924610000 -3.0787940000 9.5313730000
F 9.7844840000 -5.0850340000 9.2868530000
F 14.5190160000 -6.0473280000 9.1243390000
F 15.9035300000 -4.4700560000 8.8472170000
F 15.0610820000 -5.4500210000 7.1874350000
O 8.7824330000 5.1599870000 8.7607610000
O 11.2318670000 0.6816870000 7.8575180000
N 9.3427760000 2.9713460000 8.4732640000
H 10.0581180000 2.2707750000 8.3111110000
N 10.9573800000 4.5404850000 9.0430740000
N 13.1592000000 1.8636250000 7.6384400000
H 14.1733360000 1.8238910000 7.6303340000
N 13.2794720000 -0.3866030000 7.8896300000
H 14.2747710000 -0.2063720000 7.8206410000
C 13.9355110000 -2.6591980000 8.1516870000
H 14.9598430000 -2.3374850000 8.0366530000
C 7.8345150000 1.0971090000 8.2974040000
H 8.5149520000 0.5490680000 7.6490780000
H 6.8187870000 0.7892280000 8.0603020000
H 8.0458240000 0.8140760000 9.3265520000
C 11.2487370000 6.8239890000 6.8325060000
H 10.1833750000 6.6393760000 6.8642180000
C 7.9862340000 2.6001300000 8.0946250000
H 7.3106430000 3.1197900000 8.7727810000
C 7.6844690000 2.9919990000 6.6665220000
H 7.8037760000 4.0631160000 6.5177790000
H 6.6634810000 2.7257180000 6.3983960000

H 8.3563290000 2.4733790000 5.9832310000
C 9.6537830000 4.2641230000 8.7540930000
C 11.4004130000 5.9275140000 9.1880590000
H 12.1196800000 5.9632610000 10.0055690000
H 10.5454020000 6.5167850000 9.4944980000
C 12.0348800000 6.4972530000 7.9400150000
C 13.4081420000 6.7028810000 7.8693770000
H 14.0329750000 6.4408520000 8.7112580000
C 13.9820820000 7.2371800000 6.7272910000
H 15.0533980000 7.3874420000 6.6956600000
C 13.2015170000 7.5922420000 5.6593010000
H 13.6500550000 8.0202450000 4.7720500000
C 11.8264980000 7.3714890000 5.6993120000
H 11.2106790000 7.6289950000 4.8479440000
C 11.9844190000 3.5105680000 9.0319610000
H 11.6130830000 2.6241760000 9.5403530000
H 12.8271950000 3.8747640000 9.6125830000
C 12.4570660000 3.1350860000 7.6226310000
H 11.6074160000 3.0746420000 6.9464990000
H 13.1275670000 3.8952730000 7.2333850000
C 12.4638460000 0.7215590000 7.8029350000
C 12.9104800000 -1.7115210000 8.0909260000
C 11.5932840000 -2.1355740000 8.2615950000
H 10.7907690000 -1.4217650000 8.2332370000
C 11.3416250000 -3.4894510000 8.4846280000
C 12.3474460000 -4.4296890000 8.5290870000
H 12.1289840000 -5.4698080000 8.7087190000
C 13.6568670000 -3.9954550000 8.3633580000
C 9.9088240000 -3.9145250000 8.6476400000
C 14.7819160000 -4.9795050000 8.3806490000
F 30.5340700000 9.8806920000 7.5678230000
F 29.7795800000 9.5869060000 9.5314010000
F 31.2710240000 11.0161310000 9.2868830000
F 29.7371300000 15.5975020000 9.1243610000
F 27.6789160000 16.0078900000 8.8472340000
F 28.9488180000 15.7682900000 7.1874560000
O 22.8996030000 5.0258170000 8.7607800000
O 25.5532100000 9.3862380000 7.8575380000
N 24.5148510000 6.6054080000 8.4732840000
H 24.7636000000 7.5751010000 8.3101200000
N 22.3486330000 7.2191290000 9.0430890000
N 23.5659550000 10.4643890000 7.6384540000
H 23.0934900000 11.3626190000 7.6294820000
N 25.4545720000 11.6936610000 7.8896470000
H 24.8009980000 12.4655850000 7.8204050000
C 27.0946770000 13.3981050000 8.1517060000
H 26.3038450000 14.1243180000 8.0365880000
C 26.8921170000 6.2363350000 8.2974300000
H 27.0270730000 7.0991650000 7.6485760000
H 27.6667160000 5.5104410000 8.0612480000
H 27.0311750000 6.5616350000 9.3263890000
C 20.2253860000 6.3296970000 6.8325170000
H 20.9181830000 5.4995480000 6.8639660000
C 25.5146050000 5.6162170000 8.0946480000
H 25.4026720000 4.7713600000 8.7728450000
C 25.3261210000 5.1589440000 6.6665460000
H 24.3394270000 4.7253310000 6.5178990000
H 26.0681460000 4.4090340000 6.3978460000
H 25.4378700000 6.0006250000 5.9836380000
C 23.2397680000 6.2283600000 8.7541110000
C 20.9259140000 6.9092910000 9.1880710000
H 20.5354370000 7.5143170000 10.0056250000
H 20.8429260000 5.8740730000 9.4941730000

C 20.1152750000 7.1738850000 7.9400250000
C 19.2505660000 8.2603510000 7.8693840000
H 19.1649970000 8.9323880000 8.7113660000
C 18.5008810000 8.4902480000 6.7272970000
H 17.8348760000 9.3427740000 6.6955550000
C 18.5836720000 7.6367250000 5.6593080000
H 17.9887360000 7.8112030000 4.7720690000
C 19.4623600000 6.5563010000 5.6993210000
H 19.5473600000 5.8942800000 4.8479340000
C 22.7270470000 8.6235280000 9.0319760000
H 23.6802130000 8.7451800000 9.5406320000
H 21.9901240000 9.1711780000 9.6124090000
C 22.8159040000 9.2205920000 7.6226450000
H 23.2930440000 8.5151070000 6.9463570000
H 21.8222260000 9.4214640000 7.2336290000
C 24.9026910000 10.4332270000 7.8029520000
C 26.7864810000 12.0365640000 8.0909460000
C 27.8123180000 11.1078650000 8.2616180000
H 27.5953680000 10.0559480000 8.2332780000
C 29.1106400000 11.5668610000 8.4846530000
C 29.4219990000 12.9080460000 8.5291110000
H 30.4320140000 13.2388940000 8.7087080000
C 28.3912310000 13.8249210000 8.3633790000
C 30.1951650000 10.5385560000 8.6476680000
C 28.6809190000 15.2912670000 8.3806700000
F 23.8239650000 -3.1665780000 8.8982710000
F 23.1922970000 -2.6600660000 6.9346920000
F 25.1757630000 -3.2370810000 7.1792160000
F 28.3764000000 0.3819960000 7.3417400000
F 27.7026980000 2.3696560000 7.6188640000
F 28.1301460000 1.1500890000 9.2786440000
O 15.8022880000 1.0176270000 7.7052950000
O 20.9053240000 0.8997470000 8.6085480000
N 17.9778770000 0.4085760000 7.9927950000
H 18.9421730000 0.6781290000 8.1550200000
N 17.4262660000 2.5914360000 7.4229860000
N 20.8454030000 3.1598360000 8.8276290000
H 21.3872120000 4.0180550000 8.8357240000
N 22.8542930000 2.1388810000 8.5764410000
H 23.1960020000 3.0908440000 8.6462900000
C 25.1504380000 1.5707290000 8.3143870000
H 25.3840720000 2.6187130000 8.4292420000
C 18.8468840000 -1.8347340000 8.1686540000
H 19.6614190000 -1.5202960000 8.8177610000
H 18.6055230000 -2.8685630000 8.4045190000
H 19.1983670000 -1.7921920000 7.1397920000
C 15.5943670000 3.9855070000 9.6335540000
H 15.2215680000 2.9705340000 9.6018330000
C 17.6210890000 -0.9518310000 8.3714320000
H 16.8332970000 -1.2771610000 7.6932740000
C 17.1308350000 -1.0172350000 9.7995330000
H 16.2616180000 -0.3800570000 9.9481000000
H 16.8529040000 -2.0349940000 10.0681200000
H 17.9153060000 -0.6928610000 10.4826220000
C 17.0138040000 1.3243060000 7.7119660000
C 16.4465810000 3.6686270000 7.2780010000
H 16.7752610000 4.3092520000 6.4603840000
H 15.5087780000 3.2227220000 6.9716000000
C 16.2704040000 4.5029600000 8.5260470000
C 16.7789550000 5.7950530000 8.5966870000
H 17.3183550000 6.2048480000 7.7546360000
C 16.6032070000 6.5592490000 9.7387740000
H 17.0081290000 7.5624440000 9.7703140000

C 15.9054290000 6.0607890000 10.8067620000
H 15.7589480000 6.6633160000 11.6939470000
C 15.4090980000 4.7596110000 10.7667480000
H 14.8782140000 4.3549970000 11.6181110000
C 18.8317200000 2.9659200000 7.4341030000
H 19.4139460000 2.2015540000 6.9253800000
H 18.9370960000 3.8782200000 6.8540680000
C 19.3932170000 3.1875000000 8.8434340000
H 19.0207330000 2.4217300000 9.5198550000
H 19.0703450000 4.1485340000 9.2322630000
C 21.4867840000 1.9866070000 8.6631330000
C 23.8172110000 1.1568650000 8.3751440000
C 23.5258530000 -0.1958850000 8.2044740000
H 22.5064260000 -0.5340040000 8.2327950000
C 24.5725160000 -1.0907670000 7.9814420000
C 25.8896960000 -0.6898200000 7.9369850000
H 26.6812100000 -1.3991150000 7.7573790000
C 26.1683500000 0.6612890000 8.1027160000
C 24.2242410000 -2.5441470000 7.8184270000
C 27.5830860000 1.1435840000 8.0854290000

[1] H. Jiang, L. Sun, S. Yuan, W. Lu, W. Wan, S. Zhu and J. Hao *Tetrahedron* 2012, **68**, 2858 – 2863

[2] R. Evans, The Interpretation of Small Molecule Diffusion Coefficients: Quantitative Use of Diffusion-Ordered NMR Spectroscopy. *Prog. Nucl. Magn. Reson. Spectrosc.* 2020, **117**, 33–69.

[3] A. Jerschow and N. Müller, Suppression of Convection Artifacts in Stimulated-Echo Diffusion Experiments. Double-Stimulated-Echo Experiments. *J. Magn. Reson.* 1997, **125**, 372–375.

[4] N. A. Espinosa-Jalapa, N. Berg, M. Seidl, I. G. Shenderovich, R. M. Gschwind, and J. O. Bauer, Complexation Behaviour of LiCl and LiPF6 – Model Studies in the Solid-State and in Solution Using a Bidentate Picolyl-Based Ligand. *Chem. Commun.,* 2020, **56**, 13335–13338.

[5] C. A. Crutchfield, D. J. Harris, Molecular Mass Estimation by PFG NMR Spectroscopy. *J. Magn. Reson.* 2007, **185**, 179–182.

[6] A. Macchioni, G. Ciancaleoni, C. Zuccaccia and D. Zuccaccia, Determining Accurate Molecular Sizes in Solution through NMR Diffusion Spectroscopy. *Chem. Soc. Rev.* 2008, **37**, 479–489.

[7] Gaussian. *Gaussian 16, Revis. C.01, M. J. Frisch, G. W. Truck. H. B. Schlegel, G. E. Scuseria, M. A. Robb, J. R. Cheeseman, G. Scalmani, V. Barone, G. A. Petersson, H. Nakatsuji, X. Li, M. Caricato, A. V. Marenich, J. Bloino, B. G. Janesko, R. Gomperts, B. Mennu*.

[8] A. D. Becke, Density-functional Thermochemistry. III. The Role of Exact Exchange. *J. Chem. Phys.* 1993, **98**, 5648–5652.

[9] C. Lee, W. Yang and R. G. Parr, Development of the Colle-Salvetti Correlation-Energy Formula into a Functional of the Electron Density. *Phys. Rev. B* 1988, **37**, 785–789.

[10] S. Grimme, Density Functional Theory with London Dispersion Corrections. *Wiley Interdiscip. Rev. Comput. Mol. Sci.* 2011, **1**, 211–228.

[11] F. Weigend and R. Ahlrichs, Balanced Basis Sets of Split Valence, Triple Zeta Valence and Quadruple Zeta Valence Quality for H to Rn: Design and Assessment of Accuracy. *Phys. Chem. Chem. Phys.* 2005, **7**, 3297–3305..

[12] S. Grimme, Supramolecular Binding Thermodynamics by Dispersion-Corrected Density Functional Theory. *Chem. - A Eur. J.* 2012, **18**, 9955–9964.

[13] G. Luchini, J. V. Alegre-Requena, I. Funes-Ardoiz and R. S. Paton, GoodVibes: Automated Thermochemistry for Heterogeneous Computational Chemistry Data. *F1000Research* 2020, **9** (May), 291..

[14] J. G. Brandenburg, C. Bannwarth, A. Hansen and S. Grimme, B97-3c: A Revised Low-Cost Variant of the B97-D Density Functional Method. *J. Chem. Phys.* 2018, **148**, 064104.

[15] F. Neese, F. Wennmohs, U. Becker and C. Riplinger, The ORCA Quantum Chemistry Program Package. *J. Chem. Phys.* 2020, **152**, 224108.

[16] A. V. Marenich, C. J. Cramer and D. G. Truhlar, Universal Solvation Model Based on Solute Electron Density and on a Continuum Model of the Solvent Defined by the Bulk Dielectric Constant and Atomic Surface Tensions. *J. Phys. Chem. B* 2009, **113**, 6378–6396.

[17] A. Altun, F. Neese and G. Bistoni, HFLD: A Nonempirical London Dispersion-Corrected Hartree-Fock Method for the Quantification and Analysis of Noncovalent Interaction Energies of Large Molecular Systems *J. Chem. Theory Comput.* 2019, **15**, 5894–5907.

[18] D. G. Liakos, M. Sparta, M. K. Kesharwani, J. M. L. Martin and F. Neese, Exploring the Accuracy Limits of Local Pair Natural Orbital Coupled-Cluster Theory. *J. Chem. Theory Comput.* 2015, **11**, 1525–1539.

[19] C. Riplinger, B. Sandhoefer, A. Hansen and F. Neese, F. Natural Triple Excitations in Local Coupled Cluster Calculations with Pair Natural Orbitals. *J. Chem. Phys.* 2013, **139**.

[20] G. Bistoni, Finding Chemical Concepts in the Hilbert Space: Coupled Cluster Analyses of Noncovalent Interactions. *WIREs Comput. Mol. Sci.* 2020, **10**, e1442.

[21] W. B. Schneider, G. Bistoni, G., M. Sparta, M. Saitow, C. Riplinger, A. A. Auer and F. Neese, Decomposition of Intermolecular Interaction Energies within the Local Pair Natural Orbital Coupled Cluster Framework. *J. Chem. Theory Comput.* 2016, **12**, 4778–4792.

[22] M. Žabka, R. M. Gschwind, Ternary Complexes of Chiral Disulfonimides in Transfer-Hydrogenation of Imines: The Relevance of Late Intermediates in Ion Pair Catalysis. *Chem. Sci.* 2021, **12**, 15263–15272.

[23] E. R. Johnson, S. Keinan, P. Mori-Sánchez, J. Contreras-García, A. J. Cohen and W. Yang, Revealing Noncovalent Interactions. *J. Am. Chem. Soc.* 2010, **132**, 6498–6506.

[24] R. Laplaza, F. Peccati, R. Boto, C. Quan, A. Carbone, J. P. Piquemal, Y. Maday and J. Contreras-García, J. NCIPLOT and the Analysis of Noncovalent Interactions Using the Reduced Density Gradient. *Wiley Interdiscip. Rev. Comput. Mol. Sci.* 2020, No. March, 1–18.
